# MicroRNA93 Regulates Proliferation and Differentiation of Normal and Malignant Breast Stem Cells

**DOI:** 10.1371/journal.pgen.1002751

**Published:** 2012-06-07

**Authors:** Suling Liu, Shivani H. Patel, Christophe Ginestier, Ingrid Ibarra, Rachel Martin-Trevino, Shoumin Bai, Sean P. McDermott, Li Shang, Jia Ke, Sing J. Ou, Amber Heath, Kevin J. Zhang, Hasan Korkaya, Shawn G. Clouthier, Emmanuelle Charafe-Jauffret, Daniel Birnbaum, Gregory J. Hannon, Max S. Wicha

**Affiliations:** 1 Comprehensive Cancer Center, Department of Internal Medicine, University of Michigan, Ann Arbor, Michigan, United States of America; 2 Centre de Recherche en Cancérologie de Marseille, Laboratoire d'Oncologie Moléculaire, UMR891 Inserm/Institut Paoli-Calmettes, Université de la Méditerranée, Marseille, France; 3 Program in Genetics and Bioinformatics, Cold Spring Harbor Laboratory, Cold Spring Harbor, New York, United States of America; Baylor College of Medicine, United States of America

## Abstract

MicroRNAs (miRNAs) play important roles in normal cellular differentiation and oncogenesis. microRNA93 (mir-93), a member of the mir106b-25 cluster, located in intron 13 of the MCM7 gene, although frequently overexpressed in human malignancies may also function as a tumor suppressor gene. Using a series of breast cancer cell lines representing different stages of differentiation and mouse xenograft models, we demonstrate that mir-93 modulates the fate of breast cancer stem cells (BCSCs) by regulating their proliferation and differentiation states. In “claudin^low^” SUM159 cells, expression of mir-93 induces Mesenchymal-Epithelial Transition (MET) associated with downregulation of TGFβ signaling and downregulates multiple stem cell regulatory genes, including JAK1, STAT3, AKT3, SOX4, EZH1, and HMGA2, resulting in cancer stem cell (CSC) depletion. Enforced expression of mir-93 completely blocks tumor development in mammary fat pads and development of metastases following intracardiac injection in mouse xenografts. The effect of mir-93 on the CSC population is dependent on the cellular differentiation state, with mir-93 expression increasing the CSC population in MCF7 cells that display a more differentiated “luminal” phenotype. mir-93 also regulates the proliferation and differentiation of normal breast stem cells isolated from reduction mammoplasties. These studies demonstrate that miRNAs can regulate the states and fates of normal and malignant mammary stem cells, findings which have important biological and clinical implications.

## Author Summary

Recent evidence suggests that many cancers, including those of the breast, are maintained by a population of cancer cells that display stem cell properties. These “cancer stem cells” may also contribute to tumor metastasis, treatment resistance, and relapse. Recently, miRNAs (small non-coding RNAs) have been reported to be capable of functioning as oncogenes or tumor suppressors. miRNA93 (mir-93) is frequently overexpressed in human cancer but, paradoxically, has been found to function as a tumor suppressor in some contexts. Using a series of breast cancer cell lines representing different stages of differentiation and mouse xenograft models, we demonstrate that mir-93 modulates the fate of breast cancer stem cells by regulating their proliferation and differentiation states. In less differentiated tumors, enforced expression of mir-93 completely blocks tumor development in mammary fat pads and development of metastases following intracardiac injection in mouse xenografts by reducing breast cancer stem cells. However, the effect of mir-93 on the cancer stem cell population is dependent on the cellular differentiation state, with mir-93 expression increasing the cancer stem cell population in more differentiated breast tumors. These studies demonstrate that miRNAs can regulate breast stem cell proliferation and differentiation, an observation with important biological and clinical implications.

## Introduction

miRNAs serve vital functions in many of normal developmental processes, as well as in carcinogenesis. A number of these miRNAs have been shown to function as oncogenes with increased expression in lung cancer, prostate cancer and colorectal cancer [1], [2], [3], [4], [5], [6], [7], [8]. In contrast, other miRNAs such as Let7 are frequently downregulated in malignancies including breast cancer and lung cancer in these contexts functioning as a tumor suppressor gene [9], [10], [11]. The mir106b-25 cluster is composed of the highly conserved miRNA106b (mir-106b), miRNA93 (mir-93) and miRNA25 (mir-25) that have been reported to be overexpressed in a number of cancers including gastric, prostate and pancreatic neural endocrine tumors, neuroblastoma and multiple myeloma [1], [2], [3]. These miRNAs are located in a 515-base region on chromosome band 7q22 in intron13 of the host MCM7 gene where they are co-transcribed in the context of MCM7 primary transcripts [1]. MCM7 is a DNA licensing factor obligate for cellular replication. Studies have suggested that the mir-106b-25 miRNA cluster functions as a proto oncogene. Several studies suggest that a primary mechanism of oncogenesis involves targeting of PTEN which cooperates with MCM7 to drive cellular proliferation [12]. Despite evidence for this miRNA cluster functioning as a proto oncogene, in some contexts it has been reported to function as a tumor suppressor inhibiting tumor growth [13]. The molecular mechanisms accounting for this discrepancy have not been determined.

Studies associating miRNA expression with oncogenesis have largely been performed in bulk tumor populations. However, there is substantial evidence supporting the CSC hypothesis which suggests that tumors are hierarchically organized and that many tumors, including those of the breast, are maintained by a subpopulation of cells that displays stem cell properties [14], [15], [16]. These cells may mediate invasion and metastasis and contribute to treatment resistance [17]. miRNAs have also been found to play important roles in normal and malignant stem cell function. Silber et al, reported that mir-124 and mir-137 induced differentiation of neural and glioblastoma stem cells, a state associated with cell cycle arrest [18]. Furthermore, recent studies have shown that the miRNAs Let7 and mir-200c regulate self-renewal of BCSCs [10], [19]. Stem cell regulatory genes such as BMI-1 and HMGA2 may mediate this process [10], [19]. We have previously demonstrated that normal breast tissue, primary breast cancers and breast cancer cell lines contain subpopulations with stem cell properties that can be enriched by virtue of their expression of aldehyde dehydrogenase (ALDH) as assessed by the Aldefluor assay (Stem Cell Technologies, Inc., Vancouver, British Columbia) or by tumor initiation in NOD/SCID mice [20]. Recently, Ibarra, et al, showed that Let7, as well as mir-93 are highly depleted in mouse mammary stem/progenitor cells isolated with the stem cell marker ALDH [21]. We have utilized breast cancer cell lines representing different molecular subtypes of breast cancer as well as primary xenografts of breast cancer and normal mammary cells to examine the role of mir-93 in the regulation of normal and malignant breast stem cells. We demonstrate that this miRNA is able to regulate stem cell fate including cellular proliferation and differentiation. These studies suggest that miRNAs regulate the transition between CSC states findings which have important biological and clinical implications.

## Results

### Tumor initiating capacity is associated with low mir-93 expression

**Figure 1:**
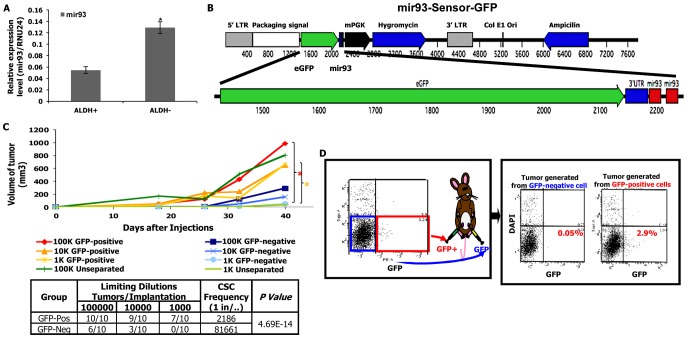
mir-93-negative SUM159 cells have increased tumor-initiating capacity. A. ALDH^+^ cells from SUM159 cells shows lower mir-93 expression level in comparison to ALDH^−^ cells as accessed by qRT-PCR. P<0.05; Error bars represent mean ± STDEV. B. A schematic of mir-93-Sensor-GFP lentiviral construct; C. SUM159 cells were transduced with the mir-93-sensor-GFP lentivirus and selected with hygromycin B, and cells were sorted based on the GFP expression. A serial dilution of mir-93-negative (sensor/GFP-positive) SUM159 cells and mir-93-positive (sensor/GFP-negative) SUM159 cells were injected into the 4^th^ fatpads of NOD/SCID mouse. *p<0.05. D. mir-93-negative cells gave rise to tumors containing both mir-93-negative and mir-93-positive cell populations, but mir-93-positive cells only gave rise to tumors containing mir-93-positive cell populations.

We have previously demonstrated that primary human breast cancers and established breast cancer cell lines contain subpopulations with stem cell properties that can be isolated by virtue of their expression of ALDH as assessed by the Aldefluor assay. These cells displayed increased tumor initiating capacity and metastatic potential compared to corresponding Aldefluor-negative cells [22]. mir-93 was shown as one of the most abundant miRNAs in ALDH^−^ cells [21]. As assessed by qRT-PCR, mir-93 expression was significantly increased in the ALDH^−^ compared to ALDH^+^ populations in SUM159 claudin^low^ and HCC1954 basal subtype of human breast cancer (Figure 1A and Figure S1A). As shown in Figure S3, mir-93 expression was lower in CSCs which were characterized by their expression of the CSC markers: ALDH^+^ or CD24^−^CD44^+^. To determine the relationship between mir-93 expression and tumor initiating capacity, we constructed a mir-93 sensor tagged with GFP (mir-93-sensor-GFP) containing a mir-93 target UTR coupled to GFP. In cells transfected with this vector, mir-93 expression results in degradation of GFP mRNA (sensor-negative), whereas mir-93-negative cells express GFP (sensor-positive) (Figure 1B). mir-93 expression was significantly higher in GFP-negative cells than GFP-positive cells (Figure S4) and the ALDH1A1 was much lower in GFP-negative cells than GFP-positive cells as accessed by western blot or immunohistochemical staining (Figure S5). Furthermore, GFP was significantly reduced by overexpression of mir-93 (Figure S6), demonstrating that the sensor reports mir-93 function. The relationship between mir-93 expression and tumor initiation was determined by introducing serial dilutions of sensor-positive (mir-93-negative) and sensor-negative (mir-93-positive) SUM159 cells into the mammary fatpads of NOD/SCID mice. As shown in Figure 1C, sensor-positive (mir-93-negative) cells had significantly higher tumor initiating capacity and CSC frequency than sensor-negative (mir-93-positive) cells. Moreover, mir-93-negative cells gave rise to tumors containing both mir-93-negative and mir-93-positive populations, whereas mir-93-positive cells gave rise only to small, slow growing tumors containing exclusively mir-93-positive populations (Figure 1D). Similar findings were seen using HCC1954 cells (data not shown). These studies demonstrated that in these breast cancer cell lines low mir-93 expression is associated with the CSC phenotype characterized by increased aldehyde dehydrogenase expression, tumor initiating capacity and the ability to generate heterogeneous tumors containing both stem cell and non-stem cell populations.

### mir-93 overexpression decreases CSCs *in vitro*

**Figure 2:**
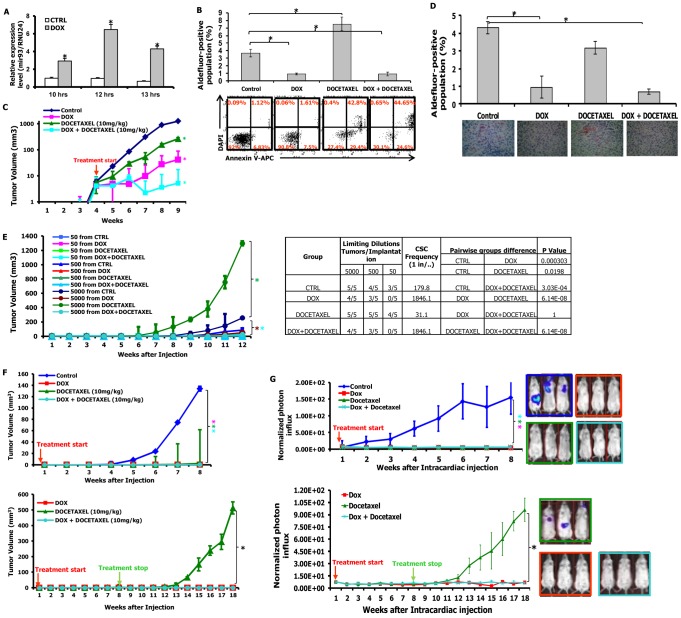
mir-93 inhibits tumor growth and metastasis by decreasing CSCs in SUM159 cells. A. SUM159 cells were transduced with the pTRIPZ-mir-93 lentivirus and selected with Puromycin for 7 days. Tetracycline (DOX) induces mir-93 expression in suspension-cultured SUM159 cells by 10 hours; B. 1×10^6^ SUM159 cells or pTRIPZ-SUM159 -mir-93 cells were plated in T75 flasks and, after overnight, the cells were treated with Vehicle control, with (DOX) or without (CTRL) DOX (1 ug/ml), docetaxel (10 nM) or the combination for 7 days. Cells were utilized for Aldefluor assay and stained for Annexin V-APC and DAPI for apoptosis assay. C. 100 k pTRIPZ-SUM159-mir-93 cells were injected into the 4^th^ fatpads of NOD/SCID mice. The treatment started as indicated by the red arrow. DOX alone (1 mg/ml in drinking water), or docetaxel (10 mg/kg i.p. once weekly) alone, or the combination inhibits SUM159 tumor growth in vivo (note: The Y-axis is on a logarithmic scale). D. Tumors from each group were collected. ALDH was accessed by the Aldefluor assay on viable dissociated cells and by ALDH1 immunohistochemistry on fixed sections. E. Serial dilutions of cells obtained from these xenografts were implanted in the 4^th^ fatpads of secondary mice, which received no further treatment. F. 10k pTRIPZ-SUM159-mir-93 cells were injected into the 4^th^ fatpads of NOD/SCID mice. The treatment started immediately after injection as indicated by the red arrow and stopped as indicated by the green arrow. G. 200k pTRIPZ-SUM159-mir-93-Luc cells in 100 ul of PBS were injected into the left ventricle of NOD/SCID mice. The treatment started immediately after injection as indicated by the red arrow and stopped as indicated by the green arrow. Metastasis formation was monitored using bioluminescence imaging. Quantification of the normalized photon flux, measured at weekly intervals following inoculation. *p<0.05; Error bars represent mean ± STDEV. The colored “*” on the side of the tumor growth curve indicates that the tumor growth or metastasis is significantly different between the control group and the group with the same colored curve.

We utilized a tetracycline (TET) inducible mir-93 construct tagged with RFP (pTRIPZ-mir-93-RFP) to determine the functional role of mir-93 in CSCs. mir-93 levels were significantly increased by ten hours following tetracycline induction in these cells (Figure 2A). Induction of mir-93 was associated with a significant decrease in the CSC population as assessed by the Aldefluor assay (Figure 2B), which were also seen in two basal breast cancer cell lines HCC1954 and SUM149 (Figure S1B and Figure S7). Furthermore, this decrease did not result from induction of apoptosis in these cells as assessed by Annexin V staining (Figure 2B). Our group and others have previously shown that CSCs were relatively resistant to cytotoxic chemotherapy. Consistent with this, addition of the cytotoxic agent docetaxel resulted in a relative increase in the percentage of Aldefluor-positive cells (Figure 2B), an increase associated with induction of apoptosis in the bulk cell population (42.8% versus 1.1% control) (Figure 2B). The relative increase in the Aldefluor-positive population seen with docetaxel treatment was abrogated by simultaneous mir-93 expression (Figure 2B). These experiments suggested that unlike cytotoxic agents which primarily target the bulk cell population, mir-93 overexpression was able to reduce the CSC population. Moreover, this did not appear to result from increased CSC apoptosis suggesting a potential role for mir-93 in promoting differentiation of CSCs. Furthermore, since the TET-inducible mir-93 system allows for the controlled regulation of CSC populations, it provides a valuable tool for assessing the role of CSCs in tumor growth in mouse xenograft models. Furthermore, the ability to regulate the CSC population during different phases of tumor growth allows for the assessment of the role of these cells in tumor initiation and maintenance.

### mir-93 overexpression inhibits the growth of established tumor xenografts

We first determined the effect of mir-93 induction on the growth of established tumors and compared these effects to those of cytotoxic chemotherapy. When tumors reached 0.2–0.3 cm in diameter, we induced mir-93 (with doxycycline treatment, hereafter DOX) or initiated cytotoxic chemotherapy with docetaxel or the combination. Induction of mir-93 significantly inhibited the growth of SUM159 and HCC1954 xenografts (Figure 2C and Figure S1C). Furthermore, induction of mir-93 further reduced tumor growth when added to the docetaxel chemotherapy (Figure 2C and Figure S1). Following five weeks of treatment, animals were sacrificed and CSC populations were assessed by the Aldefluor assay and ALDH1 immunohistochemistry. Induction of mir-93 alone or in combination with docetaxel reduced the Aldefluor-positive population by more than 60% compared to control or docetaxel alone (Figure 2D and Figure S1D). These observations were confirmed by immunohistochemistry of ALDH1 expression (Figure 2D and Figure S1D). mir-93 expression was significantly higher in DOX group compared to the control group at the end of treatment (Figure S2). To provide a more definitive assessment of CSCs, we determined the ability of serial dilutions of cells obtained from primary tumors to form tumors in secondary NOD/SCID mice. Tumor cells isolated from docetaxel treated mice, initiated tumors at lower concentrations with accelerated growth compared to control animals (Figure 2E, Figure S1E). This was consistent with previous studies demonstrating a relative increase in CSCs following chemotherapy [17]. In contrast, cells isolated from tumors with mir-93 induction with or without docetaxel chemotherapy had markedly reduced tumor initiating capacity in secondary mice with no tumors observed from introduction of fifty cells from the mir-93 docetaxel treated group (Figure 2E, Figure S1E). The CSC frequency was lower in the groups of DOX alone and DOX+docetaxel, and was significantly increased in the docetaxel group (Figure 2E and Figure S1E). These studies demonstrated that mir-93 induction reduced the CSC population reducing growth of established tumor xenografts.

In order to determine whether down-regulation of mir-93 promoted tumorigenesis, we utilized a mirZip anti-sense miRNA in SUM159 cells. qRT-PCR was utilized to confirm the efficient knock-down of mir-93 (Figure S8A). ALDH^+^ cells were significantly increased after mir-93 was knocked down (mirZip93-DsRed) (Figure S8B). As shown in Figure S8C, knockdown of mir-93 significantly promoted the growth of SUM159 cells in tumor xenografts and increased the CSC frequency. Furthermore, the proportion of ALDH^+^ cells were significantly increased after mir-93 was knocked down (mirZip93-DsRed) (Figure S8D).

### mir-93 expression in the adjuvant setting prevents tumor growth

Preclinical models have suggested that CSCs play a role in tumor recurrence and metastasis following adjuvant therapy [23]. This suggests that targeting of CSCs may have more dramatic effects in the adjuvant than in the advanced tumor settings. To simulate the adjuvant setting we induced mir-93 and/or administered docetaxel immediately after tumor cell implantation. Although tumors grew after four to five weeks in control animals, there was no observed tumor growth following mir-93 induction and/or docetaxel treatments for eight weeks (Figure 2F, Figure S1F). After eight weeks, treatments were stopped and animals observed for an additional ten weeks. In SUM159 xenografts, tumors developed in all mice who received eight weeks of docetaxel alone. In contrast, no tumors developed in mice following mir-93 induction with or without docetaxel (Figure 2F, Figure S1F). In order to extend these observations to primary breast tumors, we examined the effect of mir-93 induction on three primary breast xenografts, MC1 (Figure S10A), UM2 (Figure S10B) and UM1 (Figure S10C) which were directly established from patient tumors and not passaged *in vitro*. MC1 and UM1 were derived from claudin^low^ and UM2 from a basal breast carcinoma. Induction of mir-93 upon cell implantation completely prevented tumor growth in this model. Together these studies suggested that mir-93 regulated the CSC population and that this population mediates tumor growth following adjuvant therapy.

### mir-93 prevents tumor metastasis in the adjuvant setting

Previous studies have demonstrated that CSCs mediate tumor invasion and metastasis. To determine the effect of mir-93 expression on tumor invasion, we examined the effect of mir-93 induction and/or downregulation on invasion of SUM159 cells using a matrigel invasion assay. Overexpression of mir-93 significantly inhibited the ability of SUM159 cells to invade in this assay (Figure S8E). In contrast, knockdown of mir-93 utilizing the mirZip93-DsRed promoted tumor invasion (Figure S8F).

To determine whether the expression of mir-93 affect the growth of tumor metastasis *in vivo*, SUM159 (Figure 2G) and HCC1954 (1G) cells co-transfected with the inducible mir-93 vector and luciferase were introduced into NOD/SCID mice by intracardiac injection and metastasis formation monitored by bioluminescence imaging. DOX and/or docetaxel treatments were initiated following intracardiac injection. As shown in Figure 2G, mir-93 induction completely suppressed whereas docetaxel partially suppressed metastasis formation. Metastasis was confirmed by histologic examination with pan-cytokeratin staining (Figures S9, S1H). Treatments were stopped at eight weeks and animals were observed for an additional ten weeks for development of metastasis. In animals receiving docetaxel alone, metastasis rapidly developed following cessation of therapy. In contrast, no metastases developed in mice following mir-93 induction with or without docetaxel chemotherapy (Figure 2G). In animals injected with HCC1954 cells, animals from all groups developed metastasis following cessation of therapy. However, development of metastasis were delayed and reduced in mice following mir-93 induction with or without docetaxel chemotherapy (Figure S1G).

### mir-93 overexpression increases the CSC population and accelerates tumor growth in luminal subtype MCF7 cells

**Figure 3:**
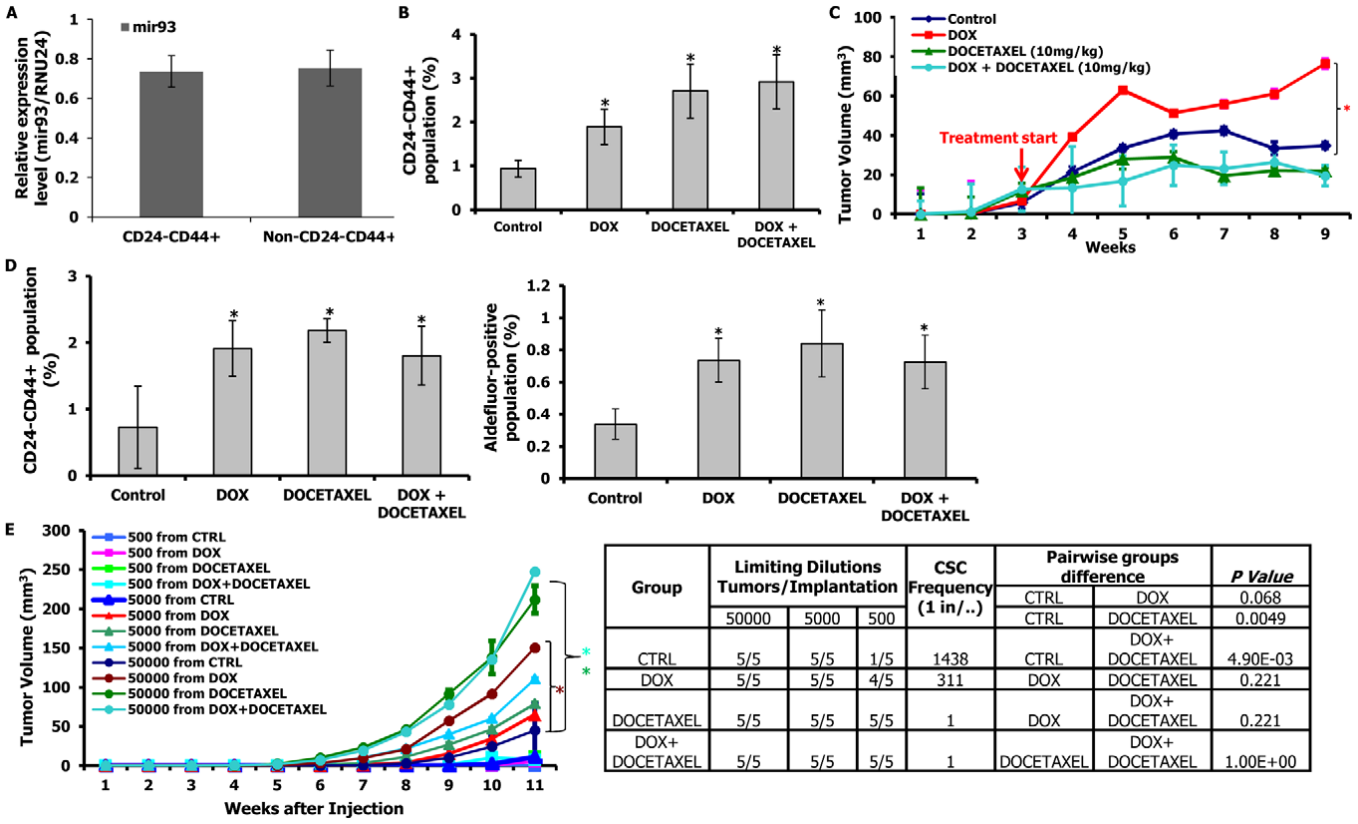
mir-93 promotes tumor growth by increasing CSCs in MCF7 cells. A. Mir-93 is expressed equally in CD24^−^CD44^+^ and bulk (non-CD24^−^CD44^+^) populations of MCF7 cells. B. 1×10^6^ pTRIPZ-MCF7-mir-93 cells were plated in T75 flasks and, after overnight, the cells were treated with Vehicle control, DOX (1 ug/ml), docetaxel (10 nM) or the combination for 7 days. DOX alone, docetaxel alone or the combination increased the CD24^−^CD44^+^ population *in vitro*. C. 1000k pTRIPZ-MCF7-mir-93 cells were injected into the 4^th^ fatpads of NOD/SCID mice. Treatment was initiated as indicated by the red arrow. DOX alone (1 mg/ml in drinking water) promoted MCF7 tumor growth in vivo; docetaxel (10 mg/kg i.p. once weekly) alone or the combination inhibits MCF7 tumor growth in vivo. D. Tumors from each group were collected. Analysis for CD24 and CD44 was performed on dissociated cells. DOX alone, docetaxel alone, or the combination increased the CD24^−^CD44^+^ populations in MCF7. E. Serial dilutions of cells obtained from these xenografts were implanted in the 4^th^ fatpads of secondary mice, which received no further treatment. Cells from DOX-, docetaxel-, or combination-treated tumors formed secondary tumors at all dilutions (50000, 5000, 500), whereas only higher numbers of cells (50000, 5000) obtained from control xenografts were able to generate tumors. *p<0.05; Error bars represent mean ± STDEV. The colored “*” on the side of the tumor growth curve indicates that tumor growth is significantly different between the control group and the group with the same colored curve.

Human breast cancer represents a heterogeneous set of diseases with distinct molecular profiles and clinical behaviors [24]. These subtypes may represent different cells of origin and/or differentiation state. It has been proposed that the most undifferentiated “claudin^low^” tumors originate from and resemble normal mammary stem cells, whereas the triple-negative basal tumors arise from a more differentiated luminal progenitor cell and the most differentiated luminal tumors which express estrogen and progesterone receptors originate from and are composed of the most differentiated cells [24]. To determine the relationship between mir-93 expression and level of cellular differentiation, we compared the expression of mir-93 in claudin^low^ (SUM159), basal (HCC1954) and luminal (MCF7) cells. As shown in Figure S11, mir-93 levels correlate with postulated differentiation state of these cell lines. Furthermore, in the claudin^low^ SUM159 cells and basal HCC1954 cells, mir-93 expression is significantly lower in Aldefluor-positive as compared to Aldefluor-negative populations (Figure S11). In contrast, the CSC population in MCF7 cells characterized by the phenotype CD24^−^CD44^+^ [25] expressed the same high level of mir-93 as did the other (non-stem) cells constituting the bulk population (Figure 3A, Figure S11). This suggests that mir-93 may play a different role in more differentiated luminal breast cancer than in the more undifferentiated claudin^low^ and basal subtype. Consistent with this, induction of mir-93 in MCF7 cells increased the CD24^−^CD44^+^ population (Figure 3B). Docetaxel also increased this population, as did the combination of mir-93 plus docetaxel (Figure 3B). In xenografts, induction of mir-93 accelerated the growth of MCF7 xenografts compared to control (Figure 3C), findings which were confirmed using two additional luminal cell lines MDA-MB-453 and T47D (Figures S12A and S13A). In contrast, docetaxel reduced tumor growth (Figure 3C). Analysis of treated MCF7 tumors confirmed that mir-93 induction increased the proportion of CD24^−^CD44^+^ cells and ALDH^+^ cells in tumors as did docetaxel or DOX plus docetaxel (Figure 3D). mir-93 expression level was significantly higher in DOX group compared to the control group at the end of treatment (Figure S14). mir-93 induction increased the proportion of ALDH^+^ cells from 1.01% to 9.5% in MDA-MB-453 tumors (Figure S12B) and from 1.26% to 3.84% in T47D tumors (Figure S13B). Furthermore, the calculated tumor initiating frequency was significantly increased after mir-93 induction (Figure 3E, Figures S12C and S13C). These results were confirmed and extended by demonstrating that mir-93 induction in primary tumors increased their tumor-initiating capacity when implanted into secondary recipients (Figure 3E, Figures S12C, S13C). Together, these experiments suggested that the effects of mir-93 on the CSC population differed in different molecular subtypes of breast cancer, an observation consistent with the hypothesis that miRNA effects might be differentiation state dependent.

### mir-93 downregulates stem cell regulatory genes in BCSCs

**Figure 4:**
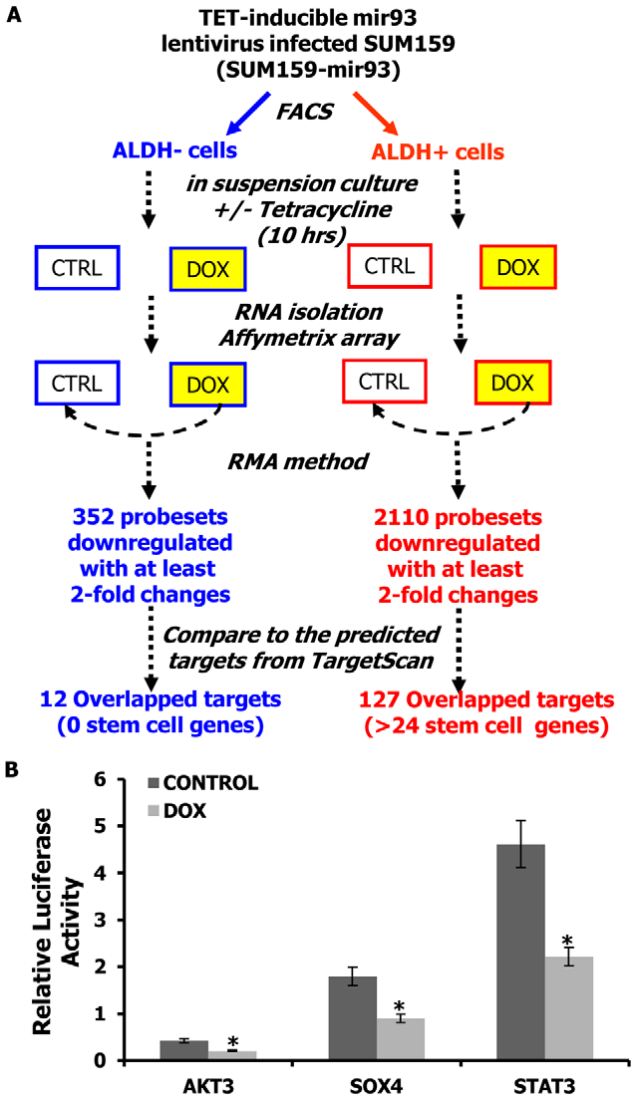
mir-93 targets stem cell regulatory genes. A. Schematic representation of the experimental design to identify the direct targets of mir-93 in SUM159 cells. B. Activity of the luciferase gene linked to the 3′UTR of AKT3, SOX4, or STAT3. The pMIR-REPORT firefly luciferase reporter plasmids with the wild-type 3′UTR sequences of AKT3, SOX4, or STAT3 were transiently transfected into pTRIPZ-mir-93-SUM159 cells and an internal control ACTB luciferase reporter was co-transfected for normalization. The cells were treated with or without DOX. Luciferase activities were measured after 48 hr. The relative luciferase activity was calculated as the ratio of (the results from the cells transfected by individual reporter)/(the results from the cells transfected by the internal control in the same cell group). The data are mean and standard deviation (SD) of separate transfections (n = 4). *p<0.05; Error bars represent mean ± STDEV.

In order to determine the cellular targets of mir-93 in BCSCs, ALDH^+^ and ALDH^−^ populations of SUM159 cells were separated and cultured in suspension in the presence or absence of DOX for ten hours. Gene expression profiles in the four populations were determined utilizing Affymetrix oligonucleotide microarrays (Figure 4A). Of the 2,000 genes downregulated at least two-fold upon DOX treatment in the ALDH^+^ population (Table S1), 127 overlapped with the predicted target sequences of mir-93 including twenty-four genes known to be involved in stem cell regulation (Figure 4A and Table S2) including JAK1, SOX4, STAT3, AKT, E2H1 and HMGAZ. The downregulation of these genes in pTRIPZ-SUM159-mir-93, pTRIPZ-HCC1954-mir-93 cell lines and pTRIPZ-MC1-mir-93 were confirmed with customized PCR array plates (Figures S15, S16, S17). In contrast, only 352 genes were significantly downregulated by DOX in the ALDH^−^ population (Table S3) with twelve of these genes (no stem cell genes) overlapping with the predicted mir-93 targets. These studies suggest that mir-93 regulates the CSC population by simultaneously targeting a number of stem cell regulatory genes. To confirm this, we utilized a luceriferase reporter assay to determine the effect of mir-93 on the expression of the stem cell regulatory genes AKT3, SOX4 and STAT3 selected from the expression profiling data. Expression of mir-93 reduced the level of these stem cell regulatory genes in SUM159 (Figure 4B) and HCC1954 cells (Figure S18) but not in luminal MCF7 and MDA-MB-453 cells (Figure S19). Furthermore, knockdown of STAT3 or SOX4 but not AKT3 decreases the proportion of ALDH^+^ SUM159 cells suggesting these genes play a role in the regulation of CSC self-renewal (Figure S20). The 127 genes in pTRIPZ-MCF7-mir-93 were also tested with customized PCR array plates, and interestingly, most of the stem cell genes were not knocked-down by mir-93 induction in the ALDH^+^ proportion of MCF7 (Figure S21).

### mir-93 regulates cell proliferation

**Figure 5:**
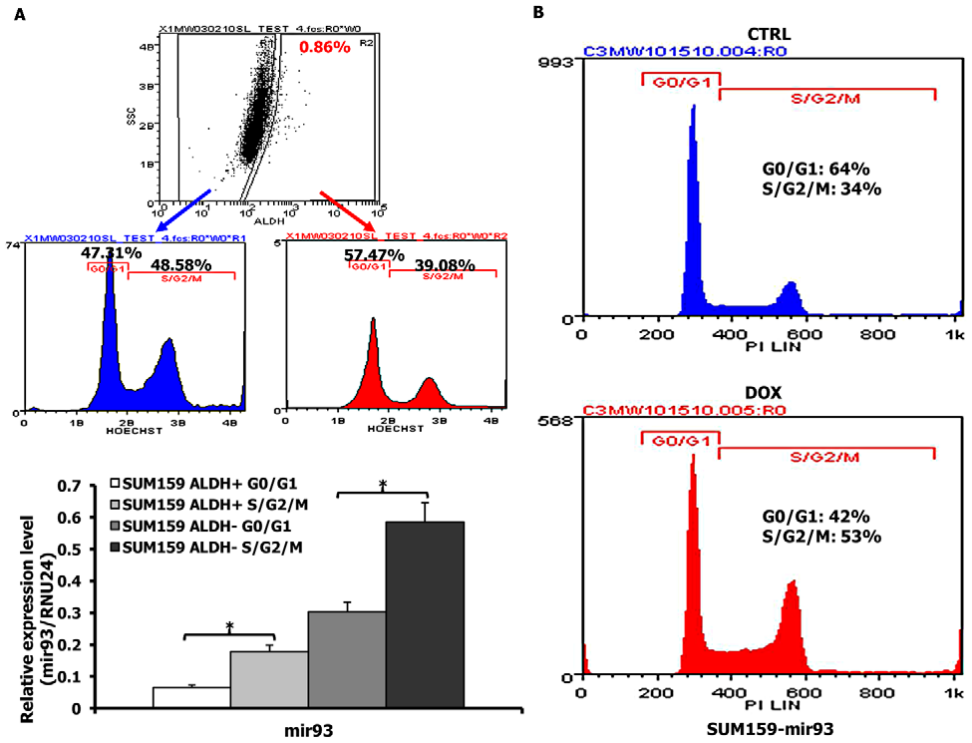
mir-93 regulates the cell cycle in SUM159 cells. A. SUM159 cells were stained with Aldefluor and Hoechst33342 and dead cells excluded by 7-AAD staining. Cells from G0/G1 and S/G2/M were sorted from ALDH^+^ or ALDH^−^ populations and mir-93 expression was measured with qRT-PCR. B. Cell cycle analysis of pTRIPZ-SUM159-mir-93 cells in the presence or absence of DOX for 7 days. Propidium iodide staining followed by flow cytometry was used to analyze cell cycle distribution. mir-93 induction with DOX resulted in a decreased proportion of cells in G0/G1 and an increased proportion of cells in S/G2/M. *p<0.05; Error bars represent mean ± STDEV.

To determine the relationship between mir-93 expression and cell cycle kinetics, we assessed mir-93 expression in quiescent and cycling ALDH^+^ and ALDH^−^ populations. Cycling (S/G2/M) cells expressed significantly higher levels of mir-93 compared to quiescent (G0/G1) cells in both the ALDH^−^ and ALDH^+^ compartments (Figure 5A). To determine whether mir-93 induces or is a consequence of cellular proliferation, we utilized the DOX inducible mir-93 construct to determine the effect of mir-93 induction on cell cycle distribution. Induction of mir-93 reduced the quiescent cell population from 64% to 42% suggesting that this miRNA has the capacity to directly regulate the cell cycle (Figure 5B). Furthermore, induction of mir-93 increased the proliferation of SUM159 by 29% (Figure S22). Although mir-93 induction had similar effects on the basal HCC1954 cell lines it had no significant effect on the cell cycle of the luminal MCF7 cells (Figure S22, Figure S23).

To determine the relationship between the stem cell phenotype and cell cycle kinetics, we determined the cell cycle distribution of ALDH^+^ and ALDH^−^ populations. The ALDH^+^ population in SUM159 cells had a higher fraction of non-cycling cells compared to ALDH^−^ cells (Figure 5A). This finding was confirmed by Ki67 and MCM7 staining (Figure S24).

### mir-93 promotes Mesenchymal-Epithelial Transition (MET) in SUM159 cells

**Figure 6:**
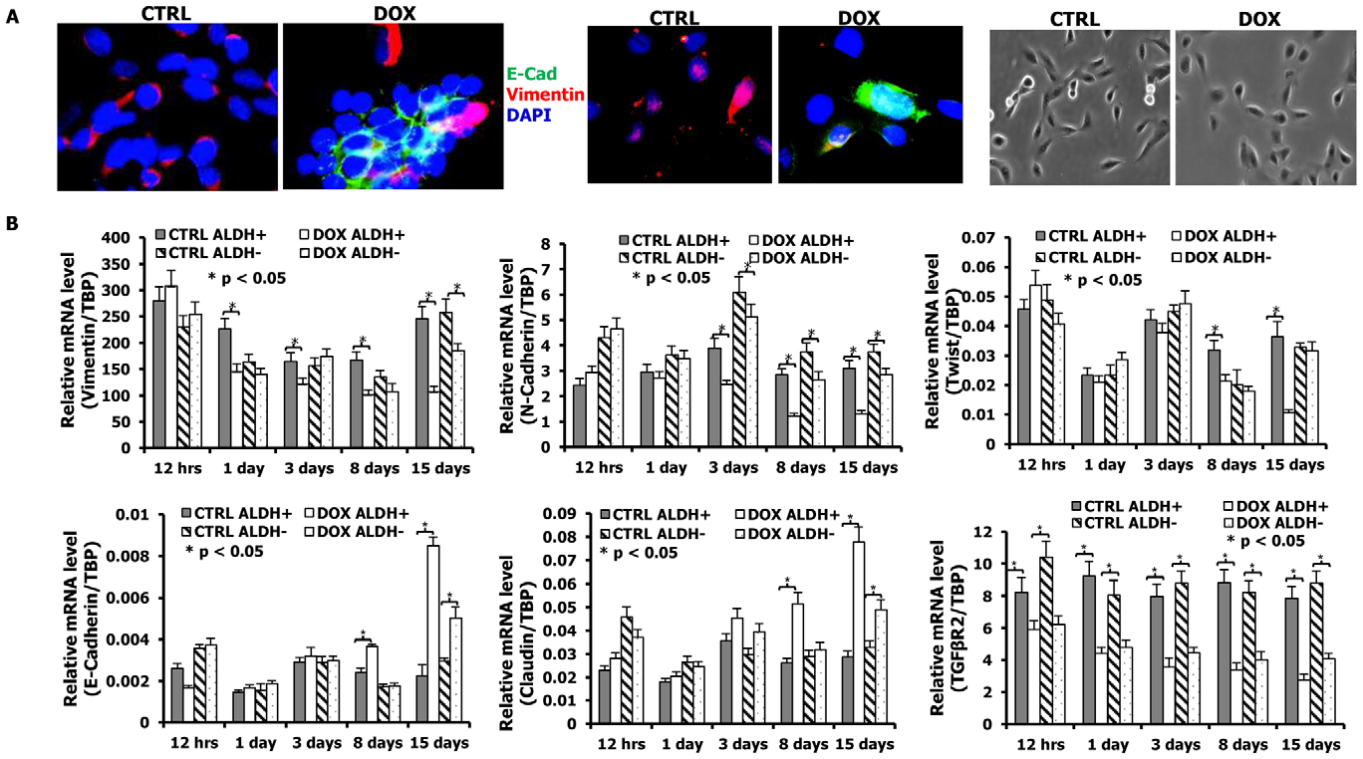
mir-93 initiates MET in SUM159 cells. A. pTRIPZ-SUM159-mir-93 cells were plated in 2-well chamber slides with (DOX) or without (CTRL) Doxycycline for 7 days. E-Cadherin and Vimentin were deleted by immunofluorescence staining. Expression of mir-93 in SUM159 cells causes them to assume a more epithelial appearance associated with a decrease in Vimentin and an increase in membrane localized E-Cadherin expression. The phase micrographs for CTRL and DOX are also shown. E-Cadherin, Green; Vimentin, Red; DAPI, Blue. A representative sample from 3 independent samples is shown. B. The effect of mir-93 expression on a panel of epithelial and mesenchymal markers at the mRNA level as accessed by qPCR. pTRIPZ-SUM159-mir-93 cells were plated with or without DOX, and ALDH^+^ and ALDH^−^ cells were sorted at different times (12 hours, 1 day, 3 days, 8 days, 15 days) by Aldefluor assay. qRT-PCR was utilized to access the effects of mir-93 on mRNA expression of mesenchymal markers (Vimentin, N-Cadherin and Twist), epithelial markers (E-Cadherin and Claudin), and TGFβR2. *p<0.05; Error bars represent mean ± STDEV.

SUM159 cells are derived from a “claudin^low^” subtype of breast cancer which is characterized as having a high proportion of cells displaying “epithelial-mesenchymal transition (EMT)”. This state is characterized by loss of epithelial characteristics such as apical basal polarity and E-Cadherin expression and acquisition of mesenchymal characteristics, including loss of cell polarity and expression of Vimentin. We determined the effects of mir-93 expression on MET of SUM159 cells by assessing markers of these states at the protein and mRNA levels. SUM159 cells have a mesenchymal morphology and express Vimentin, but not the epithelial marker E-Cadherin, an effect not dependent on cell density (Figure 6A). Expression of mir-93 in these cells caused them to assume a more epithelial appearance associated with a decrease in Vimentin and an increase in E-Cadherin expression (Figure 6A). Similar effects were seen in the basal HCC1954 cell line (Figure S25) although these were less pronounced. To confirm and extend these results we determined the effect of mir-93 expression on mRNA expression of a wider panel of epithelial and mesenchymal markers. We also determined the time course of expression of epithelial and mesenchymal marker mRNAs expressed in ALDH^+^ stem cells and ALDH^−^ non-stem cell populations. Expression of mir-93 in SUM159 cells resulted in a time dependent decrease in expression of mesenchymal markers, Vimentin, N-cadherin and Twist, and an increase in the epithelial markers E-Cadherin and Claudin (Figure 6B). Furthermore, although these effects were seen in ALDH^−^ populations, they were even more pronounced in the ALDH^+^ stem cell compartment. Since TGFβ is a major inducer of the EMT [26], [27], we examined the effect of mir-93 expression on components of this pathway. Interestingly, expression of mir-93 significantly reduced expression of the mRNA for TGFβR2 in both ALDH^+^ and ALDH^−^ SUM159 cells. This effect was seen as early as twelve hours, suggesting a potential role for down regulation of TGFβ signaling in inducing the MET.

### mir-93 maintains normal breast stem cells in an epithelial state

**Figure 7:**
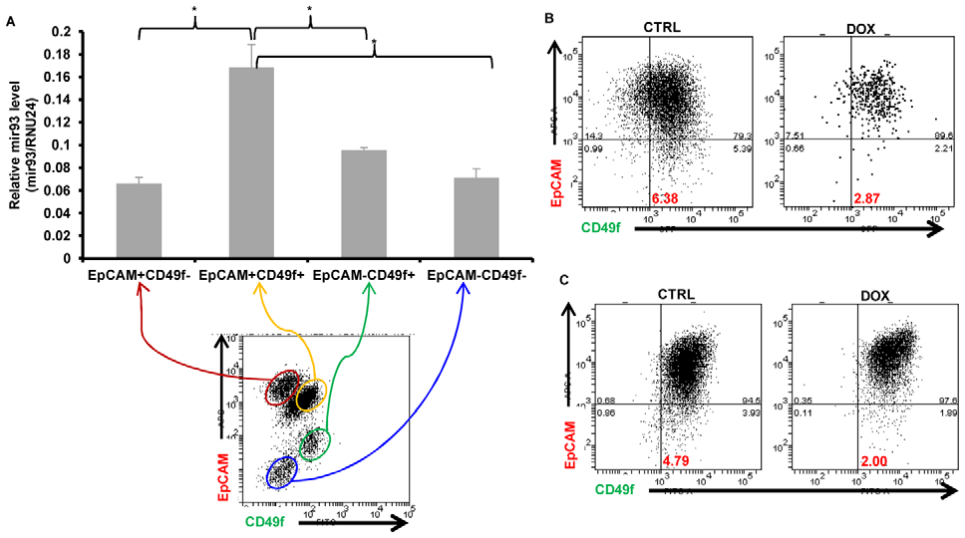
mir93 promotes MET in normal breast epithelial cells. A. Single cells were isolated from normal breast tissues and stained with EpCAM-APC and CD49f-FITC for FACS sorting. After sorting, total RNA were isolated from different sorted groups (EpCAM^+^CD49f^−^, EpCAM^+^CD49f^+^, EpCAM^−^CD49f^+^, EpCAM^−^CD49f^−^) and mir-93 expression were measured by qRT-PCR. p<0.05; Error bars represent mean ± STDEV. B. Single cells were isolated from normal breast tissues and infected with mir-93-expressing lentiviruses in suspension. After one week, mammospheres were dissociated into single cells and plated in adherent culture in the absence (CTRL) or presence (DOX) of DOX for two weeks. Then, cells were dissociated and stained with EpCAM-APC and CD49f-FITC for FACS analysis. C. MCF10A cells were cultured in the absence (CTRL) or presence (DOX) of DOX for two weeks. Cells were then dissociated and stained with EpCAM-APC and CD49f-FITC for FACS analysis.

In addition to breast cancer cells, we also determined the effects of mir-93 expression on normal breast cell differentiation. We utilized flow cytometry to access expression of EpCAM and CD49f in breast epithelial cells obtained from reduction mammoplasties. It has previously been shown that mammary stem cells are contained within the EpCAM^−^CD49f^+^ population while double positive (EpCAM^+^CD49f^+^) cells are luminal progenitors, EpCAM^+^CD49f^−^ more differentiated Luminal cells, while EpCAM^−^CD49f^−^ constitute stromal cells [28]. We compared mir-93 expression levels in these four populations. Interestingly, we found that the highest level of mir-93 is expressed in the EpCAM^+^CD49f^+^ population (Figure 7A), which suggested mir-93 was required to maintain the cells as EpCAM^+^CD49f^+^. Furthermore, overexpression of mir-93 in freshly isolated normal breast cells or in immortalized non-transformed MCF-10A cells increased the proportion of cells expressing EpCAM (Figure 7B, 7C). These studies suggested that mir-93 played a role in maintaining normal breast cells in an epithelial (EpCAM^+^) state.

## Discussion

In these studies, we demonstrate that mir-93 is capable of modulating breast CSC populations by regulating their proliferation and differentiation states. To examine this, we utilized breast cancer cell lines representing different states of differentiation. The levels of endogenous mir-93 expression paralleled cellular differentiation states with mir-93 levels lowest in the most primitive “claudin^low^” SUM159 cells, highest in the “luminal” MCF7 cells and intermediate in the “basal” HCC1915 cells. We utilized a DOX inducible system to determine the effects of enforced mir-93 expression on the CSC populations assessed by expression of the stem cell markers ALDH and CD24^−^CD44^+^ as well as by mouse xenograft assays [14], [22]. Enforced mir-93 expression in claudin^low^ and basal breast cancer cell lines significantly reduced the CSC populations as assessed by the Aldefluor assay. To assess the functional relevance of this, we determined the effect of mir-93 induction in SUM159 and HCC1954 cells on tumor growth in NOD/SCID mouse xenografts. The effects of mir-93 expression on tumor initiating capacity was confirmed using two primary breast xenografts generated without *in vitro* culture. mir-93 expression decreased the CSC in these claudin^low^ primary xenografts. In contrast, overexpresson of mir-93 in the luminal MCF7 cells line resulted in an increase in CD24^−^CD44^+^ CSC resulting in increased tumor growth. This demonstrates that the effect of mir-93 on CSC populations is dependent on the cellular differentiation state. This model allowed us to simulate potential clinical scenarios involving CSC targeting agents. To simulate the effects of CSC targeting agents in advanced disease, tumors were inoculated into mammary fatpads and when the tumors were palpable mir-93 was induced by addition of doxycycline to the mouse drinking water. In this setting, mir-93 induction had only a modest effect in reducing tumor growth. Addition of the chemotherapeutic agent docetaxel resulted in a more significant reduction in tumor size, an effect that was accentuated by mir-93 induction. CSC models predict that the efficacy of CSC targeting agents should be most pronounced in the adjuvant setting where tumor growth from micrometastasis is dependent on stem cell self-renewal [29]. Consistent with this model, induction of mir-93 immediately after fatpad implantation or after development of micrometastasis by intracardiac injection completely blocked tumor recurrence. Furthermore, when treatment was discontinued at eight weeks, animals that received chemotherapy alone developed local tumor growth and metastasis while those with mir-93 induction with or without chemotherapy showed no recurrence when animals were sacrificed after four months. These studies provide strong support for the CSC hypothesis and provide a valuable animal model for clinical trial design using CSC targeting agents.

To determine the molecular mechanisms of mir-93 CSC regulation, we employed an unbiased approach assessing the effect of mir-93 expression on early changes in global gene expression profile coupled with prediction of miRNA target sequences. Interestingly, this analysis revealed that twenty-four genes known to be involved in stem cell self-renewal including JAK1, SOX4, STAT3, AKT, EZH1, HMGA2 are targeted by mir-93. In addition, this miRNA targets two important regulators of TGFβ signaling, TGFβR2 and SMAD5.

mir-93 expression was also associated with and in turn regulates cellular proliferation. Quiescent G0/G1 cells expressed lower levels of mir-93 than proliferating cells in S/G2/M phase. Furthermore, enforced expression of mir-93 increased the fraction of cycling cells.

We demonstrate that induction of mir-93 in mesenchymal-like SUM159 cells induces an MET in the ALDH^+^ CSC population characterized by increased expression of E-Cadherin and Claudin, with concomitant downreguation of mesenchymal genes, such as Vimentin, N-Cadherin and Twist. mir-93 also inhibits TGFβ signaling by targeting TGFβR2, an effect seen within twelve hours of mir-93 induction. This was followed by an MET in the Aldefluor-positive CSC population. Since TGFβ is a major regulator of EMT, abrogation of this signaling pathway may facilitate MET. Of interest, it has been recently reported that the mir-106b-25 cluster including mir-93 is induced in the early stages of nuclear reprogramming of fibroblasts into IPS cells [30]. This is accompanied by a mesenchymal to epithelial conversion in these cells which is obligatory for reprogramming to recur. This suggests that this miRNA cluster may regulate MET in multiple biological contexts.

**Figure 8:**
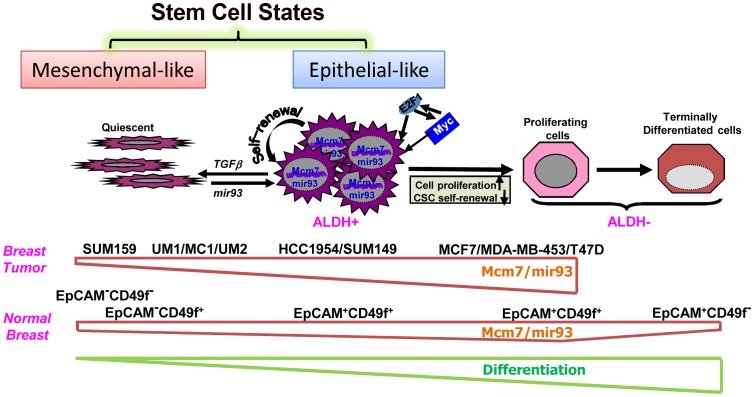
A hypothetic model illustrating regulation of normal and malignant mammary stem cell states and fates by mir-93.

In summary, our experiments suggest that CSCs can exist in two alternative epithelial and mesenchymal states, the balance of which is regulated by miRNAs including mir-93 (Figure 8). The mesenchymal state associated with an invasive phenotype characterized by quiescence and low mir-93 expression is maintained by growth factors such as TGFβ. Upon activation of cellular proliferation, MYC and E2F are induced leading to expression of MCM7, a licensing factor required for DNA synthesis. Concomitantly, mir-93 and its related miRNA cluster is co-synthesized which promotes further proliferation while simultaneously downregulating TGFβ signaling. This facilitates a mesenchymal to epithelial transition in the CSC population characterized by decreased invasiveness and increased proliferation. Continued expression of mir-93 simultaneously downregulates a number of stem cell self-renewal pathways including JAK/STAT, AKT, EZH1 and HMGH2, promoting cellular differentiation and depleting the CSC population. The model depicted in Figure 8 is consistent with our observation that mir-93 level is highest in the EpCAM^+^CD49f^+^ normal mammary cells and decreased with terminal differentiation. In contrast, the effects of mir-93 depend on the cellular differentiation state accounting for differences we observed in claudin^low^, basal and luminal breast cancers, with mir-93 level highest in the luminal MCF7 cell line compared to basal HCC1954 and claudin^low^ SUM159 cell lines. MCF7 cells are highly proliferative although unlike normal mammary cells incapable of terminal differentiation (Figure 8). The existence of alternative CSC states, associated with expression of different protein markers has important implications for understanding the plasticity of CSCs. For example, it has been claimed that CSCs may be generated from non-CSC tumor populations through induction of EMT [31]. However, the existence of alternative CSC state suggests that the acquisition of stem cell markers may reflect transition of CSC states rather than generation of CSCs from non-CSC populations. In addition, the existence of multiple stem cell states suggests the necessity of developing of therapeutic strategies capable of effectively targeting CSCs in all of these states.

## Materials and Methods

### Cell culture

Breast cancer cell line SUM159 and SUM149 have been extensively characterized (http://www.asterand.com) [32]. HCC1954, MCF-7, MDA-MB-453 and MCF10A were purchased from ATCC. The cell lines were grown using the recommended culture conditions. Briefly, the culture medium for SUM159 and SUM149 is Ham's F-12 (Invitrogen) supplemented with 5% FBS, 5 ug/mL insulin, and 1 ug/mL hydrocortisone (both from Sigma, St. Louis, MO). MCF7, MDA-MB-453 and HCC1954 cells were maintained in RPMI1640 medium (Invitrogen, Carlsbad, CA) supplemented with 10% fetal bovine serum (ThermoFisher Scientific, Pittsburgh, PA), 1% antibiotic-antimycotic (Invitrogen, Carlsbad, CA), and 5 µg/ml insulin (Sigma-Aldrich, St Louis, MO).

### Dissociation of mammary tissue

100–200 g of normal breast tissue from reduction mammoplasties was minced with scalpels, dissociated enzymatically, and single cells were isolated as described previously [33], [34]. The single cells were utilized for FACS sorting or were cultured in suspension as described previously [33], [34]. Mammospheres were dissociated into single cells enzymatically and mechanically, and then cultured in regular cell culture plates [33], [34].

### Constructs and virus infection

For construction of the mir-93 sensor, miRNA-complementary oligonucleotides were annealed and cloned into a Marx vector that directs GFP expression. The mir-93 miRNA target sequence was engineered into the 3′ untranslated region (UTR) of the cDNA encoding for the GFP fluorescent protein. Expression of this construct in cells that express the mir-93 miRNA results in a RNAi pathway-dependent degradation of GFP mRNA and thus no green fluorescence. In contrast, in cells with repressed mir-93 miRNA, the GFP mRNA is not degraded and resulting in expression of the fluorescent GFP. shRNA oligos for STAT3, AKT3 or SOX4 were inserted to PlentiLox3.7-DsRed lentiviral vector. A highly efficient lentiviral expression system (TRIPZ lentivral vector;www.openbiosystems.com/RNAi) was used to generate mir-93-expressing lentiviruses; and mirZIP-lentivector (SBI, Mountain View, CA) was used to generate mir-93-knockdown lentiviruses in UM Vector Core Facility. The cell lines were infected with the lentiviruses as described previously [34].

### Aldefluor assay and flow cytometry

The Aldefluor kit (StemCell Technologies, Inc, Vancouver, BC, Canada) was used to isolate cells with high ALDH enzymatic activity as illustrated in the manufacturer's instructions. Briefly, single cells were suspended in buffer containing ALDH substrate – BAAA (1 µmol/l per 1×10^6^ cells) and incubated at 37°C for 40 minutes. In each experiment, the specific ALDH inhibitor diethylaminobenzaldehyde (DEAB) was used as negative control at 50 mmol/L. A FACStarPLUS (Becton Dickinson) was used for FACS. Aldefluor fluorescence was excited at 488 nm and fluorescence emission was detected using a standard fluorescein isothiocyanate (FITC) 530/30 band pass filter. The sorting gates were established based on negative controls. CD44/CD24 staining was performed as previously described [14]. Briefly, cells were stained with primary antibodies anti-CD44 labeled APC (dilution 1∶10, BD Pharmingen), and anti-CD24 labeled FITC (dilution 1∶10, BD Pharmingen). In all *in vivo* experiments, mouse cells were eliminated by excluding H2Kd^+^ (mouse histocompatibility class I, BD Pharmagen) cells during flow cytometry. 0.5 µg/ml 4′,6-diamidino-2-phenylindole (DAPI) (Sigma) was used to access cell viability.

### Cell cycle analysis

Cells (1×10^6^) were harvested and washed in cold PBS followed by fixation in 70% alcohol for thirty minutes on ice. After washing in cold PBS three times, cells were resuspended in 0.8 mL of PBS solution with 40 µg of propidium iodide and 0.1 µg of RNase A for thirty minutes at 37°C. Samples were analyzed for DNA content using aFACSCalibur cytometer (Becton Dickinson, San Jose, CA).

### MTT assay

The effect of mir-93 on cell proliferation was measured using an MTT assay. Briefly, 200–500 cells from Control and DOX-treated groups were seeded in 96-well culture plates and were cultured in the absence (CTRL) and presence (DOX) of DOX for 7 days. Subsequently, 0.025 ml of MTT solution (5 mg/ml) was added to each well, and the cells were incubated for 2 h. After centrifugation, the supernatant was removed from each well. The colored formazan crystal produced from MTT was dissolved in 0.15 ml of isopropanol with 4 mM HCl and 0.1% NP40, and the optical density (OD) value was measured at 590 nm.

### RNA extraction

Total RNA was isolated using RNeasy Micro Kit (Qiagen, Valencia, CA), and total RNA with enriched miRNA was isolated using miRNeasy mini Kit, according to the manufacturer's instructions.

### Gene expression profiling with DNA microarrays

Gene expression analyses used Affymetrix U133 Plus 2.0 human oligonucleotide microarrays containing over 47,000 transcripts and variants including 38,500 well-characterized human genes. Preparation of cRNA, hybridizations, washes and detection were done as recommended by the supplier (http://www.affymetrix.com/index.affx). Expression data were analyzed by the RobustMultichip Average method in R using Bioconductor and associated packages [35].

### Real-time quantitative PCR (qRT–PCR)

MiRNA expression level was measured utilizing TaqMan qRT-PCR (Applied Biosystems, Carlsbad, CA). Single-stranded cDNA was synthesized from 10 ng of miRNA enriched total RNA using specific miRNA primers (TaqMan MiRNA Assay, PN 4427975, Applied Biosystems) and the TaqMan MiRNA Reverse Transcription Kit (PN 4366596, Applied Biosystems). Two ul of cDNA was used as a template in a 20 ul PCR reaction. PCR products were amplified using specific primers (TaqMan MiRNA Assay) and the Taq-Man Universal PCR Master Mix (PN 4324018, Applied Biosystems), and PCR was performed in a ABI PRISM 7900HT sequence detection system with 384-Well block module and automation accessory (Applied Biosystems) by incubation at 50°C for two min and then 95°C for ten min followed by forty amplification cycles (fifteen seconds of denaturation at 95°C and one min of hybridization and elongation at 60°C). PCR reactions for each sample were run in triplicate. The number of cycles required for amplification to reach the threshold limit, the Ct-value was used for quantification. RNU24 was used as an endogenous control for miRNA data normalization, and TBP was used as an endogenous control for other gene normalization. All TaqMan miRNA assays used in this study were obtained from Applied Biosystems.

### Tumorigenicity in NOD/SCID mice

All mice were housed in the AAALAC-accredited specific pathogen-free rodent facilities at the University of Michigan. Mice were housed on sterilized, ventilated racks and supplied with commercial chow and sterile water both previously autoclaved. All experimentation involving live mice were conducted in accordance with standard operating procedures approved by the University Committee on the Use and Care of Animals at the University of Michigan. Six-week old female NOD/SCID mice were purchased from Jackson Laboratories (Bar Harbor, ME) and housed in SPF microisolator cages in the animal facility of University of Michigan. Tumorigenicity of 10,000 (Adjuvant setting) cells or 100,000 (Advanced setting) cells in the mamary fatpads of NOD/SCID mice was accessed. Six mice were included in each cohort. The animals were euthanized when the tumors were 1.0–1.5 cm in diameter, in compliance with regulations for use of vertebrate animal in research. A portion of each fat pad was fixed in formalin and embedded in paraffin for histological analysis. Another portion was analyzed by the ALDH or CD24/CD44 cytometric staining.

### Primary xenografts

Human breast tumors were obtained as biopsy cores or pieces of tumors after surgery and implanted in humanized cleared fat pads of NOD/SCID mice for establishing xenotransplants. The success of xenotransplantation was approximately 20%, similar to previous reports in the literature. Three xenotransplants were used: an ER^−^PR^−^ERBB2^−^ tumor at the 20th passage in animals (MC1), an ER^−^PR^−^ERBB2^−^ tumor at the 5th passage (UM1), and an ER^+^PR^+^ERBB2^−^ tumor at the 8th passage (UM2).

### Intra-cardiac injection

All procedures were approved by the University Committee for the Use and Care of Animals (UCUCA) of the University of Michigan. The intracardiac injection was carried out according to previously published methods [36]. Briefly, six-week-old NOD/SCID mice were anesthetized with isoflurane gas (a 2% isofluorane/air mixture) and injected in the left ventricle of the heart with 100,000 cells in 100 µl of sterile Dulbecco's PBS lacking Ca^2+^ and Mg^2+^. For each of the cell lines (SUM159-luc, HCC1954-luc), five animals were injected.

### Bioluminescence imaging

Baseline bioluminescence was assessed before inoculation and each week thereafter. Mice were anesthetized with isoflurane gas and given a single i.p. dose of 150 mg/kg D-luciferin (Promega) in PBS. For photon flux counting, we used a charge-coupled device camera system (Xenogen) with a nose-cone isofluorane delivery system and heated stage for maintaining body temperature. Results were analyzed after six min of exposure using Living Image software provided with the Xenogen IVIS imaging system.

### Immunostaining

For ALDH1 staining, paraffin-embedded sections of breast tumors from xenografts were deparaffinized in xylene and rehydrated in graded alcohol. Antigen enhancement was done by incubating the sections in citrate buffer pH 6.0 (Dakocytomation, Copenhagen, Denmark) as recommended. Slides were stained using Peroxidase histostain-Plus Kit (Zymed) according to the manufacturer's protocol. ALDH1 antibody (BD biosciences) was used at a 1∶50 dilution. AEC (Zymed) was used as substrate for peroxidase. Slides were counter-stained with hematoxylin and coverslipped using glycerin. For E-Cadehrin, Vimentin, MCM7, Ki67 and DAPI fluorescent staining, cells were fixed in ice-cold methanol and permeablized with 0.15% triton X-100. E-Cadherin antibody (Santa Cruz, 1∶100 dilution), Vimentin antibody (Santa Cruz, 1∶200 dilution), MCM7 antibody (Cell signaling, 1∶100 dilution), p21 antibody (Cell Signaling, 1∶400 dilution) and Ki67 antibody (Dako, 1∶150 dilution) were used and incubated for 1 hour at room temperature. PE and FITC labeled secondary antibodies (Jackson Labs) were used at the dilution 1∶200 and incubated for twenty min. Nuclei were counterstained with DAPI/antifade (INVITROGEN) and cover slipped. Sections were examined with a Leica fluorescent microscope.

### 3′ UTR luciferase reporter assay

The pMIR-REPORT luciferase reporter plasimds with the 3′ UTR sequence of AKT3, SOX4, STAT3 or the control ACTB were transfected into the cell lines using Fugene HD tansfection reagent (Roche Applied Science) according to the manufacturer's instruction. After transfection, cells were dissociated and cultured with or without DOX. Luciferase activity was assayed by luciferase assay kit (Promega). Luciferase activities were measured after forty-eight hrs utilizing a luminometer. The results were presented as the luciferase activity of cells transfected with 3′ UTR sequence of AKT3, SOX4, or STAT3 normalized to cells transfected with the luciferase activity of cells transfected with 3′ UTR sequence of ACTB.

### Statistical analysis

Results are presented as the mean ± standard deviation (STDEV) for at least three repeated individual experiments for each group using Microsoft Excel. Statistical differences were determined by using ANOVA and student's t-test for independent samples. A p-value of less than 0.05 was considered statistically significant.

## Supporting Information

Figure S1 mir93 inhibits tumor growth and metastasis by decreasing CSCs in HCC1954 cells. A. ALDH-positive cells from HCC1954 cells shows lower mir93 expression level in comparison to ALDH-negative cells by qRT-PCR. B. 1×10^6^ pTRIPZ-HCC1954-mir93 cells were plated in T75 flasks and, after overnight, the cells were treated with Vehicle control, DOX (1 ug/ml), docetaxel (10 nM) or the combination for 3–7days. Cells were utilized for Aldefluor assay and stained for Annexin V-APC and DAPI for apoptosis assay. C. 100k pTRIPZ-HCC1954-mir93 cells were injected into the 4^th^ fatpads of NOD/SCID mice. The treatment started as indicated by the red arrow. DOX alone (1 mg/ml in drinking water), or docetaxel (10 mg/kg i.p. once weekly) alone, or the combination inhibits SUM159 tumor growth in vivo. D. Tumors from each group were collected. ALDH was accessed by the Aldefluor assay on viable dissociated cells and by ALDH1 immunohistochemistry on fixed sections. E. Serial dilutions of cells obtained from these xenografts were implanted in the 4^th^ fatpads of secondary mice, which received no further treatment. F. 10k pTRIPZ-HCC1954-mir93 cells were injected into the 4^th^ fatpads of NOD/SCID mice. The treatment started immediately after injection as indicated by the red arrow and stopped as indicated by the green arrow. G. 200k pTRIPZ-HCC1954-mir93-luc cells in 100 ul of PBS were injected into the left ventricle of NOD/SCID mice. The treatment started immediately after injection as indicated by the red arrow and stopped as indicated by the green arrow. Metastasis formation was monitored using bioluminescence imaging. Quantification of the normalized photon flux, measured at weekly intervals following inoculation. H. Histologic confirmation, by H&E staining, of metastasis in soft tissues resulting from mice with different treatments in G. *p<0.05; Error bars represent mean ± STDEV. The colored “*” on the side of the tumor growth or metastasis curve represents the tumor growth is significantly different between Control group and the group with the same colored curve.
(PDF)

Figure S2 mir-93 is induced in primary tumors with DOX. 100k pTRIPZ-SUM159-mir-93 cells were injected into the 4^th^ fatpads of NOD/SCID mice. Different treatments were initiated (Vehicle Control (Control), DOX alone (DOX), docetaxel alone, or the combination). At the end of treatment, cells from Control and DOX groups were isolated from the tumors and mir-93 expression level was measured by qRT-PCR.
(PDF)

Figure S3 Induction of mir93 decreases both ALDH^+^ and CD24^−^CD44^+^ cells in the primary breast tumor xenografts. Cells isolated from primary human breast xenografts UM2 (A), MC1 (B) and UM1 (C) were sorted for ALDH^+^ and ALDH^−^ or CD24^−^CD44^+^ and the rest (CD24^−^CD44^−^, CD24^+^CD44^+^, CD24^+^CD44^−^). RNA was isolated from each group of sorted cells and the expression level of mir-93 or RNU24 level was measured by qRT-PCR. *p<0.05; Error bars represent mean ± STDEV.
(PDF)

Figure S4 mir-93 expression in Sensor-GFP-positive and Sensor-GFP-negative SUM159 cells. Mir-93-sensor-GFP SUM159 cells were sorted for GFP-positive and GFP-negative cells by flow cytometry and RNA was isolated from both group of sorted cells and the expression level of mir-93 or RNU24 level was measured by qRT-PCR. *p<0.05; Error bars represent mean ± STDEV.
(PDF)

Figure S5 ALDH1A1 protein level in Sensor-GFP-positive and Sensor-GFP-negative SUM159 cells. mir-93-sensor-GFP SUM159 cells were sorted for GFP-positive and GFP-negative cells by flow cytometry. A portion of cells were utilized for western blot, and some cells were cytospun down and stained with ALDH1A1 by immunohistochemical staining. *p<0.05; Error bars represent mean ±STDEV.
(PDF)

Figure S6 mir-93 induction reduces GFP in mir93-sensor-GFP-SUM159 cells. mir93-sensor-GFP-SUM159 cells were infected with pTRIPZ-mir93 lentivirus and grow in T75 flasks. DOX were added to the culture medium in the DOX group. Images were taken with fluorescence microscope.
(PDF)

Figure S7 1×10^6^ pTRIPZ-SUM149-mir93 cells were plated in T75 flasks and, after overnight, the cells were treated with Vehicle control or DOX (1 ug/ml) for 3–7 days. Induction of mir93 expression by DOX decreased the ALDH-positive population. *p<0.05; Error bars represent mean ± STDEV.
(PDF)

Figure S8 Modulation of mir93 level in SUM159 cells altered cell invasion in vitro and knockdown increases tumor growth in vivo. SUM159 cells were infected with no virus (non-infection), DsRed control lentivirus (SUM159-Neg-DsRed) or mirZip antisense mir93 lentivirus (SUM159-mirZip93-DsRed). A. microRNA RT-PCR demonstrated SUM159-mirZip93-DsRed reduced mir93 expression by more than 90%. B. SUM159-non-infection, SUM159-Neg-DsRed, SUM159-mirZip93-DsRed cells were grown in T75 flasks and Aldefluor assay was utilized to measure the percentage of ALDH^+^ cells. C. Serial dilutions of cells were implanted in the 4^th^ fatpads of NOD/SCID mice. SUM159-mirZip93-DsRed cells initiated tumors sooner and accelerated growth compared to equivalent number of control cells. D. Cells were isolated from the tumors in C and Aldefluor assay was utilized to measure the percentage of ALDH^+^ cells. E. Invasive capacity was assessed by a Matrigel invasion assay using serum as attractant. pTRIPZ-SUM159-mir93 cells are less invasive than the control cells in vitro accesses at 27 hours. F. The invasion was assessed by a Matrigel invasion assay using serum as attractant. SUM159-mirZip93-DsRed cells are more invasive than the control cells in vitro accesses at 27 hours. *p<0.05; Error bars represent mean ± STDEV. The colored “*” on the side of the tumor growth curve represents the tumor growth is significantly different between Control group and the group with the same colored curve.
(PDF)

Figure S9 mir-93 inhibits tumor metastasis in SUM159 cells. 200k pTRIPZ-SUM159-mir-93-Luc cells in 100 ul of PBS were injected into the left ventricle of NOD/SCID mice. Different treatments were initiated (Vehicle Control (Control), DOX alone (DOX), docetaxel alone, or the combination). At the end of treatment, H&E staining and Pan-cytokeratin (AE1/AE3) staining (in Brown) were performed to confirm the metastasis in bone and soft tissues resulting from mice with different treatments.
(PDF)

Figure S10 mir93 inhibits tumor growth in primary human breast xenografts MC1, UM2, and UM1. Cells isolated from primary xenografts MC1 (A) or UM2 (B) or UM1 (C) were transduced with the pTRIPZ-mir93 lentivirus in suspension. 10k pTRIPZ-MC1-mir93 or pTRIPZ-UM2-mir93 cells were injected into the 4^th^ fatpads of NOD/SCID mice. The treatment started right after injection as indicated by the red arrow. DOX alone, docetaxel alone or the combination prevented tumor growth. *p<0.05; Error bars represent mean ± STDEV. The colored “*” on the side of the tumor growth curve represents the tumor growth is significantly different between Control group and the group with the same colored curve.
(PDF)

Figure S11 Endogenous mir93 expression levels parallel cell differentiation state. ALDH^+^ population and ALDH^−^ population were separated from SUM159 cells and HCC1954 cells. CD24^−^CD44^+^ population and the remaining cell populations were separated from MCF7 cells. mir93 level was analyzed by microRNA qRT-PCR. Among these three cell lines, mir93 expression level is highest in MCF7 cells and lowest in the SM159 cells. In both SUM159 cells and HCC1954 cells, ALDH^+^ cells have lower mir93 expression compared to ALDH^−^ cells. In contrast, CD24^−^CD44^+^ in MCF7 cells showed no difference for mir93 expression level in comparison to the bulk population. *p<0.05; Error bars represent mean ± STDEV.
(PDF)

Figure S12 mir93 promotes tumor growth by increasing CSCs in MDA-MB-453 cells. A. 200k pTRIPZ-MDA-MB-453-mir93 cells were injected into the 4^th^ fatpads of NOD/SCID mice. Treatment was initiated as indicated by the red arrow. DOX (1 mg/ml in drinking water) promoted MDA-MB-453 tumor growth in vivo. B. Tumors from each group were collected. Aldefluor assay was performed on dissociated cells. DOX increased the ALDH^+^ populations in MDA-MB-453. C. Serial dilutions of cells obtained from these xenografts were implanted in the 4^th^ fatpads of secondary mice, which received no further treatment. Cells from DOX-treated tumors formed secondary tumors at all dilutions (1k, 10k, 32k), whereas only higher numbers of cells (32k) obtained from control xenografts were able to generate tumors. *p<0.05; Error bars represent mean ± STDEV.
(PDF)

Figure S13 mir93 promotes tumor growth by increasing CSCs in T47D cells. A. 500k pTRIPZ-T47D-mir93 cells were injected into the 4^th^ fatpads of NOD/SCID mice. Treatment was initiated as indicated by the red arrow. DOX (1 mg/ml in drinking water) promoted T47D tumor growth in vivo. B. Tumors from each group were collected. Aldefluor assay was performed on dissociated cells. DOX increased the ALDH^+^ populations in T47D. C. Serial dilutions of cells obtained from these xenografts were implanted in the 4^th^ fatpads of secondary mice, which received no further treatment. Cells from DOX-treated tumors formed secondary tumors at all dilutions (5k, 50k, 500k). *p<0.05; Error bars represent mean ± STDEV.
(PDF)

Figure S14 mir-93 is induced in primary tumors with DOX. 1000k pTRIPZ-MCF7-mir-93 cells were injected into the 4^th^ fatpads of NOD/SCID mice. Different treatments were initiated (Vehicle Control (Control), DOX alone (DOX), docetaxel alone, or the combination). At the end of treatment, cells from Control and DOX groups were isolated from the tumors and mir-93 expression level was measured by qRT-PCR.
(PDF)

Figure S15 Validation of the 127 overlapped gene expression with customerized StellArray PCR array plate in pTRIPZ-SUM159-mir93.
(PDF)

Figure S16 Validation of the 127 overlapped gene expression with customerized StellArray PCR array plate in pTRIPZ-HCC1954-mir93.
(PDF)

Figure S17 Validation of the 127 overlapped gene expression with customerized StellArray PCR array plate in pTRIPZ-MC1-mir93.
(PDF)

Figure S18 Luciferase assay confirming mir93 targets. The 3′UTR of AKT3, SOX4, and STAT3 pMIR-REPORT firefly luciferase reporter plasmids with the wild-type 3′UTR sequences of AKT3, SOX4, or STAT3 were transiently transfected into pTRIPZ-HCC1954-mir93 cells and an internal control ACTB luciferase reporter was co-transfected for normalization. The cells were treated with or without DOX. Luciferase activities were measured after 48 hr. The relative luciferase activity is shown as the ratio of (the results from the cells transfected by individual reporter)/(the results from the cells transfected by the internal control in the same cell group). *p<0.05; Error bars represent mean ± STDEV.
(PDF)

Figure S19 Luciferase assay testing mir93 targets. The 3′UTR of AKT3, SOX4, and STAT3 pMIR-REPORT firefly luciferase reporter plasmids with the wild-type 3′UTR sequences of AKT3, SOX4, or STAT3 were transiently transfected into pTRIPZ-MCF7-mir93 (A) or pTRIPZ-MDA-MB-453-mir93 (B) cells and an internal control ACTB luciferase reporter was co-transfected for normalization. The cells were treated with or without DOX. Luciferase activities were measured after 48 hr. The relative luciferase activity is shown as the ratio of (the results from the cells transfected by individual reporter)/(the results from the cells transfected by the internal control in the same cell group). Error bars represent mean ± STDEV.
(PDF)

Figure S20 Knockdown of STAT3 (A), AKT3 (B) or SOX4 (C) decreases ALDH^+^ cells in SUM159 cells. SUM159 cells were transfected with PlentiLox3.7-shRNA-DsRed viruses and accessed for the ALDH^+^ population by Aldeflour assay. *p<0.05; Error bars represent mean ± STDEV.
(PDF)

Figure S21 Validation of the 127 overlapped gene expression with customerized StellArray PCR array plate in pTRIPZ-MCF7-mir93.
(PDF)

Figure S22 The effects of mir93 on cell proliferation. Cell proliferation was measured with the MTT assay. 200–500 cells from Control and DOX-treated groups were seeded in 96-well culture plates and were cultured in the absence (CTRL) and presence (DOX) of DOX for 7days.Data represents means SEM, *n* = 5. *p<0.05; Error bars represent mean ± STDEV.
(PDF)

Figure S23 Analysis of cell cycle for pTRIPZ-HCC1954-mir93 cells and pTRIPZ-MCF7-mir93cells. Cell cycle analysis of pTRIPZ-HCC1954-mir93 cells and pTRIPZ-MCF7-mir93 cells in the presence or absence of DOX. Propidium iodide staining followed by flow cytometry was used to analyze cell cycle distribution. Mir93 induced by DOX treatment resulted in a decreased proportion of cells in the G0/G1 phase and an increased proportion of cells in the S/G2/M phase for pTRIPZ-HCC1954-mir93 cells. In contrast, DOX treatment has no effects on the cell cycle for pTRIPZ-MCF7-mir93 cells.
(PDF)

Figure S24 MCM7 and Ki67 expression is increased in ALDH- compared to ALDH+ SUM159 cells. ALDH + and − cells were separated by Aldefluor assay and expression of Ki67 and MCM7 accessed by immunofluorescence. Ki67, Red; MCM7, Green; DAPI, Blue. One representative sample from 3 independent samples is shown.
(PDF)

Figure S25 mir93 expression induces MET in HCC1954 cells. pTRIPZ-HCC1954-mir93 cells were plated in 2-well chamber slides with (DOX) or without (CTRL) Doxycycline for 7 days. E-Cadherin and Vimentin were stained with immunofluorescence staining. E-Cadherin, Green; Vimentin, Red; DAPI, Blue. One representative sample from 3 independent samples is shown.
(PDF)

Table S1 Downregualted probe set in ALDH^+^ population from DOX vs. ALDH^+^ population from CTRL.
(PDF)

Table S2 mir93 direct targets in SUM159 cells. Overlap between mir93 predicted targets from TargetScan 5.1 and profiling data from DOX-treated cells (DOX) to non-DOX-treated cells (CTRL) in the ALDH^−^ population (12 genes) or in the ALDH^+^ population (352 genes). Known stem cell regulatory genes highlighted in red. Genes underlined and bolded were analyzed utilizing the luciferase reporter assay.
(PDF)

Table S3 Downregualted probe set in ALDH^−^ population from DOX vs. ALDH^−^ population from CTRL.
(PDF)
