# Supplementary material for: MicroRNA93 Regulates Proliferation and Differentiation of Normal and Malignant Breast Stem Cells
Source: PLoS Genet. 2012 Jun 7;8(6):e1002751. doi: 10.1371/journal.pgen.1002751 (PMC3369932; doi:10.1371/journal.pgen.1002751)
Supplement: Figure S1 — mir93 inhibits tumor growth and metastasis by decreasing CSCs in HCC1954 cells. A. ALDH-positive cells from HCC1954 cells shows lower mir93 expression level in comparison to ALDH-negative cells by qRT-PCR. B. 1×10^6^ pTRIPZ-HCC1954-mir93 cells were plated in T75 flasks and, after overnight, the cells were treated with Vehicle control, DOX (1 ug/ml), docetaxel (10 nM) or the combination for 3–7days. Cells were utilized for Aldefluor assay and stained for Annexin V-APC and DAPI for apoptosis assay. C. 100k pTRIPZ-HCC1954-mir93 cells were injected into the 4th fatpads of NOD/SCID mice. The treatment started as indicated by the red arrow. DOX alone (1 mg/ml in drinking water), or docetaxel (10 mg/kg i.p. once weekly) alone, or the combination inhibits SUM159 tumor growth in vivo. D. Tumors from each group were collected. ALDH was accessed by the Aldefluor assay on viable dissociated cells and by ALDH1 immunohistochemistry on fixed sections. E. Serial dilutions of cells obtained from these xenografts were implanted in the 4th fatpads of secondary mice, which received no further treatment. F. 10k pTRIPZ-HCC1954-mir93 cells were injected into the 4th fatpads of NOD/SCID mice. The treatment started immediately after injection as indicated by the red arrow and stopped as indicated by the green arrow. G. 200k pTRIPZ-HCC1954-mir93-luc cells in 100 ul of PBS were injected into the left ventricle of NOD/SCID mice. The treatment started immediately after injection as indicated by the red arrow and stopped as indicated by the green arrow. Metastasis formation was monitored using bioluminescence imaging. Quantification of the normalized photon flux, measured at weekly intervals following inoculation. H. Histologic confirmation, by H&E staining, of metastasis in soft tissues resulting from mice with different treatments in G. *p<0.05; Error bars represent mean ± STDEV. The colored “*” on the side of the tumor growth or metastasis curve represents the tumor growth is significantly diff [file pgen.1002751.s001.pdf]

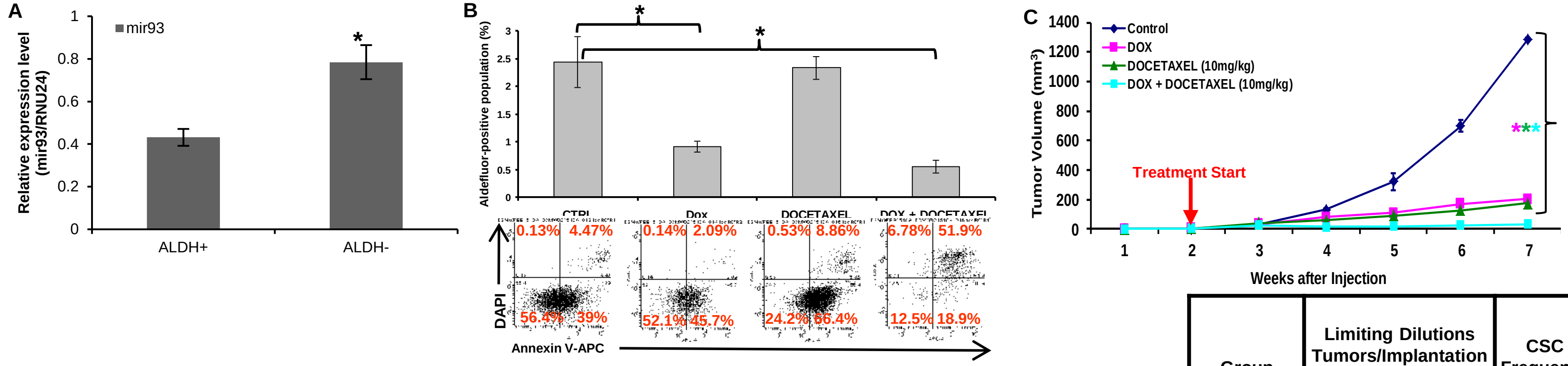

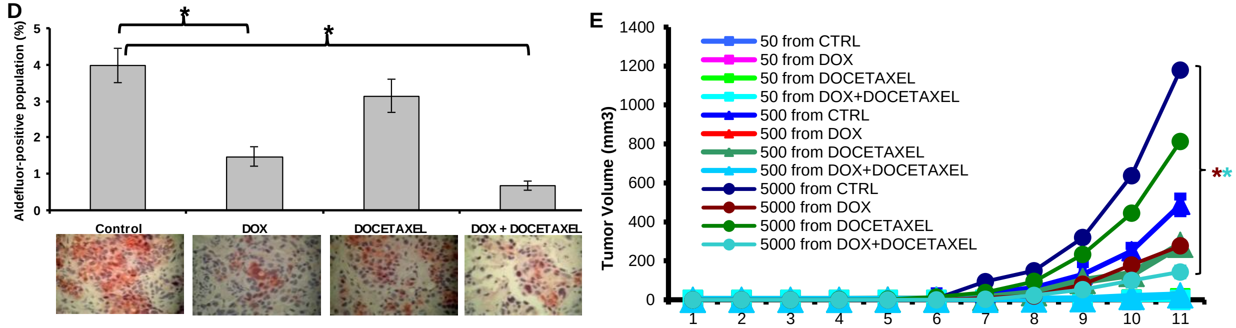

Group	Limiting Dilutions Tumors/Implantation			CSC Frequency (1 in/..)
	5000	500	50	
CTRL	5/5	5/5	5/5	1
DOX	5/5	5/5	0/5	209
DOCETAXEL	5/5	5/5	5/5	1
DOX+ DOCETAXEL	5/5	4/5	0/5	385

Pairwise groups difference		P Value
CTRL	DOX	0.00117
CTRL	DOCETAXEL	1
CTRL	DOX+DOCETAXEL	4.15E-05
DOX	DOCETAXEL	0.00117
DOX	DOX+DOCETAXEL	0.426
DOCETAXEL	DOX+DOCETAXEL	4.15E-05

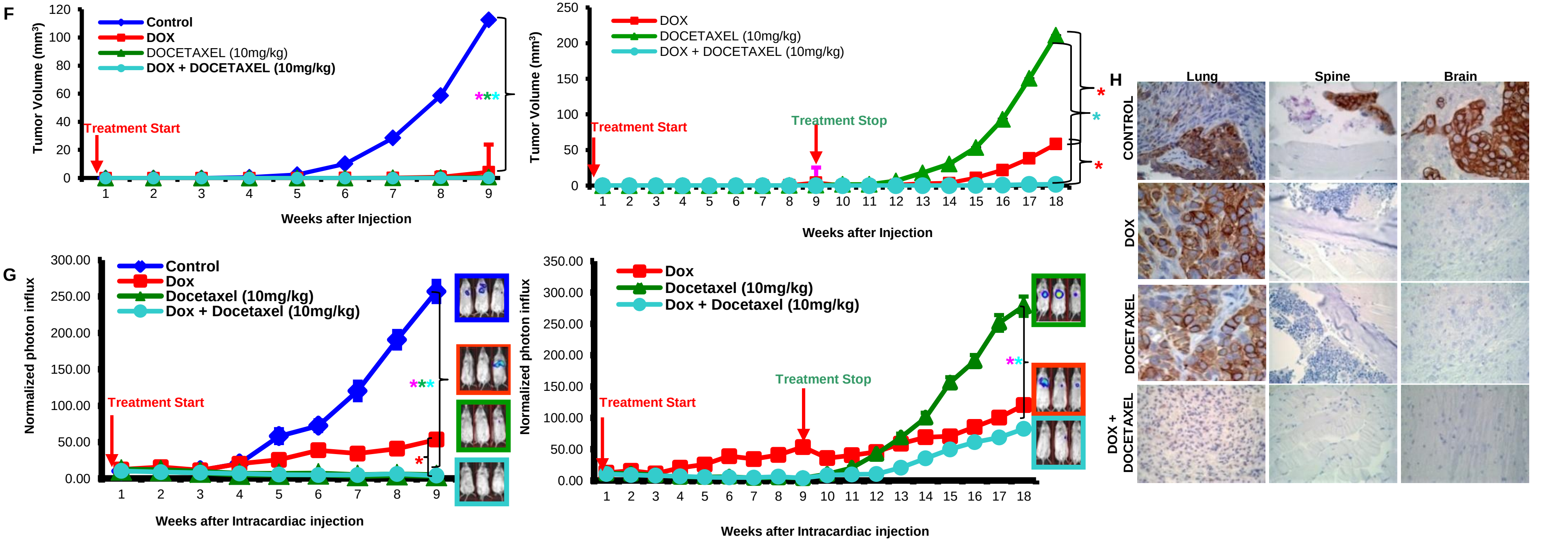

Figure S1. mir93 inhibits tumor growth and metastasis by decreasing CSCs in HCC1954 cells

A. ALDH-positive cells from HCC1954 cells shows lower mir93 expression level in comparison to ALDH-negative cells by qRT-PCR. **B**. 1x10^6 pTRIPZ-HCC1954-mir93 cells were plated in T75 flasks and, after overnight, the cells were treated with Vehicle control, DOX (1ug/ml), docetaxel (10 nM) or the combination for 3-7days. Cells were utilized for Aldefluor assay and stained for Annexin V-APC and DAPI for apoptosis assay. **C**. 100k pTRIPZ-HCC1954-mir93 cells were injected into the 4th fatpads of NOD/SCID mice. The treatment started as indicated by the red arrow. DOX alone (1mg/ml in drinking water), or docetaxel (10 mg/kg i.p. once weekly) alone, or the combination inhibits SUM159 tumor growth in vivo. **D**. Tumors from each group were collected. ALDH was accessed by the Aldefluor assay on viable dissociated cells and by ALDH1 immunohistochemistry on fixed sections. **E**. Serial dilutions of cells obtained from these xenografts were implanted in the 4th fatpads of secondary mice, which received no further treatment. **F**. 10k pTRIPZ-HCC1954-mir93 cells were injected into the 4th fatpads of NOD/SCID mice. The treatment started immediately after injection as indicated by the red arrow and stopped as indicated by the green arrow. **G**. 200k pTRIPZ-HCC1954-mir93-luc cells in 100ul of PBS were injected into the left ventricle of NOD/SCID mice. The treatment started immediately after injection as indicated by the red arrow and stopped as indicated by the green arrow. Metastasis formation was monitored using bioluminescence imaging. Quantification of the normalized photon flux, measured at weekly intervals following inoculation. **H**. Histologic confirmation, by H&E staining, of metastasis in soft tissues resulting from mice with different treatments in **G**.

$p<0.05$; Error bars represent mean ± STDEV. The colored “” on the side of the tumor growth or metastasis curve represents the tumor growth is significantly different between Control group and the group with the same colored curve.
