# Supplementary material for: MicroRNA93 Regulates Proliferation and Differentiation of Normal and Malignant Breast Stem Cells
Source: PLoS Genet. 2012 Jun 7;8(6):e1002751. doi: 10.1371/journal.pgen.1002751 (PMC3369932; doi:10.1371/journal.pgen.1002751)
Supplement: Figure S2 — mir-93 is induced in primary tumors with DOX. 100k pTRIPZ-SUM159-mir-93 cells were injected into the 4th fatpads of NOD/SCID mice. Different treatments were initiated (Vehicle Control (Control), DOX alone (DOX), docetaxel alone, or the combination). At the end of treatment, cells from Control and DOX groups were isolated from the tumors and mir-93 expression level was measured by qRT-PCR. (PDF) [file pgen.1002751.s002.pdf]

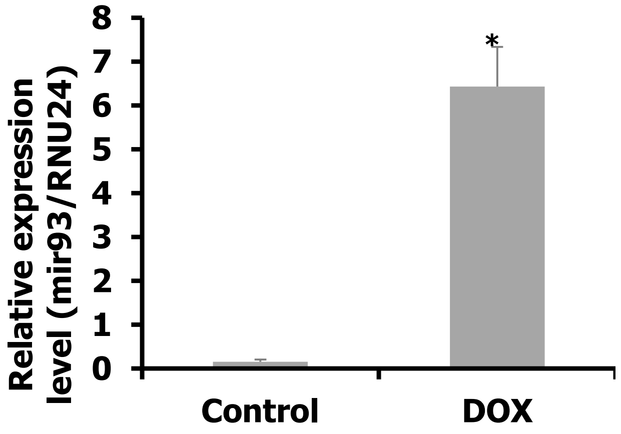

Figure S2. mir-93 is induced in primary tumors with DOX. 100k pTRIPZ-SUM159-mir-93 cells were injected into the 4th fatpads of NOD/SCID mice. Different treatments were initiated (Vehicle Control (Control), DOX alone (DOX), docetaxel alone, or the combination). At the end of treatment, cells from Control and DOX groups were isolated from the tumors and mir-93 expression level was measured by qRT-PCR.
