# Supplementary material for: MicroRNA93 Regulates Proliferation and Differentiation of Normal and Malignant Breast Stem Cells
Source: PLoS Genet. 2012 Jun 7;8(6):e1002751. doi: 10.1371/journal.pgen.1002751 (PMC3369932; doi:10.1371/journal.pgen.1002751)
Supplement: Figure S3 — Induction of mir93 decreases both ALDH+ and CD24−CD44+ cells in the primary breast tumor xenografts. Cells isolated from primary human breast xenografts UM2 (A), MC1 (B) and UM1 (C) were sorted for ALDH+ and ALDH− or CD24−CD44+ and the rest (CD24−CD44−, CD24+CD44+, CD24+CD44−). RNA was isolated from each group of sorted cells and the expression level of mir-93 or RNU24 level was measured by qRT-PCR. *p<0.05; Error bars represent mean ± STDEV. (PDF) [file pgen.1002751.s003.pdf]

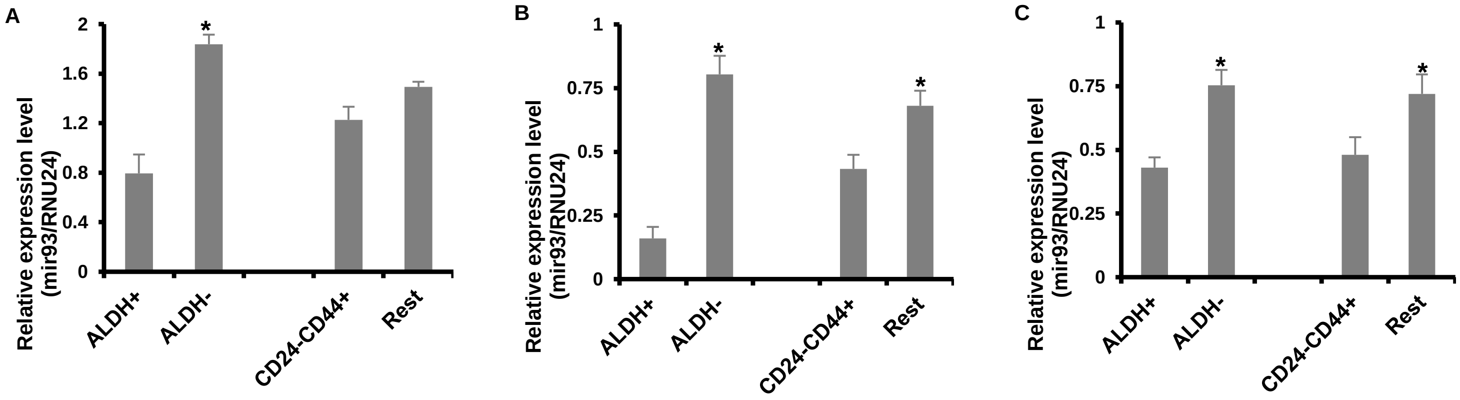

Figure S3. Induction of mir93 decreases both ALDH^{+} and CD24^{-}CD44^{+} cells in the primary breast tumor xenografts. Cells isolated from primary human breast xenografts UM2 (**A**), MC1 (**B**) and UM1 (**C**) were sorted for ALDH^{+} and ALDH^{-} or CD24^{-}CD44^{+} and the rest (CD24^{-}CD44^{-}, CD24^{+}CD44^{+}, CD24^{+}CD44^{-}). RNA was isolated from each group of sorted cells and the expression level of mir-93 or RNU24 level was measured by qRT-PCR. *$p<0.05$; Error bars represent mean ± STDEV.
