# Supplementary material for: MicroRNA93 Regulates Proliferation and Differentiation of Normal and Malignant Breast Stem Cells
Source: PLoS Genet. 2012 Jun 7;8(6):e1002751. doi: 10.1371/journal.pgen.1002751 (PMC3369932; doi:10.1371/journal.pgen.1002751)
Supplement: Figure S4 — mir-93 expression in Sensor-GFP-positive and Sensor-GFP-negative SUM159 cells. Mir-93-sensor-GFP SUM159 cells were sorted for GFP-positive and GFP-negative cells by flow cytometry and RNA was isolated from both group of sorted cells and the expression level of mir-93 or RNU24 level was measured by qRT-PCR. *p<0.05; Error bars represent mean ± STDEV. (PDF) [file pgen.1002751.s004.pdf]

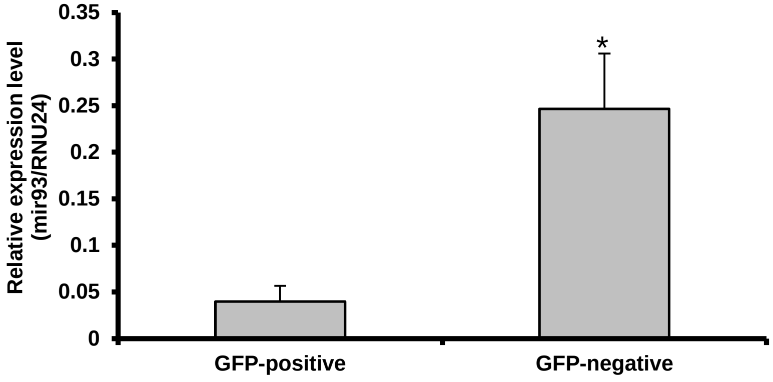

Figure S4. mir-93 expression in Sensor-GFP-positive and Sensor-GFP-negative SUM159 cells.
Mir-93-sensor-GFP SUM159 cells were sorted for GFP-positive and GFP-negative cells by flow cytometry and RNA was isolated from both group of sorted cells and the expression level of mir-93 or RNU24 level was measured by qRT-PCR. *$p<0.05$; Error bars represent mean ± STDEV.
