# Supplementary material for: MicroRNA93 Regulates Proliferation and Differentiation of Normal and Malignant Breast Stem Cells
Source: PLoS Genet. 2012 Jun 7;8(6):e1002751. doi: 10.1371/journal.pgen.1002751 (PMC3369932; doi:10.1371/journal.pgen.1002751)
Supplement: Figure S5 — ALDH1A1 protein level in Sensor-GFP-positive and Sensor-GFP-negative SUM159 cells. mir-93-sensor-GFP SUM159 cells were sorted for GFP-positive and GFP-negative cells by flow cytometry. A portion of cells were utilized for western blot, and some cells were cytospun down and stained with ALDH1A1 by immunohistochemical staining. *p<0.05; Error bars represent mean ±STDEV. (PDF) [file pgen.1002751.s005.pdf]

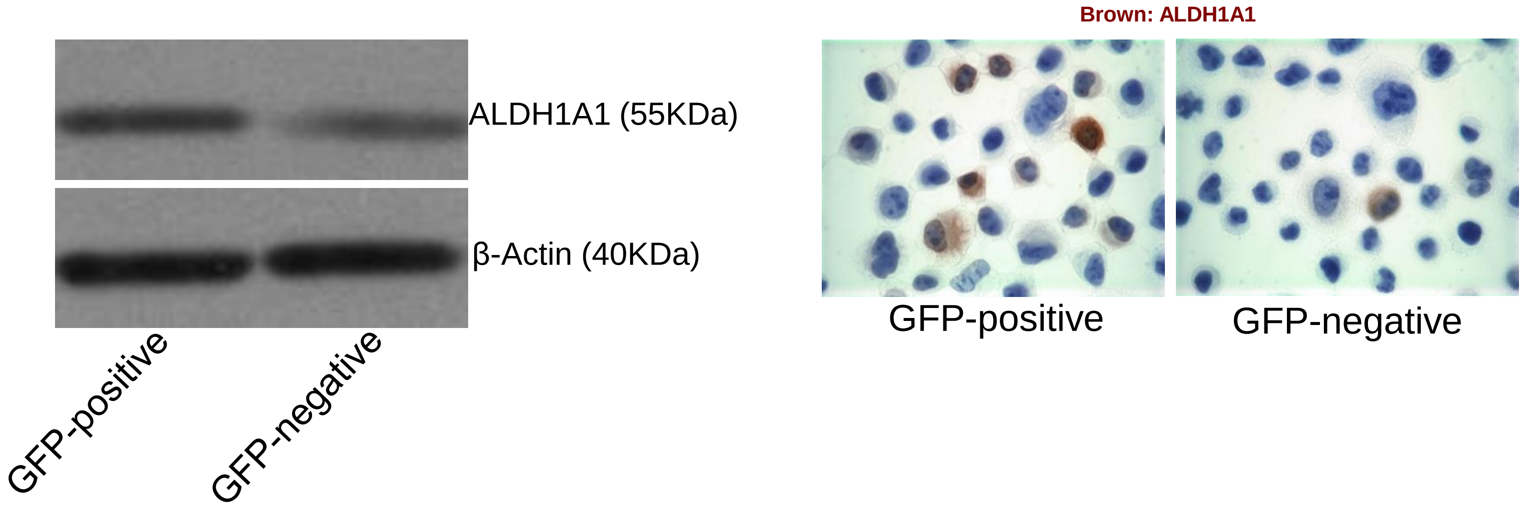

Figure S5. ALDH1A1 protein level in Sensor-GFP-positive and Sensor-GFP-negative SUM159 cells.
mir-93-sensor-GFP SUM159 cells were sorted for GFP-positive and GFP-negative cells by flow cytometry. A portion of cells were utilized for western blot, and some cells were cytospun down and stained with ALDH1A1 by immunohistochemical staining. $*p<0.05$; Error bars represent mean ± STDEV.
