# Supplementary material for: MicroRNA93 Regulates Proliferation and Differentiation of Normal and Malignant Breast Stem Cells
Source: PLoS Genet. 2012 Jun 7;8(6):e1002751. doi: 10.1371/journal.pgen.1002751 (PMC3369932; doi:10.1371/journal.pgen.1002751)
Supplement: Figure S6 — mir-93 induction reduces GFP in mir93-sensor-GFP-SUM159 cells. mir93-sensor-GFP-SUM159 cells were infected with pTRIPZ-mir93 lentivirus and grow in T75 flasks. DOX were added to the culture medium in the DOX group. Images were taken with fluorescence microscope. (PDF) [file pgen.1002751.s006.pdf]

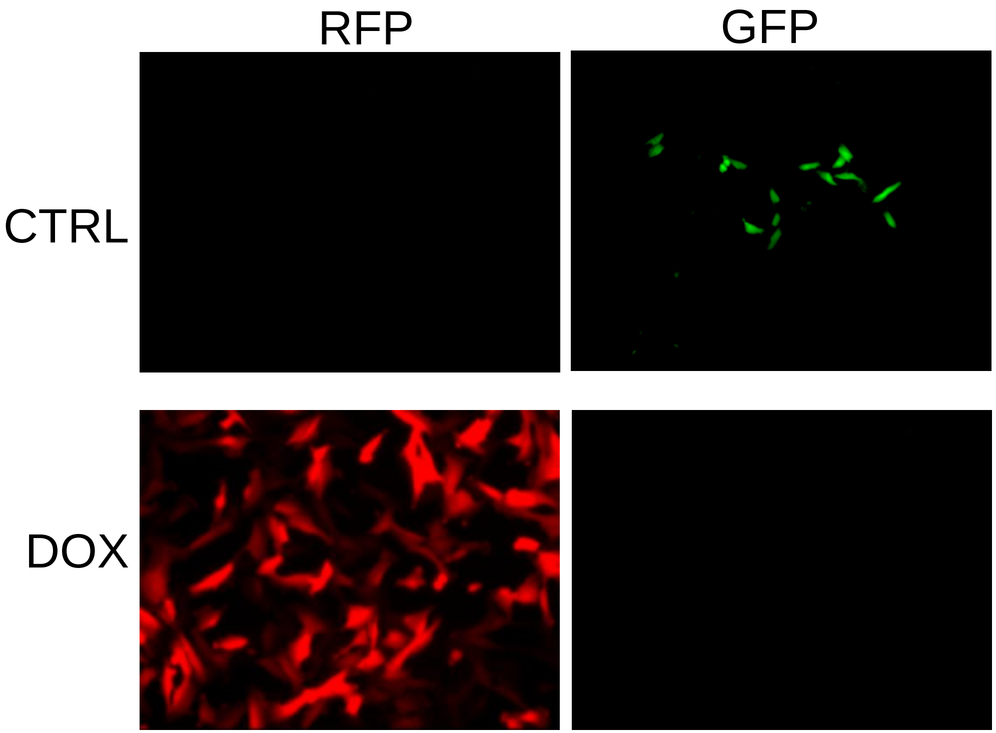

Figure S6. mir-93 induction reduces GFP in mir93-sensor-GFP-SUM159 cells.
mir93-sensor-GFP-SUM159 cells were infected with pTRIPZ-mir93 lentivirus and grow in T75 flasks. DOX were added to the culture medium in the DOX group. Images were taken with fluorescence microscope.
