# Supplementary material for: MicroRNA93 Regulates Proliferation and Differentiation of Normal and Malignant Breast Stem Cells
Source: PLoS Genet. 2012 Jun 7;8(6):e1002751. doi: 10.1371/journal.pgen.1002751 (PMC3369932; doi:10.1371/journal.pgen.1002751)
Supplement: Figure S7 — 1×10^6^ pTRIPZ-SUM149-mir93 cells were plated in T75 flasks and, after overnight, the cells were treated with Vehicle control or DOX (1 ug/ml) for 3–7 days. Induction of mir93 expression by DOX decreased the ALDH-positive population. *p<0.05; Error bars represent mean ± STDEV. (PDF) [file pgen.1002751.s007.pdf]

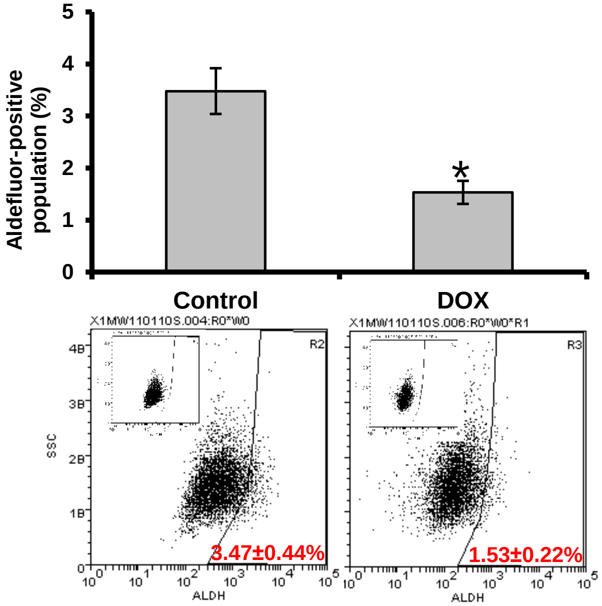

Figure S7. 1x10^{6} pTRIPZ-SUM149-mir93 cells were plated in T75 flasks and, after overnight, the cells were treated with Vehicle control or DOX (1ug/ml) for 3-7days. Induction of mir93 expression by DOX decreased the ALDH-positive population. *p<0.05; Error bars represent mean ± STDEV.
