# Supplementary material for: MicroRNA93 Regulates Proliferation and Differentiation of Normal and Malignant Breast Stem Cells
Source: PLoS Genet. 2012 Jun 7;8(6):e1002751. doi: 10.1371/journal.pgen.1002751 (PMC3369932; doi:10.1371/journal.pgen.1002751)
Supplement: Figure S8 — Modulation of mir93 level in SUM159 cells altered cell invasion in vitro and knockdown increases tumor growth in vivo. SUM159 cells were infected with no virus (non-infection), DsRed control lentivirus (SUM159-Neg-DsRed) or mirZip antisense mir93 lentivirus (SUM159-mirZip93-DsRed). A. microRNA RT-PCR demonstrated SUM159-mirZip93-DsRed reduced mir93 expression by more than 90%. B. SUM159-non-infection, SUM159-Neg-DsRed, SUM159-mirZip93-DsRed cells were grown in T75 flasks and Aldefluor assay was utilized to measure the percentage of ALDH+ cells. C. Serial dilutions of cells were implanted in the 4th fatpads of NOD/SCID mice. SUM159-mirZip93-DsRed cells initiated tumors sooner and accelerated growth compared to equivalent number of control cells. D. Cells were isolated from the tumors in C and Aldefluor assay was utilized to measure the percentage of ALDH+ cells. E. Invasive capacity was assessed by a Matrigel invasion assay using serum as attractant. pTRIPZ-SUM159-mir93 cells are less invasive than the control cells in vitro accesses at 27 hours. F. The invasion was assessed by a Matrigel invasion assay using serum as attractant. SUM159-mirZip93-DsRed cells are more invasive than the control cells in vitro accesses at 27 hours. *p<0.05; Error bars represent mean ± STDEV. The colored “*” on the side of the tumor growth curve represents the tumor growth is significantly different between Control group and the group with the same colored curve. (PDF) [file pgen.1002751.s008.pdf]

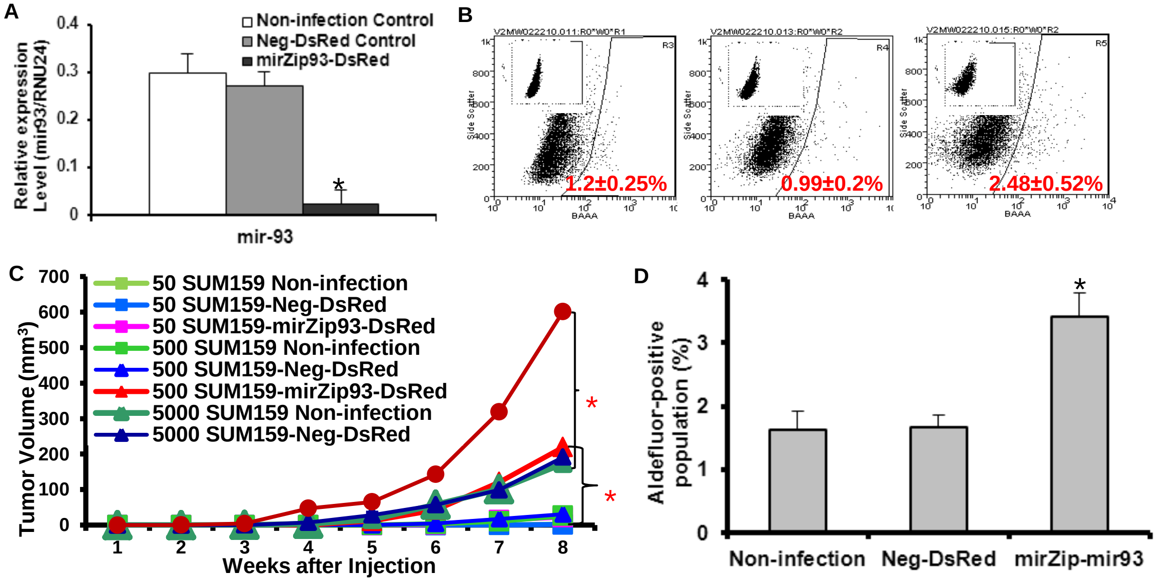

Group	Limiting Dilutions Tumors/Implantation			CSC Frequency (1 in/..)	Pairwise groups difference		*P Value*
	5000	500	50				
Non-infection	5/5	4/5	3/5	180	Non-infection	Neg-DsRed	1
Neg-DsRed	5/5	4/5	3/5	1	Non-infection	mirZip93-DsRed	7.60E-04
mirZip93-DsRed	5/5	5/5	5/5	180	Neg-DsRed	mirZip93-DsRed	0.00076

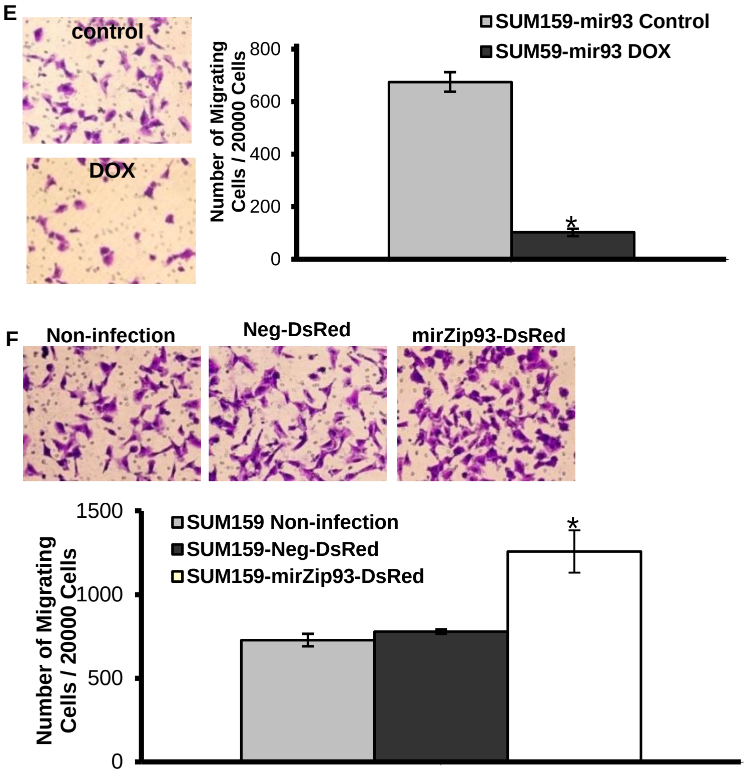

Figure S8. Modulation of mir93 level in SUM159 cells altered cell invasion in vitro and knockdown increases tumor growth in vivo

SUM159 cells were infected with no virus (non-infection), DsRed control lentivirus (SUM159-Neg-DsRed) or mirZip antisense mir93 lentivirus (SUM159-mirZip93-DsRed). **A**. microRNA RT-PCR demonstrated SUM159-mirZip93-DsRed reduced mir93 expression by more than 90%. **B**. SUM159-non-infection, SUM159-Neg-DsRed, SUM159-mirZip93-DsRed cells were grown in T75 flasks and Aldefluor assay was utilized to measure the percentage of $ALDH^+$ cells. **C**. Serial dilutions of cells were implanted in the 4th fatpads of NOD/SCID mice. SUM159-mirZip93-DsRed cells initiated tumors sooner and accelerated growth compared to equivalent number of control cells. **D**. Cells were isolated from the tumors in **C** and Aldefluor assay was utilized to measure the percentage of $ALDH^+$ cells. E. Invasive capacity was assessed by a Matrigel invasion assay using serum as attractant. pTRIPZ-SUM159-mir93 cells are less invasive than the control cells in vitro accesses at 27 hours. **F**. The invasion was assessed by a Matrigel invasion assay using serum as attractant. SUM159-mirZip93-DsRed cells are more invasive than the control cells in vitro accesses at 27 hours.

p<0.05; Error bars represent mean ± STDEV. The colored "" on the side of the tumor growth curve represents the tumor growth is significantly different between Control group and the group with the same colored curve.
