# Supplementary material for: MicroRNA93 Regulates Proliferation and Differentiation of Normal and Malignant Breast Stem Cells
Source: PLoS Genet. 2012 Jun 7;8(6):e1002751. doi: 10.1371/journal.pgen.1002751 (PMC3369932; doi:10.1371/journal.pgen.1002751)
Supplement: Figure S9 — mir-93 inhibits tumor metastasis in SUM159 cells. 200k pTRIPZ-SUM159-mir-93-Luc cells in 100 ul of PBS were injected into the left ventricle of NOD/SCID mice. Different treatments were initiated (Vehicle Control (Control), DOX alone (DOX), docetaxel alone, or the combination). At the end of treatment, H&E staining and Pan-cytokeratin (AE1/AE3) staining (in Brown) were performed to confirm the metastasis in bone and soft tissues resulting from mice with different treatments. (PDF) [file pgen.1002751.s009.pdf]

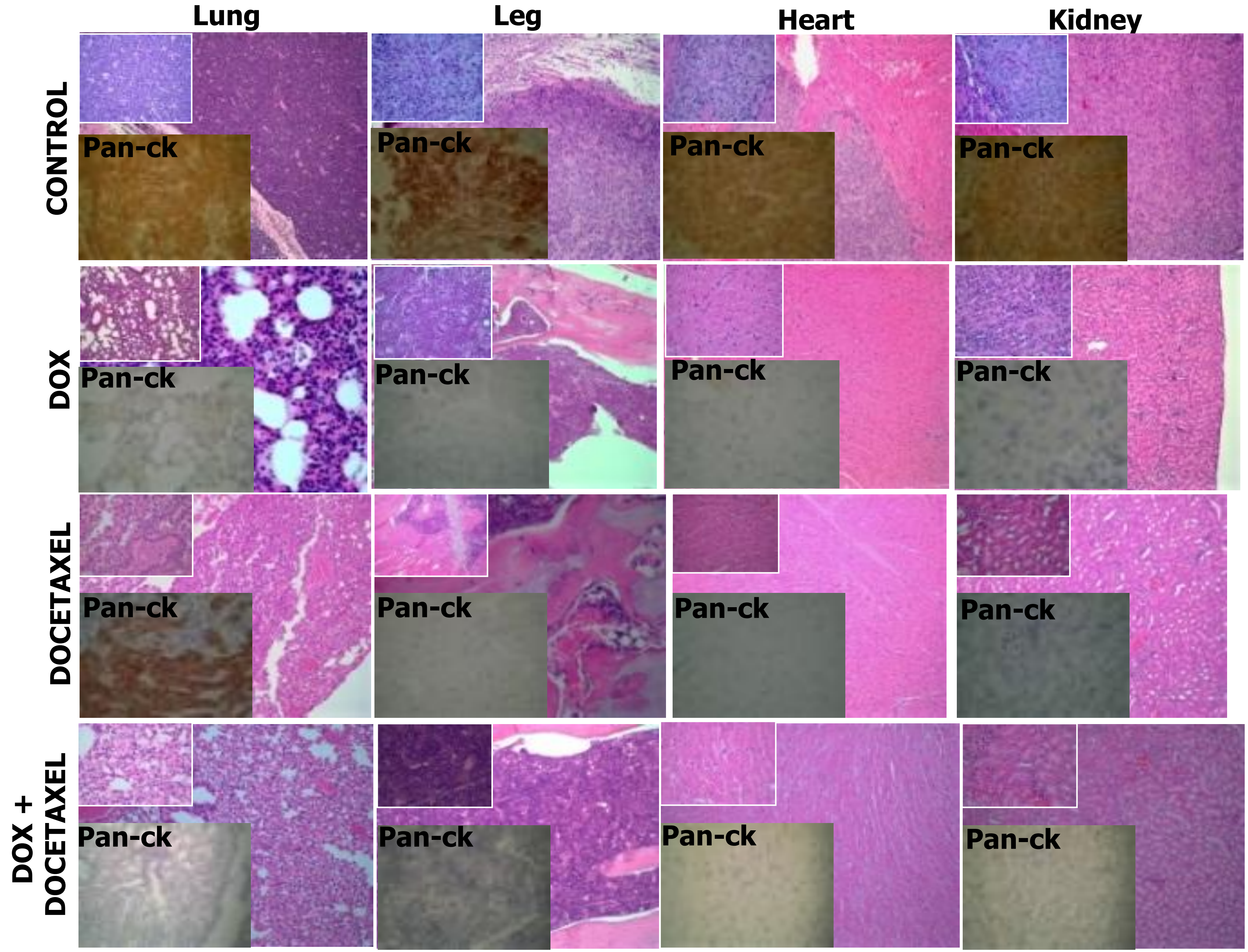

Figure S9. mir-93 inhibits tumor metastasis in SUM159 cells. 200k pTRIPZ-SUM159-mir-93-Luc cells in 100ul of PBS were injected into the left ventricle of NOD/SCID mice. Different treatments were initiated (Vehicle Control (Control), DOX alone (DOX), docetaxel alone, or the combination). At the end of treatment, H&E staining and Pan-cytokeratin (AE1/AE3) staining (in Brown) were performed to confirm the metastasis in bone and soft tissues resulting from mice with different treatments.
