# Supplementary material for: MicroRNA93 Regulates Proliferation and Differentiation of Normal and Malignant Breast Stem Cells
Source: PLoS Genet. 2012 Jun 7;8(6):e1002751. doi: 10.1371/journal.pgen.1002751 (PMC3369932; doi:10.1371/journal.pgen.1002751)
Supplement: Figure S10 — mir93 inhibits tumor growth in primary human breast xenografts MC1, UM2, and UM1. Cells isolated from primary xenografts MC1 (A) or UM2 (B) or UM1 (C) were transduced with the pTRIPZ-mir93 lentivirus in suspension. 10k pTRIPZ-MC1-mir93 or pTRIPZ-UM2-mir93 cells were injected into the 4th fatpads of NOD/SCID mice. The treatment started right after injection as indicated by the red arrow. DOX alone, docetaxel alone or the combination prevented tumor growth. *p<0.05; Error bars represent mean ± STDEV. The colored “*” on the side of the tumor growth curve represents the tumor growth is significantly different between Control group and the group with the same colored curve. (PDF) [file pgen.1002751.s010.pdf]

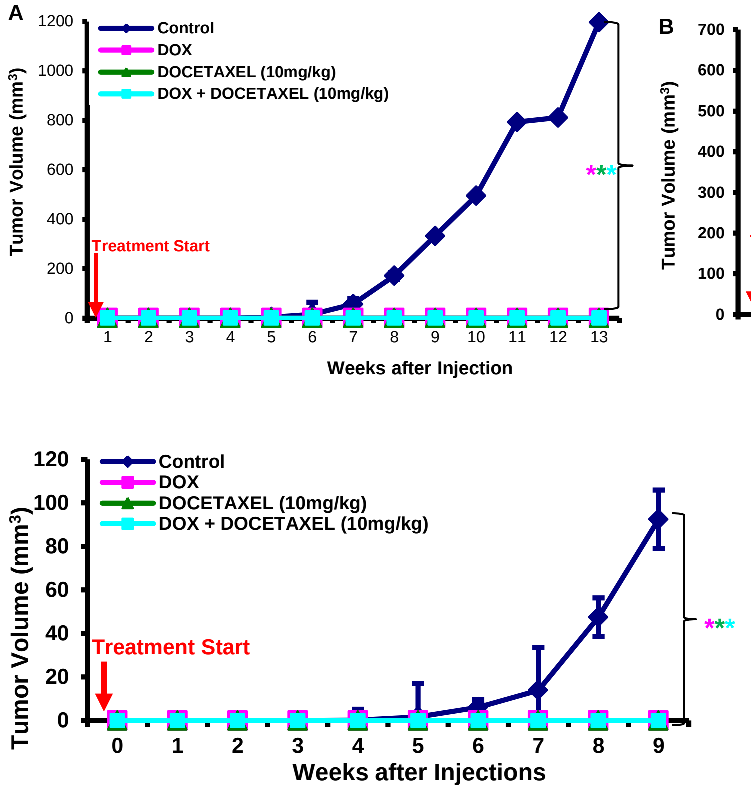

Figure S10. mir93 inhibits tumor growth in primary human breast xenografts MC1, UM2 and UM1
Cells isolated from primary xenografts MC1 (**A**) or UM2 (**B**) or UM1 (**C**) were transduced with the pTRIPZ-mir93 lentivirus in suspension. 10k pTRIPZ-MC1-mir93 or pTRIPZ-UM2-mir93 cells were injected into the 4th fatpads of NOD/SCID mice. The treatment started right after injection as indicated by the red arrow. DOX alone, docetaxel alone or the combination prevented tumor growth. *p<0.05; Error bars represent mean ± STDEV. The colored "*" on the side of the tumor growth curve represents the tumor growth is significantly different between Control group and the group with the same colored curve.
