# Supplementary material for: MicroRNA93 Regulates Proliferation and Differentiation of Normal and Malignant Breast Stem Cells
Source: PLoS Genet. 2012 Jun 7;8(6):e1002751. doi: 10.1371/journal.pgen.1002751 (PMC3369932; doi:10.1371/journal.pgen.1002751)
Supplement: Figure S11 — Endogenous mir93 expression levels parallel cell differentiation state. ALDH+ population and ALDH− population were separated from SUM159 cells and HCC1954 cells. CD24−CD44+ population and the remaining cell populations were separated from MCF7 cells. mir93 level was analyzed by microRNA qRT-PCR. Among these three cell lines, mir93 expression level is highest in MCF7 cells and lowest in the SM159 cells. In both SUM159 cells and HCC1954 cells, ALDH+ cells have lower mir93 expression compared to ALDH− cells. In contrast, CD24−CD44+ in MCF7 cells showed no difference for mir93 expression level in comparison to the bulk population. *p<0.05; Error bars represent mean ± STDEV. (PDF) [file pgen.1002751.s011.pdf]

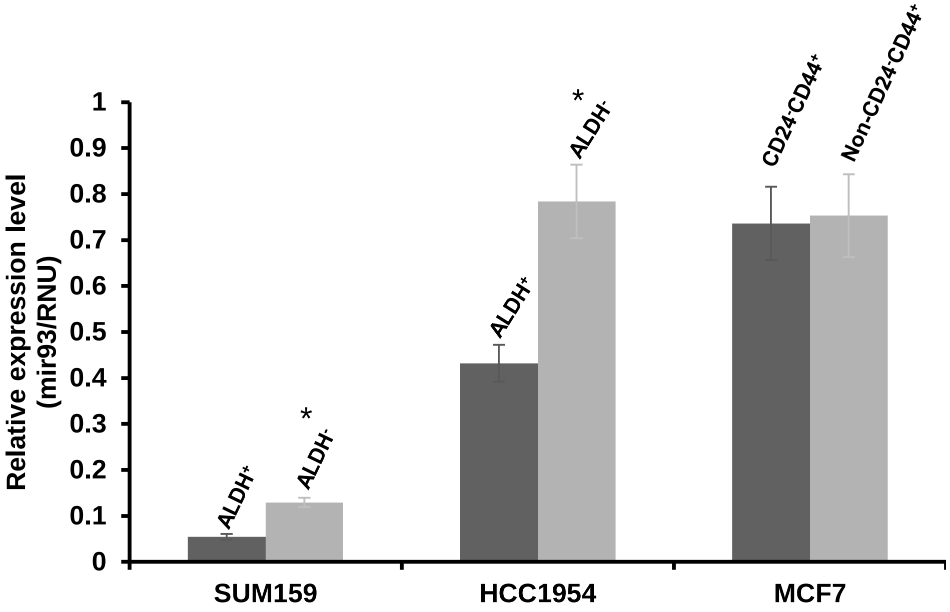

Figure S11. Endogenous mir93 expression levels parallel cell differentiation state

$ALDH^{+}$ population and $ALDH^{-}$ population were separated from SUM159 cells and HCC1954 cells. $CD24^{-}CD44^{+}$ population and the remaining cell populations were separated from MCF7 cells. mir93 level was analyzed by microRNA qRT-PCR. Among these three cell lines, mir93 expression level is highest in MCF7 cells and lowest in the SM159 cells. In both SUM159 cells and HCC1954 cells, $ALDH^{+}$ cells have lower mir93 expression compared to $ALDH^{-}$ cells. In contrast, $CD24^{-}CD44^{+}$ in MCF7 cells showed no difference for mir93 expression level in comparison to the bulk population.

*$p<0.05$; Error bars represent mean ± STDEV.
