# Supplementary material for: MicroRNA93 Regulates Proliferation and Differentiation of Normal and Malignant Breast Stem Cells
Source: PLoS Genet. 2012 Jun 7;8(6):e1002751. doi: 10.1371/journal.pgen.1002751 (PMC3369932; doi:10.1371/journal.pgen.1002751)
Supplement: Figure S12 — mir93 promotes tumor growth by increasing CSCs in MDA-MB-453 cells. A. 200k pTRIPZ-MDA-MB-453-mir93 cells were injected into the 4th fatpads of NOD/SCID mice. Treatment was initiated as indicated by the red arrow. DOX (1 mg/ml in drinking water) promoted MDA-MB-453 tumor growth in vivo. B. Tumors from each group were collected. Aldefluor assay was performed on dissociated cells. DOX increased the ALDH+ populations in MDA-MB-453. C. Serial dilutions of cells obtained from these xenografts were implanted in the 4th fatpads of secondary mice, which received no further treatment. Cells from DOX-treated tumors formed secondary tumors at all dilutions (1k, 10k, 32k), whereas only higher numbers of cells (32k) obtained from control xenografts were able to generate tumors. *p<0.05; Error bars represent mean ± STDEV. (PDF) [file pgen.1002751.s012.pdf]

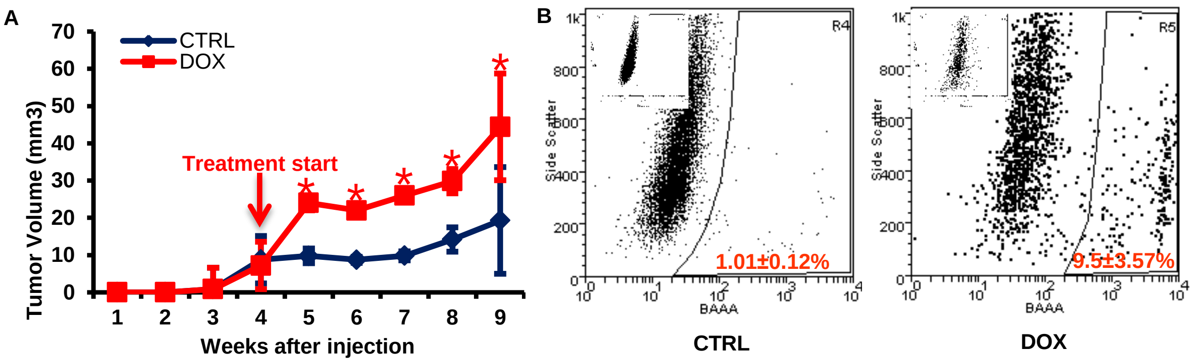

C

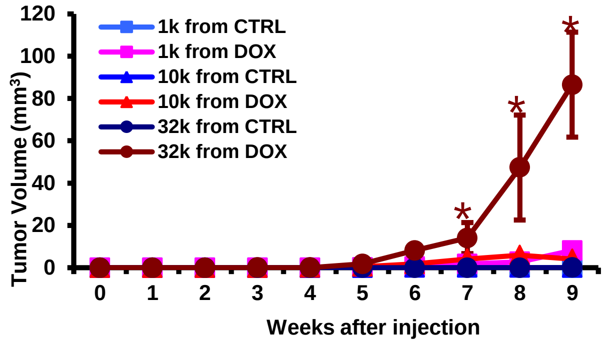

Group	Limiting Dilutions Tumors/Implantation			CSC Frequency (1 in/..)	P Value
	32000	10000	1000		
CTRL	3/5	2/5	0/5	29590	0.000613
DOX	5/5	4/5	3/5	3592	

Figure S12. mir93 promotes tumor growth by increasing CSCs in MDA-MB-453 cells

A. 200k pTRIPZ-MDA-MB-453-mir93 cells were injected into the 4th fatpads of NOD/SCID mice. Treatment was initiated as indicated by the red arrow. DOX (1mg/ml in drinking water) promoted MDA-MB-453 tumor growth in vivo. **B**. Tumors from each group were collected. Aldefluor assay was performed on dissociated cells. DOX increased the ALDH^{+} populations in MDA-MB-453. **C**. Serial dilutions of cells obtained from these xenografts were implanted in the 4th fatpads of secondary mice, which received no further treatment. Cells from DOX-treated tumors formed secondary tumors at all dilutions (1k, 10k, 32k), whereas only higher numbers of cells (32k) obtained from control xenografts were able to generate tumors. *$p<0.05$; Error bars represent mean ± STDEV.
