# Supplementary material for: MicroRNA93 Regulates Proliferation and Differentiation of Normal and Malignant Breast Stem Cells
Source: PLoS Genet. 2012 Jun 7;8(6):e1002751. doi: 10.1371/journal.pgen.1002751 (PMC3369932; doi:10.1371/journal.pgen.1002751)
Supplement: Figure S13 — mir93 promotes tumor growth by increasing CSCs in T47D cells. A. 500k pTRIPZ-T47D-mir93 cells were injected into the 4th fatpads of NOD/SCID mice. Treatment was initiated as indicated by the red arrow. DOX (1 mg/ml in drinking water) promoted T47D tumor growth in vivo. B. Tumors from each group were collected. Aldefluor assay was performed on dissociated cells. DOX increased the ALDH+ populations in T47D. C. Serial dilutions of cells obtained from these xenografts were implanted in the 4th fatpads of secondary mice, which received no further treatment. Cells from DOX-treated tumors formed secondary tumors at all dilutions (5k, 50k, 500k). *p<0.05; Error bars represent mean ± STDEV. (PDF) [file pgen.1002751.s013.pdf]

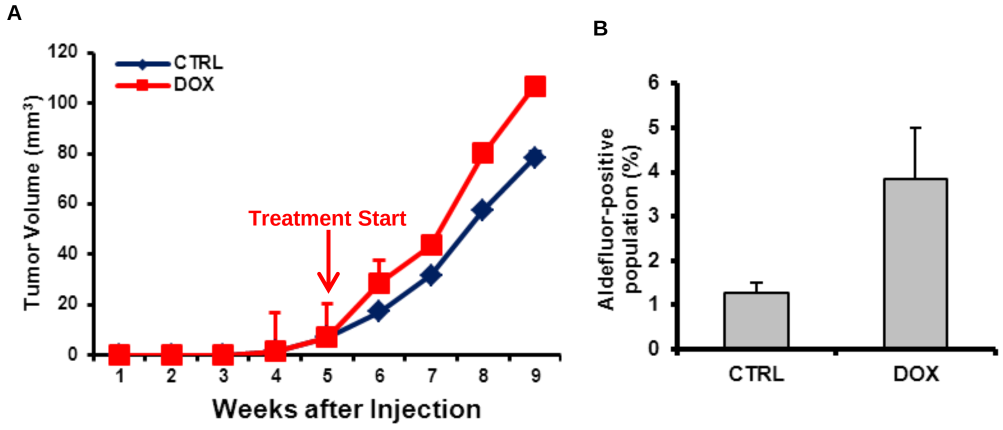

C

Group	Limiting Dilutions Tumors/Implantation			CSC Frequency (1 in/..)	P Value
	500000	50000	5000		
CTRL	3/5	1/5	0/5	467056	0.0415
DOX	4/5	3/5	2/5	128614	

Figure S13. mir93 promotes tumor growth by increasing CSCs in T47D cells

A. 500k pTRIPZ-T47D-mir93 cells were injected into the 4th fatpads of NOD/SCID mice. Treatment was initiated as indicated by the red arrow. DOX (1mg/ml in drinking water) promoted T47D tumor growth in vivo. **B**. Tumors from each group were collected. Aldefluor assay was performed on dissociated cells. DOX increased the ALDH^{+} populations in T47D. **C**. Serial dilutions of cells obtained from these xenografts were implanted in the 4th fatpads of secondary mice, which received no further treatment. Cells from DOX-treated tumors formed secondary tumors at all dilutions (5k, 50k, 500k).

*p<0.05; Error bars represent mean ± STDEV.
