# Supplementary material for: MicroRNA93 Regulates Proliferation and Differentiation of Normal and Malignant Breast Stem Cells
Source: PLoS Genet. 2012 Jun 7;8(6):e1002751. doi: 10.1371/journal.pgen.1002751 (PMC3369932; doi:10.1371/journal.pgen.1002751)
Supplement: Figure S15 — Validation of the 127 overlapped gene expression with customerized StellArray PCR array plate in pTRIPZ-SUM159-mir93. (PDF) [file pgen.1002751.s015.pdf]

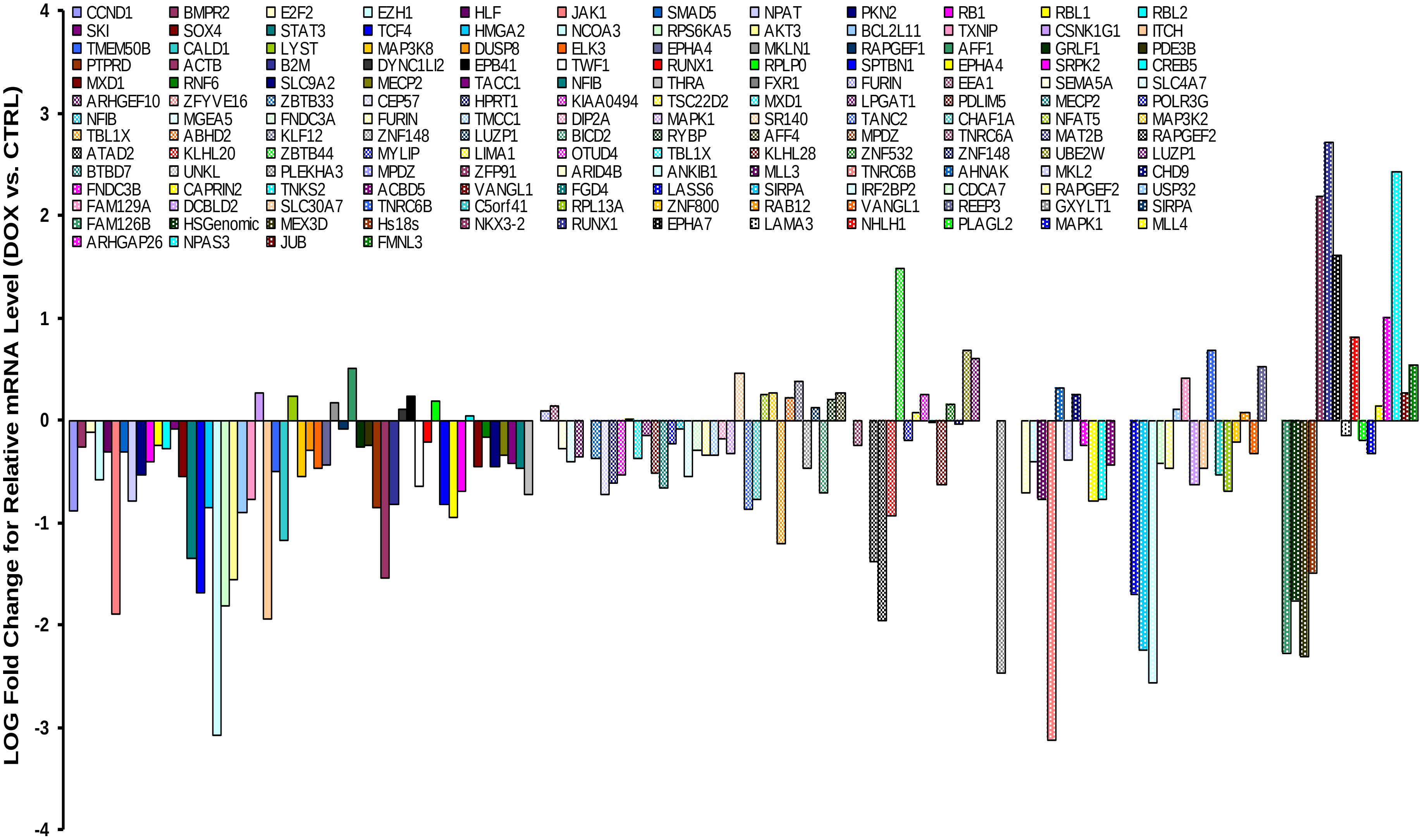

Figure S15. Validation of the 127 overlapped gene expression with customerized StellArray PCR array plate in pTRIPZ-SUM159-mir93.
