# Supplementary material for: MicroRNA93 Regulates Proliferation and Differentiation of Normal and Malignant Breast Stem Cells
Source: PLoS Genet. 2012 Jun 7;8(6):e1002751. doi: 10.1371/journal.pgen.1002751 (PMC3369932; doi:10.1371/journal.pgen.1002751)
Supplement: Figure S19 — Luciferase assay testing mir93 targets. The 3′UTR of AKT3, SOX4, and STAT3 pMIR-REPORT firefly luciferase reporter plasmids with the wild-type 3′UTR sequences of AKT3, SOX4, or STAT3 were transiently transfected into pTRIPZ-MCF7-mir93 (A) or pTRIPZ-MDA-MB-453-mir93 (B) cells and an internal control ACTB luciferase reporter was co-transfected for normalization. The cells were treated with or without DOX. Luciferase activities were measured after 48 hr. The relative luciferase activity is shown as the ratio of (the results from the cells transfected by individual reporter)/(the results from the cells transfected by the internal control in the same cell group). Error bars represent mean ± STDEV. (PDF) [file pgen.1002751.s019.pdf]

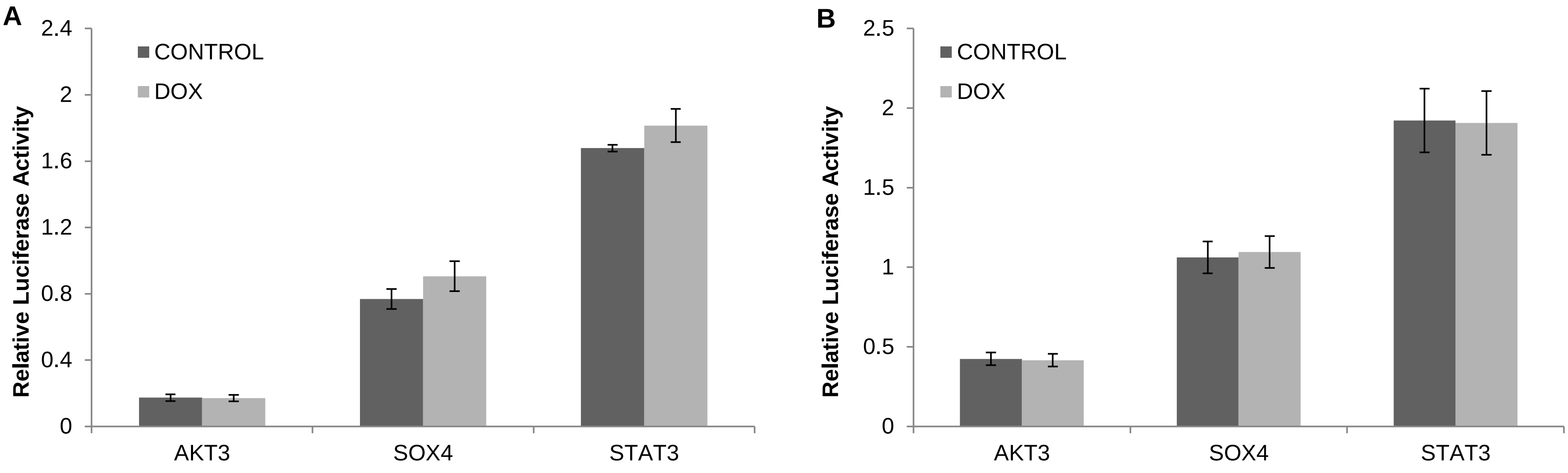

Figure S19. Luciferase assay testing mir93 targets
The 3'UTR of AKT3, SOX4, and STAT3 pMIR-REPORT^{TM} firefly luciferase reporter plasmids with the wild-type 3'UTR sequences of AKT3, SOX4, or STAT3 were transiently transfected into pTRIPZ-MCF7-mir93 (A) or pTRIPZ-MDA-MB-453-mir93 (B) cells and an internal control ACTB luciferase reporter was co-transfected for normalization. The cells were treated with or without DOX. Luciferase activities were measured after 48 hr. The relative luciferase activity is shown as the ratio of (the results from the cells transfected by individual reporter) / (the results from the cells transfected by the internal control in the same cell group). Error bars represent mean ± STDEV.
