# Supplementary material for: MicroRNA93 Regulates Proliferation and Differentiation of Normal and Malignant Breast Stem Cells
Source: PLoS Genet. 2012 Jun 7;8(6):e1002751. doi: 10.1371/journal.pgen.1002751 (PMC3369932; doi:10.1371/journal.pgen.1002751)
Supplement: Figure S20 — Knockdown of STAT3 (A), AKT3 (B) or SOX4 (C) decreases ALDH+ cells in SUM159 cells. SUM159 cells were transfected with PlentiLox3.7-shRNA-DsRed viruses and accessed for the ALDH+ population by Aldeflour assay. *p<0.05; Error bars represent mean ± STDEV. (PDF) [file pgen.1002751.s020.pdf]

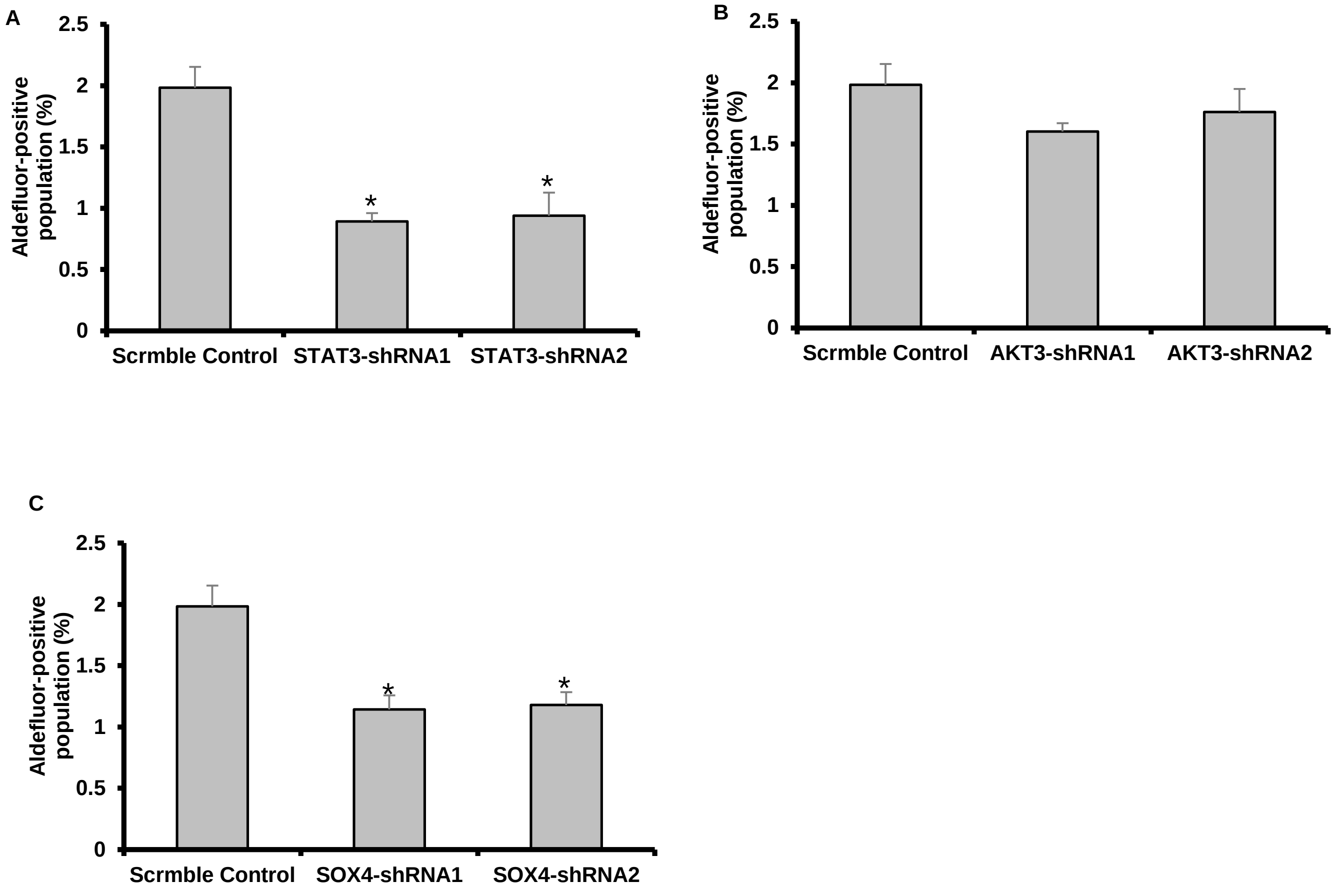


**Figure S20. Knockdown of STAT3 (A), AKT3 (B) or SOX4 (C) decreases ALDH⁺ cells in SUM159 cells.**
SUM159 cells were transfected with PlentiLox3.7-shRNA-DsRed viruses and accessed for the $ALDH^+$ population by Aldeflour assay. *$p<0.05$; Error bars represent mean ± STDEV.
