# Supplementary material for: MicroRNA93 Regulates Proliferation and Differentiation of Normal and Malignant Breast Stem Cells
Source: PLoS Genet. 2012 Jun 7;8(6):e1002751. doi: 10.1371/journal.pgen.1002751 (PMC3369932; doi:10.1371/journal.pgen.1002751)
Supplement: Figure S22 — The effects of mir93 on cell proliferation. Cell proliferation was measured with the MTT assay. 200–500 cells from Control and DOX-treated groups were seeded in 96-well culture plates and were cultured in the absence (CTRL) and presence (DOX) of DOX for 7days.Data represents means SEM, n = 5. *p<0.05; Error bars represent mean ± STDEV. (PDF) [file pgen.1002751.s022.pdf]

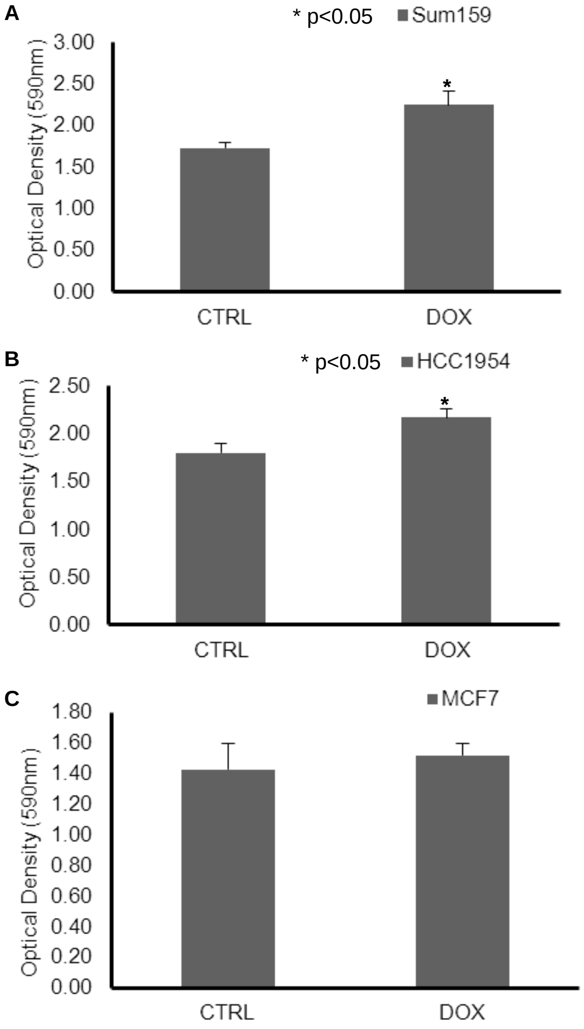

Figure S22. The effects of mir93 on cell proliferation

Cell proliferation was measured with the MTT assay. 200-500 cells from Control and DOX-treated groups were seeded in 96-well culture plates and were cultured in the absence (CTRL) and presence (DOX) of DOX for 7days.Data represents means SEM, $n = 5$. *$p<0.05$; Error bars represent mean ± STDEV.
