# Supplementary material for: MicroRNA93 Regulates Proliferation and Differentiation of Normal and Malignant Breast Stem Cells
Source: PLoS Genet. 2012 Jun 7;8(6):e1002751. doi: 10.1371/journal.pgen.1002751 (PMC3369932; doi:10.1371/journal.pgen.1002751)
Supplement: Figure S23 — Analysis of cell cycle for pTRIPZ-HCC1954-mir93 cells and pTRIPZ-MCF7-mir93cells. Cell cycle analysis of pTRIPZ-HCC1954-mir93 cells and pTRIPZ-MCF7-mir93 cells in the presence or absence of DOX. Propidium iodide staining followed by flow cytometry was used to analyze cell cycle distribution. Mir93 induced by DOX treatment resulted in a decreased proportion of cells in the G0/G1 phase and an increased proportion of cells in the S/G2/M phase for pTRIPZ-HCC1954-mir93 cells. In contrast, DOX treatment has no effects on the cell cycle for pTRIPZ-MCF7-mir93 cells. (PDF) [file pgen.1002751.s023.pdf]

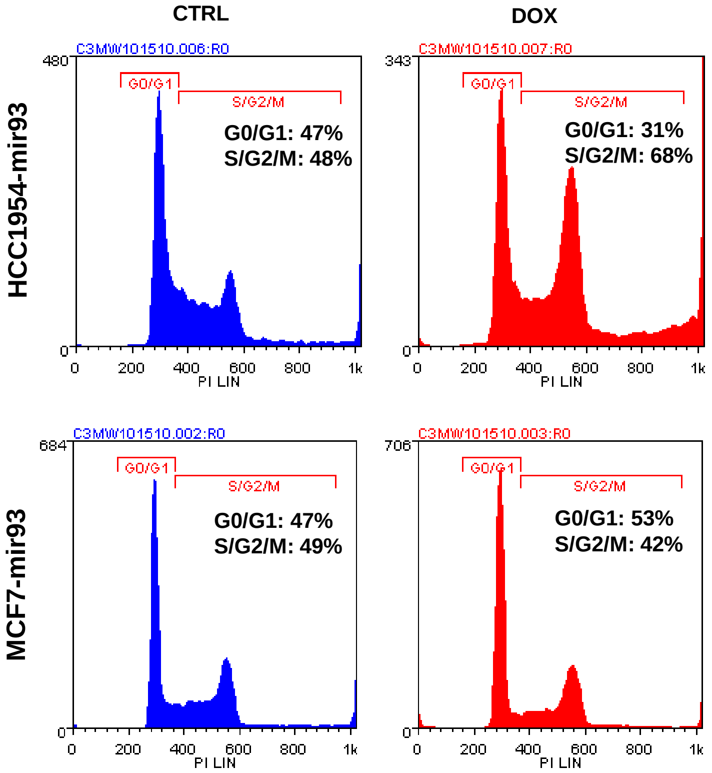

Figure S23. Analysis of cell cycle for pTRIPZ-HCC1954-mir93 cells and pTRIPZ-MCF7-mir93cells

Cell cycle analysis of pTRIPZ-HCC1954-mir93 cells and pTRIPZ-MCF7-mir93 cells in the presence or absence of DOX. Propidium iodide staining followed by flow cytometry was used to analyze cell cycle distribution. Mir93 induced by DOX treatment resulted in a decreased proportion of cells in the G0/G1 phase and an increased proportion of cells in the S/G2/M phase for pTRIPZ-HCC1954-mir93 cells. In contrast, DOX treatment has no effects on the cell cycle for pTRIPZ-MCF7-mir93 cells.
