# Supplementary material for: MicroRNA93 Regulates Proliferation and Differentiation of Normal and Malignant Breast Stem Cells
Source: PLoS Genet. 2012 Jun 7;8(6):e1002751. doi: 10.1371/journal.pgen.1002751 (PMC3369932; doi:10.1371/journal.pgen.1002751)
Supplement: Figure S24 — MCM7 and Ki67 expression is increased in ALDH- compared to ALDH+ SUM159 cells. ALDH + and − cells were separated by Aldefluor assay and expression of Ki67 and MCM7 accessed by immunofluorescence. Ki67, Red; MCM7, Green; DAPI, Blue. One representative sample from 3 independent samples is shown. (PDF) [file pgen.1002751.s024.pdf]

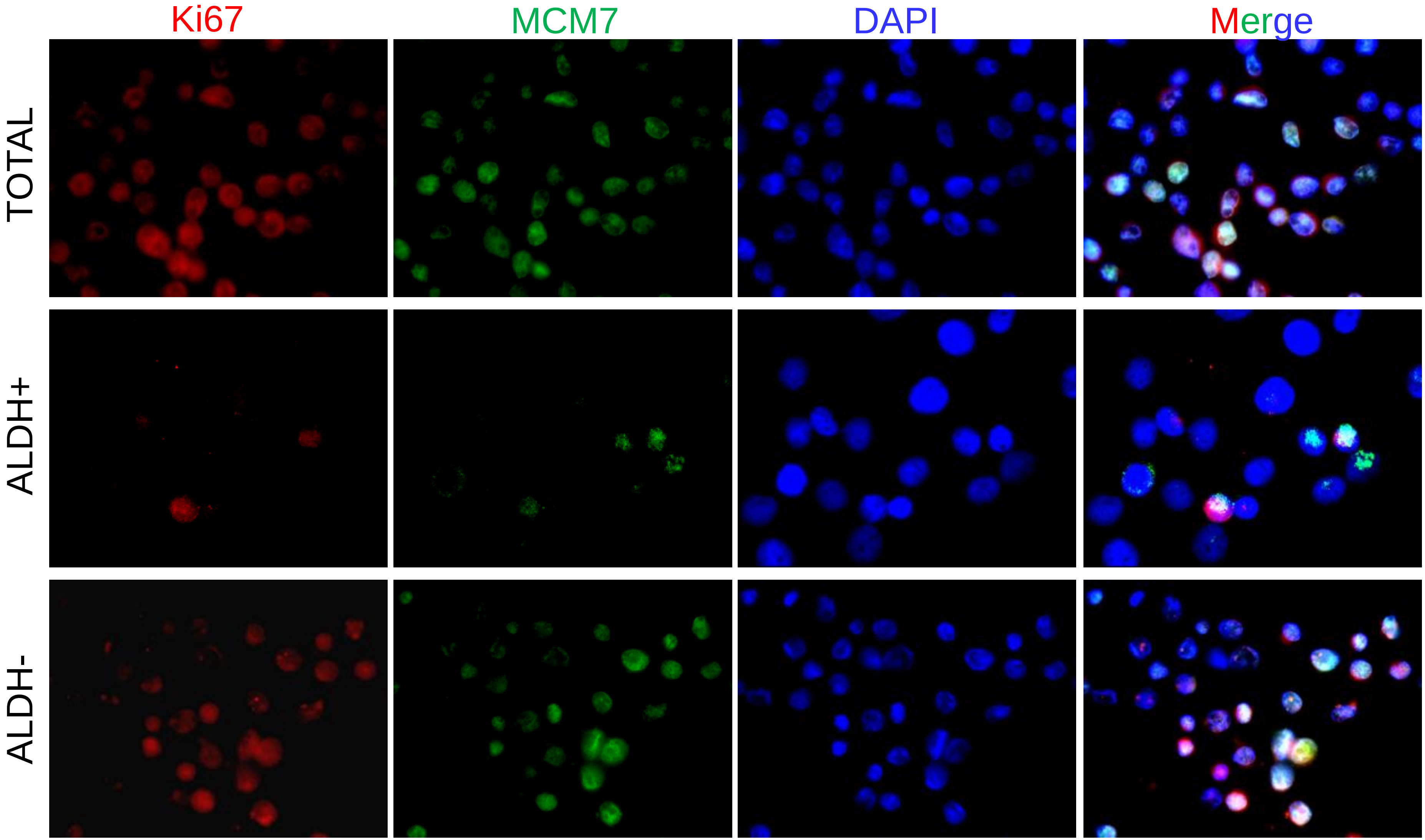

Figure S24. MCM7 and Ki67 expression is increased in ALDH- compared to ALDH+ SUM159 cells
ALDH + and – cells were separated by Aldefluor assay and expression of Ki67 and MCM7 accessed by immunofluorescence. Ki67, Red; MCM7, Green; DAPI, Blue. One representative sample from 3 independent samples is shown.
