# Supplementary material for: MicroRNA93 Regulates Proliferation and Differentiation of Normal and Malignant Breast Stem Cells
Source: PLoS Genet. 2012 Jun 7;8(6):e1002751. doi: 10.1371/journal.pgen.1002751 (PMC3369932; doi:10.1371/journal.pgen.1002751)
Supplement: Figure S25 — mir93 expression induces MET in HCC1954 cells. pTRIPZ-HCC1954-mir93 cells were plated in 2-well chamber slides with (DOX) or without (CTRL) Doxycycline for 7 days. E-Cadherin and Vimentin were stained with immunofluorescence staining. E-Cadherin, Green; Vimentin, Red; DAPI, Blue. One representative sample from 3 independent samples is shown. (PDF) [file pgen.1002751.s025.pdf]

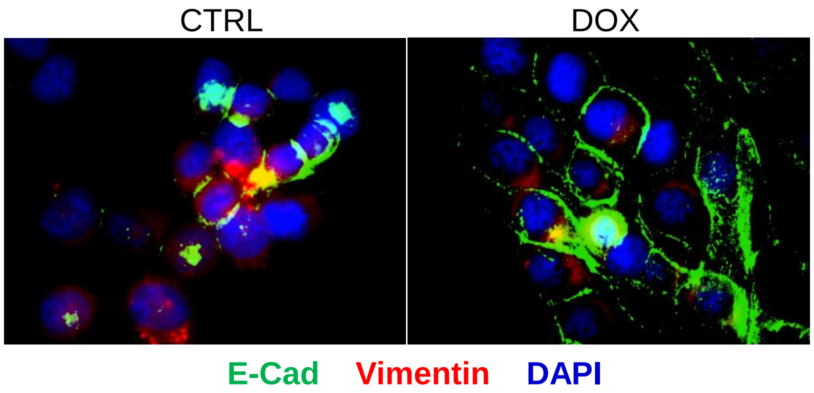


**Figure S25. Mir93 expression induces MET in HCC1954 cells**
pTRIPZ-HCC1954-mir93 cells were plated in 2-well chamber slides with (DOX) or without (CTRL) Doxycycline for 7 days. E-Cadherin and Vimentin were stained with immunofluorescence staining. E-Cadherin, Green; Vimentin, Red; DAPI, Blue. One representative sample from 3 independent samples are shown.
