# Supplementary material for: MicroRNA93 Regulates Proliferation and Differentiation of Normal and Malignant Breast Stem Cells
Source: PLoS Genet. 2012 Jun 7;8(6):e1002751. doi: 10.1371/journal.pgen.1002751 (PMC3369932; doi:10.1371/journal.pgen.1002751)
Supplement: Table S1 — Downregualted probe set in ALDH+ population from DOX vs. ALDH+ population from CTRL. (PDF) [file pgen.1002751.s026.pdf]

Downregualted probe set in ALDH+ DOX vs. CTRL

Probe	Symbol	GenBank	Gene	UniGene	Fold Change	DOX ALDH+	CTRL ALDH+
230180_at		AA521056			-3.800545149	4.040523376	7.841068525
230397_at		AI383996			-3.628527599	4.954934924	8.583462523
208003_s_at	NFAT5	NM_006599	10725	Hs.371987	-3.453179054	4.706805486	8.159984539
209136_s_at	USP10	BG390445	9100	Hs.136778	-3.450490456	7.496398092	10.94688855
207276_at	CDR1	NM_004065	1038	Hs.446675	-3.405377326	8.179748067	11.58512539
210172_at	SF1	D26121	7536	Hs.502829, Hs.708209	-3.362259025	4.734388958	8.096647983
231873_at	BMPR2	AL046696	659	Hs.471119	-3.317845656	4.685527121	8.003372777
1564378_a_at		AK025101			-3.301967938	3.906932914	7.208900852
240263_at		N74924			-3.289312999	3.223136282	6.512449281
238353_at	RASL11A	AW450584	387496	Hs.192131	-3.243042393	4.278211001	7.521253394
AFFX-HUMRGE/M10098_5_at		AFFX-HUMRGE/M10098_5			-3.239422989	7.347671939	10.58709493
207210_at	GABRA3	NM_000808	2556	Hs.123024	-3.216881477	4.684067062	7.900948539
1563947_a_at	ERC1	AK097177	23085	Hs.655744, Hs.658200	-3.214398339	4.926804296	8.141202635
215606_s_at	ERC1	AB029004	23085	Hs.655744, Hs.658200	-3.197952434	5.05872826	8.256680694
239571_at		AI123399			-3.153697805	3.38958671	6.543284515
244006_at	POU2F1	BF238986	5451	Hs.493649	-3.150757717	3.29607295	6.446830666
241621_at	SMCHD1	AW974517	23347	Hs.8118	-3.134848238	3.454555405	6.589403643
244414_at		AI148006			-3.115771068	3.958590652	7.07436172
238831_at	TMEM33	BF114679	55161	Hs.31082	-3.109304896	7.860136421	10.96944132
220342_x_at	EDEM3	NM_017992	80267	Hs.523811	-3.082835634	5.998727433	9.081563067
244181_at		AA018968			-3.07666723	4.834306461	7.910973691
235281_x_at	AHNAK	AA523289	79026	Hs.502756	-3.028139266	5.960112458	8.988251723
201299_s_at	MOBKL1B	NM_018221	55233	Hs.196437	-3.007700923	7.076772675	10.0844736
216361_s_at	MYST3	AJ251844	7994	Hs.491577	-3.006764468	5.473928961	8.480693429
243435_at	KCNQ1OT1	AI023707	10984	Hs.647285, Hs.655148	-2.988294992	4.957847818	7.94614281
241320_at		AI821449			-2.982535395	4.100170589	7.082705984
209754_s_at	TMPO	AF113682	7112	Hs.11355	-2.974942803	4.528558339	7.503501142
229403_at	B4GALT1	AI572046	2683	Hs.272011	-2.9662584	3.956256893	6.922515293
1564430_at		BC007549			2.965155303	7.895941085	4.930785782
1558747_at	SMCHD1	AA336502	23347	Hs.8118	-2.960709638	7.072827428	10.03353707
231719_at	IFRG15	NM_022347	64163	Hs.655655, Hs.716051	-2.952408014	5.189739934	8.142147947
241245_at	SFRS4	AV647470	6429	Hs.469970	-2.949788389	4.99619032	7.945978709
1559485_at	ATG2B	BC015851	55102	Hs.168241	-2.946000209	5.06653389	8.0125341
203354_s_at	PSD3	AW117368	23362	Hs.434255	-2.941252911	7.068742809	10.00999572
243455_at		AW207738			-2.939783165	5.268983097	8.208766263
213286_at	ZFR	BF445199	51663	Hs.435231	-2.93052493	7.560701396	10.49122633
235060_at	LOC10019098(	AL047052	100190986	Hs.611072, Hs.648439	-2.924751916	3.233686108	6.158438024
229694_at	BRWD2	BF062828	55717	Hs.144447	-2.92082807	4.787112424	7.707940493
221419_s_at		NM_013307			-2.907199521	7.56468149	10.47188101
206708_at	FOXN2	NM_002158	3344	Hs.468478	-2.905942439	4.747322798	7.653265237
1565886_at		W04694			-2.902452987	3.447398013	6.349851001

230036_at	SAMD9L	BE669858	219285	Hs.489118	2.899548418	9.004680577	6.105132158
216555_at	C22orf30	AK026712	253143	Hs.438906	-2.89208436	6.467566358	9.359650718
204114_at	NID2	NM_007361	22795	Hs.369840	2.857889671	8.826778953	5.968889282
241403_at	CLK4	AA468591	57396	Hs.406557	-2.855448894	3.953627255	6.809076149
1563560_at	AHNAK	M80899	79026	Hs.502756	-2.846702815	6.228329799	9.075032614
1556348_at	HEATR1	AK095692	55127	Hs.708114	-2.829347959	4.536522776	7.365870736
1568846_at		BC017718			-2.825793847	6.9335621	9.759355947
242471_at		AI916641			-2.818154383	4.104838744	6.922993127
214673_s_at	HUWE1	AU140931	10075	Hs.136905	-2.817469003	4.95390653	7.771375533
224771_at	NAV1	AI937060	89796	Hs.585374	-2.817369193	7.536676224	10.35404542
33148_at	ZFR	AI459274	51663	Hs.435231	-2.813735265	8.040686292	10.85442156
230028_at	KIAA0907	BF508843	22889	Hs.719258	-2.805656533	3.984890753	6.790547286
203056_s_at	PRDM2	AI681013	7799	Hs.371823	-2.805446695	7.906971986	10.71241868
231005_at		T91195			-2.801569509	4.188217288	6.989786796
232021_at	GLT8D3	AI864273	283464	Hs.259347	-2.79868894	3.901938602	6.700627541
221753_at	SSH1	AI651213	54434	Hs.199763	-2.795325997	6.013448535	8.808774532
206663_at	SP4	NM_003112	6671	Hs.88013	-2.793522073	3.731168029	6.524690102
231207_at		AW263539			-2.787348119	4.725525838	7.512873957
213162_at	DYNC1LI2	AI640861	1783	Hs.369068	-2.773858335	7.838511025	10.61236936
1564886_at		AL359595			-2.767544079	5.193501585	7.961045665
229943_at	TRIM13	BF939833	10206	Hs.436922	-2.757359546	4.45132393	7.208683476
239448_at		AI475033			-2.755619279	4.09421581	6.849835089
227741_at	PTPLB	AI813654	201562	Hs.705480	-2.753251738	7.637406825	10.39065856
216915_s_at	PTPN12	S69182	5782	Hs.61812	-2.74059352	5.887432522	8.628026042
215206_at		AK025143			-2.7402199	4.502282834	7.242502734
91816_f_at	MEX3D	C18318	399664	Hs.436495	-2.70654701	7.20119131	9.90773832
210943_s_at	LYST	U84744	1130	Hs.532411	-2.700099806	6.34252109	9.042620896
243088_at		W84667			-2.67868604	4.852834028	7.531520068
238362_at		AW271932			-2.673766137	5.247710052	7.921476188
241683_at	HECTD1	AW207734	25831	Hs.708017	-2.667834863	5.942451088	8.610285951
224853_at	SLAIN2	AI979301	57606	Hs.479677	-2.664561729	7.281389556	9.945951285
212142_at	MCM4	AI936566	4173	Hs.460184	-2.661856559	6.863011566	9.524868126
239147_at	ARSK	AI243677	153642	Hs.585051	-2.66094394	5.323576047	7.984519987
222145_at		AK027225			-2.660821759	4.237855672	6.898677432
244331_at		AW296971			-2.64933977	4.310294813	6.959634583
1557812_a_at		AI140531			-2.646531706	3.439620046	6.086151752
228553_at	ENAH	AI692870	55740	Hs.497893	-2.630100623	6.494486534	9.124587157
228250_at	FNIP1	AW263086	96459	Hs.591273	-2.628025487	7.150073891	9.778099378
236207_at	SSFA2	BE083088	6744	Hs.591602	-2.619212668	7.287480176	9.906692844
1569664_at		BC035915			-2.610810511	4.31112801	6.92193852
235167_at	LOC10019098	BE972419	100190986	Hs.611072, Hs.648439	-2.597718187	5.644654235	8.242372423
1563687_a_at	FRYL	U80082	285527	Hs.631525	-2.579676481	5.539031509	8.118707989
239046_at		AA322245			-2.579236796	4.176493803	6.755730599

235653_s_at	THAP6	BF685315	152815	Hs.479971	-2.575973183	4.744428973	7.320402156
235172_at		AW977516			-2.574668313	5.954266285	8.528934597
1558378_a_at	AHNAK2	BC004283	113146	Hs.441783	-2.563199313	8.642932097	11.20613141
1557227_s_at	TPR	AW235355	7175	Hs.279640	-2.56028106	8.972057155	11.53233821
242302_at	PDS5B	BF222521	23047	Hs.716441	-2.560011527	5.105605245	7.665616772
230775_s_at		BF590192			-2.557246882	5.601938149	8.159185031
1569312_at		BE383308			-2.555138846	5.626127455	8.181266301
229193_at		AA005430			-2.550396378	5.512913251	8.063309629
244753_at		BF000430			-2.547652184	4.464126148	7.011778332
1556352_at		AI692624			-2.546014248	4.790049392	7.33606364
1555294_a_at	ERC1	AB053469	23085	Hs.655744, Hs.658200	-2.541966789	4.252092402	6.794059191
214474_at	PRKAB2	NM_005399	5565	Hs.50732	-2.535407522	5.017077616	7.552485138
203319_s_at	ZNF148	L04282	7707	Hs.592591	-2.52416535	6.821116323	9.345281673
230400_s_at	PKN2	BE502469	5586	Hs.440833	-2.519740684	6.049694539	8.569435223
235709_at	GAS2L3	H37811	283431	Hs.20575	-2.518620692	6.625647189	9.144267881
235461_at	TET2	BG250414	54790	Hs.367639	-2.518531909	3.871154205	6.389686114
214375_at		AI962377			-2.517805954	4.942967179	7.460773133
230332_at	ZCCHC7	AA872187	84186	Hs.654700	-2.514486271	4.262275288	6.776761559
242068_at		AA608834			-2.513718971	4.579181271	7.092900242
239274_at		AV729557			-2.501756694	3.867366781	6.369123475
230669_at	RASA2	W38444	5922	Hs.655941	-2.501744846	5.942792566	8.444537412
204654_s_at	TFAP2A	NM_003220	7020	Hs.519880	-2.500849171	6.128110591	8.628959762
235811_at		AW590853			-2.499236463	3.946692673	6.445929136
216211_at		AL049233			-2.490446166	4.357552618	6.847998784
229858_at		AU146893			-2.480718791	5.073783264	7.554502055
1558561_at	HM13	AK074686	81502	Hs.373741	-2.479242902	4.357552618	6.836795521
235009_at	BOD1L	AI049791	259282	Hs.718459	-2.471626832	7.47584317	9.947470001
242019_at	LASS6	BG257755	253782	Hs.506829, Hs.718403	-2.469099153	5.900056864	8.369156016
229783_at		AI523593			-2.466768859	6.154198232	8.620967091
205809_s_at	WASL	BE504979	8976	Hs.143728, Hs.708402	-2.465595215	7.027286517	9.492881732
224563_at	WASF2	BG338758	10163	Hs.590909	-2.465537619	8.608612904	11.07415052
1559391_s_at		AI084451			-2.463672018	4.337589334	6.801261352
221220_s_at	SCYL2	NM_017988	55681	Hs.506481	-2.459780879	4.781028302	7.240809181
244768_at	DYNC1H1	BF447122	1778	Hs.649497	-2.457953617	5.233272228	7.691225845
236462_at		AA742310			-2.456591817	4.292994843	6.749586659
1556551_s_at	SLC39A6	BQ027619	25800	Hs.719277	-2.434393941	6.763246656	9.197640598
1558392_at	SYNE2	BQ363771	23224	Hs.525392	-2.432181558	4.531834324	6.964015882
238350_at	UBN2	AW967956	254048	Hs.153458, Hs.511486, Hs.62	-2.428680569	5.669148615	8.097829184
242918_at	NASP	AU144734	4678	Hs.319334	-2.428310725	5.543310653	7.971621378
213799_s_at	PTPRA	BF740139	5786	Hs.269577	-2.425893906	6.313559056	8.739452962
224770_s_at	NAV1	AI937060	89796	Hs.585374	-2.422580048	7.190604271	9.613184318
229935_s_at	MLL	BF057352	4297	Hs.258855	-2.422070496	5.777090787	8.199161284
241079_at		R97021			-2.419487982	4.63038948	7.049877462

212758_s_at	ZEB1	AI373166	6935	Hs.124503	-2.419298085	7.344910499	9.764208585
1568765_at	SERPINE1	BC020765	5054	Hs.414795, Hs.713079	-2.413625162	6.423626621	8.837251783
235160_at	ATF7	BG105181	11016	Hs.12286	-2.411221094	5.52136373	7.932584825
201683_x_at	TOX4	BE783632	9878	Hs.555910	-2.409278507	6.638460369	9.047738876
224854_s_at	SLAIN2	AI979301	57606	Hs.479677	-2.40897806	7.069818937	9.478796997
213816_s_at	MET	AA005141	4233	Hs.132966	-2.408468263	6.89569118	9.304159442
220797_at	METT10D	NM_024086	79066	Hs.632237	-2.405767666	5.565297659	7.971065325
1567045_at		AF085866			-2.395125282	5.299825108	7.694950391
233928_at	ADAMTS9	AB037733	56999	Hs.656071	-2.394984668	4.560194672	6.95517934
212620_at	ZNF609	AW165979	23060	Hs.595451, Hs.696256, Hs.71	-2.392850978	6.444094367	8.836945344
243303_at		AA811657			-2.3895071	5.05872826	7.448235359
241407_at		BF032023			-2.388118164	5.108395695	7.496513859
215774_s_at	SUCLG2	AV650470	8801	Hs.655250	-2.385832698	5.746619369	8.132452067
241661_at	JMJD1C	AA001021	221037	Hs.413416	-2.385643641	6.219886029	8.605529669
1553191_at	DST	NM_020388	667	Hs.631992, Hs.669931	-2.382479831	4.161960958	6.544440789
216884_at	PTPN12	S69182	5782	Hs.61812	-2.379731653	6.056765865	8.436497518
219273_at	CCNK	NM_003858	8812	Hs.510409	-2.378223732	7.573321682	9.951545414
205017_s_at	MBNL2	AI088145	10150	Hs.657347	-2.37682099	4.827520368	7.204341358
211584_s_at	NPAT	U58852	4863	Hs.171061, Hs.367437	-2.376293243	4.608150458	6.984443702
211914_x_at	NF1	M60915	4763	Hs.113577	-2.372760723	5.557886369	7.930647092
226603_at	SAMD9L	BE966604	219285	Hs.489118	2.372621519	8.707961657	6.335340138
217620_s_at	PIK3CB	AA805318	5291	Hs.239818	-2.369239082	3.669907648	6.03914673
230630_at	AK3L1	AI566130	205	Hs.10862, Hs.592601	-2.365446743	4.938237573	7.303684316
243648_at		AA280627			-2.361221001	4.612951871	6.974172872
243995_at	PTAR1	N36417	375743	Hs.719295	-2.359680049	5.413700081	7.77338013
222282_at		AV761453			-2.359047858	5.977963784	8.337011642
244357_at		T90760			-2.357848799	4.489069532	6.846918331
214786_at	MAP3K1	AA361361	4214	Hs.653654	-2.351298611	4.190899102	6.542197713
223963_s_at	IGF2BP2	AF117107	10644	Hs.35354	-2.351089586	5.876222104	8.227311689
AFFX-HUMRGE/M10098_3_at		AFFX-HUMRGE/M10098_3			-2.347640224	8.550298084	10.89793831
237895_at		AV700930			-2.345810669	5.030933492	7.376744161
229658_at	BAZ1B	BF221513	9031	Hs.719243	-2.344255503	6.337122103	8.681377606
208750_s_at	ARF1	AA580004	375	Hs.286221	-2.33904141	9.51975809	11.8587995
214127_s_at	SRRT	H28020	51593	Hs.111801	-2.322637859	6.233571531	8.55620939
1556432_at		BM987612			-2.322133535	4.290850813	6.612984348
1552680_a_at	CASC5	NM_020380	57082	Hs.181855	-2.319534247	6.756426719	9.075960966
212113_at	LOC552889	AI927479	552889	Hs.213541	-2.3171593	5.728904935	8.046064235
242038_at	LRRC8B	BG037106	23507	Hs.482017, Hs.632420	-2.316898625	4.702720125	7.01961875
204969_s_at	RDX	NM_002906	5962	Hs.263671, Hs.592679	-2.312309639	4.442549661	6.7548593
242645_at		N58278			-2.304070864	5.112919905	7.41699077
235713_at	ALKBH8	BF242537	91801	Hs.503763	-2.301123776	5.537754911	7.838878687
214537_at	HIST1H1D	NM_005320	3007	Hs.136857	-2.299483823	8.46833978	10.7678236
210932_s_at	RNF6	AF293342	6049	Hs.136885	-2.295537182	6.164943881	8.460481063

232556_at		AK021952			-2.295105296	7.041007034	9.336112329
1565887_at	TRPM7	AF086174	54822	Hs.512894	-2.293585264	4.898714429	7.192299692
228582_x_at		AI475544			-2.29260057	9.730803235	12.0234038
1554671_a_at	SRRM2	BC041155	23524	Hs.433343, Hs.719123	-2.292287832	7.973673127	10.26596096
210407_at	PPM1A	AF070670	5494	Hs.130036	-2.290768855	4.577405822	6.868174677
220825_s_at	KIRREL	NM_018240	55243	Hs.609291, Hs.657006	-2.290640544	6.069114366	8.359754909
1554986_a_at	SNX19	BC031620	399979	Hs.444024	-2.290110985	5.211815397	7.501926381
200607_s_at	RAD21	BG289967	5885	Hs.81848	-2.286070653	9.203693259	11.48976391
1558000_at	ARID5B	BU171496	84159	Hs.535297	-2.284965873	4.108792766	6.393758638
233370_at		AK024973			-2.284041413	3.842677667	6.12671908
215451_s_at	AFF1	BF575588	4299	Hs.480190	-2.283301485	6.282571132	8.565872618
244793_at		AI733237			-2.2805164	3.983403167	6.263919567
216519_s_at	PROSC	AK021923	11212	Hs.304792, Hs.608177	-2.279974567	5.643071788	7.923046355
243253_at		AA699346			-2.278261243	6.384417618	8.66267886
1553685_s_at	SP1	NM_138473	6667	Hs.620754, Hs.649191	-2.276791572	4.38161332	6.658404891
215447_at	TFPI	AL080215	7035	Hs.516578	-2.273820193	4.050128411	6.323948604
243293_at		AA765786			-2.27360731	4.489069532	6.762676842
1560705_at		AW627717			-2.273368925	4.06379958	6.337168505
218554_s_at	ASH1L	NM_018489	55870	Hs.491060	-2.267308929	5.050432209	7.317741138
213763_at	HIPK2	R37104	28996	Hs.397465, Hs.632033	-2.262379959	8.055154561	10.31753452
1555408_at		AF218570			-2.262124755	4.833440777	7.095565532
81737_at	NPIPL3	AI424872	23117	Hs.552700, Hs.611072, Hs.63	-2.260005757	4.922790085	7.182795842
215541_s_at	DIAPH1	AK023345	1729	Hs.529451	-2.259781306	5.675800338	7.935581644
235716_at		AW157450			-2.256022579	5.769086337	8.025108915
238751_at		AI343000			-2.251552371	5.536785598	7.78833797
226172_at	USP42	AU124746	84132	Hs.31856, Hs.707731	-2.249612485	5.966416534	8.216029019
227510_x_at	MALAT1	AL037917	378938	Hs.642877, Hs.714394	-2.249461091	10.16680774	12.41626883
222787_s_at	TMEM106B	AV705186	54664	Hs.396358	-2.243678903	7.623581696	9.8672606
1552611_a_at	JAK1	AL555086	3716	Hs.207538	-2.242617391	7.704464757	9.947082148
215511_at	TCF20	U19345	6942	Hs.475018	-2.2386616	4.346601342	6.585262942
243552_at	MBTD1	AW008914	54799	Hs.656803	-2.235642357	3.88448038	6.120122737
214483_s_at	ARFIP1	AF124489	27236	Hs.416089	-2.234954093	7.53710784	9.772061933
212452_x_at	MYST4	AF113514	23522	Hs.35758, Hs.599543	-2.231147619	6.422713481	8.653861101
232095_at		BG109134			-2.229692043	7.288980266	9.518672309
222755_s_at	CHD7	AI475906	55636	Hs.20395	-2.229172676	4.176134043	6.405306718
229982_at	QSER1	AW195525	79832	Hs.369368	-2.223783995	6.059613647	8.283397642
238688_at	TPM1	AI521618	7168	Hs.133892	-2.222287361	4.596605537	6.818892898
242349_at	HECTD1	AW275658	25831	Hs.708017	-2.218982013	5.245357542	7.464339555
241630_at		AA742279			-2.217489049	4.457482912	6.67497196
203899_s_at	CRCP	NM_014478	27297	Hs.300684	-2.214180875	6.864895506	9.079076381
238145_at	ZNF496	AI798611	84838	Hs.654803	-2.213950821	3.958771879	6.1727227
204180_s_at	ZBTB43	AI745225	23099	Hs.355581, Hs.718657	-2.212454214	7.038498219	9.250952432
203653_s_at	COIL	BG391060	8161	Hs.532795	-2.211868653	7.969412702	10.18128135

211088_s_at	PLK4	Z25433	10733	Hs.172052	-2.211139914	4.721875674	6.933015588
211874_s_at	MYST4	AF119230	23522	Hs.35758, Hs.599543	-2.20818169	6.273058129	8.481239819
210057_at	SMG1	U32581	23049	Hs.460179, Hs.552700	-2.206323696	7.025916005	9.232239701
212477_at	ACAP2	D26069	23527	Hs.593373	-2.205617578	6.255754012	8.461371591
230392_at		AW298141			-2.202140307	4.531834324	6.733974631
244574_at	PTPRG	AV752058	5793	Hs.595541, Hs.654488	-2.201462984	5.637826104	7.839289088
238848_at	OTUD4	BF750565	54726	Hs.270851	-2.201116792	6.393436001	8.594552793
1558214_s_at	CTNNA1	BG330076	1495	Hs.534797, Hs.656653	-2.201026919	7.41159507	9.612621989
225718_at	KIAA1715	AL133768	80856	Hs.209561	-2.196950454	6.489396918	8.686347372
238706_at	PAPD4	BG168850	167153	Hs.418198	-2.196422157	4.149234718	6.345656875
217332_at	RP11-159J2.1	AL133018	647288	Hs.567920	2.196087552	7.314631349	5.118543797
1556381_at	NARG1	AK091308	80155	Hs.715706	-2.194746304	4.069173263	6.263919567
242722_at	LMO7	AA100793	4008	Hs.207631	-2.192194573	5.640705692	7.832900265
212634_at	KIAA0776	AW298092	23376	Hs.149367	-2.192078077	7.069126923	9.261205
201766_at	ELAC2	AF304370	60528	Hs.434232	-2.189172143	5.262023582	7.451195726
224235_at		AF119853			-2.184751004	5.316985666	7.50173667
1557128_at	FAM111B	AA960844	374393	Hs.186579	-2.183813027	6.241620291	8.425433317
224828_at	CPEB4	AV704132	80315	Hs.127126	-2.182310362	7.427762543	9.610072905
203294_s_at	LMAN1	U09716	3998	Hs.465295	-2.182269158	5.174668322	7.35693748
237241_at	ECT2	AW269645	1894	Hs.518299	-2.181367954	7.712046871	9.893414826
222826_at	PLDN	BC004819	26258	Hs.719102	-2.176937084	5.672527347	7.849464432
206035_at	REL	NM_002908	5966	Hs.631886	-2.175716481	4.369868443	6.545584924
1558486_at	ZNF493	BC022394	284443	Hs.656558	-2.174775976	4.211944605	6.386720581
1557852_at		AW418842			-2.174421351	7.008691307	9.183112657
216375_s_at	ETV5	X76184	2119	Hs.43697	-2.173685698	6.709285602	8.8829713
242049_s_at	NBAS	BE783098	51594	Hs.467759	-2.170032474	6.021674879	8.191707352
216867_s_at	PDGFA	X03795	5154	Hs.535898	-2.168430423	7.781762577	9.950193
232307_at		AK021554			-2.162528115	4.357552618	6.520080733
233105_at		AK026280			-2.156976302	5.35002122	7.506997522
1559723_s_at		BC042892			-2.154547284	4.476074044	6.630621328
230340_s_at	WASL	AW173001	8976	Hs.143728, Hs.708402	-2.154377977	6.545798403	8.700176381
204182_s_at	ZBTB43	NM_014007	23099	Hs.355581, Hs.718657	-2.153931084	7.521064231	9.674995314
244396_at	G3BP1	BE673925	10146	Hs.587054	-2.15391874	7.681919171	9.835837911
1565436_s_at	MLL	AF024540	4297	Hs.258855	-2.152458156	7.268583196	9.421041352
238420_at		AV721958			-2.152240237	5.452597077	7.604837314
240105_at		AI021902			-2.149867133	5.012882734	7.162749867
208701_at	APLP2	BC000373	334	Hs.709184	-2.149610655	8.822873038	10.97248369
202549_at	VAPB	AK025720	9217	Hs.182625	-2.149363391	7.082767555	9.232130945
243361_at	SFRS12	N51597	140890	Hs.519347	-2.148804301	6.112183836	8.260988136
240787_at		AW449433			-2.145453858	4.49112033	6.636574189
225115_at	HIPK2	BF529628	28996	Hs.397465, Hs.632033	-2.145074304	7.363974993	9.509049297
240451_at		AA004844			-2.142462557	4.234590774	6.377053331
226812_at	ZFP91	AI924343	80829	Hs.524920	-2.141905514	6.877288045	9.019193559

1555677_s_at	SMC1A	BC046147	8243	Hs.211602	-2.140237321	7.446971717	9.587209037
235645_at	ESCO1	AW501507	114799	Hs.464733	-2.13647875	7.323878391	9.460357141
1558135_at	TAF11	BQ709323	6882	Hs.112444	-2.13578568	5.299825108	7.435610788
242343_x_at		H57111			-2.135230392	5.475142643	7.610373035
242983_at		AI806626			-2.132844413	5.151689475	7.284533888
1570202_a_at	MKL2	BC034934	57496	Hs.49143	-2.132409974	4.30944653	6.441856503
229216_s_at	CSNK2A1	AI090987	1457	Hs.644056	-2.13103404	8.371454813	10.50248885
239238_at		AI208857			-2.130064606	5.436894039	7.566958645
224580_at	SLC38A1	BF515894	81539	Hs.694701	-2.129843384	7.328048413	9.457891797
241659_at	JMJD1C	AA001021	221037	Hs.413416	-2.12949687	5.730876849	7.860373719
1561705_at		BC037861			-2.127588118	5.678843569	7.806431687
243496_at	RAB18	AW367507	22931	Hs.406799	-2.124918609	4.886430365	7.011348974
223038_s_at	FAM60A	BG479856	58516	Hs.505154	-2.124859233	8.500947825	10.62580706
1564039_at	ZSCAN23	AK092117	222696	Hs.376873	-2.124425066	4.162830666	6.287255732
233465_at		AA026390			-2.123290188	4.887613174	7.010903361
211540_s_at	RB1	M19701	5925	Hs.408528	-2.122952848	3.943991307	6.066944156
235803_at		AA843122			-2.121376853	4.195157949	6.316534802
1568680_s_at	YTHDC2	BC019100	64848	Hs.231942	-2.120431516	7.204542029	9.324973545
237333_at	RBBP4	T90771	5928	Hs.16003	-2.118981651	8.008226743	10.12720839
242352_at	NIPBL	AW272262	25836	Hs.481927	-2.116680181	8.537389255	10.65406944
210973_s_at	FGFR1	M63889	2260	Hs.264887	-2.11508078	6.082906406	8.197987185
209355_s_at	PPAP2B	AB000889	8613	Hs.405156, Hs.715848	-2.114272244	5.391755931	7.506028175
1562056_at		BU955063			-2.114064364	7.467475582	9.581539946
1558028_x_at	LOC647979	BI857154	647979	Hs.649310, Hs.718413	-2.114008538	6.514871092	8.62887963
214496_x_at	MYST4	NM_012330	23522	Hs.35758, Hs.599543	-2.112789844	6.404793547	8.517583391
228657_at	KIF1B	AI952999	23095	Hs.97858	-2.11264163	6.381562423	8.494204053
240262_at		AI251870			-2.111887086	4.726755112	6.838642198
232379_at	SKIL	Z19588	6498	Hs.581632	-2.111319742	4.67824429	6.789564031
231979_at		AU155091			-2.111041395	4.320503862	6.431545257
205688_at	TFAP4	NM_003223	7023	Hs.513305	-2.10821072	6.919310252	9.027520973
231064_s_at	NUP50	AW629423	10762	Hs.715672	-2.106665597	5.595478388	7.702143985
244185_at		AA921841			-2.106136608	5.196977542	7.30311415
205787_x_at	ZC3H11A	AI803216	9877	Hs.532399	-2.104924634	8.828473384	10.93339802
241702_at		AI521273			-2.103820936	5.226906686	7.330727622
238610_s_at		AI906424			-2.101011371	5.366931004	7.467942375
225730_s_at	THUMPD3	AI921788	25917	Hs.443081	-2.099182831	8.939111122	11.03829395
202173_s_at	VEZF1	NM_007146	7716	Hs.463569	-2.098659429	7.721460992	9.820120421
34478_at	RAB11B	X79780	9230	Hs.626404	-2.097384954	6.768799869	8.866184824
204323_x_at	NF1	M61213	4763	Hs.113577	-2.094675741	5.833173434	7.927849175
211077_s_at	TLK1	Z25421	9874	Hs.719163	-2.092779014	6.39675293	8.489531943
1560680_at		AL833513			-2.091488433	5.387056297	7.47854473
238558_at		AI445833			-2.084751742	6.367888469	8.452640211
212649_at	DHX29	AL079292	54505	Hs.719180	-2.081909497	7.856147203	9.9380567

211352_s_at	NCOA3	U80737	8202	Hs.592142	-2.077088107	7.205761687	9.282849793
235592_at		AW960145			-2.077070516	4.625927349	6.702997865
231866_at	LNPEP	AA767440	4012	Hs.527199, Hs.656905	-2.075544809	6.841450273	8.916995082
238349_at	UBN2	AW967956	254048	Hs.153458, Hs.511486, Hs.62	-2.075008195	4.587810946	6.662819141
214216_s_at	LARP4B	AW628686	23185	Hs.681734, Hs.713649	-2.069680993	5.074895242	7.144576234
232364_at	FBXO11	AF176706	80204	Hs.352677	-2.068399665	4.473523229	6.541922893
1556865_at		AF143885			-2.066615491	5.551981619	7.618597109
203348_s_at	ETV5	BF060791	2119	Hs.43697	-2.066136657	8.194470846	10.2606075
239002_at	ASPM	AA748494	259266	Hs.121028	-2.065201645	7.401919924	9.467121569
231106_at	BMS1P5	AI684591	399761	Hs.314437, Hs.711074	-2.062741031	5.330020301	7.392761331
234233_s_at		AK024577			-2.059311291	4.435437629	6.49474892
232275_s_at	HS6ST3	AF339824	266722	Hs.171001	-2.057920015	6.008939773	8.066859789
1559587_at	SYMPK	AL831859	8189	Hs.515475	-2.05291617	4.505651958	6.558568128
1557384_at	ZNF131	AL832081	7690	Hs.535804	-2.050502803	6.471422312	8.521925116
1557193_at		AI085450			-2.047723828	4.121314309	6.169038137
228610_at	TM9SF3	AI040432	56889	Hs.500674	-2.046807328	6.061614759	8.108422087
213606_s_at	ARHGDIA	AI571798	396	Hs.159161	-2.040983012	7.112700317	9.153683328
244464_at		AA668789			-2.040143089	3.997301338	6.037444428
201615_x_at	CALD1	AI685060	800	Hs.490203	-2.039999279	9.619032826	11.65903211
233401_at		BF723605			-2.039946708	4.228746801	6.268693508
231768_at	USF1	X55666	7391	Hs.414880	-2.0379552	5.647172771	7.685127971
230057_at	LOC285178	AI609832	285178	Hs.586683	-2.037240598	7.555895549	9.593136147
219437_s_at	ANKRD11	NM_013275	29123	Hs.335003	-2.035831319	8.866850397	10.90268172
229834_at	NFIX	AI937201	4784	Hs.257970	-2.034342208	5.880548436	7.914890644
1554873_at	CSPP1	BC029445	79848	Hs.370147	-2.031082529	5.508437263	7.539519792
214352_s_at	KRAS	BF673699	3845	Hs.505033	-2.029321852	8.148121683	10.17744353
203628_at	IGF1R	H05812	3480	Hs.643120, Hs.653608, Hs.71	-2.028089699	6.300984803	8.329074501
237158_s_at	MPHOSPH9	AW449069	10198	Hs.577404	-2.027391007	6.26591234	8.293303347
223510_at	NRP2	AF280545	8828	Hs.471200	-2.026895794	5.699703163	7.726598957
241611_s_at	FNDC3A	BE675600	22862	Hs.508010	-2.026428178	5.331663727	7.358091904
228180_at		AA805653			-2.026377547	5.256064174	7.282441721
221098_x_at	UTP14A	NM_006649	10813	Hs.458598	-2.024417587	5.798492291	7.822909878
243839_s_at		AW297257			-2.021703514	4.350673126	6.37237664
1558220_at		BM914560			-2.019901192	4.69566336	6.715564552
242092_at	EPB41L2	AA019300	2037	Hs.486470	-2.015842478	4.130807194	6.146649672
211681_s_at	PDLIM5	AF116705	10611	Hs.480311	-2.014803536	6.037481134	8.05228467
1554277_s_at	FANCM	BC036056	57697	Hs.509229	-2.014727746	5.349414048	7.364141794
236841_at	WASH3P	BE464132	374666	Hs.459573, Hs.585931	-2.013697497	5.556203199	7.569900696
204047_s_at	PHACTR2	AW295193	9749	Hs.102471	-2.009093935	6.827273008	8.836366943
235575_at		AA682539			-2.009052843	5.088100244	7.097153087
224282_s_at	AGPAT3	AB040138	56894	Hs.248785	-2.006790645	6.124466308	8.131256953
214008_at	TWF1	N25562	5756	Hs.189075	-2.005497439	5.990751759	7.996249198
232028_at	ZNF678	AK026475	339500	Hs.30323	-2.005321403	5.254613662	7.259935065

1556656_at		BF477401			-2.00505903	4.11645306	6.12151209
236595_at		AA776458			-2.002957876	5.412360905	7.415318781
242859_at		BE156563			-2.000850096	5.971809099	7.972659195
238951_at		BF843343			-2.000440144	5.238682196	7.239122341
200841_s_at	EPRS	AI142677	2058	Hs.497788	-1.998270134	8.419588423	10.41785856
236957_at	CDCA2	AI248208	157313	Hs.33366	-1.997885698	7.203197273	9.201082971
208425_s_at	TANC2	NM_015623	26115	Hs.410889	-1.997193983	6.118195906	8.115389889
237184_at		BF110186			-1.996942731	4.879656572	6.876599303
243589_at	KIAA1267	AI823453	284058	Hs.648744	-1.9960773	5.644438168	7.640515468
220154_at	DST	NM_020388	667	Hs.631992, Hs.669931	-1.995867329	7.972578973	9.968446302
200702_s_at	DDX24	BG421209	57062	Hs.510328	-1.992161289	9.745344605	11.73750589
1558449_at		BC040475			-1.991446495	4.182863829	6.174310324
238468_at	TNRC6B	AA214704	23112	Hs.372082	-1.990164193	7.213653356	9.20381755
244026_at		BF063657			-1.986595949	4.032843122	6.01943907
244356_at		AL079909			-1.98614886	6.133274709	8.119423569
205461_at	RAB35	NM_006861	11021	Hs.524788, Hs.719173	-1.984906755	6.073411496	8.058318252
225233_at	MSI2	T71491	124540	Hs.658922	-1.984390809	6.194549113	8.178939921
243670_at		BE670161			-1.983686406	5.011076363	6.994762769
230099_at		AI139993			-1.982946771	4.661550678	6.644497449
220410_s_at	CAMSAP1	NM_018627	157922	Hs.522493	-1.982856191	5.130579375	7.113435566
239512_at	SFRS4	R05895	6429	Hs.469970	-1.982312961	6.06943664	8.0517496
234168_at	TAF15	AK000942	8148	Hs.402752	-1.977259414	5.518021552	7.495280966
205426_s_at	HIP1	U79734	3092	Hs.329266, Hs.619089	-1.974510846	6.212971522	8.187482368
227931_at	INO80D	AI823917	54891	Hs.445036	-1.972990771	8.245246684	10.21823745
232778_at		AK026036			-1.971228967	5.830597247	7.801826213
1558015_s_at	ACTR2	BU175810	10097	Hs.643727, Hs.719274	-1.969612086	7.595699584	9.56531167
212404_s_at	UBE3B	AL096740	89910	Hs.374067	-1.966671149	4.603771654	6.570442804
223818_s_at	RSF1	AF059317	51773	Hs.420229	-1.963054472	7.444242061	9.407296533
214718_at	GATAD1	AK026142	57798	Hs.21145	-1.962396664	4.558500391	6.520897055
1555372_at	BCL2L11	AF455755	10018	Hs.469658	-1.961583002	4.175268979	6.136851981
1570651_at	CCBL1	BC022468	883	Hs.495250	-1.961162841	4.418766659	6.3799295
240248_at		AA778783			-1.961144198	5.325334908	7.286479106
229574_at	TRA2A	AI268231	29896	Hs.445652	-1.957351547	5.337442842	7.29479439
212105_s_at	DHX9	BF313832	1660	Hs.191518	-1.955573446	8.889226024	10.84479947
206929_s_at	NFIC	NM_005597	4782	Hs.170131	-1.952901335	9.108878857	11.06178019
236023_at	CDK9	AI703465	1025	Hs.150423, Hs.706809	-1.951455998	5.371441712	7.32289771
237118_at		AA057437			-1.9494911	4.375471476	6.324962576
232711_at		AU146197			-1.948905614	5.713314354	7.662219968
207520_at	TROVE2	BG494940	6738	Hs.288178	-1.948292438	5.309223074	7.257515512
230216_at	C12orf51	BF434969	283450	Hs.695995	-1.947571608	4.531834324	6.479405931
236700_at	EIF3C	AI377875	8663	Hs.567374	-1.94749657	5.59692859	7.54442516
1559369_at	C5orf44	BI668018	80006	Hs.591760	-1.943138776	4.221261967	6.164400744
202412_s_at	USP1	AW499935	7398	Hs.35086	-1.940941603	8.594950254	10.53589186

1569477_at		BC025999			-1.940517042	5.419260078	7.359777121
236752_at		AA913146			-1.939528661	4.26528432	6.204812981
233674_at		AK026286			-1.938878469	5.980071196	7.918949665
1569106_s_at	SETD5	BI087313	55209	Hs.288164	-1.935857893	5.066566971	7.002424865
223888_s_at	LARS	AF151026	51520	Hs.432674	-1.935777587	9.718600798	11.65437838
1570425_s_at	LATS1	AF119846	9113	Hs.716697	-1.935567265	4.623796524	6.55936379
229115_at	DYNC1H1	BF000332	1778	Hs.649497	-1.935202572	8.382779123	10.31798169
205097_at	SLC26A2	AI025519	1836	Hs.302738	-1.934843573	7.497993486	9.432837059
213446_s_at	IQGAP1	AI679073	8826	Hs.430551	-1.934162684	9.092157928	11.02632061
233323_at		AK024973			-1.932147677	4.651273545	6.583421222
242886_at		AW007763			-1.930997461	5.859006645	7.790004107
206269_at	GCM1	AB026493	8521	Hs.28346	1.928457963	6.558289543	4.62983158
222371_at		AI732802			-1.925165396	4.604719674	6.52988507
215780_s_at		Z95126			-1.923906125	8.794364923	10.71827105
241155_at		AA704588			-1.922847495	4.772481098	6.695328594
241762_at	FBXO32	BF244402	114907	Hs.403933	-1.922208504	4.53317919	6.455387694
1569607_s_at		BC016022			-1.921418456	5.15732166	7.078740116
208719_s_at	DDX17	U59321	10521	Hs.528305, Hs.665429, Hs.71	-1.921200016	5.911399081	7.832599097
229895_s_at	NCK1	AI377384	4690	Hs.477693	-1.921144971	6.144227355	8.065372326
239957_at		AW510793			-1.91978254	4.399604943	6.319387483
206652_at	ZMYM5	NM_016384	9205	Hs.530988	-1.917934338	6.370141997	8.288076335
238619_at		AA417078			-1.917907841	6.416643186	8.334551027
241820_at	RIF1	BF666241	55183	Hs.655671	-1.917904345	6.171972855	8.0898772
235588_at	ESCO2	AA740849	157570	Hs.99480	-1.917721627	7.982759267	9.900480894
235059_at	RAB12	BF574430	201475	Hs.270074	-1.916647017	7.820289286	9.736936303
239596_at	SLC30A7	AA521381	148867	Hs.533903	-1.914210943	5.103146137	7.01735708
1561640_at		BC008637			-1.911784491	5.26063402	7.172418511
201868_s_at	TBL1X	AI082187	6907	Hs.495656	-1.910031047	8.618883152	10.5289142
227961_at	CTSB	AA130998	1508	Hs.520898	-1.909796301	7.575543555	9.485339856
235216_at	ESCO1	BG532121	114799	Hs.464733	-1.909058926	8.558619228	10.46767815
242617_at	TMED8	AI290654	283578	Hs.200413, Hs.26403	-1.907673101	7.61926337	9.526936471
227924_at	INO80D	BF571256	54891	Hs.445036	-1.906053981	4.849318857	6.755372837
208151_x_at	DDX17	NM_030881	10521	Hs.528305, Hs.665429, Hs.71	-1.905256916	6.314689354	8.21994627
235547_at	N4BP2L2	BG548427	10443	Hs.507680	-1.903506902	6.735693461	8.639200363
203491_s_at	CEP57	AI123527	9702	Hs.101014	-1.89911262	9.141290253	11.04040287
238795_at	C10orf18	AA424537	54906	Hs.699500	-1.898625681	5.461381647	7.360007328
213998_s_at	DDX17	AW188131	10521	Hs.528305, Hs.665429, Hs.71	-1.898582983	8.282378982	10.18096196
200917_s_at	SRPR	BG474541	6734	Hs.368376	-1.898214225	6.85027245	8.748486676
214007_s_at	TWF1	AW665024	5756	Hs.189075	-1.895198751	6.907745321	8.802944072
209203_s_at	BICD2	BC002327	23299	Hs.436939	-1.894816336	7.644155686	9.538972022
1569538_at		AF130084			-1.892059635	4.988736393	6.880796029
228563_at	GJC1	BE504215	10052	Hs.532593, Hs.712052	-1.890739615	6.562544775	8.45328439
1559881_s_at	ZNF12	BM463827	7559	Hs.431471	-1.890599224	6.291151218	8.181750442

239234_at		AW452419			-1.889779838	6.326115007	8.215894845
227659_at	PLEKHA3	BE550332	65977	Hs.41086	-1.888035358	6.39073542	8.278770778
244826_at		R24061			-1.886506486	5.431097947	7.317604434
238774_at		AW960454			-1.885667351	5.342918306	7.228585657
232882_at		AA079839			-1.882579518	5.634813199	7.517392717
215079_at		AK026723			-1.879730678	5.285483365	7.165214044
202378_s_at	LEPROT	NM_017526	54741	Hs.705413	-1.879035615	9.310381652	11.18941727
243074_at		N48990			-1.879027886	4.426879197	6.305907083
235680_at		AI914925			-1.878701616	4.966543914	6.845245529
234932_s_at	CDCP1	AK026028	64866	Hs.476093	-1.878568457	8.58501337	10.46358183
213926_s_at	AGFG1	AI742626	3267	Hs.352962	-1.87708336	5.179355671	7.056439031
225571_at	LIFR	AA701657	3977	Hs.133421	-1.876499196	7.838626834	9.715126029
225801_at	FBXO32	AW518714	114907	Hs.403933	-1.876414122	5.495766132	7.372180253
227602_at	RAB7A	BE858244	7879	Hs.15738, Hs.716392	-1.876162093	10.43226457	12.30842667
228803_at		AI809749			-1.87522815	4.904140714	6.779368864
240438_at		AI215674			-1.875011652	5.170438173	7.045449825
213494_s_at	YY1	AA748649	7528	Hs.388927	-1.875010619	9.227247398	11.10225802
216275_at	BUB1	AK023540	699	Hs.469649	-1.873251026	7.224392689	9.097643716
1555039_a_at	ABCC4	AY133679	10257	Hs.508423	-1.873098526	6.429982955	8.303081481
231920_s_at	CSNK1G1	AK025179	53944	Hs.646508	-1.873008006	6.217053641	8.090061648
230173_at	TRIM4	BE503987	89122	Hs.50749	-1.872609712	5.606578487	7.479188199
217208_s_at	DLG1	AL121981	1739	Hs.292549	-1.868492233	6.116700096	7.985192329
220466_at	CCDC15	NM_025004	80071	Hs.287555	-1.867897891	6.685194939	8.55309283
1558410_s_at		AW974642			-1.863165869	5.111972583	6.975138452
214888_at	CAPN2	AK023851	824	Hs.350899	-1.86308018	4.343745793	6.206825972
219843_at	IPP	NM_005897	3652	Hs.699548	-1.859946091	6.122799987	7.982746078
222873_s_at	EHMT1	AI739378	79813	Hs.495511	-1.858643549	5.600208631	7.45885218
241499_at	ZNF621	AI672426	285268	Hs.19977	-1.857228414	5.395903909	7.253132323
213957_s_at	CEP350	AA635523	9857	Hs.413045	-1.855788377	7.159403747	9.015192124
1563009_at	LOC284930	BC039485	284930	Hs.407567	-1.855067621	5.279818129	7.13488575
228712_at		AI924616			-1.853844891	7.115337834	8.969182725
1562208_a_at		AL137309			-1.853474159	4.692636816	6.546110975
209997_x_at	PCM1	BC000453	5108	Hs.491148	-1.852610186	7.289270676	9.141880862
242422_at	G3BP1	AA180161	10146	Hs.587054	-1.850679904	8.354711273	10.20539118
1552621_at		BQ613856			-1.849127428	6.693542978	8.542670406
214753_at	N4BP2L2	AW084068	10443	Hs.507680	-1.848411704	7.094775953	8.943187656
216629_at	SRRM2	AC004493	23524	Hs.433343, Hs.719123	-1.847957799	4.68745218	6.535409979
241832_at	FAM98A	AI864271	25940	Hs.468140	-1.846900623	4.606060813	6.452961437
1554984_a_at	HLA-DOB	BC020226	3112	Hs.1802	-1.846461997	4.952632179	6.799094177
235705_at		BF676361			-1.843540175	7.329852533	9.173392708
228237_at	PAPPA2	N30053	60676	Hs.187284	-1.842113364	5.718593829	7.560707193
1557953_at	ZKSCAN1	BG761185	7586	Hs.615360	-1.841883807	8.453741757	10.29562556
205580_s_at	HRH1	D28481	3269	Hs.1570	-1.839821807	7.328048413	9.16787022

205188_s_at	SMAD5	NM_005903	4090	Hs.167700	-1.839710297	7.575707987	9.415418284
208503_s_at	GATAD1	NM_021167	57798	Hs.21145	-1.83888296	7.090851702	8.929734662
1557100_s_at	HECTD1	AL038005	25831	Hs.708017	-1.838775911	7.089461241	8.928237152
1567044_s_at		AF085866			-1.836897711	4.906775809	6.743673521
236668_at		AW205474			1.835840282	7.264617078	5.428776796
1556834_at		BC042986			-1.83278175	7.158396744	8.991178494
241063_at		BE672556			-1.831262074	5.35938929	7.190651364
201167_x_at	ARHGDIA	D13989	396	Hs.159161	-1.831035723	6.889577492	8.720613215
1554311_a_at	SUPT6H	BC033074	6830	Hs.250429	-1.830273866	4.622696028	6.452969894
215561_s_at	IL1R1	AK026803	3554	Hs.701982	-1.830242663	7.276110926	9.106353589
225377_at	C9orf86	BE783949	55684	Hs.370555	-1.827787929	7.732063394	9.559851323
242762_s_at	FAM171B	AA372349	165215	Hs.28872	-1.827715468	5.623481864	7.451197332
1558807_at	ATAD2B	AK094821	54454	Hs.467862	-1.826527582	5.025521797	6.852049379
220609_at	LOC202181	NM_024651	202181	Hs.189914, Hs.653052	-1.825223167	4.344962258	6.170185425
207057_at	SLC16A7	NM_004731	9194	Hs.439643	-1.822017243	6.996516047	8.818533291
227744_s_at	HNRNPD	AW005670	3184	Hs.480073	-1.821779918	9.45514509	11.27692501
218006_s_at	ZNF22	NM_006963	7570	Hs.462693, Hs.655124	-1.81968722	6.637728469	8.457415689
209807_s_at	NFIX	U18759	4784	Hs.257970	-1.815762915	8.119265957	9.935028872
233309_at		AU145723			-1.815753179	4.532072489	6.347825668
241391_at		AA654772			-1.81530639	4.450368217	6.265674607
1563130_a_at		AL109710			-1.814742135	4.905594755	6.72033689
239718_at		R42552			-1.813665362	5.09738949	6.911054852
225097_at	HIPK2	BF594155	28996	Hs.397465, Hs.632033	-1.81237817	9.950953154	11.76333132
243236_at		AA807545			-1.810271829	4.400719573	6.210991401
215041_s_at	DOCK9	BE259050	23348	Hs.596105	-1.810008571	5.164886205	6.974894775
1564494_s_at	P4HB	AK075503	5034	Hs.464336	-1.808683276	7.7486398	9.557323077
235744_at	PPTC7	BG252924	160760	Hs.13854	-1.808450435	4.774100341	6.582550776
238564_at	FAM171B	BE326579	165215	Hs.28872	-1.804162741	5.711718816	7.515881557
1554260_a_at	FRYL	BC021803	285527	Hs.631525	-1.803791397	7.167888765	8.971680162
242125_at		BG280919			-1.802874885	5.554787036	7.357661921
223940_x_at	MALAT1	AF132202	378938	Hs.642877, Hs.714394	-1.80231828	11.09845474	12.90077301
1557543_at		AL832672			-1.801922137	4.546077126	6.347999263
233303_at		AL110175			-1.800745389	6.304361028	8.105106417
242774_at	SYNE2	AI684761	23224	Hs.525392	-1.800682158	4.504077137	6.304759295
217659_at		AA457019			-1.800565631	4.368357593	6.168923224
216048_s_at	RHOBTB3	AK023621	22836	Hs.445030	-1.798064286	6.078610722	7.876675008
1558093_s_at	MATR3	BI832461	9782	Hs.268939	-1.797594469	10.16818966	11.96578413
212468_at	SPAG9	AK023512	9043	Hs.463439	-1.79711466	8.564005822	10.36112048
222404_x_at	PTPLAD1	AI984229	51495	Hs.512973	-1.796737262	9.62918506	11.42592232
242647_at	USP34	AI148382	9736	Hs.644708	-1.795747493	5.035638749	6.831386241
227383_at		AW340595			-1.79542139	5.952862314	7.748283704
236899_at		BF445387			-1.795073197	4.392389032	6.187462229
239077_at	CSGALNACT2	W81648	55454	Hs.657569	-1.795018259	6.117030878	7.912049137

201728_s_at	KIAA0100	AA904674	9703	Hs.151761	-1.794864406	9.32008559	11.11495
213298_at	NFIC	X12492	4782	Hs.170131	-1.794772061	8.792389253	10.58716131
223217_s_at	NFKBIZ	BE646573	64332	Hs.319171	-1.794127838	6.960069199	8.754197037
230590_at		BE675486			-1.792014213	5.232473536	7.024487749
236966_at		BF942281			-1.791338705	5.204611301	6.995950007
215757_at	PRKDC	AK022387	5591	Hs.491682	-1.790611772	6.461925344	8.252537116
214215_s_at	LARP4B	AW514174	23185	Hs.681734, Hs.713649	-1.790076447	4.73891489	6.528991338
227454_at	TAOK1	AB037782	57551	Hs.631758	-1.788556818	8.985090396	10.77364721
225937_at		BF002121			-1.787839947	7.71953373	9.507373677
225173_at	ARHGAP18	BE501862	93663	Hs.486458, Hs.708586	-1.784548559	9.583330385	11.36787894
232333_at		AU147805			-1.783859403	5.025528765	6.809388168
229808_at	CHAF1A	AI344306	10036	Hs.79018	-1.783560499	6.321995979	8.105556478
235927_at	XPO1	BE350122	7514	Hs.370770	-1.782319291	7.047221644	8.829540935
211094_s_at	NF1	D12625	4763	Hs.113577	-1.781711486	5.606422417	7.388133903
1559954_s_at	DDX42	AF147429	11325	Hs.702010	-1.780984013	7.32188257	9.102866583
1552829_at		NM_018505			-1.780431589	5.030933492	6.81136508
240239_at	ZNF566	N63953	84924	Hs.533939	-1.779790183	5.183610448	6.963400631
231956_at	KIAA1618	AA976354	57714	Hs.514554	-1.77955133	4.299945147	6.079496477
1557129_a_at	FAM111B	AA960844	374393	Hs.186579	-1.778200551	8.025981222	9.804181773
242665_at	FMNL2	AL042120	114793	Hs.654630	-1.777009399	7.622150975	9.399160374
212392_s_at	PDE4DIP	AI950145	9659	Hs.584841, Hs.613082, Hs.65	-1.775027021	7.400673637	9.175700658
222439_s_at	THRAP3	BE967048	9967	Hs.160211	-1.774901886	9.656183119	11.43108501
241955_at	HECTD1	BE243270	25831	Hs.708017	-1.77443282	9.388418085	11.1628509
242751_at		N55072			-1.771590659	4.8964863	6.668076959
201072_s_at	SMARCC1	AW152160	6599	Hs.476179	-1.770628294	9.584979502	11.3556078
1554029_a_at	TTC37	BC030966	9652	Hs.482868	-1.769030182	6.097313953	7.866344135
1569022_a_at	PIK3C2A	BC040952	5286	Hs.175343	-1.769024789	6.44214811	8.211172899
244341_at		AA827728			-1.768774649	4.603837825	6.372612474
221350_at	HOXC8	NM_022658	3224	Hs.664500	-1.76773289	6.417230499	8.184963388
1558527_at	LOC10013270	AK056609	100132707	Hs.586358	-1.767298037	4.883736572	6.651034609
1558275_at		BC006120			-1.766468252	5.19754763	6.964015882
239197_s_at	EZH1	BG470312	2145	Hs.194669	-1.766139429	6.973220007	8.739359436
236188_s_at	NAP1L4	AI381524	4676	Hs.501684, Hs.695395	-1.7658537	4.271590728	6.037444428
224227_s_at	BDP1	AF298152	55814	Hs.258272	-1.765415523	8.11791235	9.883327873
212079_s_at	MLL	AA715041	4297	Hs.258855	-1.765349339	6.828576721	8.59392606
224963_at	SLC26A2	AK025078	1836	Hs.302738	-1.763907523	7.766078764	9.529986287
224775_at	IWS1	AW451291	55677	Hs.469879	-1.763141196	9.227811166	10.99095236
236665_at	CCDC18	BF056459	343099	Hs.716682	-1.760309698	7.579142855	9.339452553
213756_s_at	HSF1	AI393937	3297	Hs.530227	-1.759296889	5.227576223	6.986873112
207177_at	PTGFR	NM_000959	5737	Hs.654365	-1.757396186	7.528565927	9.285962113
1557388_at	RTTN	BC013774	25914	Hs.654809	-1.756809208	6.441542063	8.198351272
212220_at	PSME4	AI972268	23198	Hs.413801	-1.755588557	7.861153479	9.616742035
229179_at	RUFY1	BE677830	80230	Hs.306769	-1.755497337	6.321390607	8.076887944

242163_at	THRAP3	AW082726	9967	Hs.160211	-1.755371463	5.297648367	7.05301983
1552622_s_at		BQ613856			-1.752659693	6.496122776	8.248782469
1569385_s_at	TET2	BC019007	54790	Hs.367639	-1.752481933	5.864567769	7.617049703
211548_s_at	HPGD	J05594	3248	Hs.596913	1.748878631	8.558018784	6.809140154
229643_at	ITGA6	AI857933	3655	Hs.133397	-1.748649855	4.787773743	6.536423597
235030_at	FAM55C	BF969254	91775	Hs.595933	-1.748327847	6.37479356	8.123121407
215250_at	TMEM111	AU147317	55831	Hs.475392	-1.746571646	5.617020283	7.363591929
1316_at	THRA	X55005	7067	Hs.724	-1.746314117	6.624591384	8.370905502
222834_s_at	GNG12	N32508	55970	Hs.431101	-1.74627058	10.53489141	12.28116199
218700_s_at	RAB7L1	BC002585	8934	Hs.115325	-1.741168004	7.482156677	9.223324681
213472_at	HNRNPH1	AI022387	3187	Hs.604001	-1.740570965	5.053468367	6.794039332
233728_at		AU148213			-1.739896399	4.509828308	6.249724708
242126_at		T53962			-1.738954949	4.87980346	6.618758409
224687_at	ANKIB1	AB037807	54467	Hs.83293	-1.736209902	8.153323344	9.889533246
211947_s_at	BAT2D1	AI359472	23215	Hs.494614	-1.736029062	8.718387896	10.45441696
66053_at	HNRNPUL2	AI738452	221092	Hs.714969	-1.735605943	7.590709304	9.326315246
212226_s_at	PPAP2B	AA628586	8613	Hs.405156, Hs.715848	-1.734417589	6.743780265	8.478197854
202601_s_at	HTATSF1	AI373539	27336	Hs.204475	-1.733846739	7.012464353	8.746311092
214985_at	EXT1	AF070571	2131	Hs.492618	-1.732906628	4.877267814	6.610174443
228304_at	RBM43	BE674118	375287	Hs.302442	-1.730071192	6.054860762	7.784931954
238961_s_at	FNDC3A	AA452239	22862	Hs.508010	-1.729250449	6.681273181	8.41052363
216235_s_at	EDNRA	S81545	1909	Hs.183713	-1.729138861	6.713169682	8.442308543
242492_at	CLNS1A	AA412065	1207	Hs.430733, Hs.598033	-1.728351963	6.134377702	7.862729664
220409_at	CAMSAP1	NM_018627	157922	Hs.522493	-1.727076483	5.418804198	7.145880681
1565928_at		AW517148			-1.726876909	5.195181433	6.922058343
205121_at	SGCB	NM_000232	6443	Hs.438953	-1.725191675	6.242006712	7.967198387
239689_at		BF063236			1.72446177	6.456260161	4.731798391
216868_s_at	LOC10012965(	D13413	100129656		-1.72339478	4.69202306	6.415417841
239597_at		AA993566			-1.72260655	4.755813714	6.478420264
203917_at	CXADR	NM_001338	1525	Hs.634837	-1.722549964	7.623069283	9.345619247
203318_s_at	ZNF148	NM_021964	7707	Hs.592591	-1.721964133	8.124005018	9.845969151
241214_at		AI939471			-1.720583523	6.608819891	8.329403414
234000_s_at	PTPLAD1	AJ271091	51495	Hs.512973	-1.719827837	8.479884027	10.19971186
1557581_x_at		BC027347			-1.719760085	4.534550553	6.254310638
239978_at		BF109370			-1.719534334	4.746958782	6.466493117
223799_at	KIAA1826	AF253976	84437	Hs.266782	-1.719187173	6.529692778	8.24887995
217189_s_at	SMG7	AL137800	9887	Hs.591463	-1.717917981	5.552668044	7.270586025
238807_at		AW973964			-1.715407206	5.421027367	7.136434573
241769_at		AW962458			-1.715083674	4.596023267	6.311106941
224852_at	TTC17	BE964325	55761	Hs.696109	-1.714833928	7.497876511	9.212710439
1553677_a_at	TIPRL	NM_152902	261726	Hs.209431	-1.712861352	6.679514207	8.392375559
201846_s_at	RYBP	NM_012234	23429	Hs.7910	-1.712712846	7.81505546	9.527768305
226589_at	TMEM192	BE964222	201931	Hs.708090	-1.710273305	5.533113882	7.243387187

244799_s_at		BE348796			-1.709098046	4.360434083	6.069532129
207700_s_at	NCOA3	NM_006534	8202	Hs.592142	-1.708872674	8.46833978	10.17721245
229527_s_at	OSBPL11	AA074624	114885	Hs.477440	-1.70676932	6.048208616	7.754977936
214464_at	CDC42BPA	NM_003607	8476	Hs.35433	-1.70676059	9.548355337	11.25511593
1554021_a_at	ZNF12	AF505656	7559	Hs.431471	-1.706188924	8.583636612	10.28982554
238519_at	RSC1A1	BG179828	6248	Hs.145049, Hs.239459, Hs.70	-1.702945368	7.628240457	9.331185824
223900_s_at	PBRM1	AF225870	55193	Hs.189920	-1.702320931	5.834355293	7.536676224
224568_x_at	MALAT1	AW005982	378938	Hs.642877, Hs.714394	-1.702078894	11.21025908	12.91233797
212093_s_at	MTUS1	AI695017	57509	Hs.7946	-1.701143132	4.612421843	6.313564975
235425_at	SGOL2	AW965339	151246	Hs.655182	-1.699172792	8.907090711	10.6062635
228484_s_at	FOXO1	AI472322	2308	Hs.370666	-1.698724944	6.27555627	7.974281214
211116_at	SLC9A2	AF073299	6549	Hs.250083	-1.698628056	5.556198249	7.254826306
223793_at	BAT2L	BC002872	84726	Hs.495349, Hs.703868	-1.698310391	4.840717419	6.539027811
229728_at		AI691075			-1.697684223	4.828857264	6.526541487
244292_at		AL119491			-1.697182772	4.99619032	6.693373092
203914_x_at	HPGD	NM_000860	3248	Hs.596913	1.696796416	8.255909379	6.559112963
233376_at		AF288406			-1.695935634	5.274907463	6.970843098
234939_s_at	PHF12	AL161953	57649	Hs.714975	-1.694252376	5.223736288	6.917988664
215987_at	RAPGEF2	AV654984	9693	Hs.113912	-1.694233872	5.152766312	6.847000184
238595_at		AV702101			-1.6941921	5.691515568	7.385707669
1567224_at	HMGA2	U29113	8091	Hs.505924	-1.69356198	4.537621394	6.231183374
214313_s_at	EIF5B	BE138647	9669	Hs.158688	1.693412814	9.248021359	7.554608545
244473_at		R37637			-1.6917251	5.433935154	7.125660254
1567032_s_at	ZNF160	X78928	90338	Hs.655967	-1.691692963	4.831214742	6.522907705
231862_at	CBX5	AK023520	23468	Hs.349283	-1.691685214	7.610011888	9.301697103
220369_at	SMEK1	NM_017936	55671	Hs.533887	-1.691578661	6.49382098	8.185399641
1555608_at	CAPRIN2	AF326778	65981	Hs.234355	-1.691193699	6.145095104	7.836288804
227315_x_at	PROSC	AA758214	11212	Hs.304792, Hs.608177	-1.690969265	6.769056993	8.460026258
241597_at		W22152			-1.690146847	5.559227744	7.249374591
212605_s_at		AK025759			-1.689903098	6.879741228	8.569644325
227349_at	HELLS	AI807356	3070	Hs.655830	-1.689640675	7.030893253	8.720533928
1553304_at	LSM14B	NM_144703	149986	Hs.105379	-1.686945608	5.758095431	7.445041038
222909_s_at	BAG4	AF111116	9530	Hs.194726	-1.683324616	6.984376111	8.667700727
236417_at		BE644770			-1.681507923	6.850524093	8.532032015
211000_s_at	IL6ST	AB015706	3572	Hs.532082	-1.681342269	9.339499358	11.02084163
242320_at		AI435586			-1.680786739	7.568984646	9.249771384
222634_s_at	TBL1XR1	AF314544	79718	Hs.715537	-1.680608873	8.168416062	9.849024935
237095_at	ASXL2	AW628946	55252	Hs.594386	-1.68049266	5.884543211	7.565035871
1554901_at	GAFA1	AF190748	100131227	Hs.713290	-1.679405665	4.863891568	6.543297234
236649_at	DTWD1	AA907927	56986	Hs.127432	-1.678389266	5.754961432	7.433350697
1555436_a_at	AFF4	BC025700	27125	Hs.519313, Hs.664840	-1.676980187	7.658988308	9.335968495
236557_at	ZBTB38	AW085625	253461	Hs.715534	-1.676307714	7.647555851	9.323863565
238883_at		AW975051			-1.675205373	5.403270237	7.07847561

1554595_at	SYMPK	BC030214	8189	Hs.515475	-1.673709	5.688348034	7.362057034
1570108_at		BC037848			-1.673552768	5.532138924	7.205691691
201942_s_at	CPD	D85390	1362	Hs.446079	-1.673381257	8.233706151	9.907087408
214139_at	ARID4B	AI051476	51742	Hs.533633, Hs.575782	-1.673089282	7.864048742	9.537138024
229753_at	POU2F1	BF511210	5451	Hs.493649	-1.672272859	6.532574401	8.20484726
201793_x_at	SMG7	BF509099	9887	Hs.591463	-1.672156319	8.967045878	10.6392022
201901_s_at	YY1	Z14077	7528	Hs.388927	-1.671108084	9.982888524	11.65399661
211973_at		AW341200			-1.669694049	5.799156728	7.468850777
210109_at	C7orf54	AF191492	27099	Hs.657377	-1.669299611	5.26944434	6.938743951
227537_s_at	SP3	AW173024	6670	Hs.531587	-1.668444896	6.381270939	8.049715834
236368_at	KIAA0368	BF059292	23392	Hs.368255	-1.668128083	6.307920608	7.976048691
213457_at	MFHAS1	BF739959	9258	Hs.379414	-1.667287008	7.522360832	9.18964784
205917_at	ZNF264	NM_003417	9422	Hs.515634	-1.667143872	7.15575115	8.822895022
201730_s_at	TPR	BF110993	7175	Hs.279640	-1.66466437	9.658729125	11.32339349
229042_s_at	DYNC1H1	N26619	1778	Hs.649497	-1.664481834	7.645257349	9.309739182
AFFX-HUMRGE/M10098_M_at		AFFX-HUMRGE/M10098_M			-1.662890643	11.81781669	13.48070734
203085_s_at	TGFB1	BC000125	7040	Hs.645227	-1.662759273	7.348384661	9.011143933
212629_s_at	PKN2	AI633689	5586	Hs.440833	-1.662721048	9.14793951	10.81066056
1559728_at	ZBTB40	BF355863	9923	Hs.418966	-1.660725933	5.979246802	7.639972735
209305_s_at	GADD45B	AF078077	4616	Hs.110571	-1.660705319	10.42671323	12.08741855
230735_at		AI653318			-1.660574432	5.357017597	7.017592029
229302_at	TMEM178	AA058832	130733	Hs.40808	1.660400787	6.562213126	4.901812339
222933_at	MORN1	NM_024848	79906	Hs.642701	-1.659608731	5.323539257	6.983147988
222810_s_at	RASAL2	BF435513	9462	Hs.549729, Hs.715627	-1.656381883	7.667798999	9.324180882
226319_s_at	THOC4	AF047002	10189	Hs.534385	-1.655913146	9.471149661	11.12706281
1559883_s_at	SAMHD1	AF147427	25939	Hs.580681	-1.655061861	4.526886547	6.181948408
210457_x_at	HMGA1	AF176039	3159	Hs.518805, Hs.703764	-1.65451885	7.67006723	9.32458608
1562442_a_at	SSBP1	BC008402	6742	Hs.490394	-1.654246413	5.113075442	6.767321855
216176_at	HCRP1	AK025343	387535	Hs.675399	-1.654246413	5.68832979	7.342576203
1555594_a_at	MBNL1	AF401998	4154	Hs.478000, Hs.558914	-1.651893351	7.309374274	8.961267625
210625_s_at	AKAP1	U34074	8165	Hs.463506	-1.649657105	6.382922463	8.032579568
217602_at	PPIA	AI191118	5478	Hs.356331, Hs.598115	-1.649574702	5.781312481	7.430887183
229966_at	EWSR1	AW089574	2130	Hs.374477	-1.64943029	6.636602036	8.286032326
238449_at	LOC595101	BG534511	595101	Hs.654650, Hs.655267	-1.648808835	5.505572855	7.15438169
1554178_a_at	FAM126B	BC039295	285172	Hs.24701	-1.648652919	7.704607493	9.353260412
226920_at	CSNK1A1	AW592437	1452	Hs.529862, Hs.712555	-1.647646061	9.645792853	11.29343891
228901_at	CDK9	AI040910	1025	Hs.150423, Hs.706809	-1.64696732	5.9514487	7.598416019
227513_s_at	LRRFIP1	AW027170	9208	Hs.471779	-1.64449165	7.600300784	9.244792433
235057_at	ITCH	AW089307	83737	Hs.632272	-1.644348029	8.264815335	9.909163364
206636_at	RASA2	NM_006506	5922	Hs.655941	-1.6428094	6.504326885	8.147136285
242137_at		H10545			-1.641945739	4.895923623	6.537869361
211110_s_at	AR	AF162704	367	Hs.496240	-1.641020509	7.148035328	8.789055837
1555117_at		BC008618			-1.6409236	4.574670737	6.215594337

222527_s_at	RBM22	AL538762	55696	Hs.713564	-1.64084565	8.603910079	10.24475573
213697_at	HIPK3	AW291829	10114	Hs.201918	-1.63952554	4.727191806	6.366717347
215992_s_at	RAPGEF2	AL117397	9693	Hs.113912	-1.63750516	5.805392734	7.442897894
213517_at	PCBP2	AW103422	5094	Hs.546271	-1.637385571	7.198837599	8.836223169
214445_at	ELL2	NM_012081	22936	Hs.192221, Hs.708710	-1.637122076	7.819649005	9.456771081
242805_at		AW081636			-1.636650735	5.373261475	7.00991221
236932_s_at	GATAD2A	AW058619	54815	Hs.709287	-1.635374208	4.758075125	6.393449333
217489_s_at	IL6R	S72848	3570	Hs.709210	-1.634433631	4.690661468	6.325095099
232323_s_at	TTC17	AK026217	55761	Hs.696109	-1.632606122	8.666149625	10.29875575
227152_at	C12orf35	AI979334	55196	Hs.445129	-1.632580603	9.285201558	10.91778216
207143_at	CDK6	NM_001259	1021	Hs.119882	-1.632060028	6.037060849	7.669120877
241301_at		AA694187			-1.630929621	5.391871385	7.022801006
207186_s_at	BPTF	NM_004459	2186	Hs.444200	-1.630740066	9.423844688	11.05458475
244774_at	PHACTR2	R81072	9749	Hs.102471	-1.630298175	6.828837274	8.459135449
219571_s_at	ZNF12	NM_016265	7559	Hs.431471	-1.627505808	8.213053665	9.840559472
205079_s_at	MPDZ	NM_003829	8777	Hs.169378	-1.626876177	7.12667222	8.753548397
222600_s_at	UBA6	AB014773	55236	Hs.719086	-1.626783728	7.542967312	9.16975104
1555526_a_at	6-Sep	AF403061	23157	Hs.496666	-1.626611477	5.403334375	7.029945852
214577_at	MAP1B	BG164365	4131	Hs.335079	-1.625266402	7.285527433	8.910793835
235341_at	DNAJC3	AL119957	5611	Hs.59214	-1.624473506	5.845692112	7.470165618
232940_s_at	MLL3	AK025911	58508	Hs.647120	-1.624450775	7.980460804	9.604911578
202247_s_at	MTA1	BE561596	9112	Hs.525629	-1.623564819	7.002171838	8.625736657
202975_s_at	RHOBTB3	N21138	22836	Hs.445030	-1.623175867	8.465466537	10.0886424
232238_at	ASPM	AK001380	259266	Hs.121028	-1.623072287	7.507901967	9.130974255
1567223_at	HMGA2	U29113	8091	Hs.505924	-1.62267937	5.182790474	6.805469845
1565347_s_at	TFE3	AY034078	7030	Hs.274184	-1.621671306	4.755921595	6.377592901
241797_at		AI904095			-1.621429698	4.574383036	6.195812734
201679_at	SRRT	BE646076	51593	Hs.111801	-1.619897383	8.271682467	9.891579849
238329_at	MPRIP	BE545235	23164	Hs.462341	-1.618657481	6.843152853	8.461810334
242476_at		AI436356			-1.612871262	5.35114462	6.964015882
221639_x_at		AF068846			-1.611460303	7.482366276	9.093826579
205967_at	HIST1H4C	NM_003542	8364	Hs.46423	-1.611442618	8.809007729	10.42045035
1553122_s_at	RBAK	NM_021163	57786	Hs.396178	-1.610646453	6.496098174	8.106744628
240395_at	LOC10012872	AI635761	100128727	Hs.636145, Hs.713106	-1.610401134	4.881217313	6.491618447
239486_at		BG111636			-1.610264958	5.396884721	7.007149679
1553105_s_at	DSG2	NM_001943	1829	Hs.412597	-1.608514925	7.609984949	9.218499874
241755_at	UQCRC2	AI961429	7385	Hs.528803	-1.608271325	4.624563832	6.232835157
1554159_a_at	ZMYND11	BC034784	10771	Hs.292265	-1.607812053	8.215045865	9.822857918
239742_at	TULP4	H15278	56995	Hs.486993	-1.607759105	6.500061922	8.107821027
1558001_s_at	ARID5B	BU171496	84159	Hs.535297	-1.60670119	4.551637111	6.158338301
235189_at	NARG2	BE780502	79664	Hs.200943	-1.602876182	6.308097509	7.910973691
210635_s_at	KLHL20	BC005253	27252	Hs.495035	-1.60216524	4.493388767	6.095554007
213015_at	BBX	BF448315	56987	Hs.124366	-1.602161728	9.462658463	11.06482019

1557987_at	LOC641298	BC042832	641298	Hs.552700	-1.601318649	4.915891938	6.517210587
242477_at	TTC39B	BF056282	158219	Hs.563630	1.600680978	6.701665635	5.100984656
227740_at	UHMK1	AW173222	127933	Hs.127310	-1.60064079	10.1205902	11.72123099
220038_at		NM_013257			-1.600540955	6.156377839	7.756918794
210892_s_at	GTF2I	BC004472	2969	Hs.647041	-1.600061641	7.133020309	8.73308195
1562387_at	C5orf42	BU189724	65250	Hs.170993, Hs.586199, Hs.64	-1.599912648	4.789704125	6.389616774
215545_at		AK024185			-1.599676632	4.817915632	6.417592264
239370_at		AW081982			1.599660556	8.335038209	6.735377653
228367_at	ALPK2	BE551416	115701	Hs.628152	1.599403378	8.669735648	7.07033227
221188_s_at	CIDEB	NM_014430	27141	Hs.642693, Hs.657595, Hs.70	-1.596257678	4.918477754	6.514735432
237262_at		AI912190			-1.596216854	6.263919567	7.860136421
231152_at	INO80D	AW452971	54891	Hs.445036	-1.594687848	6.156422228	7.751110076
227175_at		AI806486			-1.592752878	5.382111449	6.974864327
208859_s_at	ATRX	AI650257	546	Hs.533526, Hs.645562, Hs.65	-1.592139429	9.315934957	10.90807439
212279_at	TMEM97	BE779865	27346	Hs.199695	-1.591997096	7.466583978	9.058581074
1560348_at	RGNEF	AK094713	64283	Hs.482521	-1.590798544	6.701867142	8.292665687
230543_at	USP9X	AI761675	8239	Hs.77578	-1.590269073	4.700612472	6.290881544
217373_x_at	MDM2	AJ276888	4193	Hs.484551	-1.589779717	5.190523479	6.780303196
222387_s_at	VPS35	BG476669	55737	Hs.454528	-1.587604106	8.216353881	9.803957986
236355_s_at		AI076172			-1.587357344	5.415452543	7.002809887
223254_s_at	G2E3	AA887053	55632	Hs.509008, Hs.605081	-1.587272799	9.520899633	11.10817243
214972_at	MGEA5	AU144791	10724	Hs.500842	-1.586802623	5.58388227	7.170684894
241425_at	NUPL1	AA769986	9818	Hs.310453, Hs.719291	-1.585073156	7.115567626	8.700640781
204999_s_at	ATF5	BC005174	22809	Hs.9754	-1.582576102	8.273081643	9.855657745
214701_s_at	FN1	AJ276395	2335	Hs.203717	-1.582474653	6.733005822	8.315480475
219158_s_at	NARG1	NM_025085	80155	Hs.715706	-1.582025998	9.573793828	11.15581983
228623_at		AI224133			-1.581324716	7.477893068	9.059217784
210529_s_at	FAM115A	BC000609	9747	Hs.718446	-1.580710293	7.37159598	8.952306273
219024_at	PLEKHA1	NM_021622	59338	Hs.643512	-1.579953003	7.450291051	9.030244054
221745_at	DCAF7	BE538424	10238	Hs.410596	-1.579222282	5.896040303	7.475262586
229896_at	GTF2I	H41907	2969	Hs.647041	-1.578988542	7.429939297	9.00892784
207213_s_at	USP2	NM_004205	9099	Hs.524085	-1.578923914	4.683593894	6.262517808
208042_at	AGGF1	NM_013303	55109	Hs.634849	-1.576890963	6.517080521	8.093971484
1555409_a_at		AF218570			-1.576777183	5.790880398	7.367657581
244606_at	ATP1A1	AI264247	476	Hs.371889	-1.574368485	4.965226728	6.539595212
1565701_at		AL832624			-1.573880825	5.171356236	6.745237061
237761_at	NEK6	AI479332	10783	Hs.197071	-1.573851983	5.540971876	7.114823859
214545_s_at	PROSC	NM_007198	11212	Hs.304792, Hs.608177	-1.572436025	7.010223321	8.582659346
239022_at		AW090199			-1.571954002	6.397202807	7.969156809
217608_at	SFRS12IP1	AW408767	285672	Hs.69504	-1.569478728	8.945444932	10.51492366
212106_at	FAF2	BF116183	23197	Hs.484242	-1.569384623	8.946040969	10.51542559
230607_at		T86874			-1.567437437	5.725161733	7.29259917
AFFX-HSAC07/X00351_5_at	ACTB	AFFX-HSAC07/X00351_5	60	Hs.520640, Hs.708120	-1.566512012	10.9898621	12.55637411

202971_s_at	DYRK2	NM_006482	8445	Hs.173135	-1.565932523	5.822644851	7.388577374
234874_at	ATE1	AF079098	11101	Hs.632080	-1.565602834	5.45710473	7.022707564
240078_at	SFRS8	BG434474	6433	Hs.308171	-1.564846062	5.21614975	6.780995812
233800_at		AA805082			-1.564726775	4.574048047	6.138774822
231403_at	TRIO	N21108	7204	Hs.130031	-1.563899427	7.80234217	9.366241597
226061_s_at	NUDT3	BE674245	11165	Hs.188882	-1.563617778	8.34039654	9.904014318
220292_at	ZNF434	NM_024340	54925	Hs.592078	-1.563472966	6.419597817	7.983070783
224730_at	DCAF7	AW575465	10238	Hs.410596	-1.563217963	6.864106909	8.427324872
227484_at	SRGAP1	BF508615	57522	Hs.210751, Hs.593803	-1.562976303	8.987298309	10.55027461
1557246_at	KIDINS220	AA992480	57498	Hs.9873	-1.562186746	6.037931206	7.600117952
214808_at		AU147851			-1.561582181	7.513745685	9.075327866
231247_s_at	LOC727820	AI310647	727820	Hs.547271	-1.561457454	7.747604693	9.309062147
229703_at	DLG1	AA279428	1739	Hs.292549	-1.559583412	6.494668168	8.05425158
229272_at	FNBP4	AI083506	23360	Hs.6834	-1.558587947	4.642606175	6.201194122
202818_s_at		AI344128			-1.558537684	9.947407726	11.50594541
230629_s_at	EP400	AI809582	57634	Hs.699245	-1.55837865	7.602412725	9.160791375
234159_at		AK025182			-1.556077161	5.732004746	7.288081907
241798_at		AI339930			-1.556027857	4.481774872	6.037802729
1560476_at		AF290476			-1.555866026	4.58815252	6.144018546
1562110_at		BC022892			-1.554978188	4.751578317	6.306556505
231904_at	U2AF1	AU122448	7307	Hs.365116	-1.554743827	7.676909874	9.231653701
1556687_a_at	CLDN10	BE465772	9071	Hs.534377, Hs.656580	-1.553335382	4.506535971	6.059871353
1559038_at	2-Sep	BC043180	4735	Hs.335057, Hs.716403	-1.551648533	6.451527299	8.003175832
220843_s_at	DCAF13	NM_014156	25879	Hs.532265	-1.550644891	5.128023102	6.678667994
240139_at		AI949690			-1.550363518	5.496668164	7.047031681
233827_s_at	SUPT16H	AK024072	11198	Hs.213724	-1.549329491	10.12125899	11.67058848
231169_at		N29877			-1.548445781	8.551428279	10.09987406
229541_at		BE669703			-1.548143523	5.59849541	7.146638933
224943_at	BTBD7	AI580162	55727	Hs.525549	-1.547238533	6.734806445	8.282044978
235003_at	UHMK1	AI249980	127933	Hs.127310	-1.547110613	9.546532588	11.0936432
1556051_a_at	BICD1	CA777994	636	Hs.505202	-1.545969894	6.758483477	8.30445337
1560026_at		BC037944			-1.545815307	4.954246615	6.500061922
243874_at	LPP	AI079544	4026	Hs.444362	-1.545518969	5.841949633	7.387468602
228995_at	CAMSAP1	BF434985	157922	Hs.522493	-1.545152598	5.239568917	6.784721515
208178_x_at	TRIO	NM_007118	7204	Hs.130031	-1.544522364	7.782357194	9.326879558
216509_x_at	MLLT10	AF060938	8028	Hs.30385	-1.544402384	6.133298664	7.677701048
211089_s_at	NEK3	Z25434	4752	Hs.409989	-1.542397499	5.182151471	6.724548971
200920_s_at	BTG1	AL535380	694	Hs.255935	-1.541322291	10.80293113	12.34425342
1559614_at	FLJ38773	AK096092	284808		-1.540897402	4.772173957	6.313071359
223997_at	FNIP1	BC001956	96459	Hs.591273	-1.539284239	6.232713456	7.771997694
209052_s_at	WHSC1	BF111870	7468	Hs.113876	-1.538766316	6.22139469	7.760161005
214295_at	KIAA0485	AW129056	57235	Hs.604754	-1.538296205	5.904947793	7.443243998
215886_x_at	USP12	AF022790	219333	Hs.42400	-1.5378038	5.479306194	7.017109994

239232_at	MSI2	AA521410	124540	Hs.658922	-1.536766816	4.887998857	6.424765673
1553322_s_at	TEAD1	NM_021961	7003	Hs.655331	-1.536499164	5.615454713	7.151953877
243318_at	DCAF8	AI208342	50717	Hs.632447	-1.536468634	7.487032644	9.023501278
211832_s_at	MDM2	AF201370	4193	Hs.484551	-1.536194922	5.073586184	6.609781106
227631_at	ABI2	BF058849	10152	Hs.471156	-1.536014451	5.251966718	6.787981169
220085_at	HELLS	NM_018063	3070	Hs.655830	-1.535923463	8.711426786	10.24735025
233566_at	LOC84856	AK024638	84856	Hs.55977	-1.53448797	4.843023478	6.377511448
236496_at	DEGS2	AW006352	123099	Hs.159643	1.531864925	6.211209146	4.679344221
222087_at	PVT1	AW451806	5820	Hs.133107, Hs.675281	-1.531750014	6.763945407	8.295695421
243474_at		W87425			-1.531356477	4.665908159	6.197264637
1552656_s_at	UHMK1	NM_144624	127933	Hs.127310	-1.528918891	4.713153357	6.242072248
202723_s_at	FOXO1	AW117498	2308	Hs.370666	-1.528537637	9.10178472	10.63032236
235409_at	MGA	AU149225	23269	Hs.187569	-1.528136486	7.797906871	9.326043357
202323_s_at	ACBD3	AI636775	64746	Hs.520207	-1.52691938	5.935423501	7.462342881
236836_at		BE503070			-1.526329386	5.646653133	7.172982519
208297_s_at	EVI5	NM_005665	7813	Hs.594434	-1.526308932	6.85661164	8.382920571
1552536_at	VTI1A	NM_145206	143187	Hs.194554	-1.52627152	6.309467082	7.835738601
224829_at	CPEB4	AA772278	80315	Hs.127126	-1.526268621	6.209661062	7.735929684
210866_s_at	CNOT4	AF180475	4850	Hs.490224	-1.526145049	5.752403015	7.278548064
1554574_a_at	CYB5R3	AF361370	1727	Hs.700572	-1.525671699	7.640723462	9.166395161
205854_at	TULP3	AK024246	7289	Hs.655333	-1.525006127	7.662266518	9.187272645
229274_at	GNAS	AI693143	2778	Hs.125898, Hs.694849	-1.523014757	5.166007683	6.68902244
236561_at	TGFBR1	AV700621	7046	Hs.494622	-1.522340018	4.950899183	6.473239201
1554213_at	ARHGEF10	BC036809	9639	Hs.98594	-1.522040791	5.669148615	7.191189406
206521_s_at	GTF2A1	NM_015859	2957	Hs.592334, Hs.593630	-1.521671421	7.647555851	9.169227272
239901_at		BF642798			-1.520138913	4.847300895	6.367439808
1557029_at		BC035392			-1.520025662	7.767170598	9.287196259
238611_at		AI906424			-1.5185577	6.034125649	7.55268335
210436_at	CCT8	BC005220	10694	Hs.125113	-1.518314887	4.825768618	6.344083506
214447_at	ETS1	NM_005238	2113	Hs.369438	-1.517432016	6.574048145	8.09148016
244677_at		AA416756			-1.517033252	6.900854066	8.417887318
232569_at		AK024255			-1.515397534	6.632141727	8.147539261
1558775_s_at	NSMAF	AU142380	8439	Hs.372000	-1.51512519	5.782679512	7.297804702
1566001_at		AK096064			-1.515053466	4.560597413	6.075650879
228079_at	C3orf58	AI343351	205428	Hs.288954	1.514109777	6.171380507	4.65727073
231592_at		AV646335			-1.5137031	7.922317886	9.436020986
230628_at	EP400	AI809582	57634	Hs.699245	-1.513210896	5.868891626	7.382102522
244383_at		AI283051			-1.513051453	5.730815198	7.243866651
242223_at		AA505323			-1.512677791	4.869660233	6.382338024
206767_at	RBMS3	NM_014483	27303	Hs.696468	-1.512123705	6.176872731	7.688996436
225957_at	C5orf41	AI307750	153222	Hs.484195	-1.510848196	7.613738961	9.124587157
232264_at		AK022204			-1.510767573	7.093365975	8.604133548
210743_s_at	CDC14A	AF064103	8556	Hs.127411	-1.510288937	4.72797043	6.238259367

230742_at	RBM6	AA742596	10180	Hs.439480	-1.510243792	6.557136759	8.067380552
213755_s_at	SKI	BF431501	6497	Hs.663133	-1.508263216	8.512464761	10.02072798
233560_x_at	MCM8	AA370141	84515	Hs.631506	-1.507878603	5.740177419	7.248056023
222667_s_at	ASH1L	AI806500	55870	Hs.491060	-1.507318913	8.075282085	9.582600998
224676_at	TMED4	AI472339	222068	Hs.598832, Hs.715273	-1.506790967	8.638678251	10.14546922
213668_s_at	SOX4	AI989477	6659	Hs.643910	-1.506170635	6.681957202	8.188127837
1555515_a_at	FAM189B	BC008854	10712	Hs.348308	-1.505632935	4.617835909	6.123468844
242514_at		R16900			-1.505144741	4.633150786	6.138295527
229191_at	TBCD	AL096745	6904	Hs.464391	1.505002463	6.177311472	4.672309009
1569525_s_at		BC040855			-1.504975634	5.726738445	7.231714079
241625_at	LOC389834	BE221330	389834	Hs.595418	-1.504951527	6.953976646	8.458928173
227918_s_at	ZYG11B	N21008	79699	Hs.476280	-1.504909271	4.885750924	6.390660196
239339_at		AW820262			-1.501364398	5.421628729	6.922993127
238938_at		AI674059			-1.501225647	4.649620293	6.15084594
230503_at		AA151917			-1.499797518	5.85875957	7.358557088
1560926_at		AF085924			-1.499374782	6.999710641	8.499085423
204073_s_at	C11orf9	NM_013279	745	Hs.473109	1.499349357	8.132948576	6.633599219
211949_s_at	NOLC1	AI355279	9221	Hs.523238	-1.499302141	8.492454364	9.991756505
212451_at	SECISBP2L	N52532	9728	Hs.9997	-1.499177002	6.812856977	8.312033979
234811_at	CENPN	AK026313	55839	Hs.55028	-1.499079187	5.169567791	6.668646978
220617_s_at	ZNF532	NM_018181	55205	Hs.529023, Hs.607676	-1.499028434	9.946981952	11.44601039
1560492_at		BC014174			-1.498968232	6.199060457	7.698028689
244766_at		BG180003			-1.498579053	4.873797587	6.37237664
221705_s_at	SIKE1	BC005934	80143	Hs.709277	-1.498236359	9.479720081	10.97795644
204426_at	TMED2	NM_006815	10959	Hs.592682, Hs.75914	-1.497917979	8.555913177	10.05383116
225100_at	FBXO45	BF590021	200933	Hs.169815	-1.497273556	7.465509391	8.962782947
241965_at		BF589232			-1.497231006	6.060926151	7.558157157
238800_s_at	ZCCHC6	AA776496	79670	Hs.655162	-1.496570129	7.43615132	8.932721449
243593_s_at		BF003018			-1.496101193	5.279357434	6.775458626
235757_at		AA814006			-1.495800758	5.300057947	6.795858705
238013_at	PLEKHA2	BF347859	59339	Hs.369123	-1.494354813	4.898469108	6.392823921
235730_at		AA830545			-1.493939486	4.677146868	6.171086354
232465_at		AK021749			-1.492805579	4.953514725	6.446320304
209655_s_at	TMEM47	AI803181	83604	Hs.8769	-1.492770827	8.428834917	9.921605744
244546_at	CYCS	AI760495	54205	Hs.437060, Hs.617193	-1.492218273	4.574613374	6.066831647
211016_x_at	HSPA4	BC002526	3308	Hs.90093	-1.492079116	9.165324983	10.6574041
1561884_at	CEPT1	AL833102	10390	Hs.363572, Hs.636850	-1.491941238	5.606797219	7.098738457
204315_s_at	GTSE1	AI340239	51512	Hs.386189	-1.491849917	8.553699239	10.04554916
216627_s_at	B4GALT1	U10473	2683	Hs.272011	-1.491607466	4.802099016	6.293706482
201340_s_at	ENC1	AF010314	8507	Hs.104925	-1.491600394	5.668309542	7.159909936
208879_x_at	PRPF6	BG469030	24148	Hs.31334	-1.491495338	7.424424484	8.915919822
1567015_at	NFE2L2	AF323119	4780	Hs.715540	-1.490292001	8.598734954	10.08902695
211965_at	ZFP36L1	BE620915	677	Hs.707091, Hs.85155	-1.490050038	9.212626696	10.70267673

201164_s_at	PUM1	BG474429	9698	Hs.281707	-1.490018917	10.26249955	11.75251846
217966_s_at	FAM129A	NM_022083	116496	Hs.518662	-1.488960581	7.394804939	8.88376552
1560752_at	FBXW2	AK025767	26190	Hs.494985	-1.488804267	4.577108825	6.065913091
215547_at	TSC22D2	AF201291	9819	Hs.665220, Hs.715600	-1.488086803	5.289072584	6.777159387
1569302_at	KIAA1731	BC017394	85459	Hs.458418	-1.487880628	7.164420604	8.652301232
1553185_at	RASEF	NM_152573	158158	Hs.657750	-1.487083101	9.568184423	11.05526752
217626_at		BF508244			-1.486118899	8.639771663	10.12589056
204270_at	SKI	AI568728	6497	Hs.663133	-1.485737255	9.77021325	11.2559505
1570264_at		BC017949			-1.48511548	4.695076956	6.180192436
224754_at	SP1	BG431266	6667	Hs.620754, Hs.649191	-1.485105785	8.932065042	10.41717083
229171_at	MGC16385	AI761337	92806	Hs.513832	-1.483723712	4.531834324	6.015558036
71933_at	WNT6	AI218134	7475	Hs.29764	1.483640303	6.560279395	5.076639092
202821_s_at	LPP	AL044018	4026	Hs.444362	-1.48288668	8.223855207	9.706741887
233349_at	TLK2	AI800481	11011	Hs.445078	-1.482203599	5.789401252	7.271604851
226587_at	SNRPN	BE783065	6638	Hs.555970, Hs.564847, Hs.57	-1.481091972	4.544297825	6.025389797
220729_at		NM_014092			-1.481060984	6.417260536	7.898321519
213548_s_at	CDV3	BG257762	55573	Hs.518265	-1.480630637	8.302086541	9.782717178
214805_at	EIF4A1	U79273	1973	Hs.129673	-1.479988424	6.500061922	7.980050346
219496_at	ANKRD57	NM_023016	65124	Hs.355455	-1.479341403	8.365262873	9.844604276
1568853_at		BC038201			1.479237444	9.08174552	7.602508076
1566809_a_at		AK097593			-1.478923564	5.035404324	6.514327888
241595_at		BF223007			-1.478252645	5.864806644	7.343059289
243558_at		AI040122			-1.476734822	4.863202131	6.339936953
204463_s_at	EDNRA	AU118882	1909	Hs.183713	-1.475201751	5.703621889	7.17882364
208591_s_at	PDE3B	NM_000922	5140	Hs.445711	-1.475099748	4.978744481	6.453844229
239227_at		AW182675			-1.474776368	5.888339552	7.36311592
207754_at	RASSF8	NM_007211	11228	Hs.696433	-1.474688045	6.340369978	7.815058023
220946_s_at	SETD2	NM_014159	29072	Hs.517941	-1.474299633	8.425029569	9.899329202
1554096_a_at	RBM33	BC011923	155435	Hs.591815	-1.473483533	6.79945552	8.272939053
1555562_a_at	ZCCHC7	BC022434	84186	Hs.654700	-1.472850998	8.137674477	9.610525476
236254_at	VPS13B	BE048857	157680	Hs.191540	-1.472823462	8.076666426	9.549489888
242143_at		BE674964			-1.47039605	4.706490315	6.176886364
223399_x_at	PBRM1	AF197569	55193	Hs.189920	-1.469841658	4.981964389	6.451806047
217951_s_at	PHF3	AW189430	23469	Hs.348921	-1.467907271	8.539569227	10.0074765
236216_at		AA598661			-1.467839618	5.860533501	7.328373119
210214_s_at	BMPR2	U25110	659	Hs.471119	-1.46735355	6.579636554	8.046990103
205798_at	IL7R	NM_002185	3575	Hs.591742, Hs.635723	-1.467281898	5.648642903	7.115924801
203626_s_at	SKP2	NM_005983	6502	Hs.23348	-1.467102244	6.396719829	7.863822073
206829_x_at	ZNF430	NM_025189	80264	Hs.466289	-1.464824461	6.535711102	8.000535563
232152_at	C6orf182	BE567344	285753	Hs.632616	-1.464566902	5.037673624	6.502240527
226320_at	THOC4	AF047002	10189	Hs.534385	-1.464462763	10.18134413	11.64580689
203803_at	PCYOX1	N45309	51449	Hs.567502	-1.462493201	6.763049563	8.225542764
227384_s_at		AW340595			-1.461822814	8.119553416	9.581376229

235660_at		AW970002			-1.461424746	5.253006805	6.714431551
1565715_at	FUS	BE930017	2521	Hs.513522	-1.460700697	9.038345963	10.49904666
1554721_a_at	TAF2	AF040701	6873	Hs.122752	-1.458613129	8.065264646	9.523877774
237018_at		AI051967			-1.456779931	5.604607994	7.061387925
215188_at	STK24	AF339785	8428	Hs.508514, Hs.713712	-1.455300109	4.714740835	6.170040944
235055_x_at	MUC4	BF913667	4585	Hs.369646	1.454776845	6.466925956	5.012149111
227956_at	ITPRIPL2	AI458417	162073	Hs.530899, Hs.648523	-1.454769929	7.351867258	8.806637187
239946_at		AA776723			-1.453766921	5.989208656	7.442975577
244784_at	DHX57	T61977	90957	Hs.468226	-1.453758018	4.671951234	6.125709251
215310_at	APC	AF038181	324	Hs.158932	-1.453573518	4.622165258	6.075738776
1567014_s_at	NFE2L2	AF323119	4780	Hs.715540	-1.453019565	8.045046119	9.498065683
209677_at	PRKCI	L18964	5584	Hs.478199	-1.451249552	5.523149964	6.974399516
207686_s_at	CASP8	NM_001228	841	Hs.599762	-1.44944598	5.33563883	6.78508481
241413_at		W80457			-1.448721606	4.783021515	6.231743122
1554084_a_at	NOL9	BF969522	79707	Hs.59425	-1.448545554	6.649958655	8.098504209
238855_at	AHNAK	BF816551	79026	Hs.502756	-1.447875478	5.323539257	6.771414734
237502_at	CRLS1	BE671045	54675	Hs.224764	-1.447745653	5.574488477	7.02223413
1564907_s_at		AJ224167			-1.447246116	6.37237664	7.819622757
215198_s_at	CALD1	AU147402	800	Hs.490203	-1.447202306	5.79125867	7.238460975
216057_at	RAB3GAP2	AK021928	25782	Hs.654849, Hs.708165	-1.445855949	4.580392823	6.026248773
1563321_s_at	MLLT10	AF272384	8028	Hs.30385	-1.445773936	6.68675814	8.132532077
237867_s_at	PID1	AI142544	55022	Hs.715695	-1.445620883	7.783326655	9.228947538
225345_s_at		AU155376			-1.444484274	7.541853271	8.986337545
214682_at	LOC399491	AK023376	399491	Hs.648395, Hs.656655	-1.444425835	6.488723627	7.933149462
220220_at	LRRC37A4	NM_018001	55073	Hs.663277, Hs.675446	-1.443798548	6.768966322	8.212764869
228691_at	BICD1	AI694946	636	Hs.505202	-1.443075728	5.769906096	7.212981824
223323_x_at	TRPM7	BE044733	54822	Hs.512894	-1.442710502	7.895066364	9.337776866
217069_at	MLL4	AF105279	9757	Hs.676457, Hs.92236	1.44216835	6.154223498	4.712055148
205018_s_at	MBNL2	NM_005757	10150	Hs.657347	-1.440253388	6.489892957	7.930146345
219628_at	ZMAT3	NM_022470	64393	Hs.386299	-1.440020642	8.815952674	10.25597332
222955_s_at		AF168713			-1.439962031	8.161326672	9.601288703
1556204_a_at	ZNF814	AK096401	730051	Hs.634143, Hs.669024, Hs.71	-1.439170386	4.797385136	6.236555522
223899_at	PBRM1	AF225870	55193	Hs.189920	-1.438432641	4.621741996	6.060174637
225961_at	KLHDC5	AI334297	57542	Hs.505104	-1.436941579	8.121330046	9.558271625
215898_at	TTLL5	AK021879	23093	Hs.709609	-1.436560171	4.62445267	6.061012841
242972_at		AI022648			-1.435187554	5.526588046	6.9617756
1555434_a_at	SLC39A14	BC015770	23516	Hs.491232	-1.434329596	5.451304734	6.88563433
226999_at	RNPC3	AL137730	55599	Hs.632423, Hs.632430	-1.434063566	5.922424016	7.356487582
213556_at	LOC390940	BE673445	390940	Hs.22049	1.43372186	8.464941333	7.031219474
239414_at		BF942260			-1.432951607	7.329182622	8.762134229
222007_s_at	FKBP8	N95418	23770	Hs.173464	-1.430696208	5.159450616	6.590146823
223984_s_at	NUPL1	BC001104	9818	Hs.310453, Hs.719291	-1.43003904	7.696282192	9.126321232
202046_s_at	GRLF1	NM_004491	2909	Hs.509447	-1.429546959	7.183356228	8.612903186

240886_at	CASR	W37989	846	Hs.435615	1.428879341	6.400733931	4.97185459
224216_at		AF222858			-1.427721246	6.735577244	8.16329849
201070_x_at	SF3B1	AI739389	23451	Hs.632554	-1.426844521	9.776500912	11.20334543
212070_at	GPR56	AL554008	9289	Hs.513633	1.426547627	10.00448548	8.577937854
219980_at	C4orf29	NM_025097	80167	Hs.445817	-1.42649449	6.181756365	7.608250855
241824_at		AA019641			-1.426409215	5.711901776	7.138310992
239348_at		AI285970			-1.425554915	5.70644132	7.131996236
224254_x_at		AF116695			-1.425368006	4.699318695	6.124686701
1553186_x_at	RASEF	NM_152573	158158	Hs.657750	-1.423460608	9.063446524	10.48690713
235582_at	E2F2	BG388715	1870	Hs.194333	-1.422617865	5.936518534	7.359136399
1558078_at		BQ219651			-1.421840632	5.545265116	6.967105747
239783_at		AV699637			-1.421489247	6.309783278	7.731272525
1559458_at		L04489			-1.421319423	4.693522563	6.114841986
209013_x_at	TRIO	AF091395	7204	Hs.130031	-1.421199664	7.99632984	9.417529504
209996_x_at	PCM1	AA931266	5108	Hs.491148	-1.420983895	6.841472601	8.262456496
211205_x_at	PIP5K1A	U78577	8394	Hs.655131, Hs.707569	-1.41989075	6.409886465	7.829777215
236375_at		AA516469			-1.419301506	5.009264132	6.428565638
232784_at		R42604			-1.419086442	6.828068078	8.24715452
213577_at	SQLE	AA639705	6713	Hs.71465	-1.418774298	9.076993715	10.49576801
240232_at		AA503803			-1.417652213	5.723074018	7.140726231
215078_at	SOD2	AL050388	6648	Hs.487046	-1.41760267	9.331228061	10.74883073
228613_at	RAB11FIP3	BF183535	9727	Hs.531642	-1.417071681	7.507526113	8.924597794
217182_at	MUC5AC	Z34282	4586	Hs.534332, Hs.558950	1.416832876	6.924038188	5.507205312
232592_at		AU146731			-1.416618971	6.182299088	7.598918059
243117_at		AL038973			1.416417505	8.206799525	6.790382021
1557961_s_at	LOC10012798:	BG492376	100127983		-1.416171902	7.489339697	8.905511599
1569202_x_at		BF847120			-1.416113341	4.668961972	6.085075313
1555384_a_at	LARP4	BC022377	113251	Hs.26613	-1.41563412	8.649388116	10.06502224
228919_at		AA601031			-1.413873587	9.603116337	11.01698992
242114_at	BOLA2	BF088991	552900	Hs.444600, Hs.655267	-1.413475448	5.852777746	7.266253194
236533_at	ASAP1	AW236958	50807	Hs.655552	-1.411281796	8.08459377	9.495875566
204840_s_at	EEA1	AI916242	8411	Hs.567367	-1.411245318	8.853897695	10.26514301
211022_s_at	ATRX	BC002521	546	Hs.533526, Hs.645562, Hs.65	-1.411131568	7.473588778	8.884720345
238687_x_at	ZNF770	AV753065	54989	Hs.718498	-1.409718426	7.950974907	9.360693333
233658_at		AK022413			-1.409333259	5.170438173	6.579771431
242440_at		N52821			-1.40880093	4.813685687	6.222486617
236987_at	ALPK2	AI741514	115701	Hs.628152	1.407092729	7.536676224	6.129583496
229399_at	C10orf118	BF438440	55088	Hs.159066, Hs.603328	-1.405573521	8.668436531	10.07401005
1560659_at		AL832439			-1.405207103	4.905442723	6.310649826
242576_x_at	N4BP2L2	AW503542	10443	Hs.507680	-1.402963057	4.747160545	6.150123602
243841_at	SYNE2	BE673396	23224	Hs.525392	-1.402300787	9.899789236	11.30209002
233405_at		AU155384			-1.402202857	4.609031424	6.011234281
233122_at	KRTCAP2	AU147619	200185	Hs.516671, Hs.704676	1.401954755	8.142714873	6.740760118

1557124_at	LOC440104	AK091705	440104	Hs.616500	1.401952033	6.304711025	4.902758992
239005_at		AW675572			-1.401730062	5.384171882	6.785901944
222407_s_at	ZFP106	AI493587	64397	Hs.511143	-1.401580673	8.400458105	9.802038778
206667_s_at	SCAMP1	AF005037	9522	Hs.482587	-1.400679693	6.071049497	7.47172919
201353_s_at	BAZ2A	AI653126	11176	Hs.314263	1.400318531	8.73381754	7.333499009
243745_at		N74507			-1.399921266	7.158479702	8.558400968
1559119_at		W01252			-1.399737357	4.890818407	6.290555764
225856_at		BF512028			-1.399555907	8.915262665	10.31481857
210807_s_at	SLC16A7	AF049608	9194	Hs.439643	-1.398390844	7.637300072	9.035690916
216521_s_at	BRCC3	S72931	79184	Hs.558537	-1.397861258	8.938884842	10.3367461
244674_at		AA936428			-1.39769894	4.914022305	6.311721245
210984_x_at	EGFR	U95089	1956	Hs.488293	-1.397576559	7.378642912	8.776219471
59433_at		N32185			-1.397300646	5.627416309	7.024716955
208662_s_at	TTC3	AI885338	7267	Hs.368214	-1.396067241	9.084115322	10.48018256
225289_at	STAT3	AI139252	6774	Hs.463059	-2.395670195	8.880156004	11.2758262
1565882_at		AK022126			-1.395483502	4.990235873	6.385719375
212368_at	ZNF292	AA972711	23036	Hs.485892, Hs.656621	-1.395290003	6.704567321	8.099857324
1558002_at	STRAP	BQ944989	11171	Hs.719087	-1.395241309	6.337122103	7.732363412
202354_s_at	GTF2F1	AW190445	2962	Hs.68257	-1.394792654	6.956063101	8.350855756
213156_at		BG251521			-1.39453162	8.639414092	10.03394571
244801_at	PSMB7	AI248671	5695	Hs.213470	-1.394445541	5.800647006	7.195092546
240152_at		BF792954			-1.394443631	6.33016258	7.724606211
209055_s_at	CDC5L	AW268817	988	Hs.485471	-1.3943081	8.753496981	10.14780508
237568_at		H67156			-1.393232825	6.434854562	7.828087387
231939_s_at	BDP1	AJ238520	55814	Hs.258272	-1.392521823	5.641151729	7.033673552
238787_at		AA988769			-1.392312261	5.800175963	7.192488224
219978_s_at	NUSAP1	NM_018454	51203	Hs.615092	-1.391413734	9.785202118	11.17661585
234605_at	CDC14B	AK024886	8555	Hs.40582	-1.391402871	5.11957591	6.510978781
222267_at	TMEM209	BE619220	84928	Hs.267245	-1.39121638	5.81596159	7.20717797
222303_at		AV700891			-1.390397041	7.336632003	8.727029044
211503_s_at	RAB14	AF112206	51552	Hs.371563, Hs.713585	-1.389495502	9.179910324	10.56940583
219757_s_at	C14orf101	NM_017799	54916	Hs.497253	-1.388483353	6.452477666	7.840961019
1555920_at	CBX3	BU683892	11335	Hs.381189, Hs.706294	-1.387779782	6.693063395	8.080843177
242857_at		AA748613			-1.387603413	7.223905939	8.611509352
224631_at	ZFP91	AA758013	80829	Hs.524920	-1.386916566	9.259679914	10.64659648
208250_s_at	DMBT1	NM_004406	1755	Hs.279611	1.385892914	7.117856553	5.731963638
242210_at	ZNF24	AA749167	7572	Hs.514802	-1.385750349	5.74278766	7.128538009
212332_at	RBL2	BF110947	5934	Hs.513609	-1.384893886	9.391516575	10.77641046
215509_s_at	BUB1	AL137654	699	Hs.469649	-1.384556149	8.27825196	9.662808109
1557081_at	RBM25	AA580691	58517	Hs.531106	-1.384335935	8.264886654	9.649222589
243709_at	SLC38A9	BG054799	153129	Hs.649685	-1.384283697	5.657879163	7.04216286
235891_at	LOC10012817	AI961147	100128178	Hs.584880	1.384265298	8.649616613	7.265351315
216362_at		AJ251844			-1.383721372	5.697236741	7.080958113

241775_at		AW298119			-1.382800796	5.151462383	6.53426318
203096_s_at	RAPGEF2	BF439282	9693	Hs.113912	-1.381884365	5.809719805	7.191604169
232198_at		BF509125			-1.380645734	6.455516725	7.83616246
221043_at		NM_013395			-1.379548842	6.641738322	8.021287164
1556382_a_at	NARG1	AK091308	80155	Hs.715706	-1.379218877	4.820430546	6.199649422
239228_at		AI298887			-1.379058712	6.106426093	7.485484804
226107_at		AU156755			-1.37821753	7.303149462	8.681366992
229600_s_at	CPD	AW297717	1362	Hs.446079	-1.377642743	5.270044868	6.647687611
234723_x_at		AK024881			-1.377489699	7.556823871	8.93431357
213672_at	MARS	AA621558	4141	Hs.632707	1.377246068	9.128541496	7.751295428
1554930_a_at	FUT8	AB049740	2530	Hs.654961	-1.376485582	6.478510841	7.854996423
221955_at	GNA11	AI040021	2767	Hs.650575, Hs.654784	-1.376344466	5.423495103	6.799839569
211631_x_at	B4GALT1	M22921	2683	Hs.272011	-1.376263523	5.738634424	7.114897947
1559397_s_at	PRR14	BE788667	78994	Hs.293629	-1.37531619	6.201498302	7.576814492
205425_at	HIP1	NM_005338	3092	Hs.329266, Hs.619089	-1.373212086	7.142524238	8.515736323
209629_s_at	NXT2	AF201942	55916	Hs.25010	-1.373045055	5.940369677	7.313414732
229692_at		AW135003			-1.372761974	4.949092005	6.321853979
223578_x_at	MALAT1	AF113016	378938	Hs.642877, Hs.714394	-1.372727552	10.64251638	12.01524394
212382_at	TCF4	BF433429	6925	Hs.605153	-1.371358019	9.170417176	10.5417752
229684_s_at	ZNF644	AI582177	84146	Hs.173001	-1.371271002	8.719150649	10.09042165
201101_s_at	BCLAF1	BE963370	9774	Hs.486542	-1.370975873	10.02931004	11.40028591
228573_at	ANTXR2	BE673665	118429	Hs.162963	1.368350067	9.83555831	8.467208243
217370_x_at		S75762			-1.367340038	10.24734707	11.61468711
232913_at	TMED8	AC007954	283578	Hs.200413, Hs.26403	-1.366890397	7.018452124	8.385342521
236241_at	MED31	BF593977	51003	Hs.567493	-1.366730999	5.117303279	6.484034278
202600_s_at	NRIP1	AI824012	8204	Hs.155017	-1.366180777	7.043619517	8.409800294
233518_at		AU144449			-1.36604184	6.230468636	7.596510476
211721_s_at	ZNF551	BC005868	90233	Hs.109540	-1.364881117	6.3244758	7.689356916
209127_s_at	SART3	AW173076	9733	Hs.584842	-1.364229749	10.0325216	11.39675135
223111_x_at	ARID4B	BF058966	51742	Hs.533633, Hs.575782	-1.363197405	7.230047119	8.593244523
237551_at	URB1	AW444712	9875	Hs.473611	-1.362130148	4.901789419	6.263919567
242191_at		AI701905			-1.361884392	7.158983819	8.520868211
242575_at	SMG7	AW295593	9887	Hs.591463	-1.360845855	5.18368404	6.544529895
203975_s_at	CHAF1A	BF000239	10036	Hs.79018	-1.359937872	8.03729008	9.397227952
232013_at	C9orf102	AL133663	375748	Hs.432364	-1.358044943	8.514474385	9.872519328
228629_s_at		BF116063			-1.356705638	6.312474163	7.669179801
233818_at	RNF160	AK023499	26046	Hs.288773	-1.355498394	4.874015065	6.229513459
217024_x_at	SIRPA	AC004832	140885	Hs.581021, Hs.679042	-1.354968268	6.560101308	7.915069575
244248_at	TTC27	AI129850	55622	Hs.468125	-1.354600803	4.866409292	6.221010095
1558710_at		BI791845			-1.354096807	4.903700815	6.257797622
1554310_a_at	EIF4G3	BC030578	8672	Hs.467084	-1.353781325	8.033274223	9.387055548
242688_at		AI149880			-1.353269554	7.899027289	9.252296843
214543_x_at	QKI	AF142421	9444	Hs.510324, Hs.593520	-1.352354733	6.948739909	8.301094641

228634_s_at	CSDA	BF195718	8531	Hs.221889	-1.351894177	8.883223047	10.23511722
225237_s_at	MSI2	BF435123	124540	Hs.658922	-1.351704927	5.806564598	7.158269524
210256_s_at	PIP5K1A	U78576	8394	Hs.655131, Hs.707569	-1.351590697	6.888908086	8.240498783
226526_s_at		BG165420			-1.350998031	4.709719275	6.060717306
242321_at		AI628689			-1.350968689	7.438912324	8.789881012
201668_x_at	MARCKS	AW163148	4082	Hs.519909, Hs.712721	-1.350955704	8.156670226	9.50762593
240008_at		AI955765			-1.3502129	4.89797758	6.24819048
1559403_at		BC042096			1.350157357	6.184463818	4.834306461
238940_at	KLF12	BE328128	11278	Hs.373857, Hs.592500	-1.348683449	4.863993113	6.212676562
216711_s_at	TAF1	M73444	6872	Hs.158560	-1.348127008	6.095045369	7.443172377
1555154_a_at	QKI	AF142421	9444	Hs.510324, Hs.593520	-1.348096658	7.318471326	8.666567984
223627_at	MEX3B	AL136778	84206	Hs.104744	-1.348045117	5.108874328	6.456919445
1555754_s_at	ATN1	Z22814	1822	Hs.143766	-1.347946811	5.090740382	6.438687193
210995_s_at	TRIM23	AF230399	373	Hs.792	-1.34792047	6.757461794	8.105382264
220368_s_at	SMEK1	NM_017936	55671	Hs.533887	-1.347845687	8.578109252	9.92595494
201711_x_at	RANBP2	AI681120	5903	Hs.199561, Hs.715056	-1.347430985	9.696221112	11.0436521
1569142_at	TRIM13	BC029514	10206	Hs.436922	-1.347210598	4.839124035	6.186334633
1555153_s_at	FCHO2	BC014311	115548	Hs.719247	-1.346400965	5.973134215	7.319535179
238809_at	C5orf51	BF439305	285636	Hs.29899	-1.346160723	6.23765072	7.583811443
219262_at	SUV39H2	NM_024670	79723	Hs.554883	-1.34605887	6.160442718	7.506501589
235014_at	LOC147727	BF345728	147727	Hs.631616	-1.346026862	8.075185748	9.42121261
232704_s_at	LRRFIP2	AK025207	9209	Hs.719246	-1.345353577	5.085510664	6.430864242
236066_at		AI292073			-1.3450528	7.3943533	8.7394061
244682_at	CAMSAP1	H11471	157922	Hs.522493	-1.344318274	4.995774894	6.340093168
244401_at	LCA5	AI922939	167691	Hs.21945	-1.344313649	5.813784091	7.15809774
223950_s_at	FLYWCH1	AL136585	84256	Hs.655321	1.343897662	9.706417684	8.362520022
232551_at	SLC26A6	AA521443	65010	Hs.631925, Hs.663208	-1.343530657	4.696154192	6.039684849
211078_s_at	STK3	Z25422	6788	Hs.492333	-1.343469651	4.741008222	6.084477873
1568983_a_at		BI547087			-1.343233256	6.313835398	7.657068654
242696_at		AI252004			-1.342103933	6.47440567	7.816509603
234951_s_at	COL12A1	AL080250	1303	Hs.101302	-1.34131722	7.197894102	8.539211322
217813_s_at	SPIN1	NM_006717	10927	Hs.146804	-1.341195211	6.585723075	7.926918286
241736_at	FBXW2	AW501195	26190	Hs.494985	-1.341170061	6.05790138	7.39907144
205701_at	IPO8	NM_006390	10526	Hs.505136	-1.341109613	8.25803859	9.599148203
242325_at	YWHAH	AA909983	7533	Hs.226755	-1.340515105	5.637406966	6.97792207
233621_s_at	ARHGEF12	AL137456	23365	Hs.24598	-1.340384708	7.014428176	8.354812884
215515_at		AL049268			-1.339953286	7.255665518	8.595618804
1558733_at	ZBTB38	BE386445	253461	Hs.715534	-1.339726759	9.591266925	10.93099368
215415_s_at	LYST	U70064	1130	Hs.532411	-1.339455631	5.020339362	6.359794993
215220_s_at	TPR	AK023111	7175	Hs.279640	-1.339423374	9.097727478	10.43715085
243206_at		BE082914			-1.339123036	5.349385659	6.688508694
244852_at	DSEL	AU119545	92126	Hs.124673	-1.338409849	6.645928903	7.984338752
213362_at	PTPRD	N73931	5789	Hs.446083	-1.338372616	4.976065759	6.314438374

243304_at	LOC286109	AI733824	286109	Hs.660189	-1.338178339	6.718162463	8.056340802
235016_at	REEP3	AL118571	221035	Hs.499833	-1.337369715	5.442101346	6.77947106
232222_at	C18orf49	AK000229	400653	Hs.114191	1.337149825	6.947961846	5.610812021
207198_s_at	LIMS1	NM_004987	3987	Hs.597715, Hs.613268	-1.336856798	9.28505484	10.62191164
235632_at		AI638195			-1.336420754	5.350920263	6.687341016
232796_at		AK021556			-1.3356486	5.251533636	6.587182236
230377_s_at	TBC1D2B	BE504351	23102	Hs.719242	-1.334454905	6.271882413	7.606337318
1562681_at	LOC338651	AK055141	338651	Hs.633229, Hs.673618	-1.333946048	4.84695901	6.180905058
212808_at	NFATC2IP	AI884627	84901	Hs.513470	-1.333445987	8.906278082	10.23972407
214305_s_at	SF3B1	AW003030	23451	Hs.632554	-1.333056186	10.01343122	11.34648741
206654_s_at	POLR3G	NM_006467	10622	Hs.282387	-1.33259618	6.771350827	8.103947007
204741_at	BICD1	NM_001714	636	Hs.505202	-1.332593741	6.116303104	7.448896845
1553750_a_at	FAM76B	NM_144664	143684	Hs.288304	-1.33153671	7.082293198	8.413829909
206638_at	HTR2B	NM_000867	3357	Hs.421649	-1.330887261	6.140122292	7.471009553
232503_at	ELP2	AK022709	55250	Hs.8739	-1.330690401	6.54387974	7.874570141
228556_at	YTHDC1	AI990739	91746	Hs.175955	-1.330016965	8.639620384	9.969637349
209131_s_at	SNAP23	U55936	8773	Hs.719238	-1.329595147	7.040894696	8.370489844
222616_s_at	USP16	AI806796	10600	Hs.99819	-1.329418524	10.20854951	11.53796803
208629_s_at	HADHA	BG472176	3030	Hs.516032	-1.329312822	9.487031733	10.81634455
202082_s_at	SEC14L1	AV748469	6397	Hs.464184	-1.327407742	9.667419978	10.99482772
227524_at		H06187			-1.327043602	6.357530779	7.684574381
211467_s_at	NFIB	U70862	4781	Hs.644095	-1.327026587	5.756493442	7.083520029
1565717_s_at		BE930017			-1.326628125	8.426157402	9.752785527
210170_at	PDLIM3	BC001017	27295	Hs.85862	1.326533686	6.646843465	5.320309779
218618_s_at	FNDC3B	NM_022763	64778	Hs.159430	-1.326364698	7.43613024	8.762494938
240757_at	CLASP1	H49240	23332	Hs.469840, Hs.708183	-1.326237518	5.102066387	6.428303905
224098_at		AF116638			-1.325761246	5.048239411	6.374000656
232615_at		AA632758			-1.325027243	7.767170598	9.092197841
1569126_at	CCNC	BC026272	892	Hs.430646, Hs.633351	-1.32422139	4.815504046	6.139725436
231012_at	TMEM20	AI123333	159371	Hs.632085	-1.323584403	5.066101711	6.389686114
205356_at	USP13	NM_003940	8975	Hs.175322	-1.322552331	8.497912219	9.820464551
225942_at	NLN	AB033052	57486	Hs.247460	-1.32237603	7.00261547	8.324991499
211340_s_at	MCAM	M28882	4162	Hs.599039	1.321767936	9.081714196	7.759946259
1562957_at		BC010059			-1.321453436	5.410445408	6.731898844
215855_s_at	TMF1	AK021741	7110	Hs.267632	-1.320969537	5.198394393	6.51936393
1553691_at	B3GALNT2	NM_152490	148789	Hs.716571	-1.319472711	5.993533096	7.313005807
229301_at	TUG1	AA046436	55000	Hs.554829	-1.318946756	5.37914388	6.698090636
202072_at	HNRNPL	NM_001533	3191	Hs.644906	-1.318011838	10.08834282	11.40635466
207604_s_at	SLC4A7	NM_003615	9497	Hs.250072	-1.318001293	8.27371944	9.591720733
231716_at	RC3H2	AF255304	54542	Hs.533499	-1.317529859	8.469914058	9.787443917
206967_at	CCNT1	NM_001240	904	Hs.279906	-1.316871094	6.068133269	7.385004363
219717_at	DCAF16	NM_017741	54876	Hs.614787	-1.316733539	8.228791588	9.545525127
217054_at	MUC3A	AF007194	4584	Hs.489354	1.31648546	6.77985026	5.4633648

1557372_at	FLJ41757	AF086285	440862	Hs.437691	-1.315983972	5.007216507	6.323200479
243025_at		AL119189			-1.315326362	5.064529933	6.379856295
213962_s_at	ANKLE2	AI924382	23141	Hs.654628	-1.315144625	8.290013647	9.605158272
217218_at	WAPAL	AK027005	23063	Hs.203099, Hs.714876	-1.314788713	5.821917763	7.136706476
242691_at		AA829017			-1.314469123	6.862288632	8.176757756
235188_at	C14orf135	AI479328	64430	Hs.509499	-1.314439501	5.131392994	6.445832495
226971_at	CCDC136	AI678057	64753	Hs.521178	1.313948845	6.532487768	5.218538923
226429_at	KIAA1704	BE218238	55425	Hs.507922	-1.313821625	7.027634659	8.341456284
215481_s_at	PEX5	AW468717	5830	Hs.567327	-1.313731045	6.272137225	7.58586827
212417_at	SCAMP1	BF058944	9522	Hs.482587	-1.313377932	8.602127992	9.915505924
236371_s_at	TGS1	AI806471	96764	Hs.335068	-1.313068745	9.008357443	10.32142619
223139_s_at	DHX36	BE501133	170506	Hs.446270	-1.312967682	9.982043671	11.29501135
233888_s_at	SRGAP1	AK023899	57522	Hs.210751, Hs.593803	-1.312944102	8.650095587	9.963039689
1558647_at	SH3D19	AA100736	152503	Hs.567725	-1.311990593	6.27182085	7.583811443
204909_at	DDX6	NM_004397	1656	Hs.654366	-1.311697208	9.508175569	10.81987278
201224_s_at	SRRM1	AU147713	10250	Hs.18192	-1.311336968	9.346598119	10.65793509
236268_at	SEC22C	BF732413	9117	Hs.445892	-1.310707886	6.522245347	7.832953233
212095_s_at	MTUS1	BE552421	57509	Hs.7946	-1.310406422	6.153421528	7.463827951
241624_at	LOC389834	BE221330	389834	Hs.595418	-1.310214836	7.167058484	8.47727332
239729_at		BE066500			-1.309971673	5.020360401	6.330332074
220820_at		NM_018539			-1.309769745	5.282765153	6.592534898
211607_x_at	EGFR	U48722	1956	Hs.488293	-1.309136835	7.427822831	8.736959666
218521_s_at	UBE2W	NM_018299	55284	Hs.597486	-1.308870212	7.296303867	8.605174079
232229_at	SETX	AK024331	23064	Hs.460317	-1.308665687	6.776675601	8.085341288
238563_at		AV762916			-1.308412426	5.236117469	6.544529895
1557938_s_at	PTRF	AL545542	284119	Hs.437191	-1.307736804	8.274081063	9.581817867
213328_at	NEK1	AI936517	4750	Hs.481181	-1.307540093	8.689462969	9.997003062
208221_s_at	SLIT1	NM_003061	6585	Hs.632082	1.307449415	6.22824244	4.920793025
239735_at		N67106			-1.306876145	5.642266564	6.949142709
239606_at		AA669135			-1.306719255	6.241278002	7.547997257
219790_s_at	NPR3	NM_000908	4883	Hs.237028, Hs.619466	-1.306684105	7.129699013	8.436383118
209936_at	RBM5	AF107493	10181	Hs.439480	-1.306383333	5.778401592	7.084784925
1554390_s_at	ACTR2	BC036253	10097	Hs.643727, Hs.719274	-1.30597541	9.710617726	11.01659314
213352_at	TMCC1	AB018322	23023	Hs.477547, Hs.709936	-1.305506158	5.597027898	6.902534056
220219_s_at		NM_018001			-1.305432271	9.799713708	11.10514598
208677_s_at	BSG	AL550657	682	Hs.501293	-1.304238157	10.17451133	11.47874949
223584_s_at	KBTBD2	BF000166	25948	Hs.372541	-1.304097864	7.834912456	9.13901032
209700_x_at	PDE4DIP	AB042555	9659	Hs.584841, Hs.613082, Hs.65	-1.303117991	4.750662753	6.053780744
235112_at		AA088388			-1.301909383	6.065458742	7.367368125
1566557_at	FLJ90757	AK096609	440465	Hs.448889	1.301743859	8.892330933	7.590587074
214734_at	EXPH5	AB014524	23086	Hs.28540	-1.301467018	4.836431761	6.137898779
1555058_a_at	LPGAT1	BC034621	9926	Hs.497674	-1.30139593	8.291036306	9.592432236
205966_at	TAF13	NM_005645	6884	Hs.632426	-1.301225835	6.292641238	7.593867073

231689_at	TRPM7	BE044721	54822	Hs.512894	-1.301154494	7.453995946	8.755150439
230892_at		AI912194			-1.300677231	4.951023867	6.251701098
242630_at		AA005355			-1.300468719	4.70362295	6.00409167
243070_at	INO80D	AI954752	54891	Hs.445036	-1.30036918	6.330298764	7.630667944
236019_at	RAB12	AI076335	201475	Hs.270074	-1.300334322	4.727083507	6.027417829
1568822_at	GTPBP5	BC040178	26164	Hs.340636	-1.300306879	4.817847738	6.118154616
206744_s_at	ZMYM5	NM_014242	9205	Hs.530988	-1.300182889	5.299835222	6.600018111
235789_at	KDM4B	AW450344	23030	Hs.654816	-1.298941725	5.418251845	6.717193571
214374_s_at	PPFIBP1	AI962377	8496	Hs.172445	-1.298317569	9.868586263	11.16690383
1563497_at	USP25	AL833500	29761	Hs.473370	-1.298295571	5.015546548	6.31384212
201732_s_at	CLCN3	AF029346	1182	Hs.481186	-1.298279636	8.746095112	10.04437475
203293_s_at	LMAN1	NM_005570	3998	Hs.465295	-1.298123746	6.869741747	8.167865493
208610_s_at	SRRM2	AI655799	23524	Hs.433343, Hs.719123	-1.297386298	8.179104486	9.476490784
205868_s_at	PTPN11	L07527	5781	Hs.506852, Hs.646231	-1.29666565	7.246898368	8.543564018
207319_s_at	CDC2L5	NM_003718	8621	Hs.233552	-1.296413809	7.17253282	8.468946628
232835_at		AA533080			-1.295979577	6.339534491	7.635514068
238988_at		AI863675			-1.295580239	5.422064332	6.717644572
241508_at		T63605			-1.295369174	4.966813911	6.262183086
241865_at		AI056689			-1.295106513	6.61795125	7.913057763
208711_s_at	CCND1	BC000076	595	Hs.523852	-1.294351979	7.982387974	9.276739953
239393_at		AW510927			-1.293612567	5.500979274	6.794591841
239965_at	LOC151878	AW009761	151878	Hs.680377	-1.29337665	5.606779576	6.900156226
213478_at	RP1-21O18.1	AB028949	23254	Hs.368823	-1.293206146	6.647431676	7.940637822
209438_at	PHKA2	AL096700	5256	Hs.54941, Hs.607628, Hs.622	1.293135642	6.649564586	5.356428945
1560100_at	DLX1	AK095266	1745	Hs.407015	-1.292939331	5.556895759	6.849835089
239899_at	RNF145	AW378236	153830	Hs.349306	-1.292893664	8.083994475	9.37688814
201559_s_at	CLIC4	AF109196	25932	Hs.440544, Hs.595507	-1.292768984	8.834023034	10.12679202
236696_at	SR140	BE464843	23350	Hs.596572	-1.292743908	6.402185985	7.694929893
1554466_a_at	C16orf13	BC007207	84326	Hs.239500	-1.292228222	9.02029198	10.3125202
1559722_at		BC042892			-1.292157355	5.853621989	7.145779344
212420_at	ELF1	AL559590	1997	Hs.135646	-1.291597912	9.652938829	10.94453674
208553_at	HIST1H1E	NM_005321	3008	Hs.248133	-1.290598191	5.501012188	6.791610379
214941_s_at	PRPF40A	AA675900	55660	Hs.643580	-1.290501014	7.426592028	8.717093041
218300_at	C16orf53	NM_024516	79447	Hs.702841	-1.289837255	10.34709716	11.63693441
231520_at	SLC35F3	BF195423	148641	Hs.158748	1.289763249	6.057214247	4.767450998
220254_at	LRP12	NM_013437	29967	Hs.600630	-1.289313357	7.032215527	8.321528883
240050_s_at		BF751607			-1.288983736	7.015039127	8.304022863
211804_s_at	CDK2	AB012305	1017	Hs.19192, Hs.689624	-1.288951837	5.249270112	6.538221949
231011_at	LARP1B	AI339785	55132	Hs.657067	-1.288265127	6.922993127	8.211258254
213272_s_at	TMEM159	AF070596	57146	Hs.258212	-1.288234595	6.541425976	7.829660571
214192_at	NUP88	Y08613	4927	Hs.584784	-1.288227173	6.942002515	8.230229687
237554_at		BE348304			-1.28756907	5.293317506	6.580886576
229284_at	MAT2B	R60683	27430	Hs.54642	-1.287509414	6.153421528	7.440930942

215203_at	GOLGA4	AW438464	2803	Hs.344151	-1.286782041	6.999026296	8.285808337
219221_at	ZBTB38	NM_024724	253461	Hs.715534	-1.286427739	9.892076002	11.17850374
231957_s_at	DPP9	AC005594	91039	Hs.515081	-1.286372284	5.943160107	7.229532391
1559496_at	PPA2	AL833123	27068	Hs.654957	-1.284540756	5.07797186	6.362512616
1555469_a_at	CLASP2	BC029035	23122	Hs.108614	-1.28401349	6.129451231	7.413464721
205187_at	SMAD5	AF010601	4090	Hs.167700	-1.283263374	8.421567265	9.704830639
229434_at		AA865357			-1.283038777	7.291052364	8.574091141
1552677_a_at	DIP2A	NM_015151	23181	Hs.189585	-1.282053443	5.389207816	6.671261259
213490_s_at	MAP2K2	AI762811	5605	Hs.465627	-1.280531933	6.212971522	7.493503455
215062_at		AL390143			-1.280445033	4.916096696	6.196541729
222311_s_at	SFRS15	AA648521	57466	Hs.17255	-1.280400813	8.166726959	9.447127773
225640_at	LOC401504	AA875998	401504	Hs.446271	-1.280392235	7.952162552	9.232554788
244515_at	PSMD7	AI640348	5713	Hs.440604	-1.280318685	5.018796249	6.299114933
215820_x_at	SNX13	AK001861	23161	Hs.487648	-1.279675056	5.949799401	7.229474457
215434_x_at		AV684285			-1.279433066	8.733824919	10.01325798
216213_at	NEK1	AF155113	4750	Hs.481181	-1.279051377	5.743469921	7.022521298
241701_at	ARHGAP21	BF369489	57584	Hs.524195	-1.278878798	6.275720027	7.554598825
228628_at	SRGAP2P1	AI478268	653464	Hs.523529, Hs.698027	-1.278421358	7.54442516	8.822846519
211085_s_at	STK4	Z25430	6789	Hs.472838	-1.278408633	7.319848982	8.598257615
243003_at		AV702197			-1.278214508	7.687762031	8.965976539
235938_at		AI018174			-1.277558372	5.85669793	7.134256302
219878_s_at	KLF13	NM_015995	51621	Hs.525752	-1.277148479	4.842546038	6.119694516
213862_at	PNPLA2	AI979087	57104	Hs.654697	-1.276894974	4.920804589	6.197699563
212078_s_at	MLL	AA704766	4297	Hs.258855	-1.276275595	8.588960137	9.865235732
200641_s_at	YWHAZ	U28964	7534	Hs.492407, Hs.594673	-1.275922229	10.21998673	11.49590896
229462_at	C9orf82	AW296077	79886	Hs.178357	-1.275423708	4.908904642	6.18432835
215990_s_at	BCL6	S67779	604	Hs.478588	-1.272944253	8.469449528	9.742393782
225383_at	ZNF275	BF793625	10838	Hs.348963	-1.272248872	8.400225694	9.672474565
241193_at	ETS2	AI797080	2114	Hs.644231	-1.272128842	6.749389048	8.02151789
229281_at	NPAS3	N51682	64067	Hs.657892	1.271806463	6.047517018	4.775710556
227514_at	ITPRIPL2	AI766311	162073	Hs.530899, Hs.648523	-1.271683328	9.669311696	10.94099502
214487_s_at		NM_002886			-1.270796106	6.00151018	7.272306286
238419_at	PHLDB2	T68150	90102	Hs.477114, Hs.655022, Hs.67	-1.270751406	9.069868683	10.34062009
208960_s_at	KLF6	BE675435	1316	Hs.4055, Hs.709396	-1.270334711	9.201748707	10.47208342
227954_at	ITPRIPL2	AI458417	162073	Hs.530899, Hs.648523	-1.269381429	9.701618721	10.97100015
217278_x_at		AF023203			1.269203175	6.163016533	4.893813358
205457_at	C6orf106	NM_024294	64771	Hs.643498	-1.268869625	8.111803214	9.380672839
201971_s_at	ATP6V1A	NM_001690	523	Hs.477155	-1.268837858	5.947458765	7.216296623
236007_at	AKAP10	AU147278	11216	Hs.708043	-1.268596139	6.708847711	7.97744385
230808_at	FNTA	AA833870	2339	Hs.370312	-1.268279725	5.537101394	6.805381119
206668_s_at	SCAMP1	NM_004866	9522	Hs.482587	-1.267548328	8.133187858	9.400736185
241893_at		BE927766			-1.267518025	4.973645542	6.241163568
232975_at	HCG18	AK023334	414777	Hs.485041, Hs.717017	-1.267076707	6.976955052	8.24403176

214693_x_at	NBPF10	BE732345	100132406	Hs.515947	-1.266591716	10.21484226	11.48143398
232309_at	LOC202181	Y13871	202181	Hs.189914, Hs.653052	-1.266480366	5.996492386	7.262972752
201647_s_at	SCARB2	NM_005506	950	Hs.349656, Hs.714206	-1.265825498	8.169296458	9.435121956
241359_at		AA729232			1.265779908	7.775978666	6.510198758
206108_s_at	SFRS6	NM_006275	6431	Hs.6891	-1.265576882	8.063151755	9.328728636
244110_at	MLL	BE669782	4297	Hs.258855	1.263292462	7.764430056	6.501137594
242752_at		AI434789			-1.262371324	7.398094237	8.660465561
209701_at		D16217			-1.262251565	9.764894044	11.02714561
216357_at	SNX1	AL050148	6642	Hs.188634	-1.261759404	4.916301434	6.178060838
1556277_a_at	PAPD4	BG542611	167153	Hs.418198	-1.26088477	6.479268466	7.740153236
229571_at		AV763524			-1.260820716	5.499274285	6.760095001
236235_at	ITCH	AA868238	83737	Hs.632272	-1.260645275	5.092415724	6.353060998
229765_at	ZNF207	AW511239	7756	Hs.500775	-1.260607579	6.058611825	7.319219404
213922_at	TTBK2	AW294686	146057	Hs.659846, Hs.713940	-1.260040325	5.927489736	7.18753006
1552873_s_at	NCRNA00105	NM_025091	80161	Hs.521856	1.259724536	7.381209612	6.121485076
87100_at	ABHD2	AI832249	11057	Hs.122337, Hs.705984	-1.259557689	6.961745523	8.221303211
216695_s_at	TNKS	AF082559	8658	Hs.370267	-1.258755583	5.214286589	6.473042172
235011_at	MAP3K2	BG504375	10746	Hs.145605	-1.258156045	4.88330884	6.141464885
213948_x_at	CADM3	AI564838	57863	Hs.365689	-1.257122804	6.25999701	7.517119814
206275_s_at	MICAL2	NM_014632	9645	Hs.501928	-1.256421864	7.383157157	8.639579022
238134_at		AW885748			-1.255656517	4.833445969	6.089102486
1553536_at	MBNL2	NM_018615	10150	Hs.657347	-1.255444675	6.69745415	7.952898825
1555337_a_at	ZNF317	AF307097	57693	Hs.465829	-1.255186312	4.973819562	6.229005874
225820_at	PHF17	AV646599	79960	Hs.12420	-1.254515265	8.570024496	9.824539762
235596_at		BE562520			-1.254299623	6.569569981	7.823869604
220467_at		NM_025032			-1.253477057	5.402124356	6.655601413
234562_x_at		AK000115			-1.252419832	5.939184337	7.191604169
242732_at		BG010493			-1.252289194	5.66992188	6.922211074
232903_at		BF680284			-1.251966417	6.659007274	7.910973691
214773_x_at	TIPRL	AI983505	261726	Hs.209431	-1.251865029	7.543997577	8.795862606
1558504_at		AF086554			-1.251814583	5.906413795	7.158228377
232400_at		AA703174			-1.251651944	5.440976177	6.692628121
221064_s_at	UNKL	NM_023076	64718	Hs.643536	-1.251373937	9.193204805	10.44457874
242135_at		AA927533			-1.250508734	7.115018802	8.365527535
1560250_s_at	LOC284242	BC035844	284242	Hs.714049	-1.250284436	4.90895327	6.159237706
1558697_a_at	KIAA0430	BI600341	9665	Hs.173524	-1.249537595	5.476297253	6.725834848
236961_at		AI539426			-1.249468643	5.351842669	6.601311312
214314_s_at	EIF5B	BE138647	9669	Hs.158688	1.247581563	11.13272449	9.885142925
200767_s_at	FAM120A	NM_014612	23196	Hs.372003, Hs.707324	-1.246824687	8.280440254	9.527264941
244187_at		AA053853			-1.246584775	7.049689346	8.296274121
239432_at	FLJ31306	AV729086	379025	Hs.531089	-1.245967111	5.894793037	7.140760148
217662_x_at		AI393960			-1.245743312	5.880596074	7.126339386
217486_s_at	ZDHHC17	AF161412	23390	Hs.4014	-1.244288545	6.005595305	7.24988385

228686_at	FLJ33630	BE217923	644873	Hs.340623	-1.242926601	7.299788507	8.542715108
241669_x_at	PRKD2	AI251399	25865	Hs.466987	1.242876512	7.313319262	6.070442751
206471_s_at	PLXNC1	NM_005761	10154	Hs.584845	-1.24191349	5.615696409	6.857609899
204864_s_at	IL6ST	NM_002184	3572	Hs.532082	-1.241806295	8.056579546	9.298385842
240018_at		AI733650			-1.241461193	4.795983235	6.037444428
204375_at	CLSTN3	NM_014718	9746	Hs.535378	1.241391519	6.491452871	5.250061351
209151_x_at	TCF3	AA768906	6929	Hs.371282	-1.241146711	5.390795232	6.631941943
234332_at	NUB1	AK026433	51667	Hs.647082	-1.239623331	8.449821203	9.689444534
1568877_a_at	ACBD5	BC025309	91452	Hs.530597	-1.23950849	7.429406258	8.668914748
1564002_a_at	AKD1	AK092103	221264	Hs.205144, Hs.335027, Hs.48	-1.238809808	7.294745166	8.533554974
219754_at	RBM41	NM_018301	55285	Hs.139053	-1.238478727	6.874635594	8.113114321
222907_x_at	TMEM50B	BC000569	757	Hs.433668	-1.237545597	8.392375559	9.629921156
216427_at		AK026439			1.237272718	6.917435229	5.680162511
241854_at	DNASE1	AI129699	1773	Hs.629638	-1.237139666	5.540625909	6.777765575
230702_at	C8orf16	BE674736	83735	Hs.660259	-1.235474495	5.470517784	6.705992279
1559410_at		AA524609			-1.235268437	4.854710072	6.089978509
1557170_at	NEK8	AI073943	284086	Hs.448468	1.233523415	6.82009534	5.586571925
201777_s_at	KIAA0494	BC002525	9813	Hs.719205	-1.233078125	9.328622138	10.56170026
212759_s_at	TCF7L2	AI703074	6934	Hs.593995	-1.232080465	8.75829186	9.990372325
213873_at	DCBLD2	D29810	131566	Hs.203691	-1.230928759	8.268502746	9.499431505
1561092_at		AL833766			-1.230445525	4.806998903	6.037444428
224588_at	XIST	AA167449	7503	Hs.529901	-1.230354336	10.4825189	11.71287324
240859_at	ZFYVE16	N20928	9765	Hs.482660, Hs.660410	-1.230285734	5.664102642	6.894388376
231366_at	FDPSL2A	AI190575	619190		-1.230179283	5.323062461	6.553241744
227297_at	ITGA9	AI479176	3680	Hs.113157	1.229328449	6.251649158	5.022320709
201109_s_at	THBS1	AV726673	7057	Hs.164226	-1.229070428	7.262168885	8.491239313
221041_s_at	SLC17A5	NM_012434	26503	Hs.597422	-1.228689335	5.8585053	7.087194635
230441_at	PLEKHG4B	AI890356	153478	Hs.535800	1.228310028	6.012921988	4.78461196
209610_s_at	SLC1A4	BF340083	6509	Hs.654352	1.227338456	9.100758195	7.873419739
240286_at		AL041745			1.227265608	8.110074193	6.882808585
239331_at		AW954199			-1.226576333	8.160243922	9.386820255
1555559_s_at	USP25	AF419247	29761	Hs.473370	-1.225232664	5.442351442	6.667584106
1569495_at	SCLT1	BC040258	132320	Hs.654690	-1.225037379	4.852105883	6.077143262
201110_s_at	THBS1	NM_003246	7057	Hs.164226	-1.224529751	8.270543949	9.4950737
214631_at	ZBTB33	BG391005	10009	Hs.143604	-1.224018717	5.022082646	6.246101363
217614_at		AI439416			-1.223420824	6.353475987	7.576896811
228801_at	ORMDL1	AI809749	94101	Hs.709387	-1.223415107	5.699803711	6.923218818
203666_at	CXCL12	NM_000609	6387	Hs.522891	1.223158718	7.43613024	6.212971522
240636_at		AI221207			-1.222310462	4.832510747	6.054821209
1558041_a_at	KIAA0895L	AL834156	653319	Hs.651201	1.221575843	9.012496632	7.790920788
236346_at		BF115793			-1.220996546	7.28023109	8.501227636
239926_at		AI675753			-1.220934893	5.29348188	6.514416772
227487_s_at	SERPINE2	AI359165	5270	Hs.38449, Hs.708453	-1.220558033	6.069027779	7.289585811

204560_at	FKBP5	NM_004117	2289	Hs.407190	-1.220495145	10.12412354	11.34461868
200671_s_at	SPTBN1	N92501	6711	Hs.503178, Hs.705692	-1.220391676	8.383179947	9.603571623
211086_x_at	NEK1	Z25431	4750	Hs.481181	-1.218813548	7.544268905	8.763082453
236134_at	DCAF7	AA769995	10238	Hs.410596	-1.21796388	5.294754063	6.512717943
224877_s_at	MRPS5	BE970056	64969	Hs.655259	-1.217912651	7.698383356	8.916296008
201996_s_at	SPEN	AL524033	23013	Hs.558463	-1.21755908	8.411866364	9.629425444
226975_at	RNPC3	BF116157	55599	Hs.632423, Hs.632430	-1.217488424	7.647396111	8.864884535
222146_s_at	TCF4	AK026674	6925	Hs.605153	-1.217415705	8.166280876	9.383696582
214854_at		AC004490			-1.216781202	5.936940748	7.15372195
223746_at	STK4	BC005231	6789	Hs.472838	-1.216693481	5.756059085	6.972752567
243694_at		BE551421			-1.216581651	6.447572496	7.664154147
230703_at		AA001543			-1.216010989	4.887190386	6.103201375
236545_at		AA532718			-1.215366914	6.081367618	7.296734531
242712_x_at		BE856960			-1.215191328	6.915822595	8.131013923
242445_at	FGD4	AA296351	121512	Hs.117835	-1.214499234	5.058453697	6.272952931
223113_at	TMEM138	AF151030	51524	Hs.406530	1.214222969	10.65285711	9.438634137
230058_at		AI738717			-1.213667313	5.880434779	7.094102092
207983_s_at	STAG2	NM_006603	10735	Hs.496710, Hs.624663	-1.213032827	8.829926549	10.04295938
227101_at	ZNF800	AF218032	168850	Hs.159006	-1.212384156	9.00087136	10.21325552
228246_s_at	SPTBN1	AA772306	6711	Hs.503178, Hs.705692	-1.212214627	6.143651297	7.355865924
242407_at		H71242			-1.212009645	6.405516093	7.617525738
215888_at	PDS5B	AK026889	23047	Hs.716441	-1.211949745	4.884105278	6.096055023
213016_at	BBX	AA573805	56987	Hs.124366	-1.211928276	8.444417813	9.656346089
1569025_s_at	FAM13A	BC041029	10144	Hs.97270	-1.211607149	7.282089532	8.493696681
210180_s_at	TRA2B	U87836	6434	Hs.533122	-1.211530869	9.987273173	11.19880404
214242_at	MAN1A2	AI189305	10905	Hs.435938	-1.211268482	6.37878693	7.590055413
224250_s_at	SECISBP2	BC001189	79048	Hs.59804	-1.211169801	8.775222953	9.986392754
227658_s_at	PLEKHA3	BE550332	65977	Hs.41086	-1.210950405	6.668222252	7.879172657
201945_at	FURIN	NM_002569	5045	Hs.513153	-1.210282222	9.358836937	10.56911916
232964_at	SPDYE1	AL137266	285955	Hs.645483	-1.210101775	6.704862552	7.914964327
213406_at	WSB1	AA521269	26118	Hs.446017	-1.209096407	7.36565777	8.574754176
203656_at	FIG4	NM_014845	9896	Hs.529959	1.208976164	10.80064126	9.5916651
230260_s_at	RFTN1	BF434651	23180	Hs.98910	-1.208490406	4.82985972	6.038350125
213326_at	VAMP1	AU150319	6843	Hs.20021	1.208381205	10.13187003	8.923488822
236571_at		AW197431			-1.208242986	5.093754497	6.301997483
221496_s_at	TOB2	D64109	10766	Hs.719120	-1.207811872	5.896805129	7.104617001
244881_at	LMLN	AA629059	89782	Hs.518540	-1.207377441	8.43863873	9.646016171
1552695_a_at	SLC2A13	NM_052885	114134	Hs.558595	-1.207167514	6.213720364	7.420887878
236862_at	GOPC	AA279958	57120	Hs.191539	-1.206845994	5.194702386	6.401548379
228156_at		AW342078			-1.206782376	7.172868499	8.379650875
1556449_at		BM717927			1.206258961	7.940637822	6.73437886
227121_at		BF476076			-1.205725163	6.91129401	8.117019172
239817_at		AI803727			-1.204898247	5.013807335	6.218705582

228209_at		AI147033			1.204649986	8.49536075	7.290710765
223123_s_at	C1orf128	AI867781	57095	Hs.31819	-1.20429164	6.700675323	7.904966963
203007_x_at	LYPLA1	AF077198	10434	Hs.435850	-1.204095391	9.056644086	10.26073948
235859_at	MLL3	BE551763	58508	Hs.647120	-1.203962214	5.023903365	6.227865579
219600_s_at	TMEM50B	NM_006134	757	Hs.433668	-1.203185826	9.850060069	11.0532459
204827_s_at	CCNF	U17105	899	Hs.1973	-1.202896281	6.057993637	7.260889918
236181_at		R38704			-1.202617583	5.82727925	7.029896833
217506_at	LOC339290	H49382	339290	Hs.643553, Hs.700799	-1.202553835	5.131655531	6.334209366
232614_at		AU146963			-1.202384826	4.934928269	6.137313095
201646_at	SCARB2	AA885297	950	Hs.349656, Hs.714206	-1.202359567	8.991974187	10.19433375
240089_at		BF508868			-1.202307656	5.643250693	6.84555835
227953_at	CMTM6	AW301108	54918	Hs.380627, Hs.440494	-1.202296685	8.736631384	9.938928069
1557289_s_at	GTF2IRD2	BU617476	84163	Hs.647017, Hs.647039	-1.202126743	5.095155513	6.297282256
222626_at	RBM26	T79937	64062	Hs.558528	-1.202046023	8.757635933	9.959681956
204074_s_at	KIAA0562	AI936976	9731	Hs.133089, Hs.509017	-1.201861866	8.660638477	9.862500343
1569540_at		BC035958			-1.201732398	5.489338383	6.691070781
243141_at	SGMS2	D31421	166929	Hs.595423	-1.201118405	7.955217889	9.156336294
226931_at	TMTC1	AU151239	83857	Hs.401954	-1.198407945	8.358756888	9.557164833
209920_at	BMPR2	U20165	659	Hs.471119	-1.198122957	7.918085237	9.116208194
232421_at	SCARB1	AV703311	949	Hs.709216	-1.197433272	6.10514764	7.302580913
238346_s_at	TGS1	AW973003	96764	Hs.335068	-1.196925592	10.20048162	11.39740721
208790_s_at	PTRF	AF312393	284119	Hs.437191	-1.196656154	10.08624705	11.2829032
233416_at		AU144915			1.195457405	6.277267029	5.081809624
1552610_a_at	JAK1	NM_002227	3716	Hs.207538	-1.195044495	9.100185924	10.29523042
238811_at	ATP11B	AA639797	23200	Hs.478429	-1.194310825	6.960158825	8.154469651
208451_s_at		NM_000592			1.194280369	6.500061922	5.305781553
242579_at	BMPR1B	AA935461	658	Hs.598475	-1.19425234	6.877913418	8.072165758
206127_at	ELK3	NM_005230	2004	Hs.46523	-1.19378769	6.114514637	7.308302326
1555240_s_at	GNG12	AF493879	55970	Hs.431101	-1.193032829	6.914955416	8.107988246
201779_s_at	RNF13	AF070558	11342	Hs.12333, Hs.712766	-1.192853275	9.71767379	10.91052706
206917_at	GNA13	NM_006572	10672	Hs.515018	-1.192558952	5.083343458	6.27590241
235294_at	SIKE1	AV685172	80143	Hs.709277	-1.192511141	6.606318377	7.798829518
213593_s_at	TRA2A	AW978896	29896	Hs.445652	-1.191966927	5.292190284	6.484157212
237067_at		C15240			-1.191864431	6.246314127	7.438178558
212529_at	LSM12	BF197707	124801	Hs.355570	-1.191847768	9.869532566	11.06138033
217856_at	RBM8A	AF182415	9939	Hs.356873	-1.191407806	9.252267297	10.4436751
222640_at	DNMT3A	N26002	1788	Hs.515840	1.190433829	9.434465588	8.24403176
221628_s_at	GLYR1	AF326966	84656	Hs.387255	-1.189744348	5.323539257	6.513283605
202316_x_at	UBE4B	AW241715	10277	Hs.632370	-1.189256871	8.877607864	10.06686473
233037_at		AF138859			-1.188567049	5.136609704	6.325176754
1560622_at		AK000203			-1.188207507	7.451913439	8.640120946
238758_at		AA749253			1.187417206	8.004521779	6.817104573
233211_at	MTBP	AK022122	27085	Hs.657656	-1.187394621	4.89753711	6.084931731

1559585_at	DDX60L	AK096369	91351	Hs.535011	-1.187243327	5.427466588	6.614709915
236749_at	MNT	AI968443	4335	Hs.626579, Hs.632239	1.187240077	6.448684572	5.261444496
241867_at		BE676407			-1.187113023	5.727376137	6.914489161
210894_s_at	CEP250	BC001433	11190	Hs.443976	-1.18707557	5.035280862	6.222356432
236816_at	C12orf30	BF110370	80018	Hs.530941	-1.1870205	6.559902881	7.746923381
239415_at	MAP9	AW117322	79884	Hs.61271	-1.18609863	5.717412865	6.903511496
233261_at	EBF1	AU145682	1879	Hs.573143	-1.186014341	5.967863617	7.153877959
202368_s_at	TRAM2	AI986461	9697	Hs.520182	-1.184758078	7.853303632	9.038061711
1556131_s_at	FBF1	AK074045	85302	Hs.442609	1.184560994	7.996397037	6.811836043
229389_at	ATG16L2	AA741058	89849	Hs.653186	-1.184418848	5.113123688	6.297542537
230515_at		AF138861			-1.183615377	5.536785598	6.720400975
1555139_a_at	OTUD7B	BC020622	56957	Hs.98322	-1.183509836	5.455561461	6.639071297
233538_s_at	CYBB	AI203028	1536	Hs.292356	-1.183313214	5.790880398	6.974193612
229098_s_at	DDX46	N76126	9879	Hs.406549	-1.182956692	7.93109123	9.114047922
201591_s_at	NISCH	NM_007184	11188	Hs.435290	1.182719066	10.60026522	9.417546158
1554518_at	GSTCD	BC032942	79807	Hs.161429	-1.181895469	5.531468177	6.713363645
232864_s_at	AFF4	N59653	27125	Hs.519313, Hs.664840	-1.181596755	7.698507512	8.880104267
204755_x_at	HLF	M95585	3131	Hs.196952	-1.181343251	4.915929962	6.097273213
211048_s_at	PDIA4	BC006344	9601	Hs.93659	-1.181256286	9.630320968	10.81157725
223134_at	BBX	AI795970	56987	Hs.124366	-1.181190421	9.216096486	10.39728691
236869_at		AI857429			-1.180841159	6.002515069	7.183356228
238644_at	MYSM1	BF511190	114803	Hs.709264	-1.180675251	7.49067548	8.671350731
1553530_a_at	ITGB1	NM_033669	3688	Hs.643813	-1.177746884	10.02077907	11.19852595
243934_at	ODF3B	AW139261	440836	Hs.531314	-1.176358966	5.056449625	6.23280859
239784_at		AA398740			-1.175682184	5.345803849	6.521486034
201752_s_at	ADD3	AI763123	120	Hs.501012	-1.175645518	5.265390396	6.441035914
238092_at		AI277300			1.174928771	6.526182235	5.351253464
203753_at	TCF4	NM_003199	6925	Hs.605153	-1.174596003	9.267779166	10.44237517
210281_s_at	ZMYM2	AL136621	7750	Hs.644041	-1.174065352	6.06336342	7.237428772
238797_at	TRIM11	BF059582	81559	Hs.13543	-1.174024427	6.242184942	7.416209369
209398_at	HIST1H1C	BC002649	3006	Hs.7644	-1.17359981	9.056608294	10.2302081
235421_at	MAP3K8	AV713062	1326	Hs.432453	-1.173349332	6.470630971	7.643980304
207645_s_at	CHD1L	NM_004284	9557	Hs.191164	-1.173108528	6.500061922	7.67317045
228098_s_at	MYLIP	AW292746	29116	Hs.484738	-1.172870325	6.950551076	8.123421401
1555894_s_at	MTSS1L	AA829283	92154	Hs.432387	-1.172866529	7.579656684	8.752523213
233324_at	TRERF1	AK002096	55809	Hs.485392	-1.172739551	5.86211767	7.034857221
210760_x_at	TRIP11	BC002656	9321	Hs.632339	-1.172324699	6.471454891	7.64377959
210477_x_at	MAPK8	U34822	5599	Hs.138211	-1.17214092	5.641476516	6.813617436
229775_s_at		AI480107			-1.172059887	6.843338105	8.015397992
238589_s_at		AW601184			-1.1719212	5.782432637	6.954353836
233819_s_at	RNF160	AK023499	26046	Hs.288773	-1.171812345	9.542989372	10.71480172
221579_s_at	NUDT3	AF062530	11165	Hs.188882	-1.171722452	9.577847723	10.74957017
1561402_at	LOC339894	BC040669	339894	Hs.478050, Hs.634263	1.170809059	7.103608773	5.932799714

212994_at	THOC2	BE543527	57187	Hs.592243	-1.170720822	9.126475281	10.2971961
201445_at	CNN3	NM_001839	1266	Hs.706617	-1.170547404	7.256472918	8.427020323
212384_at	BAT1	AI282485	7919	Hs.254042	-1.170311087	5.717783338	6.888094425
206748_s_at	SPAG9	NM_003971	9043	Hs.463439	-1.17017224	6.841740356	8.011912596
1556319_at	LOC283270	BQ025632	283270	Hs.629770	1.170009719	7.032000849	5.861991129
1555830_s_at	ESYT2	BC001224	57488	Hs.490795	-1.169470927	6.402483796	7.571954723
229178_at	PRTG	AV699825	283659	Hs.130957	-1.169051724	6.964032812	8.133084536
237215_s_at	TFRC	N76327	7037	Hs.529618, Hs.592950	-1.168431376	8.097923099	9.266354476
1566558_x_at	FLJ90757	AK096609	440465	Hs.448889	1.167704884	8.789440804	7.62173592
208442_s_at	ATM	NM_000051	472	Hs.367437	1.16732556	7.466357749	6.299032189
229791_at	LPCAT2	N32611	54947	Hs.460857	-1.167015769	6.763945407	7.930961176
202269_x_at	GBP1	BC002666	2633	Hs.62661	-1.166527534	7.983228979	9.149756513
215177_s_at	ITGA6	AV733308	3655	Hs.133397	-1.166306342	8.362825244	9.529131586
1556630_at	CASC2	AJ344228	255082	Hs.89387	1.1662018	6.846822743	5.680620943
1555450_a_at	NARG1L	BC032318	79612	Hs.512914	-1.166151909	6.268653319	7.434805229
242862_x_at		AI804210			-1.166061899	5.742489462	6.908551362
226492_at	SEMA6D	AL036088	80031	Hs.511265	1.166035297	8.621284308	7.455249011
203413_at	NELL2	NM_006159	4753	Hs.505326	1.165016979	7.611218536	6.446201557
229890_at	PRRT1	AI799702	80863	Hs.549204, Hs.699821	1.164904272	8.2761271	7.111222828
233823_at	FAM184B	AB033102	27146	Hs.18861	-1.164791014	6.966147651	8.130938666
209087_x_at	MCAM	AF089868	4162	Hs.599039	1.164719832	10.42360383	9.258884001
201562_s_at	SORD	NM_003104	6652	Hs.633539, Hs.878	-1.164296133	9.844988481	11.00928461
215092_s_at	NFAT5	AJ005683	10725	Hs.371987	-1.164000592	8.557196417	9.721197008
1569676_at		BC024226			-1.16330666	5.585171822	6.748478482
210916_s_at	CD44	AF098641	960	Hs.502328	-1.162849873	9.540344467	10.70319434
203220_s_at	TLE1	AI951720	7088	Hs.197320, Hs.689805	-1.161626979	5.162336948	6.323963926
209193_at	PIM1	M24779	5292	Hs.81170	-1.161050171	9.079284054	10.24033422
230663_at		AI741025			-1.160903725	7.318451292	8.479355017
219501_at	ENOX1	NM_017993	55068	Hs.128258, Hs.711520	1.160780977	8.881411729	7.720630752
210581_x_at	PATZ1	AF254088	23598	Hs.517557	-1.160749874	4.983116906	6.14386678
228288_at		AA772299			-1.160693687	5.977532407	7.138226094
212926_at	SMC5	AW183677	23137	Hs.534189, Hs.601181	-1.160577692	7.129263903	8.289841595
234681_s_at	CHD6	AK026022	84181	Hs.371979	-1.159752684	5.350089886	6.509842571
227661_at	ANKRD11	BF195623	29123	Hs.335003	-1.159651445	6.104372771	7.264024216
205945_at	IL6R	NM_000565	3570	Hs.709210	-1.15946672	9.514024774	10.67349149
225551_at	C1orf71	AW291187	163882	Hs.368353	-1.159167135	6.098348377	7.257515512
230127_at		AW044663			1.158617937	10.54189092	9.383272984
215248_at	GRB10	AU145003	2887	Hs.164060	-1.158568783	5.677737007	6.83630579
235000_at		T86629			-1.158093066	7.786902598	8.944995664
1553130_at	PRO0461	NM_152340	652276	Hs.459691, Hs.61960	-1.157040177	5.299825108	6.456865285
242776_at	ZCCHC6	AA584428	79670	Hs.655162	-1.156793655	7.417483109	8.574276764
215455_at	TIMELESS	AK000721	8914	Hs.118631	1.156634282	7.1058527	5.949218417
208661_s_at	TTC3	AW510696	7267	Hs.368214	-1.156440801	9.348153168	10.50459397

230533_at	ZMYND8	AF144233	23613	Hs.446240, Hs.658553, Hs.66	-1.156402975	6.077143262	7.233546237
241885_at		BF431050			-1.15635908	6.366522638	7.522881718
206562_s_at	CSNK1A1	NM_001892	1452	Hs.529862, Hs.712555	-1.156355861	10.63267874	11.7890346
224766_at	RPL37	AW008221	6167	Hs.558601, Hs.80545	-1.156039243	8.613309763	9.769349006
1559227_s_at	VHL	BF972755	7428	Hs.517792	-1.155910729	6.412821293	7.568732022
223189_x_at	MLL5	AW082219	55904	Hs.592262	-1.154969118	8.541172773	9.696141891
219492_at	CHIC2	NM_012110	26511	Hs.335393	-1.154620338	9.532991644	10.68761198
216959_x_at	NRCAM	U55258	4897	Hs.21422	-1.154215571	6.767805917	7.922021488
235606_at	LOC344595	AA417117	344595	Hs.543039, Hs.655735	-1.153747875	5.740830273	6.894578149
216226_at	TAF4B	Y09321	6875	Hs.369519	-1.153703074	6.588978277	7.742681351
213665_at	SOX4	AI989477	6659	Hs.643910	-1.653483857	7.0201211	8.673604956
202924_s_at	PLAGL2	AL562280	5326	Hs.154104	1.153310417	8.807130149	7.653819732
1557810_at		BM352108			-1.153264388	4.889067029	6.042331417
201123_s_at	EIF5A	NM_001970	1984	Hs.534314	-1.153251743	9.949386627	11.10263837
244871_s_at	USP32	AW268357	84669	Hs.132868	-1.153068914	6.988739437	8.141808351
202062_s_at	SEL1L	NM_005065	6400	Hs.181300	-1.152248242	7.228843116	8.381091358
234649_at		AK026259			-1.151799007	4.981107267	6.132906275
227844_at	FMNL3	AI089932	91010	Hs.179838	1.151590901	8.206351136	7.054760236
239923_at		AI056872			-1.151577236	5.105399744	6.25697698
202479_s_at	TRIB2	BC002637	28951	Hs.467751	-1.151444269	6.520008055	7.671452324
229004_at		AI970797			1.151166849	6.672616852	5.521450002
236079_at	DKFZp667E05:	AA649070	202025	Hs.618463	1.151161447	7.851788988	6.700627541
210458_s_at	TANK	BC003388	10010	Hs.132257	-1.149697216	7.40630571	8.556002925
212073_at		AI631874			-1.149620774	9.590006069	10.73962684
227016_at	ERICH1	AA767385	157697	Hs.655310	-1.149422321	8.069909944	9.219332264
201741_x_at	SFRS1	M69040	6426	Hs.68714	-1.149417144	6.210344735	7.359761879
214978_s_at	PPFIA4	AK023365	8497	Hs.153648	1.148808971	6.076095437	4.927286466
213650_at		AW006438			-1.14847332	7.71390032	8.86237364
213514_s_at	DIAPH1	AU158818	1729	Hs.529451	-1.148046399	9.846410158	10.99445656
228251_at		BE467577			-1.14695428	6.968387823	8.115342102
227389_x_at	IRF2BP2	AA058858	359948	Hs.693837	-1.146938481	6.122689733	7.269628215
1554249_a_at	ZNF638	BC024000	27332	Hs.434401	-1.146812823	7.64113393	8.787946754
35201_at	HNRNPL	X16135	3191	Hs.644906	-1.146594455	10.56023919	11.70683364
232216_at	YME1L1	AA828049	10730	Hs.499145, Hs.74647	-1.14630936	4.983274136	6.129583496
203141_s_at	AP3B1	AW058575	8546	Hs.532091	-1.145580434	8.0556092	9.201189634
240212_at		AW340175			1.144899172	6.610768788	5.465869616
225238_at	MSI2	BF435123	124540	Hs.658922	-1.144843928	6.921290831	8.066134758
219557_s_at	NRIP3	NM_020645	56675	Hs.523467	-1.144843219	7.579806962	8.724650181
1553679_s_at	VKORC1L1	NM_173517	154807	Hs.427232	-1.144670903	6.133013687	7.27768459
1556375_at		AF086103			-1.144446212	6.347717166	7.492163378
213748_at	TRIM66	AW271713	9866	Hs.130836	-1.143595901	6.660083845	7.803679746
208078_s_at		NM_030751			-1.143466467	9.260559399	10.40402587
211300_s_at	TP53	K03199	7157	Hs.654481			

1554980_a_at	ATF3	AB066566	467	Hs.460	-1.142784199	8.05633703	9.19912123
1553768_a_at	DCBLD1	NM_173674	285761	Hs.658304	-1.142292529	8.475646148	9.617938678
222568_at	UGGT1	AI672492	56886	Hs.598715, Hs.719174	-1.142047616	8.037536602	9.179584218
227946_at	OSBPL7	AI955239	114881	Hs.463320	1.141531609	9.025213059	7.88368145
222540_s_at	RSF1	BG286920	51773	Hs.420229	-1.140651611	9.121551772	10.26220338
1567105_at		AF362887			-1.14051606	9.829839277	10.97035534
235693_at		D81004			-1.139945225	7.153266995	8.293212221
225219_at	SMAD5	BF526175	4090	Hs.167700	-1.139467854	9.903100364	11.04256822
226819_at		AW206435			-1.139067048	6.829578982	7.968646031
41386_i_at	KDM6B	AB002344	23135	Hs.223678	-1.138803404	9.91370854	11.05251194
1556369_a_at	PHKG2	CA449954	5261	Hs.196177	1.138796172	7.456764787	6.317968615
204427_s_at	TMED2	NM_006815	10959	Hs.592682, Hs.75914	-1.138238011	9.212481593	10.3507196
231643_s_at	CMIP	BE045541	80790	Hs.709248	-1.137967303	7.587625753	8.725593056
221221_s_at	KLHL3	NM_017415	26249	Hs.655084	1.137721942	7.503638729	6.365916786
41037_at	TEAD4	U63824	7004	Hs.94865	1.137603296	11.9117993	10.774196
213875_x_at	C6orf62	BG252842	81688	Hs.519930	-1.137555346	10.0933771	11.23093245
238937_at	ZNF420	AI339586	147923	Hs.444992	-1.137003132	5.841441112	6.978444244
236442_at	DPF3	BF672019	8110	Hs.162868	1.137000722	6.011036696	4.874035975
230879_at	BAG2	AI654091	9532	Hs.719303	-1.136997527	6.005796404	7.14279393
217465_at	NCKAP1	AK001291	10787	Hs.603732	-1.136932714	9.161887382	10.2988201
229994_at	NFIA	R45950	4774	Hs.594180, Hs.710546	-1.136232504	6.180720211	7.316952714
232555_at	CREB5	AI689210	9586	Hs.437075	-1.136147837	5.889436923	7.025584761
1554629_at	EPHA7	BC027940	2045	Hs.73962	1.136051424	6.037444428	4.901393003
210001_s_at	SOCS1	AB005043	8651	Hs.50640	-1.13590775	7.236925427	8.372833177
1558569_at	UNQ6228	AL832308	100131541	Hs.661972	-1.134645678	6.531105528	7.665751206
205369_x_at	DBT	J03208	1629	Hs.709187	-1.134343047	5.542124597	6.676467645
204324_s_at	GOLIM4	NM_014498	27333	Hs.143600	-1.134296581	8.006237861	9.140534442
236452_at		BE219380			1.133954586	8.539035043	7.405080457
1565689_at		BG400570			-1.133746245	5.586469947	6.720216192
228173_at	GNAS	AA810695	2778	Hs.125898, Hs.694849	-1.133520972	6.72321671	7.856737683
1558732_at	MAP4K4	AK074900	9448	Hs.719073	-1.133505191	6.736603028	7.870108219
210012_s_at	EWSR1	BC000527	2130	Hs.374477	-1.133495693	5.794888094	6.928383787
215682_at	LOC440792	AB051440	440792	Hs.613849	1.132986194	6.197653925	5.064667732
230580_at		AI222805			1.132564755	8.335679789	7.203115034
210051_at	RAPGEF3	U78168	10411	Hs.8578	1.132216774	6.740095156	5.607878383
227992_s_at	NCRNA00085	AA725913	147650	Hs.467174	1.132002647	6.7128045	5.580801852
1557050_at		CA448125			1.131898222	7.712046871	6.580148649
206114_at	EPHA4	NM_004438	2043	Hs.371218	-1.131694789	7.228948913	8.360643702
205921_s_at	SLC6A6	U16120	6533	Hs.529488	-1.131169302	7.409505839	8.540675141
241546_at	SPATA5	BE550289	166378	Hs.709648	-1.131028864	5.906641963	7.037670826
230256_at	C1orf104	AW009436	284618	Hs.650801, Hs.708354	-1.130467775	8.433539931	9.564007706
241997_at		AA700817			-1.130431866	5.85394444	6.984376306
206897_at	PAGE1	NM_003785	8712	Hs.128231	1.130132907	6.133013687	5.00288078

243509_at		AI475680			-1.12976706	5.915903115	7.045670175
1552274_at	PXK	BC014479	54899	Hs.190544	-1.12962661	7.841844901	8.971471511
1554690_a_at	TACC1	BC041391	6867	Hs.279245	-1.129385015	6.763945407	7.893330422
227543_at	RNASEH2C	AI990526	84153	Hs.718438	1.128211336	9.051787571	7.923576235
1554489_a_at	CEP70	BC016050	80321	Hs.531962	-1.128133931	8.087279621	9.215413551
220940_at	ANKRD36B	NM_025190	57730	Hs.532921	-1.127646364	8.144659773	9.272306137
217482_at		AK021987			-1.12763544	6.710439581	7.838075021
1569104_a_at		BE646227			1.12717116	7.231390103	6.104218942
229946_at	FAM168B	BF056651	130074	Hs.534679	-1.127153287	7.02878615	8.155939437
218621_at	HEMK1	NM_016173	51409	Hs.517987, Hs.599362	1.127082911	8.980495985	7.853413075
229675_at	MINA	AA772075	84864	Hs.607776	-1.125761049	7.170307375	8.296068423
240452_at	GSPT1	AA580082	2935	Hs.528780	-1.125020556	6.465173881	7.590194438
241174_at	AP4E1	AV647279	23431	Hs.413366	-1.124638448	5.800571601	6.925210049
237310_at		AI743607			-1.124154217	6.849835089	7.973989307
212423_at	ZCCHC24	AK024784	219654	Hs.523080	-1.124148873	6.778719864	7.902868737
243947_s_at		AW300612			1.124081647	8.358039712	7.233958065
235388_at	CHD9	BG538482	80205	Hs.59159, Hs.622347	-1.124057565	6.383368415	7.50742598
228392_at	ZNF302	BF508739	55900	Hs.436350	-1.123902548	5.176272549	6.300175097
216855_s_at	HNRNPU	D13413	3192	Hs.106212	-1.123176634	7.583653897	8.706830531
215533_s_at	UBE4B	AF091093	10277	Hs.632370	-1.12312556	8.839118331	9.962243891
230666_at	HOXA11AS	AA622837	221883	Hs.587427	1.122654641	6.319581101	5.19692646
208299_at	CACNA1I	NM_021096	8911	Hs.125116	1.122491039	6.263919567	5.141428528
223190_s_at	MLL5	AF067804	55904	Hs.592262	-1.121994727	9.763271702	10.88526643
222633_at	TBL1XR1	AF268193	79718	Hs.715537	-1.121927476	10.06428533	11.18621281
205405_at	SEMA5A	NM_003966	9037	Hs.27621	-1.121919383	5.972110562	7.094029945
224620_at	MAPK1	AL157438	5594	Hs.431850	1.121688954	6.698811649	5.577122694
1558173_a_at	LUZP1	AK093016	7798	Hs.257900, Hs.654306	-1.121601723	10.39964843	11.52125015
216023_at	KDM4B	AK026040	23030	Hs.654816	1.12152018	6.364900723	5.243380543
239544_at		BE671251			1.121380896	6.56876052	5.447379624
224335_s_at	BACE1	AB050436	23621	Hs.504003	-1.121347693	5.973552286	7.09489998
223129_x_at	MYLIP	T63512	29116	Hs.484738	-1.12113962	7.84034926	8.96148888
233445_at		AK022040			-1.12069458	6.602590633	7.723285214
231978_at	TPCN2	AL137479	219931	Hs.131851	1.120587367	6.212971522	5.092384155
217051_s_at	SS18	AF257501	6760	Hs.404263	-1.120499868	5.703503256	6.824003124
219001_s_at	DCAF10	NM_024345	79269	Hs.118394	-1.120095396	7.841988137	8.962083532
214780_s_at	MYO9B	AK002201	4650	Hs.123198	1.120057861	10.30493746	9.184879597
1555448_at	MUDENG	BC013174	55745	Hs.597349	-1.119159116	5.93564107	7.054800186
204543_at	RAPGEF1	NM_005312	2889	Hs.127897	-1.118619044	6.638541358	7.757160402
219330_at	VANGL1	NM_024062	81839	Hs.515130	-1.118374382	9.238717828	10.35709221
232476_at		AK025105			-1.116669833	5.086756568	6.203426401
210971_s_at	ARNTL	AB000815	406	Hs.65734	-1.116629987	8.116210257	9.232840244
235792_x_at	PIK3C2A	AU154663	5286	Hs.175343	-1.116442706	8.095706301	9.212149007
213089_at	LOC10027221(	AU158490	100272216	Hs.631974	-1.11644126	6.159871287	7.276312547

213596_at	CASP4	AL050391	837	Hs.138378	1.116374161	7.508283127	6.391908966
207100_s_at	VAMP1	NM_016830	6843	Hs.20021	1.116023271	8.005461624	6.889438353
1553668_at	LRCH3	NM_032773	84859	Hs.518414, Hs.659335	-1.11543427	5.312662236	6.428096505
206113_s_at	RAB5A	NM_004162	5868	Hs.475663	-1.115205506	8.348184074	9.46338958
204577_s_at	CLUAP1	NM_024793	23059	Hs.155995	-1.114931301	7.201045669	8.31597697
212019_at	RSL1D1	AK025446	26156	Hs.401842	-1.114516335	5.198614855	6.31313119
229293_at		AI869532			-1.114154526	6.787069699	7.901224224
209459_s_at	ABAT	AF237813	18	Hs.336768, Hs.715664	1.113421163	7.753278617	6.639857454
208584_at	SNCG	NM_016432	6623	Hs.349470	1.11291753	6.258218625	5.145301096
212188_at	KCTD12	AA551075	115207	Hs.644125	-1.112668045	9.179854595	10.29252264
215236_s_at	PICALM	AV721177	8301	Hs.163893	-1.112549831	7.473774624	8.586324455
235454_at		AI436561			-1.112471198	6.022107041	7.134578239
230838_s_at	CIAPIN1	AW771492	57019	Hs.4900	-1.110807286	6.529186568	7.639993854
239469_at		BF513404			-1.110476558	6.107161463	7.217638021
237330_at		AA603494			-1.110225782	6.491734723	7.601960506
214367_at	RASGRP2	AI688812	10235	Hs.99491	1.109981295	6.06921853	4.959237235
212362_at	ATP2A2	AA805753	488	Hs.506759	-1.109784691	6.531389112	7.641173803
244554_at		AI351179			-1.109049756	5.149571958	6.258621714
236853_at	C13orf16	AW665078	121793	Hs.210677	1.109028146	6.520870466	5.41184232
204281_at	TEAD4	NM_003213	7004	Hs.94865	1.107936166	12.2774153	11.16947914
228478_at		AA889954			-1.107928072	8.516701139	9.624629211
226474_at	NLRC5	AA005023	84166	Hs.528836	1.107020834	10.11188118	9.004860347
238517_at		BF815640			-1.10653717	8.239583849	9.34612102
238487_at	GNL1	BE166476	2794	Hs.83147	-1.106090056	5.849643183	6.955733239
216531_at	YY2	U73479	404281	Hs.443490, Hs.673601	-1.105392742	4.971024847	6.076417588
204863_s_at	IL6ST	BE856546	3572	Hs.532082	-1.104929237	9.511862183	10.61679142
227478_at	SETBP1	BF739885	26040	Hs.435458	1.104925976	9.41619897	8.311272993
1554451_s_at	DNAJC14	AF141342	85406	Hs.709320	-1.10377359	7.480005863	8.583779453
1554433_a_at	ZNF146	BC005154	7705	Hs.643436	-1.103480507	9.395880621	10.49936113
214900_at	ZKSCAN1	AC004522	7586	Hs.615360	-1.102826415	6.967525774	8.070352189
1560599_a_at	CCDC123	BC020195	84902	Hs.599703	-1.102502652	8.207892064	9.310394715
219435_at	C17orf68	NM_025099	80169	Hs.156055	1.102474151	7.828308999	6.725834848
241606_s_at	TRUB1	BE736287	142940	Hs.21187	-1.102380006	6.818817736	7.921197742
207950_s_at	ANK3	NM_001149	288	Hs.499725	-1.102336848	7.111390515	8.213727363
213351_s_at	TMCC1	AB018322	23023	Hs.477547, Hs.709936	-1.102176603	5.681154476	6.783331079
243559_at		BF515306			-1.102000329	5.206730615	6.308730944
240246_at		AW294722			-1.101533377	5.439191932	6.540725309
210828_s_at	ARNT	AF001307	405	Hs.632446	-1.101517861	5.815490939	6.9170088
214600_at	TEAD1	AW771935	7003	Hs.655331	-1.101472135	8.981204736	10.08267687
219691_at	SAMD9	NM_017654	54809	Hs.65641	1.100988335	9.303811656	8.202823322
239976_at	ACAD9	AW182960	28976	Hs.567482	1.100401688	6.130208055	5.029806367
1558740_s_at		R30807			1.099601765	10.13503402	9.035432253
232601_at		AL353951			-1.099038187	6.001987568	7.101025755

206073_at	COLQ	AF057036	8292	Hs.146735	1.098983139	6.665248531	5.566265392
1559384_at		BG200365			-1.098477986	5.755067174	6.85354516
1560006_a_at	LOC646762	BG501482	646762	Hs.30579	-1.098184897	6.692340879	7.790525776
211599_x_at	MET	U19348	4233	Hs.132966	-1.09747271	5.911399081	7.008871792
207001_x_at	TSC22D3	NM_004089	1831	Hs.716410	-1.09727254	6.798348059	7.895620599
213185_at	KIAA0556	AI758896	23247	Hs.460459	1.09712862	8.797165002	7.700036382
205808_at	ASPH	NM_004318	444	Hs.591874	-1.097035487	6.875240402	7.97227589
205196_s_at	AP1S1	NM_001283	1174	Hs.489365, Hs.718546	-1.096944635	8.853166119	9.950110754
1557300_s_at		AI741292			-1.096137097	7.990488545	9.086625642
209346_s_at	PI4K2A	BC003167	55361	Hs.25300	-1.095936701	7.721884406	8.817821107
206877_at	MXD1	NM_002357	4084	Hs.468908	-1.095496562	6.52621936	7.621715922
209385_s_at	PROSC	AL136616	11212	Hs.304792, Hs.608177	-1.095395542	8.774954589	9.870350131
216283_s_at	PVR	X64116	5817	Hs.171844	-1.095341598	6.431415167	7.526756764
203568_s_at	TRIM38	NM_006355	10475	Hs.584851	-1.095190352	6.319049866	7.414240218
236020_s_at	TRUB1	BF116232	142940	Hs.21187	-1.094667823	5.277708818	6.37237664
239540_at		AI671903			-1.094514428	5.216319062	6.31083349
1559469_s_at	SIPA1L2	BC006013	57568	Hs.715656	-1.094254721	5.202641387	6.296896108
225009_at	CMTM4	AA191708	146223	Hs.643961	-1.09402093	8.467231848	9.561252778
1552760_at	HDAC9	BM726008	9734	Hs.196054	-1.093872787	6.317234664	7.411107452
225656_at	EFHC1	AI564473	114327	Hs.403171	-1.093760582	6.059782256	7.153542838
222302_at		BE813017			1.092982428	7.306887289	6.213904861
242812_at	TRIM26	AW294604	7726	Hs.485041	-1.092356092	7.356160326	8.448516418
222570_at	FREQ	AA045247	23413	Hs.642946, Hs.714951	-1.091906135	10.20880628	11.30071242
222562_s_at	TNKS2	BF060683	80351	Hs.329327	-1.091518026	7.788561154	8.880079181
226367_at	KDM5A	AA854032	5927	Hs.76272	-1.091349787	6.735818546	7.827168333
231167_at		AW444881			-1.090906876	7.757589431	8.848496307
232372_at		AL157491			-1.089998143	5.282378497	6.37237664
230270_at	PRPF38B	N32872	55119	Hs.342307	-1.089977884	7.620990109	8.710967993
211448_s_at	RGS6	AF107619	9628	Hs.509872	1.089885363	6.580525269	5.490639907
235646_at		BF515595			-1.089555623	5.703855042	6.793410665
216062_at		AW851559			-1.089213609	5.099045441	6.18825905
203795_s_at	BCL7A	NM_020993	605	Hs.530970	-1.088336368	7.553463117	8.641799485
1554660_a_at	C1orf71	BC036200	163882	Hs.368353	-1.088062932	5.819719122	6.907782054
227858_at	PCNXL3	AI379451	399909	Hs.380801	-1.087966143	7.649570311	8.737536453
226470_at	GGT7	AL049709	2686	Hs.433738	1.087219366	7.110046694	6.022827328
211944_at	BAT2D1	BE729523	23215	Hs.494614	-1.086679786	10.3875885	11.47426828
1554481_a_at	EPB41	BC039079	2035	Hs.175437, Hs.708933	-1.0866319	7.075120595	8.161752495
1552507_at	KCNE4	NM_080671	23704	Hs.348522	-1.08635189	5.526433464	6.612785354
217100_s_at	UBXN7	AK026451	26043	Hs.518524	-1.086265959	9.634416352	10.72068231
222062_at	IL27RA	AI983115	9466	Hs.132781	1.086018546	8.974037479	7.888018933
230060_at	CDCA7	AI277642	83879	Hs.470654	-1.085843024	6.654688228	7.740531252
1555611_s_at	MBD1	BC012487	4152	Hs.405610	-1.085192631	5.587055327	6.672247958
221830_at	RAP2A	AI302106	5911	Hs.508480	-1.084421823	8.754015385	9.838437208

242316_at		AI810103			1.084372542	6.123735231	5.039362689
202838_at	FUCA1	NM_000147	2517	Hs.370858	1.084233515	9.200550851	8.116317336
207031_at	NKX3-2	NM_001189	579	Hs.590927	1.083862365	6.175840173	5.091977808
238368_at		AW271932			-1.08320377	6.725834848	7.809038618
213774_s_at	PPP1R2	AW614578	5504	Hs.535731, Hs.706920	-1.08261368	8.504541768	9.587155448
204117_at	PREP	NM_002726	5550	Hs.436564	-1.082529057	10.08514498	11.16767403
241349_at		BF854663			-1.082477108	5.441876712	6.52435382
230779_at	TNRC6B	BF594371	23112	Hs.372082	-1.082387197	9.304877494	10.38726469
223535_at	NUDT12	AL136592	83594	Hs.434289	1.082349572	8.097196776	7.014847204
1570151_at		BC013779			-1.08220191	5.377718211	6.45992012
209446_s_at	C7orf44	BC001743	55744	Hs.654779, Hs.655165, Hs.65	-1.081951542	7.858483956	8.940435499
219968_at	ZNF589	NM_016089	51385	Hs.172602	1.080991437	9.588953173	8.507961736
213319_s_at	CSDA	AW170359	8531	Hs.221889	-1.080728477	8.179641394	9.260369872
240282_at	WDR1	AW770902	9948	Hs.128548, Hs.713658	-1.080540163	7.016768021	8.097308184
202834_at	AGT	NM_000029	183	Hs.19383	-1.079694366	6.720121912	7.799816278
237317_at		AW136338			-1.078615002	5.85599133	6.934606332
229848_at	ZNF10	W72653	7556	Hs.507355	1.078598314	7.398229802	6.319631489
1553787_at	C11orf45	NM_145013	219833	Hs.351133	1.078585546	8.157325662	7.078740116
210654_at	TNFRSF10D	AF021233	8793	Hs.213467	-1.07836386	5.658784859	6.737148719
225116_at	HIPK2	AW300045	28996	Hs.397465, Hs.632033	-1.078133419	9.897604855	10.97573827
204732_s_at	TRIM23	AI021991	373	Hs.792	-1.077968016	6.319999233	7.397967249
239219_at	AURKB	N55457	9212	Hs.442658	-1.077944062	5.52262918	6.600573242
225488_at	DERL1	AI967978	79139	Hs.241576	1.077087465	6.378190947	5.301103482
234153_at		AK024662			-1.076741516	6.300984803	7.377726318
213487_at	MAP2K2	AI762811	5605	Hs.465627	-1.076621629	6.544119258	7.620740887
243171_at	LOC10012832!	AA570178	100128325	Hs.105944	1.076419961	6.667086518	5.590666558
225584_at		BE880820			-1.076312974	7.858400698	8.934713672
243801_x_at		AA971709			-1.076113137	7.289539483	8.36565262
217066_s_at	DMPK	M87313	1760	Hs.631596	1.074938994	8.599722823	7.524783829
233690_at		AK026743			-1.073998274	5.918975238	6.992973513
204188_s_at	RARG	M57707	5916	Hs.1497	-1.073737084	5.405718968	6.479456052
1557889_at		BC006326			-1.073605913	5.405712676	6.479318589
231321_s_at	ACER3	AI681372	55331	Hs.23862	-1.073549842	7.79436514	8.867914982
242208_at	ZNF37B	AI634543	100129482	Hs.646695, Hs.648656, Hs.65	-1.072925259	6.596324273	7.669249532
232050_at		AU151146			-1.072699422	6.446929219	7.519628641
226874_at	KLHL8	BF591270	57563	Hs.106601, Hs.715202	-1.072628777	8.467027002	9.539655779
1553107_s_at		BF436799			-1.072495703	8.282172689	9.354668393
228408_s_at	SDAD1	AI738666	55153	Hs.632604	-1.072217508	10.78306161	11.85527912
239071_at	RBBP4	AI972451	5928	Hs.16003	-1.071805519	6.912580784	7.984386303
1556195_a_at		BC033316			1.0717544	7.142754922	6.071000522
228771_at	ADRBK2	AI651212	157	Hs.657494	1.07167059	8.406347421	7.33467683
214177_s_at	PBXIP1	AI935162	57326	Hs.505806	1.071340119	8.916915099	7.84557498
1554217_a_at	CCDC132	BC017888	55610	Hs.222282	-1.071196789	7.283901918	8.355098707

223194_s_at	SLC22A23	AL512737	63027	Hs.713588	1.070578005	9.087551558	8.016973553
212576_at	MGRN1	AB011116	23295	Hs.526494	-1.070233246	9.664276165	10.73450941
1556389_at	CNPY3	AF161347	10695	Hs.414099	1.070161827	7.02893795	5.958776123
211786_at	TNFRSF9	BC006196	3604	Hs.654459	1.070099516	7.069616146	5.999516629
241788_x_at		AW168912			-1.069587539	5.454218627	6.523806166
243456_at	ZNF214	AW182291	7761	Hs.445849	1.069549991	7.028055677	5.958505685
215905_s_at	SNRNP40	AL157420	9410	Hs.33962	-1.069127691	10.72708108	11.79620877
224992_s_at	CMIP	AI363061	80790	Hs.709248	-1.068227552	7.39603313	8.464260682
226407_at		BE549656			-1.067910741	8.976841534	10.04475227
208129_x_at	RUNX1	NM_001754	861	Hs.149261, Hs.612648	1.067733923	6.624561706	5.556827782
237127_at		AA142959			-1.06749871	5.291089937	6.358588648
230140_at	PTPN9	AI742739	5780	Hs.445775	-1.067335885	5.062247611	6.129583496
201008_s_at	TXNIP	AA812232	10628	Hs.533977, Hs.715525	-1.067206822	11.29366691	12.36087374
226368_at	CHST11	AI806905	50515	Hs.17569	-1.067046476	8.298538996	9.365585472
37950_at	PREP	X74496	5550	Hs.436564	-1.066586826	9.766123366	10.83271019
214929_s_at	KIAA1109	AL137384	84162	Hs.408142	-1.066471848	5.73890927	6.805381119
1554844_at	EYA3	BC041667	2140	Hs.185774	1.066452597	7.028055677	5.96160308
203181_x_at	SRPK2	AW149364	6733	Hs.285197	-1.066040752	10.12019191	11.18623266
203681_at	IVD	M34192	3712	Hs.513646	-1.064515093	5.918632894	6.983147988
207824_s_at	MAZ	NM_002383	4150	Hs.23650	-1.064493312	6.336501707	7.400995019
211826_s_at	AFF1	L22179	4299	Hs.480190	-1.064062243	5.544998819	6.609061063
231793_s_at	CAMK2D	AA448956	817	Hs.144114	-1.063371347	5.942160683	7.00553203
213167_s_at		BF982927			-1.063334674	5.519838835	6.583173509
238050_at	ANTXR2	R94785	118429	Hs.162963	1.06267109	8.141411206	7.078740116
232164_s_at	EPPK1	AL137725	83481	Hs.200412	1.06260544	6.699463322	5.636857882
226843_s_at	PAPD5	AU146704	64282	Hs.514342	-1.062541855	9.163408416	10.22595027
1555261_at		AL832319			-1.062358351	5.714801036	6.777159387
204147_s_at	TFDP1	NM_007111	7027	Hs.79353	-1.061964479	8.745861079	9.807825559
232122_s_at	VEPH1	AK022666	79674	Hs.658046	1.061344099	7.224863663	6.163519564
238761_at		BE645241			-1.061292494	7.97493772	9.036230213
230878_s_at	COG1	BF510252	9382	Hs.283109	-1.06127818	5.202641387	6.263919567
243487_at	AFF4	AV652437	27125	Hs.519313, Hs.664840	-1.061025187	6.957827673	8.01885286
1556049_at	RTN4	CA428769	57142	Hs.704007	-1.059980933	5.976566318	7.036547252
205195_at	AP1S1	NM_001283	1174	Hs.489365, Hs.718546	-1.059717876	9.733135342	10.79285322
242071_x_at	ITGA8	BF446919	8516	Hs.171311	1.059665285	6.507044909	5.447379624
223916_s_at	BCOR	AF317392	54880	Hs.659681	-1.059346869	7.184684891	8.24403176
204633_s_at	RPS6KA5	AF074393	9252	Hs.510225	-1.058992339	6.109167754	7.168160093
215329_s_at		AL031282			-1.058451313	7.802749283	8.861200596
222457_s_at	LIMA1	BC001247	51474	Hs.525419	-1.058363456	10.62538219	11.68374565
222055_at		AA723370			1.058243213	7.383419967	6.325176754
229095_s_at		AI797263			1.058002316	10.1484648	9.090462487
37996_s_at	DMPK	L08835	1760	Hs.631596	1.05799136	9.8760688	8.81807744
228528_at		AI927692			1.057752648	7.215323087	6.157570439

210062_s_at	ZNF589	AF114817	51385	Hs.172602	1.057135271	9.449690131	8.39255486
228834_at	TOB1	BF240286	10140	Hs.709952, Hs.714409, Hs.71	-1.056984323	9.382509464	10.43949379
1565705_x_at		AK025048			1.056618609	7.213919758	6.157301148
235266_at	ATAD2	AI139629	29028	Hs.370834	-1.055921373	9.207035822	10.2629572
221011_s_at	LBH	NM_030915	81606	Hs.567598, Hs.593113	-1.055114232	8.174725647	9.229839879
217595_at	GSPT1	AV701723	2935	Hs.528780	-1.054725407	5.300802662	6.355528069
236728_at	LNPEP	AW070437	4012	Hs.527199, Hs.656905	1.054466478	6.351262908	5.29679643
232485_at	RUNDC2A	AK022425	84127	Hs.458401	-1.054429087	6.058967424	7.113396511
241457_at		AI821935			-1.053603425	5.118904193	6.172507618
243046_at		BF679700			-1.053272274	5.697155221	6.750427495
210655_s_at		AF041336			-1.053126481	7.877550724	8.930677205
215501_s_at	DUSP10	AK022513	11221	Hs.497822	-1.052896056	9.282712106	10.33560816
201635_s_at	FXR1	AI990766	8087	Hs.478407	-1.052784195	9.167273083	10.22005728
242767_at	LMCD1	N95437	29995	Hs.475353	1.052748263	7.462712303	6.409964041
223661_at	NUCKS1	AF130080	64710	Hs.213061, Hs.652429	-1.052375643	10.29348244	11.34585808
232527_at		AU146179			-1.052185969	5.660661583	6.712847552
206532_at	SMARCB1	NM_003073	6598	Hs.534350	-1.051820061	8.406140212	9.457960274
204666_s_at	SIKE1	NM_025073	80143	Hs.709277	-1.051659831	7.094765642	8.146425473
205872_x_at	PDE4DIP	NM_022359	9659	Hs.584841, Hs.613082, Hs.65	-1.051461499	7.635514068	8.686975567
235551_at	WDR4	AA555280	10785	Hs.248815	-1.051010507	6.239048831	7.290059338
1557073_s_at	TTBK2	AK074481	146057	Hs.659846, Hs.713940	-1.05071238	6.869075213	7.919787593
221553_at	MAGT1	AL136636	84061	Hs.323562, Hs.710189	-1.049823574	7.778110253	8.827933826
230803_s_at	ARHGAP24	AI761947	83478	Hs.444229	-1.049463602	6.924717409	7.974181012
224760_at	SP1	AI795991	6667	Hs.620754, Hs.649191	-1.049414317	8.967103649	10.01651797
223557_s_at	TMEFF2	AB017269	23671	Hs.144513	1.048889773	9.10790761	8.059017837
1555122_at	GPR125	BC026009	166647	Hs.99195	-1.048704528	6.056658512	7.105363039
219969_at	CXorf15	NM_018360	55787	Hs.555961	-1.048336574	9.257380037	10.30571661
207524_at	ST7	NM_021908	7982	Hs.368131	1.048158008	9.169947905	8.121789897
210994_x_at	TRIM23	AF230398	373	Hs.792	-1.047689439	7.487058776	8.534748214
220656_at	NARG1L	NM_018527	79612	Hs.512914	-1.047232178	5.801969658	6.849201836
203788_s_at	SEMA3C	AI962897	10512	Hs.269109	-1.047147016	7.657379884	8.7045269
225524_at	ANTXR2	AU152178	118429	Hs.162963	1.047116403	12.70710391	11.6599875
223946_at	MED23	AL136776	9439	Hs.29679	1.045968538	7.348054343	6.302085805
237315_at		AI683864			1.045620835	6.251350246	5.20572941
212520_s_at	SMARCA4	AI684141	6597	Hs.327527	-1.04561096	9.339880148	10.38549111
216320_x_at	MST1	U37055	4485	Hs.349110, Hs.512587	1.045600397	8.556190394	7.510589997
209713_s_at	SLC35D1	AB044343	23169	Hs.213642	1.045202119	7.709739228	6.664537109
201914_s_at	SEC63	AK001465	11231	Hs.26904	-1.044945692	10.61824795	11.66319364
238651_at		BF512491			-1.044841803	7.122323859	8.167165662
229984_at	DTWD1	AI971519	56986	Hs.127432	-1.044740489	6.153169784	7.197910273
231419_at		AI028737			1.04454399	8.540439863	7.495895873
229998_x_at	FAM176B	AI304355	55194	Hs.87016	1.044491723	6.080496408	5.036004684
206082_at	HCP5	NM_006674	10866	Hs.654480	1.043990739	6.546590126	5.502599387

215068_s_at	FBXL18	BC004228	80028	Hs.623974, Hs.706587	-1.043808988	7.276952433	8.320761421
215869_at		AK022254			1.043746451	6.232288353	5.188541902
202308_at	SREBF1	NM_004176	6720	Hs.592123	1.043737149	8.163653078	7.119915929
220918_at	C21orf96	NM_025143	80215	Hs.672131	-1.04360599	5.656815631	6.700421621
222054_at	LOC728448	BF511556	728448	Hs.472508, Hs.710761	1.043070234	8.216625202	7.173554968
225968_at	PRICKLE2	BG285881	166336	Hs.699317	1.041651065	9.559516796	8.517865731
209889_at	SEC31B	AF274863	25956	Hs.18889	1.041603408	8.591873923	7.550270514
222687_s_at	ACER3	R12678	55331	Hs.23862	-1.041362666	8.71991833	9.761280996
204036_at	LPAR1	AW269335	1902	Hs.126667	1.041033762	10.49753274	9.456498977
225137_at		BF111111			-1.040952968	8.058552695	9.099505663
211165_x_at	EPHB2	D31661	2048	Hs.523329	-1.040896985	6.63645036	7.677347345
1566171_at	RFFL	AK092922	117584	Hs.13680	-1.040259104	4.992639579	6.032898684
1557409_at		CA313226			-1.040191257	5.951257438	6.991448695
201747_s_at	SAFB	AI769566	6294	Hs.23978	-1.039898767	7.928334704	8.968233471
201043_s_at	ANP32A	NM_006305	8125	Hs.458747	-1.039744746	11.12745747	12.16720222
210671_x_at	MAPK8	U35004	5599	Hs.138211	-1.039719263	5.797106482	6.836825745
201593_s_at	ZC3H15	AV716798	55854	Hs.368598	-1.039599765	11.12550419	12.16510395
201775_s_at	KIAA0494	AA676790	9813	Hs.719205	-1.039544381	8.932408805	9.971953187
206569_at	IL24	NM_006850	11009	Hs.58831, Hs.719320	1.03937498	8.295341521	7.255966541
1554825_at		BC033184			-1.039310488	7.481515983	8.52082647
240247_at		AI653240			-1.038345411	7.335617501	8.373962912
208583_x_at	HIST1H2AJ	NM_021066	8331	Hs.406691	-1.038120389	6.980584878	8.018705267
244695_at	GHRLOS	AW138672	100126793	Hs.166924, Hs.698339	-1.037675082	6.046640686	7.084315768
219864_s_at	RCAN3	NM_013441	11123	Hs.656799	-1.036544327	6.37161451	7.408158837
211899_s_at	TRAF4	AF082185	9618	Hs.8375	-1.036527148	5.931034484	6.967561632
233152_x_at		AL049979			-1.03650833	5.921354698	6.957863028
244637_at		AA302745			-1.03641262	5.303098558	6.339511177
226576_at	ARHGAP26	AI768563	23092	Hs.654668	1.036382767	7.126818558	6.090435791
204786_s_at	IFNAR2	L41944	3455	Hs.708195	-1.036322795	8.485587739	9.521910535
223185_s_at	BHLHE41	AI819798	79365	Hs.177841	-1.035969846	5.177394715	6.21336456
1558373_s_at		T10213			-1.035952634	5.865077631	6.901030265
209770_at	BTN3A1	U90552	11119	Hs.191510, Hs.628564	1.035869413	8.646877952	7.61100854
209579_s_at	MBD4	AL556619	8930	Hs.35947	-1.035775867	10.45661212	11.49238799
235417_at	SPOCD1	BF689253	90853	Hs.62604	1.035752827	9.448316376	8.412563549
206675_s_at	SKIL	NM_005414	6498	Hs.581632	-1.03567056	7.448372802	8.484043362
1555358_a_at	ENTPD4	BC034477	9583	Hs.444389	-1.035552912	4.998977794	6.034530705
204285_s_at	PMAIP1	AI857639	5366	Hs.96	-1.03549384	9.410464042	10.44595788
238594_x_at	DUSP8	AI864441	1850	Hs.41688	1.035444698	7.370778511	6.335333813
220337_at	NGB	NM_021257	58157	Hs.274363	1.035185001	6.370905039	5.335720038
1559307_s_at	RBL1	BG387892	5933	Hs.207745	-1.034501622	5.003860992	6.038362614
241898_at		AA991267			1.034367527	7.715293559	6.680926033
223788_at		AF116627			1.034231491	6.501282045	5.467050554
1559051_s_at	C6orf150	AK097148	115004	Hs.658405	-1.033789965	6.318005066	7.351795031

229584_at	LRRK2	AK026776	120892	Hs.187636	1.033169168	8.580751333	7.547582165
222880_at	AKT3	AF135794	10000	Hs.498292	-2.032246946	5.55852434	8.590771286
226568_at	FAM102B	AI478747	284611	Hs.200230	-1.03222448	8.758823513	9.791047994
220243_at	ZBTB44	NM_014155	29068	Hs.719099	-1.032091225	4.981447979	6.013539204
244208_at		T80844			-1.031460996	5.530385881	6.561846877
231577_s_at	GBP1	AW014593	2633	Hs.62661	-1.03145909	7.824408971	8.855868062
218821_at	NPEPL1	AL139349	79716	Hs.554211, Hs.654868	1.031291474	8.605529669	7.574238195
214798_at	ATP2C2	AW291664	9914	Hs.6168	1.030939934	7.137012038	6.106072104
215064_at	SC5DL	AK027246	6309	Hs.287749	-1.030068697	6.72034743	7.750416127
232000_at	TTC39B	AW001030	158219	Hs.563630	1.029981938	6.177371739	5.147389802
243446_at	JUB	BE466527	84962	Hs.655832	1.029362329	6.237751789	5.20838946
231761_at	FFAR1	NM_005303	2864	Hs.248127	1.029165098	6.55886517	5.529700072
207453_s_at	DNAJB5	NM_012266	25822	Hs.237506	-1.028736014	5.269362966	6.29809898
226079_at	FLYWCH2	AL542248	114984	Hs.534525	-1.028670308	7.259712027	8.288382336
228298_at	FAM113B	BF056901	91523	Hs.560100	1.028318751	6.982894058	5.954575307
231343_at		AW300131			1.027705072	8.683333732	7.655628661
232207_at	GUSBL2	AK026691	375513	Hs.561539, Hs.711053	1.027641533	6.254540162	5.226898629
236505_at	NUP62	AI807145	23636	Hs.574492	-1.027636213	5.278251725	6.305887937
209223_at		BF434335			-1.02762827	5.26211968	6.28974795
204630_s_at	GOSR1	NM_004871	9527	Hs.462680	-1.027613935	9.748774631	10.77638857
1570177_at		BC026287			-1.027409786	5.687021764	6.714431551
208930_s_at	ILF3	BG032366	3609	Hs.465885	-1.026860908	10.65653009	11.683391
232035_at	HIST1H4B	BE740761	8366	Hs.143080	-1.026736376	6.712140035	7.738876411
229921_at		BF196255			1.026429107	7.511140563	6.484711455
242558_at		AW362945			-1.025306555	6.662712125	7.68801868
205210_at	TGFBRAP1	NM_004257	9392	Hs.446350	-1.025295507	7.30635727	8.331652778
1554417_s_at	APH1A	AY113699	51107	Hs.108408	-1.025206147	7.535066033	8.560272181
235435_at	AASDH	BF433048	132949	Hs.104347	-1.024868816	8.922872388	9.947741205
235067_at	MKLN1	D81987	4289	Hs.44693	-1.024627457	7.481443818	8.506071275
238405_at		AI792896			1.024486493	6.371306943	5.34682045
219992_at	TAC3	NM_013251	6866	Hs.9730	1.024285157	6.251350246	5.227065089
232781_at		AA708488			-1.023990964	5.78003611	6.804027074
205594_at	ZNF652	NM_014897	22834	Hs.463375	-1.023713275	6.406000633	7.429713908
243603_at		AI973041			1.023660919	7.645802808	6.62214189
239757_at	ZFAND6	AA741493	54469	Hs.719106	-1.023611402	6.163908933	7.187520335
212151_at	PBX1	BF967998	5087	Hs.557097	-1.022862791	8.235255071	9.258117862
206997_s_at	HS6ST1	NM_004807	9394	Hs.512841	1.022862765	6.004385685	4.981522919
232865_at	AFF4	N59653	27125	Hs.519313, Hs.664840	-1.022589455	5.452597077	6.475186532
232125_at		AU147419			-1.022559928	7.994139929	9.016699857
218496_at	RNASEH1	BG534527	246243	Hs.568006	-1.022493594	9.394744704	10.4172383
201182_s_at	CHD4	AI761771	1108	Hs.162233	-1.022110436	8.761544903	9.783655339
216990_at	GART	AF008655	2618	Hs.473648	-1.021960513	6.996008643	8.017969156
213345_at	NFATC4	AI624015	4776	Hs.77810	1.021436881	6.221839177	5.200402296

218430_s_at	RFX7	NM_022841	64864	Hs.282855	-1.021378262	7.55681616	8.578194421
223159_s_at	NEK6	BC000101	10783	Hs.197071	1.021105326	10.80440294	9.783297609
201702_s_at	PPP1R10	AI492873	5514	Hs.106019	-1.021005666	10.6734518	11.69445747
223476_s_at	C12orf65	AW007238	91574	Hs.319128	-1.020971891	8.86684118	9.88781307
229824_at		AL133706			1.020627076	7.780355205	6.759728129
242916_at	CEP110	AA642477	11064	Hs.653263	-1.020528607	7.193160223	8.21368883
227259_at	CD47	BF439618	961	Hs.446414	-1.020338999	5.935928755	6.956267754
230980_x_at		AI307713			1.020326312	6.021736543	5.001410231
231710_at	CAPS	AI923108	828	Hs.584744	1.019966435	7.031551271	6.011584836
243834_at	TNRC6A	BF507964	27327	Hs.655057	-1.019869219	7.680039557	8.699908776
237986_at		AI076355			1.019835089	6.15864829	5.138813201
202616_s_at	MECP2	AI631140	4204	Hs.200716	-1.019354649	7.399571655	8.418926304
214951_at	SLC26A10	AL050358	65012	Hs.159481	1.019262935	6.512613691	5.493350756
212341_at	YIPF6	AA195936	286451	Hs.700646, Hs.82719	-1.01905732	9.182096186	10.20115351
1568879_a_at	LAMA3	BC033663	3909	Hs.436367	1.018992587	7.46085278	6.441860193
211844_s_at	NRP2	AF022859	8828	Hs.471200	-1.018927498	5.03669572	6.055623218
230232_at	KIAA0746	AW340510	23231	Hs.479384	-1.01842615	5.191786416	6.210212567
226543_at	MUTED	AI768114	63915	Hs.719272	-1.018307609	7.077226584	8.095534193
225565_at	CREB1	AA769455	1385	Hs.516646	-1.01819817	7.622837592	8.641035762
201211_s_at	DDX3X	AF061337	1654	Hs.719127	-1.017897183	9.186105794	10.20400298
228830_s_at	ATF7	AI279868	11016	Hs.12286	-1.017895218	6.374619252	7.392514471
229430_at		AI421311			1.017644694	6.715875591	5.698230897
228375_at	IGSF11	BE221674	152404	Hs.112873	-1.016430113	5.180040149	6.196470262
213109_at	TNIK	N25621	23043	Hs.34024	-1.016380255	5.619899754	6.636280009
1569061_at	IQGAP3	BC033549	128239	Hs.591495	1.01620259	6.167510943	5.151308353
1554864_a_at	SDC3	AF248634	9672	Hs.158287	-1.015993128	5.208556986	6.224550113
240230_s_at	LOC642826	AW000942	642826	Hs.463017, Hs.647204, Hs.71	-1.015965488	5.968574787	6.984540275
213823_at	HOXA11	H94842	3207	Hs.249171	1.015934847	6.414859641	5.398924794
214434_at	HSPA12A	AB007877	259217	Hs.654682	1.015821645	9.692057854	8.67623621
242405_at		BF358386			-1.01578618	5.929049972	6.944836152
224046_s_at	PDE7A	U67932	5150	Hs.527119	-1.01575698	6.428143986	7.443900966
244790_at	MTCP1	AA744518	4515	Hs.6917	1.014897546	6.779765285	5.764867739
239451_at		AI684643			-1.014350469	5.644148481	6.65849895
220941_s_at	C21orf91	NM_017447	54149	Hs.293811	1.014298256	10.18404739	9.16974913
219507_at	RSRC1	NM_016625	51319	Hs.591283	-1.014110424	9.830114305	10.84422473
208545_x_at	TAF4	NM_003185	6874	Hs.18857	-1.01362444	5.509942324	6.523566764
239635_at		BF510708			-1.013568256	7.033255087	8.046823344
213805_at	ABHD5	AI692428	51099	Hs.19385	-1.013373926	8.095431179	9.108805105
228054_at	TMEM44	BF593660	93109	Hs.478729	1.013296068	10.12899998	9.115703916
236474_at		AI797677			-1.012768683	5.403761619	6.416530303
206723_s_at	LPAR2	AF011466	9170	Hs.122575	1.012606327	7.275394456	6.262788129
1554452_a_at	C7orf68	BC001863	29923	Hs.710088	-1.012290578	7.714002175	8.726292753
232637_at	2-Sep	AK025163	4735	Hs.335057, Hs.716403	-1.012210597	5.015339604	6.0275502

220583_at		NM_025086			1.012201775	6.026901621	5.014699846
219183_s_at	CYTH4	NM_013385	27128	Hs.170944	1.01209571	6.666234547	5.654138837
236328_at	ZNF285A	AW513227	26974	Hs.709428	1.011830667	8.51443873	7.502608062
236229_at		AW014345			-1.011639565	6.837801263	7.849440827
238299_at		AW005866			-1.011605433	5.69221533	6.703820763
228263_at	GRASP	AI190755	160622	Hs.407202	1.011518571	7.384380724	6.372862153
213865_at	DCBLD2	AI378788	131566	Hs.203691	-1.010882735	9.532967741	10.54385048
243286_at		AA682674			-1.010461201	6.665000057	7.675461258
236172_at	LTB4R	AW206817	1241	Hs.567248	1.010222971	7.541767062	6.531544091
210990_s_at	LAMA4	U77706	3910	Hs.654572	1.010125693	6.271394397	5.261268704
210359_at	MTSS1	AF116674	9788	Hs.336994, Hs.700429	-1.009633309	5.384359866	6.393993175
40562_at	GNA11	AF011499	2767	Hs.650575, Hs.654784	-1.009517305	7.3260653	8.335582606
239759_at		AV648418			-1.009044807	5.216420156	6.225464963
229387_at	LRCH3	BF514834	84859	Hs.518414, Hs.659335	-1.008952963	6.314597975	7.323550938
222463_s_at	BACE1	AF190725	23621	Hs.504003	-1.008705763	7.00403905	8.012744812
235727_at	KLHL28	AI219490	54813	Hs.653206	-1.008557742	8.240173089	9.248730831
204181_s_at	ZBTB43	T90308	23099	Hs.355581, Hs.718657	-1.00828226	9.754812538	10.7630948
238882_at		BF000697			-1.008099218	6.137764513	7.145863731
1563796_s_at	EARS2	AK095998	124454	Hs.620541	1.007842279	8.680204488	7.672362209
221528_s_at	ELMO2	BC000143	63916	Hs.210469	1.007837175	10.0433166	9.03547942
209162_s_at	PRPF4	U82756	9128	Hs.530284	-1.007751293	9.654569761	10.66232105
227748_at	CCBL2	AI971694	56267	Hs.481898	-1.007265375	8.727966525	9.7352319
226109_at	C21orf91	AK023825	54149	Hs.293811	1.007262685	10.68377173	9.676509048
230885_at	SPG7	BE670386	6687	Hs.185597	-1.007067449	7.394027769	8.401095219
210935_s_at	WDR1	AF274954	9948	Hs.128548, Hs.713658	-1.006708698	6.928176039	7.934884737
241941_at		AA778747			1.006167297	7.286921911	6.280754614
233230_s_at	SLAIN2	AV723459	57606	Hs.479677	-1.005978408	5.342916138	6.348894546
203699_s_at	DIO2	U53506	1734	Hs.202354	-1.005729753	5.980203053	6.985932806
239672_at		AW960100			-1.005536728	8.129773367	9.135310095
212009_s_at	STIP1	AL553320	10963	Hs.337295	-1.005245097	11.14339185	12.14863695
211360_s_at	ITPR2	AB012610	3709	Hs.512235	-1.005151144	5.395225213	6.400376356
242276_at		T78081			-1.005066252	6.406286226	7.411352479
1562925_at		BC012753			-1.004946914	5.405317975	6.410264889
223795_at	TSPAN10	AF325213	83882	Hs.208219	1.004766023	6.085954481	5.081188458
1556434_at		BQ777552			-1.004469536	7.324179564	8.328649101
239193_at	FUBP3	BF060981	8939	Hs.673029, Hs.98751	-1.003970812	5.43756036	6.441531172
1560017_at	TMTC3	AK074973	160418	Hs.331268	-1.003877522	9.527462993	10.53134051
233022_at	TRPM3	AF325212	80036	Hs.47288	1.003450174	6.861526528	5.858076354
243154_at		AA215381			1.002883862	7.667083068	6.664199206
220165_at	INO80D	NM_017759	54891	Hs.445036	-1.00269869	5.956439789	6.959138479
214628_at	NHLH1	M96739	4807	Hs.30956	1.002289454	7.928845261	6.926555806
219476_at	C1orf116	NM_024115	79098	Hs.32417	1.001508254	6.347075652	5.345567398
205731_s_at	NCOA2	AW027474	10499	Hs.446678	-1.001195678	5.323981076	6.325176754

232300_at		AL157440			1.001180007	7.544291683	6.543111676
228152_s_at	DDX60L	AK023743	91351	Hs.535011	1.00101736	9.709025442	8.708008083
232396_at		AV711227			-1.000976761	6.125312722	7.126289484
234156_at		AK026905			1.000109877	8.055355871	7.055245993
1561908_a_at	HS3ST3B1	AL832823	9953	Hs.48384	-1.000093282	5.725954384	6.726047666
