# Supplementary material for: MicroRNA93 Regulates Proliferation and Differentiation of Normal and Malignant Breast Stem Cells
Source: PLoS Genet. 2012 Jun 7;8(6):e1002751. doi: 10.1371/journal.pgen.1002751 (PMC3369932; doi:10.1371/journal.pgen.1002751)
Supplement: Table S2 — mir93 direct targets in SUM159 cells. Overlap between mir93 predicted targets from TargetScan 5.1 and profiling data from DOX-treated cells (DOX) to non-DOX-treated cells (CTRL) in the ALDH− population (12 genes) or in the ALDH+ population (352 genes). Known stem cell regulatory genes highlighted in red. Genes underlined and bolded were analyzed utilizing the luciferase reporter assay. (PDF) [file pgen.1002751.s027.pdf]

SUM159-mir93	
**ALDH- (DOX vs CTRL)**	**ALDH+ (DOX vs CTRL)**
ARHGEF10, PDGFRA, HMGA2, KIAA0494, POLQ, KLF11, RAB8B, NPAS3, CHAF1A, POLR3G, SR140, CNOT6L	**STAT3**, JAK1, CCND1, ITCH, EZH1, **AKT3**, **SOX4**, HMGA2, BCL2L11, RBL1, TXNIP, SKI, RPS6KA5, HLF, NCOA3, BMPR2, RB1, NPAT, RBL2, TCF4, PKN2, CSNK1G1, E2F2, THRA, DIP2A, FAM126B, ARHGEF10, EPB41, EPHA7, TACC1, LPGAT1, AFF4, CAPRIN2, LUZP1, AHNAK, ACBD5, LAMA3, MKL2, SPTBN1, CALD1, FXR1, KIAA0494, RYBP, TBL1X, FURIN, GRLF1, MECP2, PLAGL2, RAPGEF2, SRPK2, ZNF148, CEP57, CHAF1A, RAPGEF1, GOSR1, EEA1, MPDZ, SEMA5A, ZNF264, EPHA4, ELK3, POLR3G, MXD1, NKX3-2, SLC4A7, NFAT5, RUNX1, TANC2, PDE3B, BICD2, KLHL20, TRIP11, RNF6, LYST, SLC9A2, NCOA3, NFIB, PDLIM5, AFF1, DYNC1LI2, TMCC1, PTPRD, SKI, DCBLD2, TWF1, ARID4B, NHLH1, ZBTB33, MGEA5, NFAT5, CALD1, LYST, AFF1, TSC22D2, RAPGEF2, SIRPA, MLL4, FAM129A, UBE2W, FNDC3B, VANGL1, TMEM50B, ZBTB44, ZNF532, UNKL, LIMA1, TNKS2, TMEM50B, ARID4B, MYLIP, MAPK1, ZFP91, ANKIB1, BTBD7, C5orf41, ARHGAP26, ZFP91, ZNF800, IRF2BP2, PLEKHA3, FMNL3, MYLIP, SPTBN1, NPAS3, MAT2B, CHAF1A, CDCA7, PKN2, TNRC6B, BMPR2, GLT8D3, CREB5, AFF4, MLL3, MAP3K2, REEP3, ITCH, RAB12, MKLN1, ATAD2, AHNAK, CHD9, MAP3K8, KLHL28, MLL3, RAB12, SR140, TNRC6B, DUSP8, OTUD4, AHNAK, KLF12, FNDC3A, SLC30A7, ZFYVE16, LASS6, FGD4, JUB, AFF4, TNRC6A, USP32, ABHD2, MEX3D
