# Supplementary material for: MicroRNA93 Regulates Proliferation and Differentiation of Normal and Malignant Breast Stem Cells
Source: PLoS Genet. 2012 Jun 7;8(6):e1002751. doi: 10.1371/journal.pgen.1002751 (PMC3369932; doi:10.1371/journal.pgen.1002751)
Supplement: Table S3 — Downregualted probe set in ALDH− population from DOX vs. ALDH− population from CTRL. (PDF) [file pgen.1002751.s028.pdf]

Downregualted probe set in ALDH- DOX vs. CTRL

Probe	Symbol	GenBank	Gene	UniGene	Fold Change	DOX ALDH-
204114_at	NID2	NM_007361	22795	Hs.369840	4.010527511	9.977249254
230036_at	SAMD9L	BE669858	219285	Hs.489118	2.43456334	9.01219676
231644_at		AW016812			-2.28442119	3.760793278
226603_at	SAMD9L	BE966604	219285	Hs.489118	2.253728547	8.839771326
242477_at	TTC39B	BF056282	158219	Hs.563630	2.242497192	7.368197941
1559722_at		BC042892			-2.221829239	5.229921317
217332_at	RP11-159J2.1	AL133018	647288	Hs.567920	2.174029493	6.139308902
235643_at	SAMD9L	BE886225	219285	Hs.489118	2.135736034	8.625327442
211548_s_at	HPGD	J05594	3248	Hs.596913	2.12649599	9.413755752
244610_x_at		AA572726			-2.115147586	4.848868296
230607_at		T86874			-2.099198155	5.01923339
236826_at	TTC39B	H83092	158219	Hs.563630	2.095892213	6.064166248
1565701_at		AL832624			-2.046879451	4.971579139
230332_at	ZCCHC7	AA872187	84186	Hs.654700	-2.043889025	5.360124356
215206_at		AK025143			-2.017442549	5.214683376
214295_at	KIAA0485	AW129056	57235	Hs.604754	-1.990997056	5.707951367
1556865_at		AF143885			-1.984807195	5.805319923
233401_at		BF723605			-1.967534559	4.463644502
228367_at	ALPK2	BE551416	115701	Hs.628152	1.963883128	8.521473284
232565_at		AK025052			-1.9460451	5.904248272
232882_at		AA079839			-1.940352935	5.955747586
238558_at		AI445833			-1.931886747	6.998535735
1553155_x_at	ATP6V0D2	NM_152565	245972	Hs.436360	1.921061049	8.695275418
230850_at		AI652647			-1.919355579	4.681656603
232333_at		AU147805			-1.918927244	5.337175905
215898_at	TTLL5	AK021879	23093	Hs.709609	-1.916386887	4.618970142
235705_at		BF676361			-1.87895106	7.100564992
239946_at		AA776723			-1.87398213	5.530788182
203914_x_at	HPGD	NM_000860	3248	Hs.596913	1.847250176	9.124719435
1556352_at		AI692624			-1.842119819	5.638534852
243993_at		AA436887			-1.840232533	4.852075649
242188_at		AI743332			-1.837797297	4.794329111
242471_at		AI916641			-1.827734629	5.6611017
1569727_at		BC028245			1.81512361	7.18984308
241960_at	CSMD1	AA705177	64478	Hs.571466	1.807335235	7.526109148
216628_at		AL117447			-1.806715435	4.570919
215889_at	SKIL	X15217	6498	Hs.581632	-1.792590443	4.300606165
243874_at	LPP	AI079544	4026	Hs.444362	-1.7892799	5.953401452
244414_at		AI148006			-1.784667992	5.612596379
232264_at		AK022204			-1.781948978	7.101680259
1553153_at	ATP6V0D2	NM_152565	245972	Hs.436360	1.781658898	8.643414057
235811_at		AW590853			-1.761472083	5.217772631
241391_at		AA654772			-1.75172268	4.495276984
236987_at	ALPK2	AI741514	115701	Hs.628152	1.75078243	7.744788588
1564378_a_at		AK025101			-1.73803763	5.390712969
239965_at	LOC151878	AW009761	151878	Hs.680377	-1.733473828	5.863726509
1559723_s_at		BC042892			-1.730407817	4.740115765
241245_at	SFRS4	AV647470	6429	Hs.469970	-1.7243532	5.909951144

1562330_s_at	CSMD1	AB067477	64478	Hs.571466	1.722298982	7.416253855
236010_at		AI373107			-1.716358291	4.38815632
224235_at		AF119853			-1.707169413	5.131655531
236134_at	DCAF7	AA769995	10238	Hs.410596	-1.705251892	5.144583198
243271_at		AI064690			1.69933647	6.51387498
81737_at	NPIPL3	AI424872	23117	Hs.552700, Hs.6	-1.694763082	5.280099879
235912_at		BE552155			-1.691760243	5.026105519
237310_at		AI743607			-1.690836941	6.370005181
215802_at		AK000144			-1.682369067	4.715546557
1565689_at		BG400570			-1.680952426	5.211456217
242405_at		BF358386			-1.680614987	5.797690185
232307_at		AK021554			-1.67155057	5.119570994
227838_at		AW070250			1.667526065	7.092993757
238774_at		AW960454			-1.656047306	5.641757396
1559491_at		AL390180			-1.637091221	5.986215604
236355_s_at		AI076172			-1.636515011	5.452618512
206548_at		NM_024880			-1.621858313	4.752670264
202772_at	HMGCL	NM_000191	3155	Hs.533444, Hs.6	1.613732346	10.46353821
1560109_s_at	NUB1	AA193477	51667	Hs.647082	-1.61217186	4.427439129
237456_at		AI655806			-1.601058632	5.552052246
232000_at	TTC39B	AW001030	158219	Hs.563630	1.594870215	6.592156687
241214_at		AI939471			-1.594027526	6.427490364
1553151_at	ATP6V0D2	AY079172	245972	Hs.436360	1.593822177	6.127799328
242732_at		BG010493			-1.591835851	5.392188124
239512_at	SFRS4	R05895	6429	Hs.469970	-1.582467727	6.288944841
229281_at	NPAS3	N51682	64067	Hs.657892	1.577868541	7.043269326
215078_at	SOD2	AL050388	6648	Hs.487046	-1.570939987	8.567688082
203913_s_at	HPGD	AL574184	3248	Hs.596913	1.567976068	6.925410339
235757_at		AA814006			-1.567429339	5.477107509
233674_at		AK026286			-1.544754952	6.659942388
239264_at		AW973078			-1.534596082	5.022928547
240263_at		N74924			-1.527998042	5.414833397
232615_at		AA632758			-1.526607253	7.56380803
222087_at	PVT1	AW451806	5820	Hs.133107, Hs.6	-1.520964032	6.506095299
209200_at	MEF2C	AL536517	4208	Hs.653394	1.511102888	10.07206684
234605_at	CDC14B	AK024886	8555	Hs.40582	-1.506428939	5.25551146
1559156_at		BC036508			-1.493012385	7.32083969
242114_at	BOLA2	BF088991	552900	Hs.444600, Hs.6	-1.485496393	5.842246534
239414_at		BF942260			-1.472552963	7.416295721
243088_at		W84667			-1.462678259	6.463625368
236976_at	FANCA	AI569792	2175	Hs.719210	-1.462081352	5.850309365
1568846_at		BC017718			-1.460480063	8.251496935
235786_at		AI806781			-1.458479649	4.855168606
1562144_at		AF075103			-1.456709094	4.707724893
1556672_a_at	RBM6	AI190489	10180	Hs.439480	-1.447891544	7.135064691
242859_at		BE156563			-1.446764951	6.658800272
1558714_at		BC043430			-1.446140631	5.239233791
242886_at		AW007763			-1.445700255	6.777535898
242143_at		BE674964			-1.433048473	5.196505471
1557718_at	PPP2R5C	AL834350	5527	Hs.368264, Hs.7	-1.426546004	5.463246136

241457_at		AI821935			-1.425353889	5.156465629
238883_at		AW975051			-1.421705845	5.662421595
235680_at		AI914925			-1.421080784	5.485447387
242038_at	LRRC8B	BG037106	23507	Hs.482017, Hs.6	-1.415413841	5.505180003
239227_at		AW182675			-1.413753058	5.98710379
227266_s_at	FYB	BF679849	2533	Hs.370503	1.408725618	6.327739611
1558410_s_at		AW974642			-1.40857607	5.886075734
1569208_a_at		BC020895			-1.408207411	5.257810203
1564886_at		AL359595			-1.405432263	6.67497196
206408_at	LRRTM2	NM_015564	26045	Hs.656653	-1.400483571	4.79290933
239908_at		AA496799			-1.400434197	7.224845963
243003_at		AV702197			-1.400193546	7.451847543
1560680_at		AL833513			-1.391417609	6.415653397
229858_at		AU146893			-1.391347821	6.616081109
242476_at		AI436356			-1.389218591	5.897007174
240798_at		BE467916			-1.384782965	4.989657664
231205_at		BF055351			-1.377684726	5.525099202
243474_at		W87425			-1.374184785	4.875604885
235847_at		BF111312			-1.37376709	5.635878127
234095_at		AU155112			-1.371839766	6.22693389
228623_at		AI224133			-1.366709103	7.92092966
238619_at		AA417078			-1.365610656	6.878220062
228573_at	ANTXR2	BE673665	118429	Hs.162963	1.365158879	9.950818451
210109_at	C7orf54	AF191492	27099	Hs.657377	-1.364271879	5.85157939
236216_at		AA598661			-1.363306066	6.151186082
241932_at		AI073803			-1.355742401	5.365774836
239348_at		AI285970			-1.352580885	5.746394193
243149_at		AI467945			-1.350407784	4.716783739
209199_s_at	MEF2C	N22468	4208	Hs.653394	1.349690435	9.634230601
1567223_at	HMGA2	U29113	8091	Hs.505924	-1.34327983	5.283316032
242868_at		T70087			-1.342577424	5.356776997
231552_at		AW451785			-1.341249019	6.1933929
240499_at		AA482221			-1.339816138	4.765993075
202720_at	TES	NM_015641	26136	Hs.592286, Hs.6	1.335902451	7.010780215
1564798_at		AK000811			-1.332835723	4.971195426
223557_s_at	TMEFF2	AB017269	23671	Hs.144513	1.331031212	9.443686909
241372_at	ZC3H6	R34135	376940	Hs.190477	1.319913899	6.21684905
1558385_at		AL832806			-1.318738931	5.92696848
244682_at	CAMSAP1	H11471	157922	Hs.522493	-1.315917022	5.537793623
1560476_at		AF290476			-1.315897403	5.363632866
1558569_at	UNQ6228	AL832308	100131541	Hs.661972	-1.311239402	6.732864364
206638_at	HTR2B	NM_000867	3357	Hs.421649	-1.310845368	6.300984803
239567_at		AW974998			-1.307590707	5.166559629
238544_at		AA618295			-1.305567618	5.270044868
239519_at		AA927670			-1.305373616	6.949290327
240156_at		AA417099			-1.295776483	4.913869577
242688_at		AI149880			-1.294211695	8.189253645
236947_at		AI686664			-1.287292079	5.387679882
1569538_at		AF130084			-1.287115	5.635878127
235804_at		T86613			-1.287115	5.961730608

242983_at		AI806626			-1.284876621	6.055331801
222145_at		AK027225			-1.282472469	4.936361761
234159_at		AK025182			-1.280392834	6.1933929
242126_at		T53962			-1.279693914	5.073080785
239274_at		AV729557			-1.276573674	5.289077341
244473_at		R37637			-1.274649646	6.19136422
242240_at		AI805700			-1.27052649	7.981482171
1554213_at	ARHGEF10	BC036809	9639	Hs.98594	-1.269774342	6.235483101
1555074_a_at	KCNH5	BC043409	27133	Hs.27043	-1.268741856	5.152148854
230590_at		BE675486			-1.256709358	6.36043427
220969_s_at		NM_030892			-1.255823081	5.873399357
242068_at		AA608834			-1.255091279	6.334370986
243670_at		BE670161			-1.254393993	6.298453529
219210_s_at	RAB8B	NM_016530	51762	Hs.389733	1.252531426	8.896902106
1557539_at		BC008052			-1.249058254	5.257753161
243827_at		AL038125			-1.246508175	5.090613928
228885_at	MAMDC2	AI862120	256691	Hs.547172	1.244099341	7.938658868
222180_at		AU147889			-1.2424408	4.878445785
231199_at		AA701676			-1.239247755	4.97295984
217482_at		AK021987			-1.239191413	6.746678136
241681_at		AW296451			-1.237660534	8.523250339
241797_at		AI904095			-1.233064386	5.574602342
242492_at	CLNS1A	AA412065	1207	Hs.430733, Hs.5!	-1.230846072	6.81827937
227443_at	C9orf150	AI972386	286343	Hs.445356	1.230363171	9.490543805
232002_at		AW843302			-1.227981951	7.165781121
213606_s_at	ARHGDIA	AI571798	396	Hs.159161	-1.227016296	7.92480366
239957_at		AW510793			-1.226766046	5.674927094
1562059_at		AW020871			-1.223453938	4.989517584
212070_at	GPR56	AL554008	9289	Hs.513633	1.223395523	9.650393353
207746_at	POLQ	NM_014125	10721	Hs.241517	-1.22320161	5.035558454
231223_at	CSMD1	R41565	64478	Hs.571466	1.222024021	10.21594423
238563_at		AV762916			-1.220644296	5.629190794
215318_at	CG012	AL049782	116829	Hs.687692	-1.218691805	5.088582525
232835_at		AA533080			-1.216728418	6.538249517
1558599_at		AA532745			-1.216018231	4.930059765
229013_at	LOC145783	BF111029	145783	Hs.620525	1.210304432	6.928918182
234224_at		AL137541			-1.209278201	5.029854733
239238_at		AI208857			-1.209129626	6.872820177
240787_at		AW449433			-1.209108521	5.429919799
238172_at		AA192765			-1.205867076	6.452511679
239448_at		AI475033			-1.204860943	5.958505685
1569664_at		BC035915			-1.204625094	5.889268693
241762_at	FBXO32	BF244402	114907	Hs.403933	-1.201540977	5.216605613
214215_s_at	LARP4B	AW514174	23185	Hs.681734, Hs.7	-1.201398131	5.011255105
241320_at		AI821449			-1.19843653	6.438203212
1557597_at		BC036212			-1.197701235	5.270676936
241301_at		AA694187			-1.194646051	5.847261909
225345_s_at		AU155376			-1.191962436	7.13975899
239469_at		BF513404			-1.191351279	6.084684925
1552829_at		NM_018505			-1.186675757	5.871886366

239923_at		AI056872			-1.185689302	5.263571335
232465_at		AK021749			-1.184736202	5.814342576
215979_s_at	SLC7A1	AK022999	6541	Hs.14846	-1.179773588	7.40288594
240302_at		AW450681			-1.176980078	5.624145396
232134_at		AW139789			-1.173025017	8.52800424
1570108_at		BC037848			-1.167714757	6.111813249
230099_at		AI139993			-1.16735959	5.646997613
213556_at	LOC390940	BE673445	390940	Hs.22049	1.165040076	8.365901634
232711_at		AU146197			-1.163456902	7.171100511
239606_at		AA669135			-1.161466094	6.667950381
242343_x_at		H57111			-1.161073721	6.334415364
232592_at		AU146731			-1.160449332	6.553674955
231343_at		AW300131			1.157345144	9.058293683
232778_at		AK026036			-1.157341022	6.426497012
244457_at		BF724206			-1.157010166	5.497985021
231950_at	ZNF658	AW874634	26149	Hs.522147	1.154698581	6.332787832
1566079_at	RPS16P5	AL833001	647190	Hs.675022	-1.154158212	5.740014468
235454_at		AI436561			-1.154153809	5.997101564
238438_at	CNOT6L	R67226	246175	Hs.592519	-1.148244828	4.950103549
222453_at	CYBRD1	AL136693	79901	Hs.715531	-1.146416232	8.898566249
1570329_at		BC014497			-1.145979865	5.440914832
220941_s_at	C21orf91	NM_017447	54149	Hs.293811	1.144823601	9.391385285
240247_at		AI653240			-1.141014777	7.761099423
222282_at		AV761453			-1.140469185	7.165076325
205933_at	SETBP1	NM_015559	26040	Hs.435458	1.137791562	8.388530219
244347_at		BE825318			-1.130701943	6.342499118
240008_at		AI955765			-1.12656662	5.436576671
1560926_at		AF085924			-1.126257237	7.358212315
239757_at	ZFAND6	AA741493	54469	Hs.719106	-1.125568584	5.935947012
240257_at	SYNJ2	AI971212	8871	Hs.434494	-1.124140779	6.128558185
241893_at		BE927766			-1.123412459	5.081078414
233315_at		AK024947			-1.123186393	5.934117093
243768_at		AA026388			-1.122398537	7.346260473
201775_s_at	KIAA0494	AA676790	9813	Hs.719205	-1.120432801	8.679352994
244356_at		AL079909			-1.11896753	7.54442516
237377_at		AA069425			-1.11873928	4.94904123
236346_at		BF115793			-1.118574924	7.480749926
233921_s_at		AK022078			-1.118204719	7.089373219
236595_at		AA776458			-1.117894154	6.201153958
1569477_at		BC025999			-1.116530721	6.40072714
1567224_at	HMGA2	U29113	8091	Hs.505924	-1.115914833	4.921529594
220410_s_at	CAMSAP1	NM_018627	157922	Hs.522493	-1.115500301	6.264168824
238706_at	PAPD4	BG168850	167153	Hs.418198	-1.114722037	5.152322974
240248_at		AA778783			-1.11255464	6.328362658
243589_at	KIAA1267	AI823453	284058	Hs.648744	-1.11165161	6.383715285
228484_s_at	FOXO1	AI472322	2308	Hs.370666	-1.111237419	6.837802011
227478_at	SETBP1	BF739885	26040	Hs.435458	1.110884956	8.985904131
242449_at		BG054682			-1.106983063	5.823983663
1555854_at		AA594609			-1.10647736	8.971570236
244358_at		AW372457			-1.105973871	7.742020115

244633_at		AA404996			-1.105410517	5.918017712
239892_at		AW593666			-1.104384516	6.780422129
1556240_at		AI339498			-1.103723408	5.218451371
233800_at		AA805082			-1.102331772	5.4661987
239597_at		AA993566			-1.10086361	5.862675207
231106_at	BMS1P5	AI684591	399761	Hs.314437, Hs.7	-1.099573124	6.128646334
1560026_at		BC037944			-1.099214705	5.524458657
235784_at		N32155			-1.099021374	6.815068602
1554828_at	PDGFRA	BC015186	5156	Hs.74615	-1.097961856	5.07584475
214622_at	CYP21A2	M17252	1589	Hs.654479	1.097573344	7.606492748
232889_at		AU147591			-1.094440258	8.367109828
238303_at	STT3B	AW070371	201595	Hs.475812	-1.09326906	7.335209153
1569540_at		BC035958			-1.09101066	5.527228838
232281_at	LOC148189	AU147637	148189	Hs.565253	-1.090717795	6.539560384
209713_s_at	SLC35D1	AB044343	23169	Hs.213642	1.089211267	7.960559402
232500_at	C20orf74	AL121896	57186	Hs.472285	-1.088781113	6.278219266
235138_at		AA565051			-1.088354664	7.37290495
1559992_a_at		BC042069			-1.087822606	4.992877668
228271_at		AW001186			-1.087548533	7.228388535
1563452_at	AVL9	AL833560	23080	Hs.128056	-1.084069576	5.061672855
233323_at		AK024973			-1.081675985	5.204730023
202838_at	FUCA1	NM_000147	2517	Hs.370858	1.080706693	9.673504857
241865_at		AI056689			-1.0791165	6.740928354
210637_at	TACR1	M81797	6869	Hs.633301	-1.078466665	5.443308037
243482_at		BF512299			-1.077411778	5.309752677
234649_at		AK026259			-1.077154157	4.947223404
242875_at		AI659439			-1.076554358	7.470377338
241407_at		BF032023			-1.076173685	6.007137318
1565713_at		H72951			-1.075618195	5.217040913
236696_at	SR140	BE464843	23350	Hs.596572	-1.069780555	6.637092874
230918_at		BE856598			-1.067735875	6.739979575
215515_at		AL049268			-1.062647046	7.576129448
235601_at		AA907029			-1.062162695	5.821844346
243395_at		AI679555			1.060955688	9.399955457
241063_at		BE672556			-1.060951877	6.49910409
243527_at		AW793677			-1.059246806	5.788112868
236379_at		AW771958			-1.058565357	6.423163344
1555675_at	BLID	AF303179	414899	Hs.686109	-1.057171542	5.073032237
241965_at		BF589232			-1.056787238	6.639115245
215378_at		AU148255			-1.056655965	5.705184166
1558836_at		BQ024490			-1.056489458	7.582355504
217626_at		BF508244			-1.056095154	8.923111189
205239_at	AREG	NM_001657	374	Hs.270833	1.05547072	9.554607766
243286_at		AA682674			-1.055338195	6.875364372
222368_at		AW972351			-1.05444063	5.297647502
233370_at		AK024973			-1.053118124	5.171562034
242021_at		AI091255			-1.052613818	5.769086337
237895_at		AV700930			-1.052605949	6.476131211
220609_at	LOC202181	NM_024651	202181	Hs.189914, Hs.6	-1.052409727	5.257186839
236752_at		AA913146			-1.051635541	5.027329914

1558837_a_at		BQ024490			-1.050473399	7.441032706
239630_at		BF516583			-1.050146003	5.516080915
201167_x_at	ARHGDIA	D13989	396	Hs.159161	-1.049566499	7.617389457
223710_at	CCL26	AF096296	10344	Hs.131342	1.049271014	6.611049262
216847_at		AB051447			-1.04908491	4.986806215
1557828_a_at	C5orf28	BE675061	64417	Hs.558531	-1.047886644	5.563789785
225801_at	FBXO32	AW518714	114907	Hs.403933	-1.047126365	5.798112476
243841_at	SYNE2	BE673396	23224	Hs.525392	-1.046810874	10.09210527
243329_at		AI074450			-1.04655799	6.500843641
1559410_at		AA524609			-1.046482241	5.384646157
1557996_at		AK091784			-1.046314684	7.399971766
1552536_at	VTI1A	NM_145206	143187	Hs.194554	-1.042793203	6.758803124
212143_s_at	IGFBP3	BF340228	3486	Hs.450230, Hs.6	-1.042516351	5.815905056
243559_at		BF515306			-1.040851554	5.496492616
227939_s_at	TRA2A	BF439171	29896	Hs.445652	-1.038935183	5.352514263
212076_at	MLL	AI701430	4297	Hs.258855	-1.03715665	9.788740418
233219_at		AK023427			-1.036099006	5.714257651
237184_at		BF110186			-1.034797842	5.938815807
241595_at		BF223007			-1.033654804	6.775580803
217638_at		BF939092			-1.033158346	6.094608551
242664_at		AI040744			-1.0329518	5.412996668
229808_at	CHAF1A	AI344306	10036	Hs.79018	-1.032934867	7.274698443
231005_at		T91195			-1.030972465	6.098992129
213605_s_at	LOC100272216	AL049987	100272216	Hs.631974	-1.030385611	9.580915537
231544_s_at	POLR3G	AV648405	10622	Hs.282387	-1.029960007	5.584176836
230702_at	C8orf16	BE674736	83735	Hs.660259	-1.02951356	6.257649091
229834_at	NFIX	AI937201	4784	Hs.257970	-1.028209581	6.912137531
235112_at		AA088388			-1.026538514	6.363037667
219501_at	ENOX1	NM_017993	55068	Hs.128258, Hs.7	1.026430872	8.948223268
218486_at	KLF11	AA149594	8462	Hs.12229, Hs.69	-1.02620654	7.110302357
232148_at	NSMAF	BF056507	8439	Hs.372000	-1.024429516	5.969213986
1554785_at	CCDC82	BC018663	79780	Hs.525088	1.023811456	6.069041709
232372_at		AL157491			-1.022407576	5.764431944
1565558_at		N67305			-1.022063255	5.037170363
244019_at		T89845			-1.015269514	5.220730327
237502_at	CRLS1	BE671045	54675	Hs.224764	-1.014219094	6.167475831
1560082_at		AL833239			-1.014115425	5.13915711
220310_at	TUBAL3	NM_024803	79861	Hs.163079	1.011679041	8.235933284
220858_at	SORBS2	NM_014133	8470	Hs.619806, Hs.6	-1.011317623	5.215137877
222846_at	RAB8B	AB038995	51762	Hs.389733	1.010417137	7.554947032
1554588_a_at	TTC30B	BC033795	150737	Hs.447659	1.010100207	6.291711915
215470_at		U21915			-1.00960035	9.312242902
233877_at		AK000777			-1.006572539	5.019124044
226109_at	C21orf91	AK023825	54149	Hs.293811	1.006531654	10.31118602
231958_at	C3orf31	AW969235	132001	Hs.475472	-1.005823345	5.669148615
239731_at		AI674685			-1.005226297	8.011383443
239735_at		N67106			-1.0034803	6.076279083
233227_at	KIAA1109	AB037792	84162	Hs.408142	-1.003225191	6.539549407
233037_at		AF138859			-1.001796171	5.57583725
236006_s_at	AKAP10	AU147278	11216	Hs.708043	-1.001690832	5.318559463

233238_s_at		AK001582			1.001598584	8.134957044
204055_s_at	CTAGE5	NM_005930	4253	Hs.509200, Hs.5	-1.001237097	5.254109485
242440_at		N52821			-1.000774854	5.221711764
1558078_at		BQ219651			-1.000023063	5.977879986

CTRL ALDH-
5.96672174
6.57763342
6.04521447
6.58604278
5.12570075
7.45175056
3.96527941
6.48959141
7.28725976
6.96401588
7.11843154
3.96827404
7.01845859
7.40401338
7.23212593
7.69894842
7.79012712
6.43117906
6.55759016
7.85029337
7.89610052
8.93042248
6.77421437
6.60101218
7.25610315
6.53535703
8.97951605
7.40477031
7.27746926
7.48065467
6.69230818
6.63212641
7.48883633
5.37471947
5.71877391
6.37763444
6.09319661
7.74268135
7.39726437
8.88362924
6.86175516
6.97924471
6.24699966
5.99400616
7.1287506
7.59720034
6.47052358
7.63430434

5.69395487
6.10451461
6.83882494
6.84983509
4.81453851
6.97486296
6.71786576
8.06084212
6.39791562
6.89240864
7.47830517
6.79112156
5.42546769
7.2978047
7.62330682
7.08913352
6.37452858
8.84980586
6.03961099
7.15311088
4.99728647
8.02151789
4.53397715
6.98402397
7.87141257
5.46540079
10.1386281
5.35743427
7.04453685
8.20469734
6.55752463
6.94283144
9.09041528
8.02705933
8.56096395
6.7619404
8.81385207
7.32774293
8.88884868
7.92630363
7.31239072
9.711977
6.31364826
6.16443399
8.58295623
8.10556522
6.68537442
8.22323615
6.62955394
6.88979214

6.58181952
7.08412744
6.90652817
6.92059384
7.40085685
4.91901399
7.2946518
6.66601761
8.08040422
6.1933929
8.62528016
8.85204109
7.80707101
8.00742893
7.28622577
6.37444063
6.90278393
6.24978967
7.00964522
7.59877366
9.28763876
8.24383072
8.58565957
7.21585127
7.51449215
6.72151724
7.09897508
6.06719152
8.28454017
6.62659586
6.69935442
7.53464192
6.10580921
5.67487776
6.30403115
8.1126557
4.89693515
7.24570741
6.85371065
6.67953027
8.04410377
7.61183017
6.47415034
6.57561249
8.25466394
6.20964606
9.48346534
6.67497196
6.92299313
7.24884561

7.34020842
6.21883423
7.47378573
6.3527747
6.56565101
7.46601387
9.25200866
7.50525744
6.42089071
7.61714363
7.12922244
7.58946227
7.55284752
7.64437068
6.50681142
6.3371221
6.69455953
6.12088658
6.2122076
7.98586955
9.76091087
6.80766673
8.04912544
8.26018063
8.39376307
9.15181996
6.90169314
6.21297152
8.42699783
6.25876006
8.99392021
6.84983509
6.30727433
7.75497794
6.146078
5.71861375
6.23913293
8.0819498
6.63902832
7.65837875
7.16336663
7.09389379
6.41814659
6.21265324
7.63663974
6.46837817
7.04190796
8.33172143
7.2760362
7.05856212

6.44926064
6.99907878
8.58265953
6.80112547
9.70102926
7.27952801
6.8143572
7.20086156
8.33455741
7.82941648
7.49548909
7.71412429
7.90094854
7.58383803
6.65499519
5.17808925
6.89417268
7.15125537
6.09834838
10.0449825
6.5868947
8.24656168
8.9021142
8.30554551
7.25073866
7.47320106
6.56314329
8.48446955
7.0615156
7.25269896
6.20449087
7.05730349
8.46865901
9.79978579
8.66339269
6.06778051
8.59932485
8.20757794
7.31904811
7.51725786
6.03744443
7.37966913
6.26704501
7.4409173
7.49536689
7.94903943
7.87501918
6.93096673
10.0780476
8.84799399

7.02342823
7.88480665
6.32217478
6.56853047
6.96353882
7.22821946
6.62367336
7.91408998
6.17380661
6.5089194
9.46155009
8.42847821
6.6182395
7.63027818
6.87134813
7.36700038
8.46125961
6.08070027
8.31593707
6.14574243
6.28640601
8.59279816
7.82004485
6.5217747
6.38716446
6.02437756
8.5469317
7.083311
6.29265911
7.70687343
7.80771545
8.63877649
6.88400704
8.33899977
7.56005597
6.84735967
7.4817287
6.13020378
7.69590248
6.76184013
8.63884496
9.97920634
8.49913705
7.93070257
6.35208813
6.22468016
6.82170015
7.52873716
6.30959657
6.07896545

8.4915061
6.56622692
8.66695596
5.56177825
6.03589113
6.61167643
6.84523884
11.1389161
7.54740163
6.4311284
8.44628645
7.80159633
6.85842141
6.53734417
6.39144945
10.8258971
6.75035666
6.97361365
7.80923561
7.1277669
6.44594847
8.30763331
7.12996459
10.6113011
6.61413684
7.28716265
7.94034711
7.38957618
7.9217924
8.1365089
6.9936435
5.04523025
6.78683952
6.05923362
6.23599984
7.18169493
6.15327253
7.22425424
6.2264555
6.54452989
5.28161171
10.3218433
6.02569658
9.30465436
6.67497196
9.01660974
7.07975938
7.5427746
6.57763342
6.32025029

7.13335846
6.25534658
6.22248662
6.97790305
